# Long-read sequencing of single cell-derived melanoma sublines reveals divergent and parallel genomic and epigenomic evolutionary trajectories

**DOI:** 10.1038/s41467-026-75172-9

**Published:** 2026-07-16

**Authors:** Yuelin Liu, Anton Goretsky, Ayse G. Keskus, Salem Malikic, Tanveer Ahmad, E. Michael Gertz, Farid Rashidi Mehrabadi, Michael Kelly, Maria O. Hernandez, Charlie Seibert, Juan Manuel Caravaca, Kayla Kline, Yongmei Zhao, Ying Wu, Biraj Shrestha, Bao Tran, Arindam Ghosh, Xiwen Cui, Antonella Sassano, Laksh Malik, Breeana Baker, Cornelis Blauwendraat, Kimberley J. Billingsley, Sandra Burkett, Eva Perez-Guijarro, Glenn Merlino, Erin K. Molloy, S. Cenk Sahinalp, Chi-Ping Day, Mikhail Kolmogorov

**Affiliations:** 1 Cancer Data Science Laboratory, Center for Cancer Research, National Cancer Institute, National Institutes of Health, Bethesda, MD USA; 2 Department of Computer Science, University of Maryland, College Park, MD USA; 3 Laboratory of Human Carcinogenesis, Center for Cancer Research, National Cancer Institute, National Institutes of Health, Bethesda, MD USA; 4 Center for Cancer Research Single Cell and Spatial Core, Cancer Research Technology Program, Frederick National Laboratory for Cancer Research, Bethesda, MD USA; 5 Center for Cancer Research Sequencing Facility, Cancer Research Technology Program, Frederick National Laboratory for Cancer Research, Frederick, MD USA; 6 Center for Cancer Research, Sequencing Facility Bioinformatics Group, Biomedical Informatics and Data Science Directorate, Frederick National Laboratory for Cancer Research, Frederick, MD USA; 7 Laboratory of Cancer Biology and Genetics, Center for Cancer Research, National Cancer Institute, National Institutes of Health, Bethesda, MD USA; 8 Center for Alzheimer’s and Related Dementias, National Institute on Aging and National Institute of Neurological Disorders and Stroke, National Institutes of Health, Bethesda, MD USA; 9 NCI Molecular Cytogenetics Core Facility, Cancer Research Technology Program, Frederick National Laboratory for Cancer Research, Bethesda, MD USA; 10 Institute for Biomedicine Research Sols-Morreale, Spanish National Research Council-Universidad Autonoma de Madrid (IIBM, CSIC-UAM), Madrid, Spain

**Keywords:** Cancer genomics, Genome informatics

## Abstract

Tumor evolution is driven by various mutational processes, ranging from single-nucleotide variants (SNVs) to large structural variants (SVs) to dynamic shifts in DNA methylation. Current short-read sequencing methods struggle to accurately capture the full spectrum of these genomic and epigenomic alterations due to inherent technical limitations. To overcome that, here we introduce an approach to identify and analyze the genomic and epigenetic events in different stages of tumoral evolution from long-read sequencing of single-cell derived sublines. We then use it to profile 23 sublines of a mouse cutaneous melanoma cell line, characterized with distinct growth phenotypes and treatment responses. We develop a computational framework for harmonization and joint analysis of different variant types in the evolutionary context. Uniquely, our framework enables detection of recurrent amplifications of putative driver genes, generated by independent SVs across different lineages, suggesting parallel evolution. In addition, our approach revealed gradual and lineage-specific methylation changes associated with aggressive clonal phenotypes. We also show our set of phylogeny-constrained variant calls along with openly released sequencing data can be a valuable resource for the development and benchmarking of computational methods.

## Abstract (web summary)

Tumor evolution involves genetic and epigenetic changes that are difficult to resolve with standard sequencing approaches. Here, authors use long-read sequencing of single cell-derived melanoma sublines to map mutations, structural variants and DNA methylation, revealing parallel genomic changes and lineage-specific epigenetic trajectories linked to tumor behavior.

## Introduction

Tumor heterogeneity and evolution are driven by diverse genetic and epigenetic alterations, including single-nucleotide variants (SNVs), structural variants (SVs), copy number alterations (CNAs), and DNA methylation changes^1^. Traditionally, tumor evolution has been conceptualized as the gradual accumulation of point mutations under selective pressures^2^. More recently, distinct mutational processes that lead to the rapid accumulation of structural variants during catastrophic events - such as chromothripsis and chromoplexy - have been increasingly recognized as critical drivers of tumor initiation and adaptation^3,4^. Tumor evolution can also be shaped by non-genetic, or even non-heritable, determinants, especially epigenetic alterations such as DNA methylation or histone acetylation^5^. Profiling all these processes through a holistic approach is essential for improving our understanding of tumor evolution and for designing next-generation treatment strategies^6,7^.

Short-read genome sequencing (e.g., Illumina) has been a workhorse of cancer genomics and has advanced our understanding of cancer heterogeneity and evolution^8^. However, these methods struggle to accurately detect structural rearrangements due to limitations in read mappability^9^ or genomic coverage^10^, and they require error-prone bisulfite conversion to detect epigenetic alterations^11^. As a result, short-read methods provide an incomplete view of the underlying mechanisms of tumor evolution.

Emerging long-read sequencing technologies, such as Oxford Nanopore Technologies (ONT) and PacBio, overcome the limitations of short-read sequencing by offering improved read mappability, direct variant phasing, and simultaneous capture of DNA methylation^12^. Several recent studies have used long-read sequencing to study complex patterns of somatic variation and methylation in various cancers^9,13,14^, but so far mostly at bulk resolution.

To study tumor evolution on the sub-clonal level, single-cell genome sequencing has recently been used to profile variants in individual tumor cells, however the technology is limited by sampling sparsity^10,15^. Alternatively, whole genome sequencing of single-cell derived cultures have been used to precisely trace subclonal differentiation with high sequencing depth^16–18^. So far, all such studies were based on short-read results, thus providing only a partial view of the genomic and epigenomic evolutionary landscape.

Here we introduce an approach based on long-read sequencing of single-cell derived sublines, and apply it to analyze 23 sublines, each derived from a single cell of the B2905 cutaneous melanoma cell line. This cell line was originally generated from a UV-induced cutaneous melanoma in a Hgf-transgenic mouse^19^, and the sublines were previously characterized by differential therapeutic responses^20^. We generate high-quality SNV, SV, CNA, and differentially methylated region (DMR) calls in each subline and develop algorithms to infer their relative timing in distinct lineages through phylogenetic analysis. We profile the dynamic involvement of distinct mutational processes, including UV-induced damage, oxidative stress, and impaired DNA repair, offering deeper insight into their roles across the evolutionary stages of melanoma progression. We also observe recurrent amplifications of oncogenes occurring independently across multiple lineages and driven by distinct rearrangements, suggesting parallel adaptation under evolutionary pressures. Furthermore, we identify lineage-specific methylation trajectories associated with increased aggressiveness in distinct lineages, linking epigenetic changes with gradual phenotypic differentiation in this experimental system. We also release the generated data to encourage the development and benchmarking of computational methods.

## Results

### Long-read sequencing of single-cell derived sublines of B2905 mouse melanoma model

To showcase our long-read approach to profile tumor evolution at a subclonal level, we used the B2905 cell line, derived from the M4 cutaneous melanoma model — previously characterized with high intratumoral heterogeneity and shown in preclinical studies to have a diverse response to immune checkpoint blockade (ICB) therapy^21^. Here, we performed long-read sequencing of 23 single-cell derived sublines of the B2905 cell line, previously isolated for expansion to become individual clonal sublines, and implanted into C57BL/6 mice to observe distinct survival times^20^. It carries a hot-spot Kras mutation and therefore was characterized^21^ as a model of UV-associated RAS-mutated cutaneous melanoma^22,23^. While previous studies also analyzed bulk whole exome (bWES), whole transcriptome, and single-cell RNA sequencing (scRNA-seq) for each of the B2905 sublines^24–26^, the contributions of SVs, CNAs and methylation to the evolution of this model remained unexplored.

We sequenced each subline using a single Oxford Nanopore Technologies (ONT) R10.4 PromethION flow cell. On average, this yielded 69.77 Gbp of data per subline with a mean read N50 of 29.1 Kbp and an average of 82.95% of base pairs with a quality score of 20 + (Supplementary Table S1). For comparison, WES runs on the same sublines yields an average length of 123 bp per read, with a 99.36% of base pairs with a quality score of 20+ (Supplementary Table S2). We note that most of the ONT base errors are indels, previous studies have demonstrated that point mutation calling quality is similar for ONT and Illumina^27^.

As a matching normal sample, we sequenced the splenocytes of an HGF-transgenic C57BL/6N mouse, the genetic background that was used to derive the B2905 model.

**Fig. 1:**
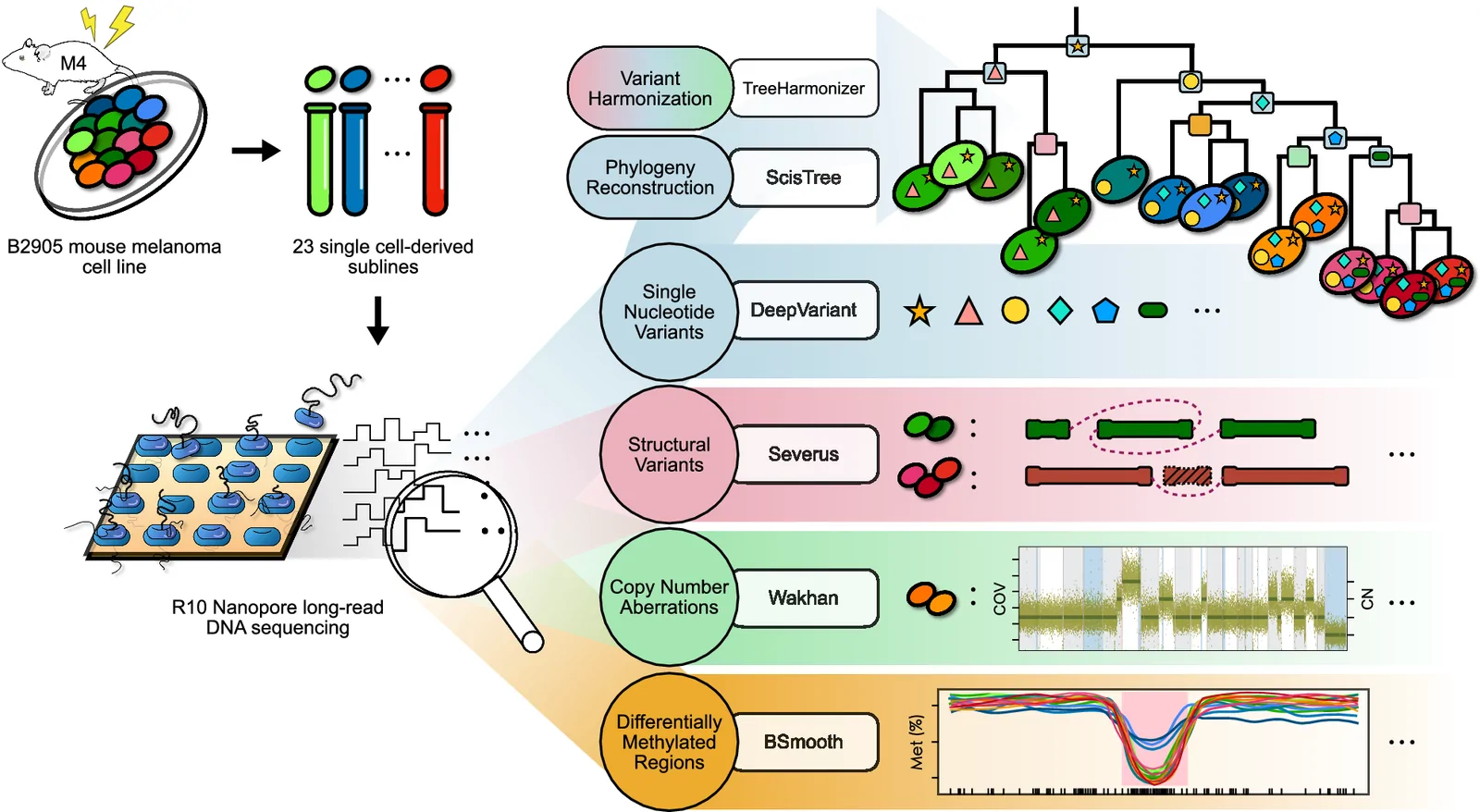
An overview of the long-read approach and analysis of 23 sublines of a mouse melanoma tumor. A tumor subline was cultured from each of the 23 single cells extracted from the B2905 cell line derived from the M4 mouse model of UV-induced melanoma, and the bulk samples were subjected to Nanopore long read sequencing. We studied melanoma tumor evolution, integrating insights from mutation, structural variation, copy number variation, and differential methylation profiled from the sequencing data of the 23 sublines. Specifically, a tumor phylogeny of the sublines was constructed using SNVs, and then other types of genomic and epigenomic events detected by various tools were placed onto the phylogeny. This provides an unprecedented look into melanoma evolution across time and various types of (epi-)genomic alteration events. Mouse graphic from NIH BIOART^129^.

Following basecalling, we aligned the reads from each subline against the GRCm38 reference genome. To generate somatic SNVs, we used DeepVariant^28^, and subtracted germline variants called from the normal sample (Methods). For SV calls, we used Severus^9^ in multi-sample mode (Methods). Since the mouse strain that derived the B2905 cell line was generated by out-crossing HGF-transgenic FVB mice with C57BL/6 mice for over 10 generations, for SNV and SV variants, we subtracted known germline variants of the FVB mouse strain (Methods). Finally, we used Wakhan^29^ for CNA calling, which uses SV breakpoints as the boundaries of CNA segments and fits a model integer copy number profile (Fig. 1). In addition, 5mC base modifications were profiled directly from the sequencing data, enabling the detection of DMRs using BSmooth^30^, which we will discuss in further detail in a subsequent section. Together, these identified variants serve as the basis of the multifaceted analyses we will describe in the following sections.

### Constructed phylogeny reveals phenotypically-distinct major clades

**Fig. 2:**
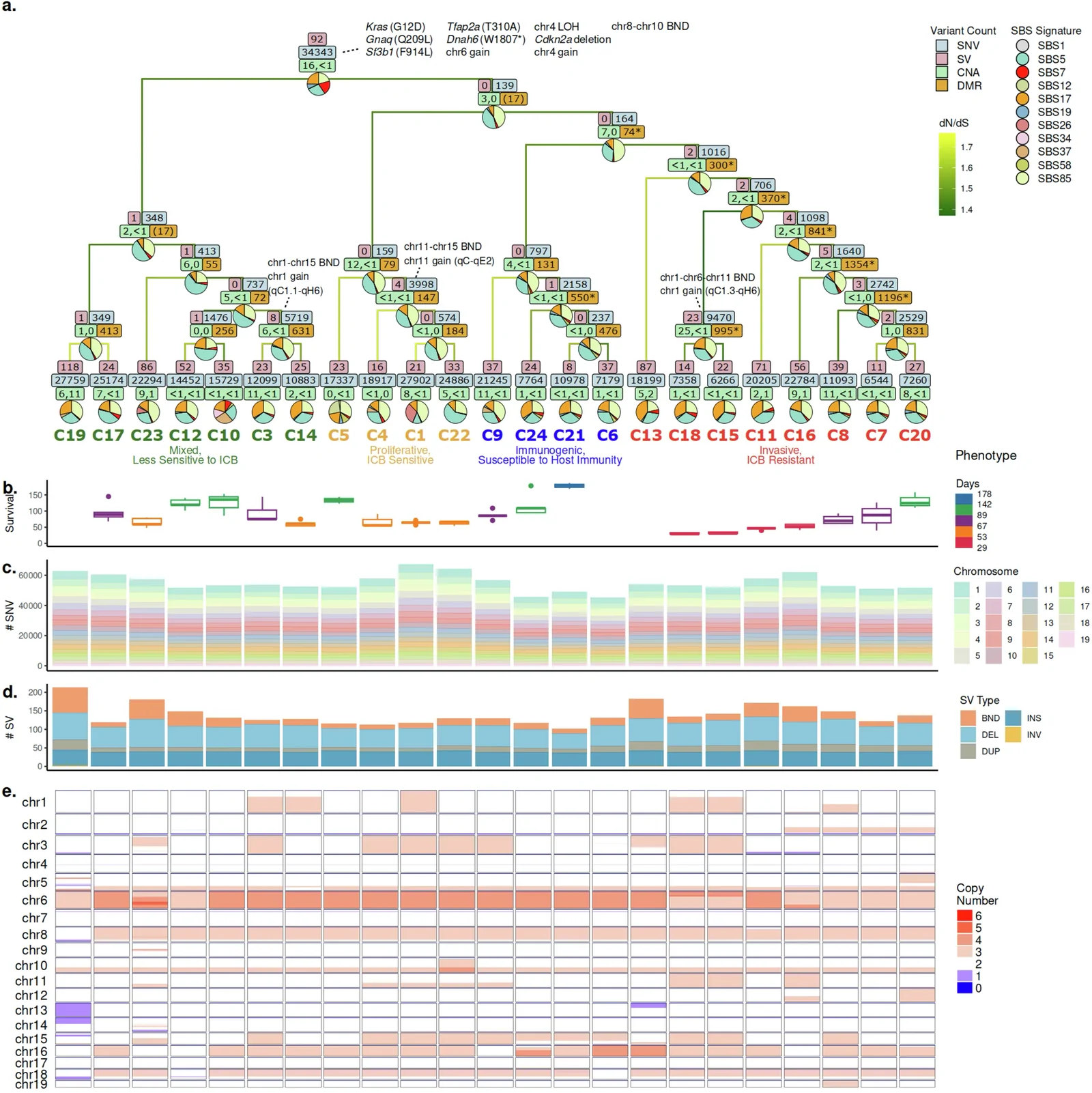
Phylogenetic tree and mutational landscape of 23 single-cell derived sublines. **a** Phylogenetic tree with each node labeled with (i) counts of single nucleotide variants (SNV), structural variants (SV), copy number aberration (CNA)-impacted percentage of the genome, and differentially methylated regions (DMRs) assigned to the incoming branch of the node, (ii) pie chart describing exposure proportion of mutational signatures active along the incoming branch of the node, and (iii) key variants and events of note placed along the incoming branch of the node, if any. SNV and SV counts reflect those originating at that node. CNA numbers represent percentages of the genome collectively amplified and lost among the sublines below the branch. DMR counts reflect the number of regions detected between the two groups of sublines defined by the bipartition of the tree at the node: the parenthesized numbers at the two immediate children of the root node reflect the same set of regions, and the numbers marked with an asterisk * denote those that are statistically significant per permutation tests (see Methods). The sublines are organized into 4 major clades, denoted by the distinct colors of the leaf labels. Sublines with previously determined phenotypes^24^ are also marked. Each branch in the phylogeny is colored by the corresponding dN/dS value. **b** Growth kinetic of tumors derived from individual sublines in C57BL/6 mice^20^. The survival time was defined as the duration from cell implantation to the tumor reaching 1000 mm^3^ in each mouse (*n *= 5). The box plot for each subline shows the minimum and maximum (two whiskers with outliers excluded), median (center), upper and lower quartiles (bounds of box), and outliers (filled circles). **c** Per subline total SNV count per autosome, colored by chromosome. **d** Per subline total SV count colored per SV type. **e** Per subline copy number profiles, colored by integer copy number, shown per chromosome. Source data is available at 10.5281/zenodo.20072601^128^.

To infer the evolutionary relationship between sublines, we constructed a phylogenetic tree with ScisTree^25,31^ using a subset of high-confidence SNVs (Methods). From the initial set of 277,818 phylogenetically informative SNVs (61.75% of all SNVs used; excludes SNVs present in one or all sublines), we removed SNVs with low coverage (7.09% of informative SNVs) and SNVs that overlapped with deletions in any subline (89.00% of informative SNVs) (Methods). This resulted in a final set of 10,862 high-confidence, informative SNVs. The constructed tree consisted of 4 clades that we will refer to as “major clades" - colored in green, orange, blue, and red - representing 4 distinct subclonal populations (clades) in the B2905 cell line (Fig. 2a).

In a parallel study, we characterized the contrasting phenotypes of selected sublines from each clade according to their in vivo growth rates, expression of cell cycle genes, and response rates to anti-CTLA4 ICB treatment^20,24^. To test the in vivo growth kinetics of each subline, cells were implanted into five C57BL/6 mice to measure the duration for the tumor growth to reach 1000 mm^3^, a pre-determined endpoint to define survival time (Fig. 2b). Sublines with zero or low tumor take rate in immunocompetent C57BL/6 mice could grow tumors in immunocompromised nude mice, suggesting that immunogenicity impacts in vivo growth of these sublines. The red clade had an aggressive phenotype with rapid in vivo growth and lower response to ICB, resembling an invasive melanoma subtype^32^; while sublines in the orange clade had slower in vivo growth and higher ICB response, resembling a proliferative subtype. Sublines in the green clade showed mixed phenotypes of those in the red and orange clades. Sublines in the blue clade had low tumor growth in immunocompetent C57BL/6 mice are referred to as immunogenic^33^. (Methods, Supplementary Table S3) Overall, the phylogenetic reconstruction reflected the observed phenotypic differences in sublines between clades and enabled the analysis of variant timing and lineage phenotype associations that we describe below.

### Phylogeny-guided harmonization with TreeHarmonizer provides evolutionary timing of genomic variants

While the tumor phylogeny described above accurately reflects the evolutionary relationship between the sublines, it excludes a large portion of SNVs that potentially overlap with deletions or have low coverage, and it does not include SVs or CNAs. This is typical for popular tumor phylogeny reconstruction methods that rely on SNVs^34^ (see more detailed discussion in Methods). Inclusion of different variant types is complicated by possible overlaps (e.g., SNVs within subclonal deletions) or erroneous variant calls. We thus developed a tool called TreeHarmonizer^35^ that (i) places variants on the branches of a phylogenetic tree and (ii) identifies variants in disagreement with the tree (we will refer to the corresponding variants as placed and unplaced, respectively). TreeHarmonizer assumes the input tree is optimal and does not directly evaluate the validity of the existing topology nor change it.

Briefly, TreeHarmonizer takes as input (i) a phylogenetic tree and (ii) individual SNV, SV, and CNA calls for every subline. For each unique variant, it evaluates whether the variant is consistent with the input phylogeny based on (i) the most-recent common ancestor (MRCA) of all sublines carrying the variant, (ii) the variant status in all sublines, and (iii) the presence of nearby deletions. A variant is denoted as “placed” if it passes all of TreeHarmonizer’s criteria for consistency with the tree and is assigned a branch that it is placed at. All variants that do not pass the criteria at any stage are denoted as “unplaced” (Methods). We denote a variant as clonal, subclonal, or private, if they were placed at the trunk, internal branch, or terminal branch of the tree, respectively (Fig. 2).

On our mouse melanoma dataset, TreeHarmonizer placed high proportions of all SNVs and SVs (96.71% and 95.49%, respectively). Of all placed SNVs, 7.89, 8.38 and 83.73% were clonal, subclonal and private, respectively. Within individual sublines, on average 62.23, 8.69 and 29.08% of SNVs were clonal, subclonal and private, respectively (Supplementary Table S4). Proportionally, SVs were distributed similarly to SNVs, where 7.60, 4.94, and 87.46% of all SVs were clonal, subclonal, and private, respectively. Within individual sublines, on average, 65.13, 5.58, and 29.29% of SVs were clonal, subclonal and private, respectively. (Supplementary Table S5). The variant allele frequency (VAF) of placed clonal and subclonal variants was distributed around 50%, as expected for heterozygous somatic variants fixed in a given lineage (Supplementary Fig. S1). Overall, the high proportion of variants placed confirms agreement between the phylogenetic tree and genomic landscape of the sublines. It also suggests that sublines were free of contamination, as contamination would result in a decrease of placed variants.

To further evaluate the correctness of variant placement, we examined the set of SNVs that were placed by TreeHarmonizer differently as compared to the initial phylogenetic assignment by ScisTree, which typically relies on more stringent filters for input variant calls in searching for the correct tree structure (Methods). We compared the partition function values^26^ which estimate the probability that, given a set of calls in individual sublines, a mutation is placed at the MRCA branch of a clade (Methods). Indeed, TreeHarmonizer variant placement led to a significant increase (one-tailed Wilcoxon signed-rank test *p* = 9.331*e* − 06) in their partition function values, confirming accurate variant placement (Supplementary Fig. S2).

### Evolutionary dynamics of somatic SNVs and mutational processes

Overall, the cumulative number of variants (all variants acquired along the course of evolution at a given tree node) was variable between sublines and major clades; in particular, sublines in the blue clade tend to have a below-median number of both SNVs and SVs (Fig. 2c, d and Supplementary Fig. S1). The number of SNVs and SVs within individual branches of the tree were strongly correlated (*r*(43) = . 798, *p* = 5.158*e* − 11 < . 001; Supplementary Fig. S1), suggesting that the rate of SNV acquisition relative to that of SV is roughly constant over time.

We next sought to explore the timing of mutational processes, commonly explained through the patterns of somatic SNVs. We estimated activities of COSMIC^36^ mutational signatures at each branch of the tumor phylogeny (Fig. 2a, Methods). Notably, SBS7 (a UV damage signature) was prevalent at the trunk (Fig. 2a and Supplementary Fig. S3), consistent with the fact that the tumor model was induced by UV radiation. Interestingly, the SBS7 signature explained only around 20% of truncal mutations, which may suggest that another strong clonal sweep occurred after cancer initiation.

Some signatures were present throughout the phylogeny, such as clock-like signatures (SBS1 and SBS5), and SBS85 (attributed to activation-induced cytidine deaminase; AID) (Fig. 2a and Supplementary Fig. S3). While SBS85 has been typically described in the context of lymphoid cancers^37^ and is unusual for melanoma, it may reflect a signature of immune response. It has also been shown that inflammation-induced AID expression in non-lymphoid cells promotes skin cancer development independently of UV damage^38^. Another possible explanation is that the SBS85 signature reflects a mouse-specific mutational process with a mutational signature similar to SBS85 in human tumors.

Other signatures were more specific with timing or lineages. For example, SBS26 (defective DNA mismatch repair), had increased activity solely at the terminal branch attached to subline C1, which had the highest cumulative number of SNVs among all sublines (Fig. 2a, c and Supplementary Fig. S3). SBS17, a signature potentially associated with damages inflicted by reactive oxygen species (ROS), shows a significant difference in activity both between internal and terminal branches (adjusted *p* = 9.08e-05; Methods), and between branches in the orange and red clade (adjusted *p* = 9.68e-03; Methods, Supplementary Fig. S3). While the differences between internal and terminal branches could be explained by the additional oxidative stress caused by cell culture^39^, the difference between the orange and red clades may reflect the difference in the metabolism between them, as we have shown previously that the expression of mitochondrial genes was selected in the former^24^. (Fig. 2a and Supplementary Fig. S3).

To identify putative initiating events and variants that contributed to lineage differentiation, we performed functional annotation and enrichment analysis of SNVs (Methods, Supplementary Fig. S4). Interestingly, dN/dS values at internal branches were significantly lower than terminal branches (*p* = 5.9e-07, Supplementary Fig. S4a, Methods), suggesting an increasing positive selection pressure over the evolutionary timeline (Fig. 2a, Methods). Among the clonal SNVs, we observe hotspot coding mutations, such as *Kras* (G12D) and *Gnaq* (Q209L) that are well-known cancer drivers, and variants in *Sf3b1* and *Tfap2a*, which are frequently mutated genes in melanoma^40,41^ (Fig. 2a). Both *Sf3b1* and *Tfap2a* mutations have been shown to drive melanomagenesis in mouse models^42,43^, though they occurred only in a relatively small subset of human cutaneous melanoma^22,44,45^. Other variants were either not in hotspot regions or rarely mutated in human cutaneous melanoma. Overall, impacted genes were enriched in pathways related to neural system development and differentiation, as well as differentiation of neural crest to melanocytes (Supplementary Fig. S4b). At the same time, individual clades exhibited enrichment in functionally distinct and phenotypically relevant pathways, such as DNA damage checkpoint signaling in the green clade (Supplementary Fig. S4), cell cycle and metabolism in the orange clade (Supplementary Fig. S4) and cell migration and methylation in the red clade (Supplementary Fig. S4). Overall, this analysis revealed the dynamics of mutational processes and putative functional effects of lineage-specific mutations, that we associate with other genomic, epigenomic or phenotypic changes below.

### Somatic SVs dynamics suggest early drivers and late genomic instability

**Fig. 3:**
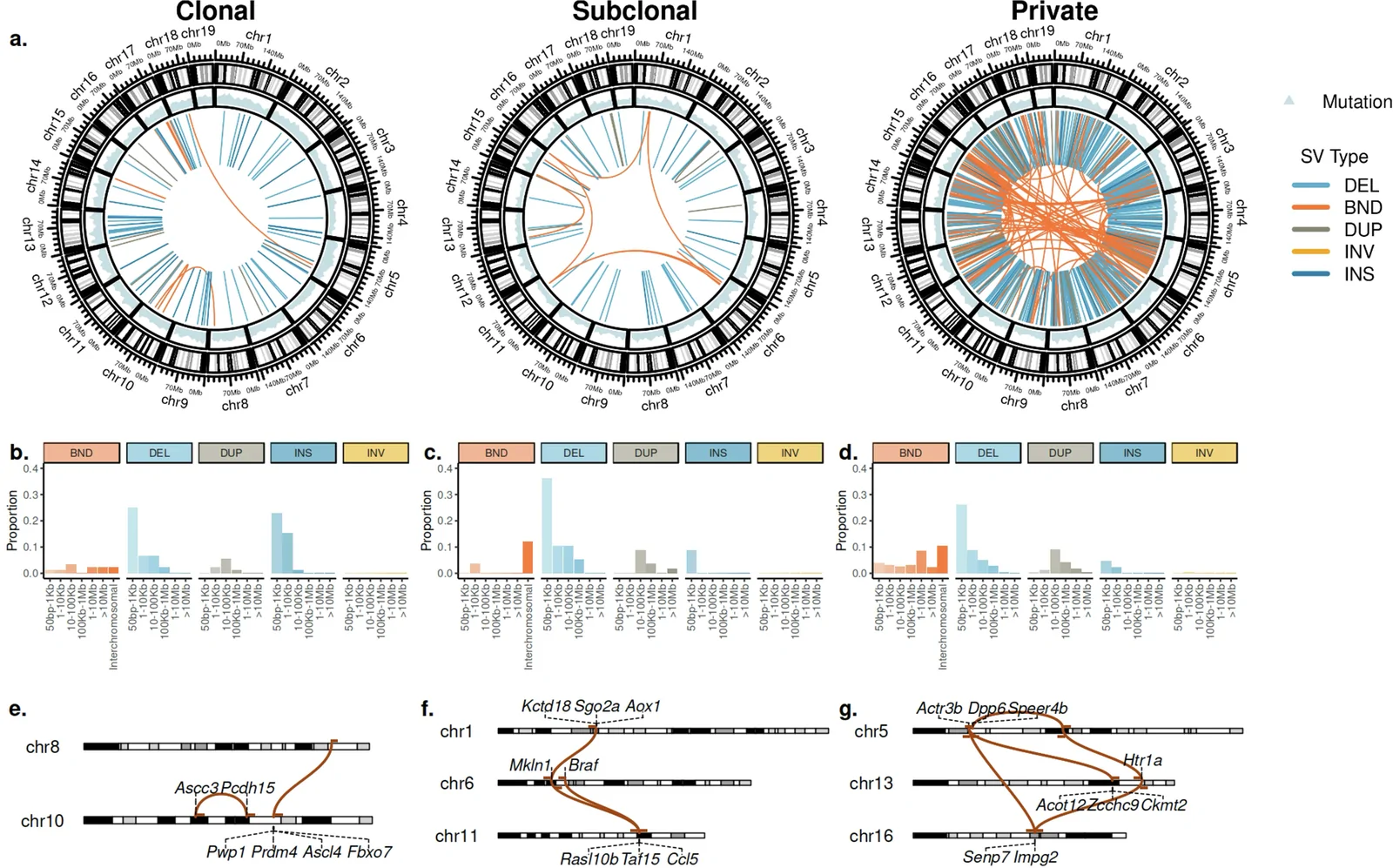
Patterns and dynamics of somatic SVs. **a** Chromosomal distribution of clonal, subclonal, and private single-nucleotide variant (SNV) and structural variant (SV) events. DEL: deletion; BND: breakend; DUP: duplication; INV: inversion; INS: insertion. **b**–**d** Distribution of clonal (**b**), subclonal (**c**), and private (**d**) SVs across categories defined by SV type and size. **e**–**g** Examples of notable clonal (**e**), complex subclonal (**f**, shared by sublines C15 and C18), and complex private (**g**, private to C10) interchromosomal SVs. Genes near breakpoints are annotated. Source data is available at 10.5281/zenodo.20072601^128^.

Long-read sequencing enables accurate detection of somatic SVs, which may be driven by orthogonal mutational processes and have a substantial impact on lineage phenotypes, as we illustrate below. Overall, small deletions (< 1 kb) were the most common SV; insertions were also common at the clonal level, but less common as private; and (interchromosomal) breakend junctions were more common at the private level (Fig. 3a–d). Severus further groups breakend junctions into clusters if they are likely to result from a single mutational event (Methods). We identified 65 such clusters involving a total of 298 SVs: of which four were composed of clonal SVs (e.g., Fig. 3e), one of subclonal SVs (e.g., Fig. 3f), and 60 of private SVs (e.g., Fig. 3g). Private SV clusters were the most complex: while clonal clusters consisted only of two SVs each, 14 private clusters had at least five SVs, and largest cluster had 28 SVs in C13 (Supplementary Figs. S5–S8). The green clade was overall enriched with clustered SVs, compared to the other clades (150/298 clustered SVs were in the green clade; Fisher’s exact test, *p* = 3.055e-07), primarily driven by C19 that had the highest number of clustered SVs (*n *= 68) in nine clusters (Supplementary Fig. S5). Overall, the vast majority of clustered SVs were private, suggesting that in this tumor complex rearrangements were late events, potentially driven by an increased genomic instability.

Using Padfoot (https://github.com/KolmogorovLab/Padfoot), we identified 564 SVs with breakpoints occurring within gene boundaries (Methods). Of these, 330 affected coding regions through exon deletions or disruptions involving exonic sequences. Notably, we observed clonal deletions affecting exon 4–9 of *Nav3* (Supplementary Fig. S9), which included the first coiled-coil domain, the mutations in which have been shown to result in a loss of function^46^. In accordance, deletion of the *NAV3* gene has been linked to poor prognosis in cancer^47^. In addition, in sublines C3 and C14, we identified a subclonal deletion in *Met* resulting in the loss of exon 4–11 (Supplementary Fig. S9), which included extracellular and regulatory domains. Such truncated *MET* has been shown to be self-activated and promoting immortalization of normal cells^48^. We also detected 24 candidate gene fusions, of which 23 were private. The only subclonal fusion between *Map4* and *Smarcc1* was identified in C3 and C14 and validated using scRNA-seq (Supplementary Fig. S9).

Beyond disrupting genes and generating oncogenic fusions, SVs also cause CNAs that alter gene expression, which will be the focus of the following subsection.

### Recurrent CNAs in independent lineages suggest parallel adaptation process

**Fig. 4:**
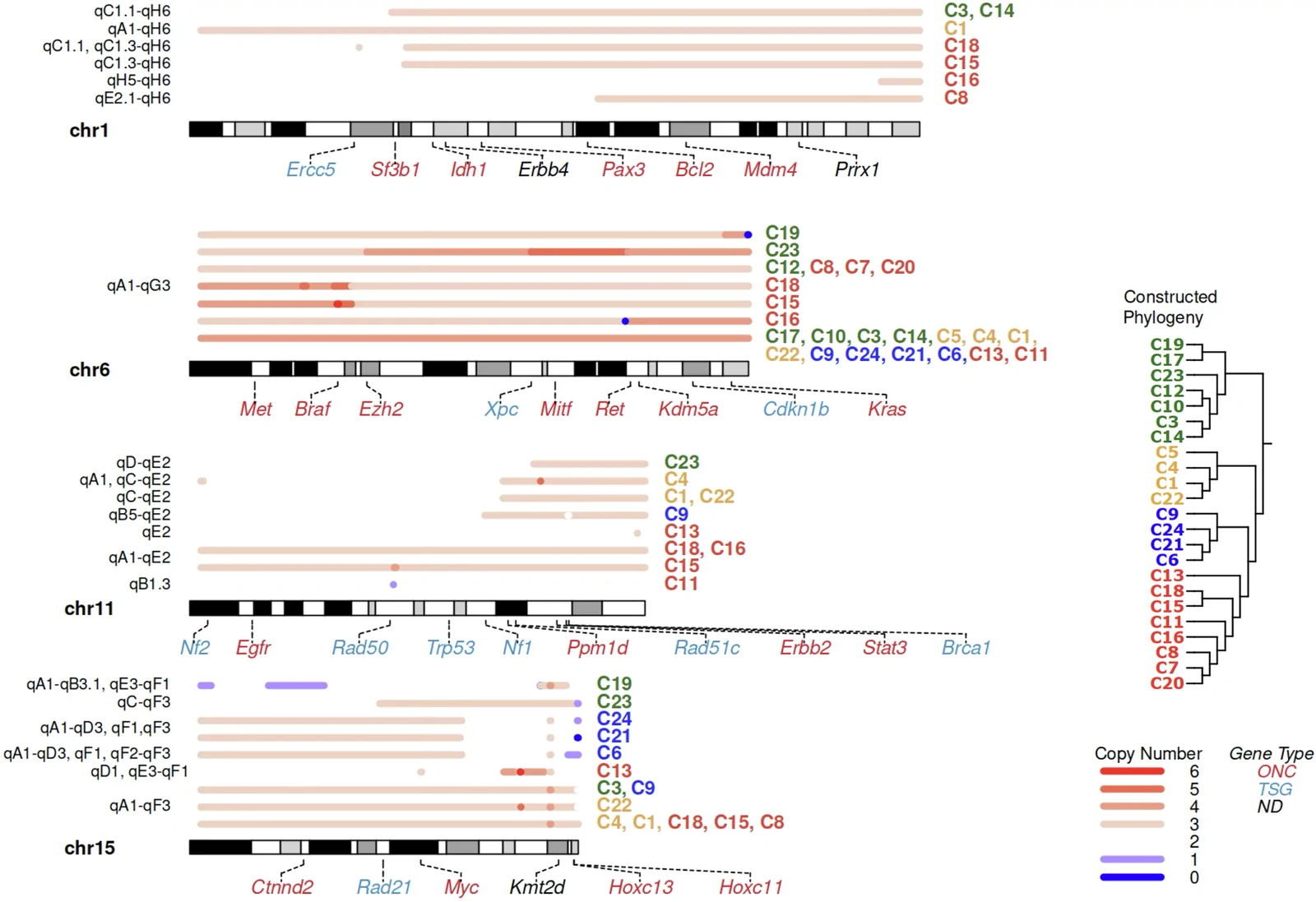
Genomic regions recurrently affected by CNAs suggest parallel evolution. Examples of regions on chr1, chr6, chr11, and chr15, recurrently harboring copy number aberrations (CNAs), possibly due to independent genomic events that impact the same region. Within each chromosome, sublines harboring CNAs are grouped by the similarity of their copy number profiles, and the respective copy number profiles are annotated by the chromosome bands they span. Copy number profile spans are colored by their respective integer copy number. Select genes are annotated along the chromosomes and colored by whether their human homologs are designated as oncogene (ONC) or tumor suppressor (TSG) according to COSMIC Gene Census^36^. Source data is available at 10.5281/zenodo.20072601^128^.

SVs often cause CNAs, potentially affecting large contiguous regions of the genome. Overall, a high proportion of the genome was impacted by amplifications (26.37% on average per subline), and a smaller fraction impacted by losses (1.24%) (Fig. 2a, e). To understand functional implications of these alterations, we focused on genes with one-to-one human homologs curated in the COSMIC Gene Census^36^ and labeled them as oncogene (ONC), tumor suppressor gene (TSG), or not designated (ND) (Methods, Fig. 4 and Supplementary Figs. S10–S16).

We first focused on clonal CNAs, such as the whole-chromosome amplification of chr6 in all sublines, although some sublines acquired additional copies in later events (Fig. 4). These CNAs affected key ONCs, such as *Met*, *Braf*, and clonally mutated *Kras* (Fig. 4 and Supplementary Fig. S12). Other putative targets included *Mitf*, a lineage-survival oncogene^49^ via copy number gains and overexpression in 10% of primary and 15–20% of metastatic cutaneous cases^50^. We also observed a focal loss of *Cdkn2a* in both copies of chr4 with the same breakpoint boundaries. This was likely a result of a focal deletion, followed by a copy-neutral loss of heterozygosity (LOH) in a larger region, which happened approximately half way through the phylogeny trunk based on variant timing analysis (Methods). This overall suggests that a substantial number of early CNA events could have contributed to tumor initiation.

Next, we analyzed the patterns of subclonal and private CNAs. While TreeHarmonizer does not directly place CNAs, we noted through profiling the copy number changes with analyses such as in Fig. 2e, and through TreeHarmonizer placement of SVs, that similar regions of the genome were amplified by different SV breakpoints in independent parts of the tree (Methods). This suggests that these amplifications were independent recurrent events in different lineages, representing examples of parallel evolution. In one case, five distinct genomic rearrangement events resulted in independent amplifications of a large portion of chr1 in seven sublines (Fig. 4): two SVs were subclonal, three were private; most of them were products of intra-chromosomal fusions, with one exception of whole-chromosome amplification in C1. In another example of a parallel CNA, a chr11 gain in C4, C1, and C22 is associated with the same subclonal interchromosomal SV involving chr15, the one in C9 involves chr18 and results in the amplification of TSG *Nf1*, which also carries a private, non-synonymous SNV in C9 (Fig. 4 and Supplementary Fig. S14). This analysis suggests that subclonal CNAs substantially contributed to the differentiation of tumor clades, and indicates convergent evolution in independent lineages.

Indeed, among COSMIC genes impacted by CNAs, ONCs were preferentially gained (*p* = 3.832e-02, Methods) and TSGs were preferentially lost (*p* = 7.294e-03, Methods) in this tumor, as expected ; suggesting that those changes may be independently selected for in a highly heterogeneous tumor^51^. For example, parallel amplification of *Myc* was recurrently present in a majority of sublines (Fig. 4 and Supplementary Fig. S15); likewise, the *MYC* proto-oncogene is commonly amplified in human cutaneous melanoma^52^. In C13, *Myc* is duplicated via a focal SV spanning merely 1.6 Kbp, likely reflecting targeted selection. Furthermore, a closer examination of ONC/TSG COSMIC gene homolog pairs adjacent to copy number change boundaries shows that, more often than expected by chance, ONC falls on the side of the boundary with the higher copy number state while TSG on the lower side (*p *= 3.74e-02, Methods). This provides evidence that relative positioning of ONC and TSG genes on chromosome may influence the selection of CNA boundaries.

### Methylation changes contribute to differentiation of an aggressive clonal lineage

In addition to genomic variants, epigenomic changes may shape the observed distinct lineage phenotypes. Long-read sequencing, combined with a phylogenetic approach, enabled us to identify genomic regions that are differentially methylated at subclonal resolution (Fig. 2a).

**Fig. 5:**
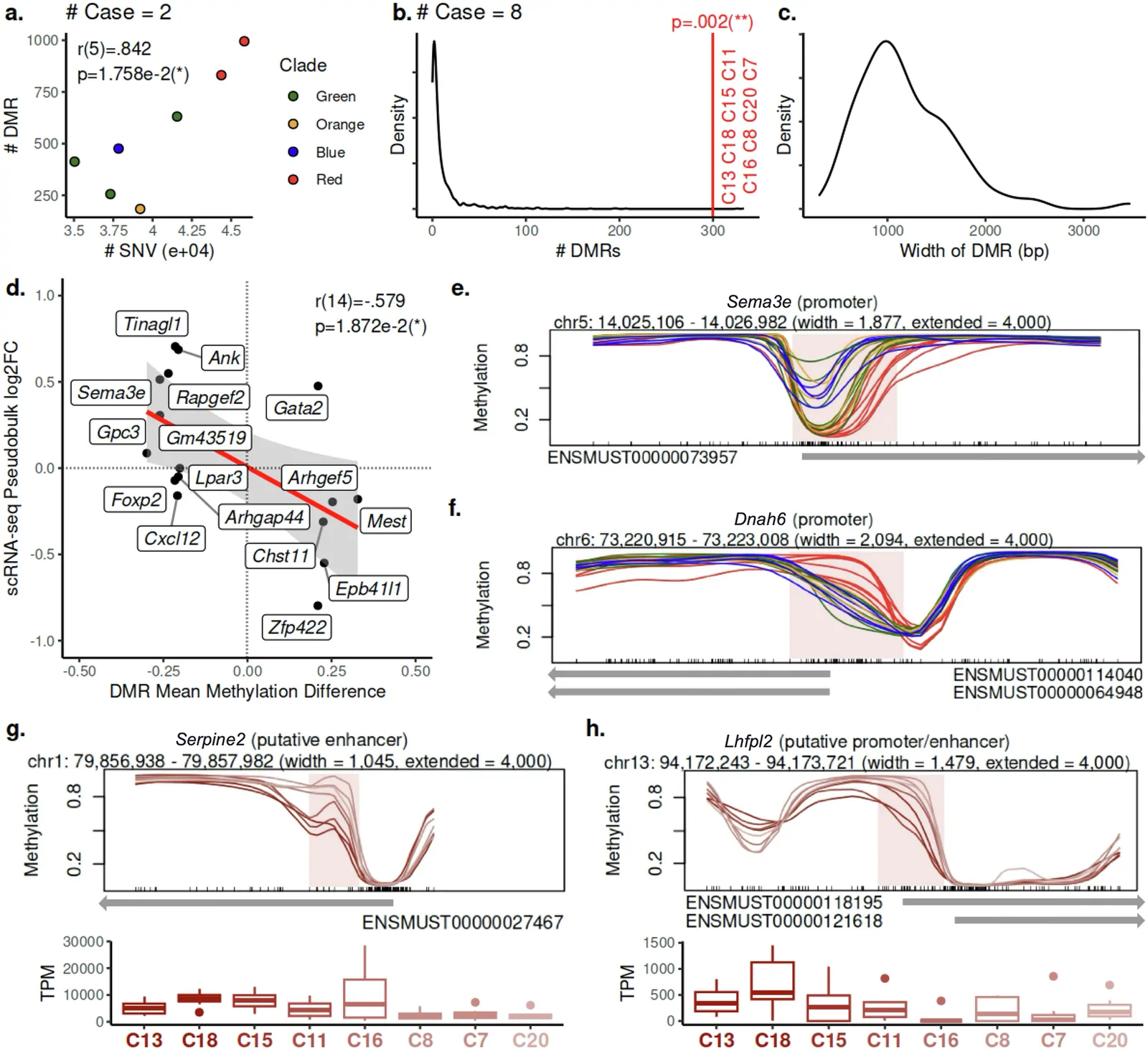
DMRs detected at subclonal resolution. **a** The number of single nucleotide variants (SNVs) cumulative from trunk to the branch and the number of differentially methylated regions (DMRs) detected at the branch, for each branch parental to 2 leaves. Assessed with a two-sided pearson correlation test. **b** The number of DMRs detected at the branch rooting the red clade and the corresponding null distribution of the number of DMRs generated during the permutation test for case groups of size 8. Assessed with a one-sided permutation test (Methods). **c** The distribution of the widths of the 300 DMRs detected at the branch rooting the red clade. **d** Mean methylation difference within DMRs between sublines in the red clade and others, and the corresponding log2 fold change in gene expression in genes whose (putative) promoter or enhancer the DMRs overlap, as measured by scRNA-seq. Assessed with a two-sided pearson correlation test. **e** DMR overlapping the promoter of *Sema3e,* where sublines in the red clade are hypomethylated. **f** DMR overlapping the promoter of Dnah6, where sublines in the red clade are hypermethylated. **g** Genomic region overlapping a putative enhancer of *Serpine2,* where it is monotonically becoming hypermethylated in the red clade, while the gene expression of *Serpine2* follows a monotonicly decreasing trend. **h** Genomic region overlapping a putative promoter of *Lhfpl2,* where it is monotonically becoming hypermethylated in the red clade, while the gene expression of *Lhfpl2* follows a monotonicly decreasing trend. In (**e**, **f**), the methylation level in each subline is plotted with the color corresponding to the clade of the subline in the tumor phylogeny. In (**g**, **h**), the methylation level and transcripts per million (TPM) values as profiled by scRNA-seq in each subline are plotted with the color hue corresponding to its phylogenetic order in the red clade. The box plot for each subline shows minimum and maximum (two whiskers with outliers excluded), median (center), upper and lower quartiles (bounds of box), outliers (filled circles) of the TPM expression values across its biological replicates. Correlation tests were not adjusted for multiple comparisons. Source data is available at 10.5281/zenodo.20072601^128^.

For each internal branch in the phylogeny, a set of DMRs between the bipartitions of the sublines induced by the corresponding tree split was identified using BSmooth^30,53^. We then evaluated significance of the number of DMRs obtained via a permutation test for branches ancestral to a particular number of leaves (Methods, Supplementary Fig. S17). We found a strong positive correlation between the number of cumulative SNVs and the number of DMRs at a branch (*r*(5) = . 842, *p* = 1.758e-2, Methods; Fig. 5a), suggesting that the rate of differential methylation to some extent mirrors that of SNVs in the evolution of this tumor. Among the genes whose (putative) promoter or enhancer overlap DMRs, the orthologs of many are also similarly impacted by differential methylation in at least one of 17 human cancer types according to OncoDB^54^ (Supplementary Figs. S22–S24).

We then focused on epigenomic analysis of the red clade since it was characterized with significantly worse survival compared to other clades (Kaplan-Meier, *p* < . 0005; Fig. 2a). For the root branch of the red clade, BSmooth^30^ obtained 300 DMRs, which is significantly more than what is expected for any group of eight sublines (*p* = . 002, Methods, Fig. 5b). The median length of the DMR regions was 1081bp (Fig. 5c). Among all 300 DMRs, 17 overlap with the putative promoter regions in GENCODE transcription start sites^55,56^, and nine were annotated in the EPDnew (v.003) database of experimentally validated promoters ^57,58^. Another nine DMRs displayed enhancer-like signatures and were within 2 Kbp of an annotated transcription start site^59–62^ (Supplementary Table S6). In a validation analysis, we found the mean methylation differences between case and control groups in such regions are negatively correlated with the measured expression differences of the corresponding genes (Fig. 5d).

Notably, the EPDnew-validated promoter of *Sema3e* was found to be hypomethylated (Fig. 5e), and its human homolog has been shown to drive cancer cell invasiveness^63^. In addition, the EPDnew-validated promoter of *Dnah6*, previously implicated in ICB response in human melanoma^64^, was found to be hypermethylated in the red clade (Fig. 5f). Coincidentally, *Dnah6* also harbors a clonal truncating mutation p.W1807* (Fig. 2a). Upon closer examination, we found evidence for haplotype-specific methylation of the *Dnah6* promoter on the mutated copy in the green, orange and blue clades, and the hypermethylation in the red clade was likely associated with the loss of the wild-type copy (Supplementary Fig. S18).

While the red clade was distinct from the rest of the phylogeny in terms of survival, it also showed gradual changes of genotype and phenotype within the clade. Specifically, we considered the ordering of the 8 sublines in the red clade with respect to their distances to the root, referred to as branching order (Fig. 2a). These sublines followed a linear differentiation pattern, instead of a more balanced branching pattern in other clades. Further, survival monotonically improved along the path from the root of the phylogeny towards leaves C7/C20. *τ*(5) = 1, *p* < . 001 (Methods, Fig. 2a). To evaluate if methylation was driving gradual change in phenotype, we analyzed DMRs within the red clade with respect to the branching order (the relative ordering of subline pairs C7 and C20, as well as the pair C15 and C18, along with leaf C11 which are equidistant to the root, was determined based on survival time) and focused on (putative) promoter or enhancers of known genes.

We found 146 genes whose (putative) promoter or enhancer overlap with regions displaying monotonic change in methylation rate along the resulting subline ordering, which is found to be significantly more than what is expected by chance via a permutation test on the order of the sublines within the red clade (*p* = . 021, Methods). Some of these methylation changes were correlated with changes in scRNA-seq expression. In particular, a putative enhancer of *Serpine2* and the putative promoter and enhancer of *Lhfpl2* are gradually hypermethylated in the red clade, and their expression correspondingly decreases (Fig. 5g, h). In human melanoma, SerpinE2 expression has previously been associated with high invasive potential of slow-cycling cells^65^, and *LHFPL2* expression was associated with mTORC1 signaling^66^, which promotes mesenchymal-epithelial transition in cancer^67^.

### Systematic analysis of multiple variation types provides insights into B2905 lineages differentiation

**Fig. 6:**
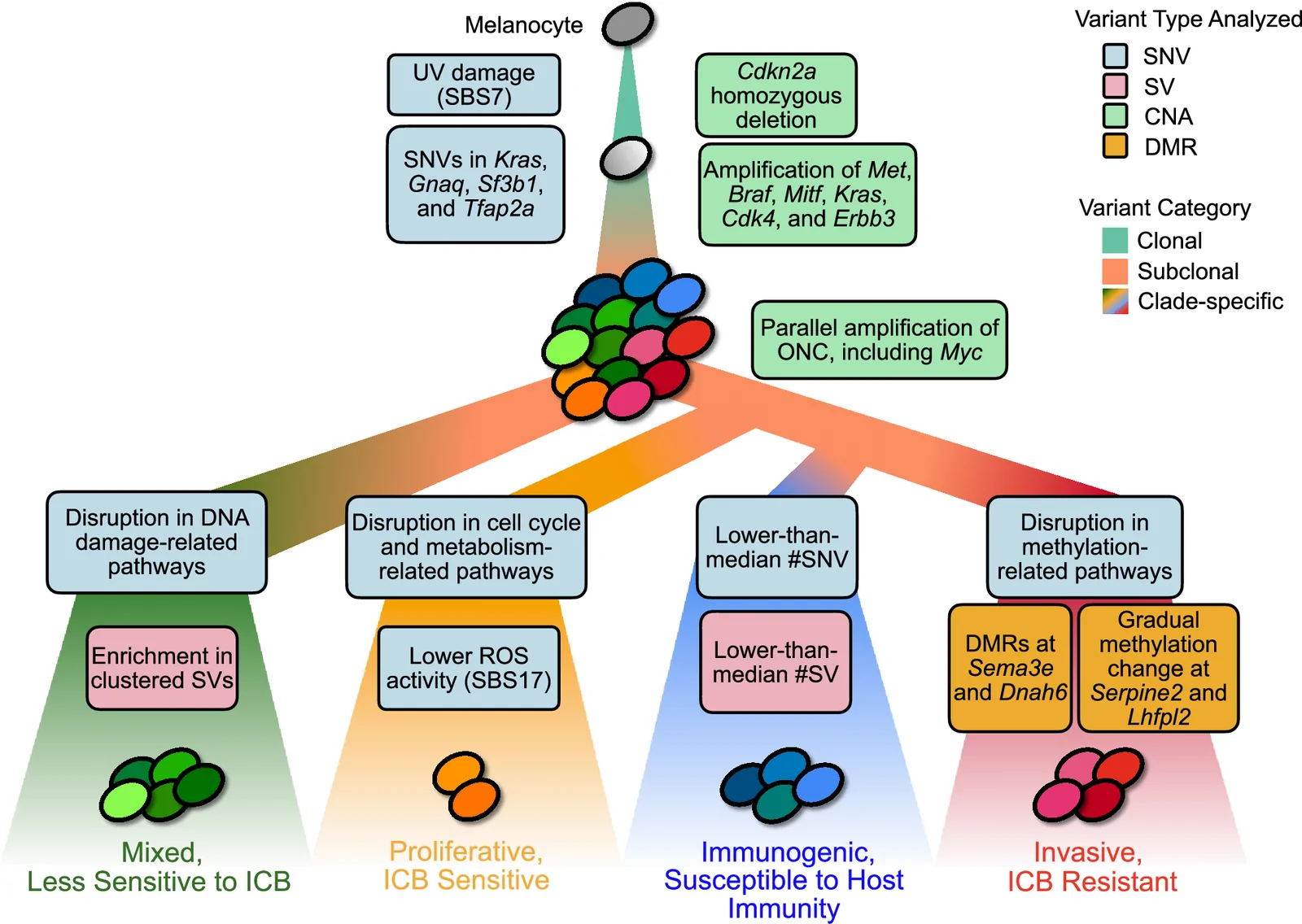
A model of B2905 melanoma evolution informed by analyses of long-read sequencing data on the 23 sublines. Key clonal, subclonal, and clade-specific observations from analyses of the single-nucleotide variants (SNVs), structural variants (SVs), copy number aberrations (CNAs), and differentially methylated regions (DMRs) are labeled along the tumor evolutionary trajectories. Box shadings represent the associated variant type analyzed. Evolutionary trajectories are shaded based on their representation of clonal, subclonal, or clade-specific variants.

Overall, our long-read approach offers us a unique lens to observe the complete spectrum of genomic and epigenomic events at the subclonal level and provided insights into the evolution of the B2905 melanoma (Fig. 6). We detected clonal mutations of all types that affected genes known to contribute to melanoma initiation, including hotspot SNVs in *Kras* and *Gnaq*, SNVs in frequently mutated sites of *Sf3b1* and *Tfap2a*, homozygous deletion of *Cdkn2a*, and amplifications of *Met*, *Braf*, *Mitf*, *Kras*, *Cdk4*, and *Erbb3*. Importantly, CDKN2A deletion^68^, amplification of human chromosome 7 (containing MET and BRAF)^69^, and copy number gain of MITF^50^ frequently happened during human melanoma progression. We observed a substantial proportion of UV damage signature on the clonal level, but not on sublonal levels, confirming UV damage as a key initiation event^70^, but unlikely the driver of lineage differentiation.

On the subclonal level, SNVs occurred in genes enriched in pathways associated with the typical phenotypes of the clades. For example, cell proliferation-related pathways in the green and orange clades, more neural development-related pathways in the blue clade, and more cell migration-related pathways in the red clade (Supplementary Fig. S7). These enrichment patterns implied that mutations may happen in the actively expressed genes^71^. Accurate and precise SV breakpoint detection, enabled by long-read sequencing, allowed us to resolve parallel CNAs events that affected overlapping genomic regions. For example, *Myc* was independently amplified across different lineages via distinct genomic rearrangements, ranging from whole-chromosome duplications and a small, focal amplification. Overall, the positioning of CNA breakpoints relative to neighboring ONC and TSG genes suggested that these events occurred randomly, and then were independently selected.

Furthermore, our phylogenetic approach enabled the comparisons among different major clades that resulted in observed phenotypic heterogeneity. In the green clade, which had a significantly higher number of clustered SVs, we observed a disproportional disruption of pathways related to DNA damage signaling and response (Supplementary Fig. S4), possibly causing an increase in genomic instability. In the orange clade, the disrupted pathways related to cell cycle and metabolism and lower ROS activity cohered with its proliferative phenotype (Supplementary Figs. S4, S3). Blue clade sublines had significantly fewer clade specific SNVs (one-tailed *t* test, *p* = 3.31*e* − 02) and SVs (one-tailed *t* test, *p* = 1.65*e* − 02); which may reflect lower selection pressure on the blue sublines, but requires further investigation.

The red clade – showing faster growth in immunocompetent hosts and higher ICB resistance – stood out in terms of increased number of characteristic DMRs, including promoters of *Sema3e* and *Dnah6* that could drive the aggressive phenotype. Interestingly, methylation-related pathways in the red clade were disproportionally impacted by non-synonymous SNVs, which may explain the increase in observed methylation changes, compared to other clades (Supplementary Fig. S4). Moreover, we observed that a significant number of genes in the red clade, such as *Serpine2* and *Lhfpl2*, displayed a monotonic change in methylation at (putative) promoters or enhancers, often correlated with aggressiveness and scRNA-seq expression changes. This suggests that DMRs may drive a gradual phenotypic change. Overall, this showcases how joint analysis of SNVs, SVs, CNAs and DMRs provides an explanation of lineage-specific phenotypes and genotypes.

### Curated set of subclonal variant calls enables benchmarking of long-read tools

**Fig. 7:**
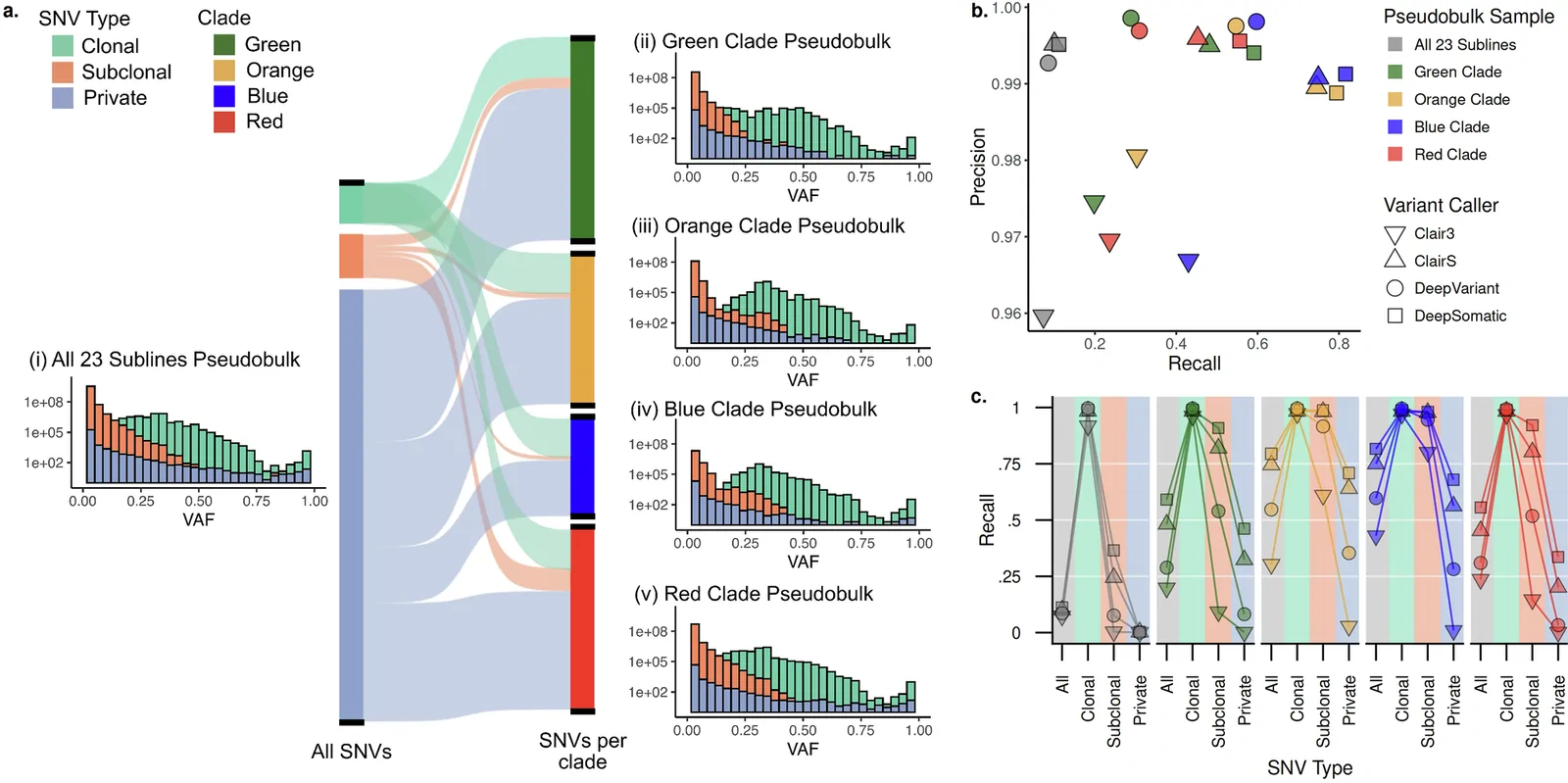
Subline-derived pseudobulk samples enables benchmarking the performances of long-read variant callers at various clonality. **a** Five pseudobulk samples generated by merging reads from (i) all 23 sublines, and sublines in (ii) green clade, (iii) orange clade, (iv) blue clade, and (v) red clade. For each pseudobulk sample, the makeup in terms of clonal, subclonal, and private variants is shown via the Sankey diagram, and the VAF distribution of the corresponding ground truth set of variants is shown, respectively. **b** Precision and recall performance of Clair3, ClairS, DeepVariant, and DeepSomatic on the five pseudobulk samples. **c** Recall performance of variant callers on the five pseudobulk samples, stratified by the clonality of the target variants in the ground truth set. Source data is available at 10.5281/zenodo.20072601^128^.

In addition to enabling a highly detailed view of melanoma evolution, our approach provides a unique resource for development of cancer genomics methods. Variant placement using TreeHarmonizer resulted in a highly accurate set of SNV, SV and CNA calls with an established phylogenetic relationship. This enables modeling a heterogeneous tumor sample by mixing subsets of sublines sequencing data, as for each such subset a benchmarking set of variants could be easily derived. As a proof of concept, we generated 5 pseudobulk samples by merging reads from different clade combinations (Fig. 7a), and then compared several recently developed state-of-the-art tools for long-read variant calling in terms of their precision and recall on somatic variants (Fig. 7b, Methods).

While precision was generally high for all callers, recall varied highly between callers and across different pseudobulk samples (Fig. 7b). Specifically, as compared to clonal SNVs, recall for subclonal and especially private SNVs was substantially lower, likely due to the decreased allelic fraction of such variants (Fig. 7a, c). Overall, we found that recent tools that were developed for somatic analysis (ClairS and DeepSomatic) performed better than general-purpose variant callers, with DeepSomatic performing the best, maintaining the high precision as other callers, while notably outperforming in recall (Fig. 7b).

This analysis underscores the difficulty of detecting rare variants from heterogeneous cancer samples – one of the key challenges in precision oncology. Even though somatic variant callers demonstrated an improvement over germline callers, even the best performers left many variants undetected in the most complex pseudobulk dataset. We publicly release the data and variant calls to encourage the development and continuous improvement of cancer genomics methods.

## Discussion

In this study, we present an approach to comprehensively map genomic and epigenomic alterations that drive the initiation, progression and clonal differentiation of a mouse melanoma tumor model. We used Nanopore sequencing of clonal sublines – each derived from a single cell of the parental tumor – to systematically catalog somatic SNVs, structural variants (SVs), copy number alterations (CNAs), and differentially methylated regions (DMRs). We developed an algorithmic framework to place all variant types in the context of the sublines phylogeny, representing dynamics of various mutational processes along the evolutionary timescale. This approach provided a highly detailed and comprehensive view of melanoma evolution, illuminating the complex interplay between mutational processes that drive phenotypic differentiation of clonal lineages. We also demonstrated that our data can serve as a useful resource for cancer genomics developments.

Long-read sequencing of individual sublines enabled precise resolution of parallel evolution events^72^, which has been challenging to resolve using conventional approaches. Prior studies have reported examples of parallel CNAs and SVs using bulk sequencing of multiple matching samples, for example, in ovarian^73^, breast^74^, and osteosarcoma^51^ cancers. However, these analyses were typically constrained by heterogeneity of individual samples and limited spatial or temporal resolution, making it difficult to confidently identify independent events affecting the same genomic regions. More recent efforts using single-cell DNA sequencing reported large parallel CNAs in cell line models^75^ and in acute myeloid leukemia with complex karyotype^76^. Yet, due to data sparsity, these approaches often rely on pseudo-bulk inference of structural breakpoints, which prevents assignment of CNAs and SVs to individual cells. In contrast, our approach provides a highly resolved view of parallel clonal evolution thanks to the high purity of sublines, a higher number of samples and the advantages of long-read sequencing.

In addition to genetic variation, Nanopore sequencing enabled the detection of lineage-specific differentially methylated regions (DMRs), offering insights into the epigenetic landscape of tumor evolution. DNA methylation has increasingly been recognized as both a marker of clonal lineage^77–80^ and a regulatory mechanism driving phenotypic diversity^81–84^. Our work focuses on the latter, extending epigenetic resolution to the subclonal level, beyond traditional comparisons of tumor versus normal tissue or across tumor subtypes.

Recent long-read studies have explored allele-specific methylation (ASM), for example, in brain tissue^27^ or medulloblastoma^85^. While genome-wide ASM analysis was difficult due to the low heterozygosity of the B2905 mouse model, we illustrated that local analysis was possible in the presence of nearby somatic SNVs; furthermore, the analytical framework presented here could be readily adapted to genome-wide allele-specific analyses, for example, in humans.

Overall, our study highlights the advantage of long-read sequencing in resolving the complete variational landscape of tumors, potentially benefiting precision oncology studies, as for various cancers it has been shown that variants other than SNVs, including SVs and DMRs, are important biomarkers for prognosis or therapeutic response^86,87^. Since these results were based on a specific mouse model of RAS-mutated cutaneous melanoma, carrying driver mutations Kras^G12D^ and Gnaq^Q209L^ that occur infrequently in human counterparts, it requires caution to analyze the representativeness of the identified mechanisms. However, our analytical framework is model agnostic and can have broader applications, and the results have important biological implications.

Tracing the variants at different genomic, epigenetic, and transcriptional levels and integration of these data can expand the scope of evolutionary modeling of cancer beyond the classic viewpoint focusing on coding regions and/or small variants in a snapshot manner, leading to testable hypotheses to gain mechanistic insights, which in turn may help develop therapeutic strategies. Likewise, the TreeHarmonizer method, as well as phylogenetic and variant calling methods described here, are not limited to mouse genome analysis, and could be applied to single-cell derived human tumor datasets. We note that analysis of multi-site bulk samples, each consisting of multiple cancer clones, would require a different approach to resolve clonal deconvolution and heterogeneity.

Finally, by publicly releasing all sequencing data and curated variant call sets, we provide a unique benchmarking resource to accelerate the development and validation of cancer genomics tools.

## Methods

### Statistics & reproducibility

For in vivo growth kinetics studies, each subline was implanted subcutaneously into groups of five syngeneic C57BL/6 mice. Since no therapeutic effect was evaluated, the sample size was chosen based on standard practice in the field for syngeneic mouse tumor models; no statistical method was used to predetermine sample size. Tumor size measurements for in vivo therapeutic studies were performed independently by an animal technician, providing a degree of observer independence for the primary endpoint measurement. Tumor volume was calculated as V = 0.5 × L × W^2^, where L and W represent the longer and shorter diameters, respectively. Endpoint was defined as tumor volume reaching 1000 mm^3^ (for in vivo growth kinetics studies), tumor ulceration, or moribund status. The survival time, which was defined as the duration from cell implantation to the endpoint, of individual mice were presented; no Kaplan-Meier analysis was performed. No data were excluded from the analyses. The experiments were not randomized. The Investigators were not blinded to allocation during experiments and outcome assessment.

All cell lines were authenticated by whole exome sequencing, HGF transgene genotyping, and spectral karyotyping, and confirmed to be Mycoplasma-negative using a MycoAlert Detection Kit. Computational analyses, including SNV calling, phylogenetic inference using Trisicell, followed previously described and validated pipelines.

### Cell lines

The cell lines analyzed in this study were generated and reported by Gruen et al.^20^ as following. B2905 (M4) mouse cell line13 and the clonal sublines were cultured in RPMI supplemented with 10% of FBS and 1% L-Glutamine. To generate single-cell derived sublines, exponentially growing M4 cells were sorted in a 96-well plate by fluorescence-activated cell sorting (FACS) using a FACSAriaIII cytometer. Single cells were successfully cultured and expanded to create early-passage sublines. Authentication of all cell lines was performed by HGF transgene genotyping^21^, whole exome sequencing, Spectral karyotyping (SKY), and long-read sequencing described in this study. Cell lines were confirmed as Mycoplasma negative using a MycoAlert Mycoplasma Detection Kit (Lonza, LT07-418).

### Animal studies

The animal studies that generated data used in this study were reported by Gruen et al.^20^ as following. The mice used in the preclinical studies were 6–12 weeks old female C57BL/6NCr mice, supplied by Charles River facility in NCI-Frederick, with no selection on the weight. They were housed in AAALAC-accredited cage as a group of five, with ad libitum food and water, and a 12 h light cycle. All mouse experiments were performed in accordance with Animal Study Protocols approved by the ACUC, NCI, NIH. For testing the in vivo growth kinetics of each subline, 10^6^ melanoma cells from each subline were implanted subcutaneously (s.c.) into five C57BL/6 mice, and tumor growth was monitored until endpoint. For all studies, tumor size and body weight were measured twice per week and the endpoint was established as the occurrence of one of the following events: (1) tumor size reached 1000 mm^3^; (2) tumor became ulcerated; and/or (3) the mouse showed moribund status or sickness behavior.

### Nanopore sequencing of B2905 sublines

Here we sequenced 23 mouse melanoma cell lines derived in a previous study^20^ and 1 mouse spleen using Oxford Nanopore. gDNA was extracted using a high molecular weight DNA extraction kit for cells and blood (New England Lab cat# T3050), following the manufacturer’s instructions. The extracted gDNA was further processed for nanopore sequencing following the protocol described previously^88^. Briefly, purified HMW gDNA from the 22 samples were hand-shear 20X with 1 mL Luer-Lock syringe (Becton Dickinson cat#309628) and a 0.8 mm x 40 mm needle (Becton Dickinson cat#305167). Molecular DNA size was determined using TapeStation 4200 (Agilent cat#G2991BA); Most of the samples were over 55 Kb. After quantification using the Qubit dsDNA BR assay kit (Invitrogen cat#Q32853), at least 5 μg of DNA for each sample was size selected with the PacBio short read eliminator kit (PacBio cat# 102-208-300) to reduce fragments of DNA under 25 kb. After eluting the samples with 150 μl of EB buffer, DNA was quantified again by Qubit BR assay. Samples with similar concentrations (typically at 30–60 ng/μl range) were sheared simultaneously to a target size of 30 kb with the Megaruptor 3 instrument (Diagenode cat#B06010003) and the DNAFluid + Kit for viscous DNA (Diagenode cat#E07020001) for 2 rounds at speed 45. Once the shearing was completed, samples volumes were reduced from 150 μl to 50 μl, using the speed vacuum instrument Vacufuge plus (Eppendorf cat#E-VPVCCS). The molecular DNA size was examined again using TapeStation to confirm that the samples were high molecular weight DNA. For ONT sequencing library construction, 48 μl of each sample with at least 2 μg of DNA were prepared using the Nanopore Ligation Sequencing Kit V14 (Nanopore cat#SQK-LSK114) with a few modifications: (i) During the DNA repair and end-prep step, 75% ethanol was used for washing instead of the recommended 70%. (ii) SFB buffer was used in the adapter ligation and clean-up step. (iii) The library elution conditions were changed to 20 min at 37 ^*o*^C. Finally, the libraries were loaded into flow cells R10.4.1 (Nanopore cat#FLO-PRO114M) for sequencing with PromethION devices. To obtain high depth coverage, each library was reloaded several times into the same flow cell, after performing a nuclease flush using the Nanopore flow cell wash kit (Nanopore cat#EXP-WSH004).

Sublines C1, C8, C11, C13, C9, C17 and the splenocyte normal were sequenced at 4 khz, while the remaining sublines and the melanocyte were sequenced at 5 khz, due to the frequency and requirement of ONT MinKNOW sequencing software updates. Basecalling was performed with either guppy(v6.4.2)^89^ for the 4khz samples or dorado(v0.3.4)^90^ for the 5khz samples. Quality of the sequencing was verified by the sequencing teams and confirmed using samtools stats^91^, seqkit stats^92^ and pycoQC^93^. No significant batch effects were observed between basecaller versions and sequencing frequency differences. Full sequencing statistics compiled from seqkit and pycoQC per subline are available in Supplementary Table S1.

We note that we did not sequence C2 subline with Nanopore, as it has showed evidence to contamination with C3.

### Data preparation and somatic SNV calling

Long-reads were aligned using minimap2^94^ to the GRCm38 (mm10) reference. Aligned BAM files were phased and haplotagged using margin^95^ and Whatshap^96^ with the splenocyte normal. SNVs were called independently per subline using DeepVariant(v1.6.1)^28^ with the default parameters for ONT R10.4 chemistry. Somatic SNVs were determined by subtracting both the splenocyte normal SNVs and the SNVs present in the FVB mouse line. We note that DeepSomatic^97^ – a specialized long-read somatic SNV caller – became available after the majority of this analysis was completed. However, because of very few germline mutations (mm10 reference was derived from the same C57BL/6 strain) and low heterogeneity within each subline, we expect DeepVariant to perform similarly to DeepSomatic on this dataset, when calling per individual subline.

SNVs for FVB were called using dipcall^98^, comparing the reference assembly for C57BL/6 to the latest long read-based FVB strain assembly^99^. FVB alignments covered most, but not all, of the C57BL/6 reference assembly, indicating either highly divergent regions or assembly errors. Since within such regions we could not distinguish somatic variants in the sublines from germline FVB variants, we removed the regions from further analysis. Excluding those regions removed approximately 11.62% of the genome (autosome only), which in turn excluded around 14.93% of all called somatic SNVs.

Our initial analysis showed very high rates of likely-erroneous calls from chrY; In humans chrY is very difficult to assemble due to high repeat complexity^100^, and this may influence read mapping in mouse chrY. Secondly, since the M4 mouse was an F1 cross between a C57BL/6 female and FVB male, it inherited FVB chrY, which seems to be substantially distinct from the grcm38 (which is based on the C57BL/6 strain). Because of the many observed mapping artifacts, we excluded chrY from the analysis and decided to focus on autosomes (excluding chrX as well). In a separate analysis of CNAs, SNVs, SVs, and DMRs on chrX, we verified that its evolutionary history is consistent with the rest of the chromosomes (Supplementary Fig. S16).

We also note that although variant calling tools used in this study support germline haplotype phasing to improve variant calling quality, our mouse strain is highly inbred and thus germline variant phasing was not possible.

### Extending Severus for multi-sample calling

SVs were called across all sublines in a single run using the multi-sample mode of Severus^9^, which eliminates the need for ad-hoc re-genotyping. Unphased BAM files from each subline and the normal sample were provided as input. Severus first produces the calls from a combined alignment of all input samples. Then, it genotypes the presence or absence of each SV in each sample individually. This allows us to discover variants supported by only a few reads if a variant is confidently called in other samples.

Severus then groups SVs into clusters to detect complex SVs. If a complex SV is shared across multiple samples, then all junctions within that complex event must be present in exactly the same set of samples.

To account for batch effects arising from differences in N50 and read quality across samples, the –low-quality parameter was enabled. Germline SVs from the FVB strain were filtered via a panel of normals (–PON) option. FVB SV calls were generated from the FVB assembly using hapdiff^27^. In addition, confident regions from the FVB assembly were used to define reliable genomic intervals and further reduce potential false positives.

### Copy number variant calling

We used Wakhan^29^ in unphased mode to call copy number aberrations in each subline separately. As an input, Wakhan requires BAM files to extract bin-level coverage and tumor phased VCFs to detect LOH. We provided Wakhan with haplotagged BAM files and the somatic variant callset VCFs per subline. We ran Wakhan with a bin size of 50kb to extract coverage from the BAM file histograms. Wakhan was provided Severus generated structural variations/breakpoints VCF files so that it could detect more precise copy number change boundaries. We used an additional option, –cpd-internal-segments, to enable a change point detection algorithm that can detect small copy number changes in the rare cases when Severus misses a breakpoint. Average ploidy for sublines was 2.25 (Supplementary Table S7).

Some genomic areas were challenging due to the poor mapping quality, reference artifacts, ends of chromosomes, and very small spans between breakpoints. However, the vast majority of such challenging regions were short. As Wakhan is optimized for calls larger than  ~ 50 Kbp, one should take care when using calls below this threshold. We only consider calls larger than 50 kb for any functional analysis in this work, unless they fall between a pair of Severus breakpoints representing one SV. We also use smaller calls for conservative region filtering during the initial tree construction, as discussed in the section below.

### Constructing tumor phylogeny using long read sequencing data from 23 sublines

To infer binary cell-linage tree of 23 sublines using ScisTree^31^, we obtained a subset of highly reliable SNVs. For that, we first filtered out (i) SNVs in regions that were impacted by copy number losses in at least 1 subline, as such mutations are subject to the violations of the infinite-sites assumption and (ii) private (present in only one subline) or clonal (present in all sublines) SNVs, as these are not informative for phylogenetic analysis. We note that although some recently developed methods allow mutational losses^101–103^, our filtered SNV set was large enough for reliable tree construction using traditional methods. After that, for each mutation in each subline, we set the mutation status to “missing” if the coverage at the site is ≤ 10, deferring to the tree-building algorithm to determine the presence-absence status of the mutation in the subline due to insufficient coverage.

Although long-read sequencing can map the vast majority of the genome (Supplementary Fig. S2), highly repetitive regions (such as centromeres or segmental duplication) remain challenging and may remain unmapped in multiple sublines. To avoid sites with consistently low coverage, as their mutation calls are unreliable across many sublines, we also filtered out sites with mean or median coverage across all sublines  < 20. In addition, we also removed mutations with mean or median VAF across all sublines in which they were called as present below 1/3, a value lower than the expected VAF for most non-private heterozygous SNVs in our dataset (which is typically 0.50 for mutations from diploid regions). Lastly, we observed that some SNVs had some, but low, read support for the alternate allele in a significant fraction of sublines for which they were called as absent. Such SNVs are typically unreliable and require the tree- building algorithm to make many false positive/negative corrections. We suspect that such ambiguous SNV calls are the result of various sequencing or mapping artifacts. To reduce the impact of such calls, we ignored sites for which there was read support for the alternate allele in at least half of the sublines in which the SNV was called absent. After the above filtering steps, we were left with 10,862 high-quality, phylogenetically informative somatic SNVs that were used for tree inference. We note that our filtering model does not explicitly consider genomic context (e.g., repetitive regions), however SNV and SV variant callers use genomic context for quality control.

To construct the phylogenetic cell lineage tree from the filtered set of SNVs, we used ScisTree^31^, a neighbor-joining based method that performs search in the space of cell-lineage trees. It is suitable for our dataset, which has a significantly lower number of cells compared to the number of SNVs. We performed a grid search over a range of false positive (10^−2^, 5 ⋅ 10^−2^, 10^−3^, 10^−4^, 10^−5^, 10^−6^, 10^−7^, 10^−8^) and a range of false negative (. 05, . 075, . 1, . 125, . 15, . 175, . 2) priors. In addition, as ScisTree performs a heuristic search, its output can depend on the order of the rows (cells) in the input. Therefore, we also repeated the tree-building process for 100 different permutations of the input rows. As the final output, we selected the tree with the maximum likelihood among all the 8 (false positive priors)  ×  7 (false negative priors)  ×  100 (permutations of rows)  = 5600 trees inferred by ScisTree.

We note that the final tree is highly consistent with the trees reconstructed from WES data in companion studies^20,24^, with a few minor differences. First, the different placement of C19 is explained by contamination in the samples used for WES. Second, the relative placement of the blue clade and the orange clade (except C5) was different, likely because of insufficient mutational signal in the WES data. Since many more mutations were called using ONT sequencing, the tree described here is likely to be more accurate compared to the WES-based trees.

### Determining the phenotypes of the 4 major clades

Here, we provide a brief summary of the previous work that characterized the phenotypes of the B2905 sublines^20^. After generation from parental B2905 melanoma cell line, the work performed the following characterization studies: (1) each subline was subjected to exome sequencing and scRNA analysis; (2) each subline was tested for in vivo growth in C57BL/6 mice; (3) selected sublines were tested for response to ICB in C57BL/6 mice.

As defined by the criteria by Hirsch et al.^24^, the invasive phenotype exhibits faster tumor growth when implanted in an immunocompetent host, lower expression of cell cycle genes, and expression of non-canonical Wnt signaling genes; meanwhile, the proliferative phenotype exhibits slower tumor growth when implanted in an immunocompetent host, higher expression of cell cycle genes, and expression of canonical Wnt signaling signature. Given the relevant results from the characterization study^20^, accordingly, the orange clade was determined to be proliferative, the red clade invasive, and the green clade exhibiting intermediate levels of these features was defined as having a mixed phenotype (Supplementary Table S3).

The response to host immunity (immunogenicity) of each subline was estimated by the delay of growth initiation (DGI, the time from implantation of cancer cells to starting continuous growth without regression) in immunocompetent C57BL/6 mice implanted with each subline^20^. The longer period of DGI indicates a stronger response to host immunity. DGI diminished when cancer cells were implanted to immunocompromised host, as compared to that in an immunocompetent host, confirming the association of DGI with tumor response to host immunity (Supplementary Fig. S19).

To test the response to ICB, one or two sublines were selected from the green, orange, and red clade^20^. The blue clade was not tested because of the low tumor take rate (the odds that implanted cancer cells can grow a tumor in the hosts). For each selected subline, its cells were implanted into ten C57BL/6, which were randomized to receive anti-CTLA4 or isotype control antibody treatment. Taken together, these results of the characterization studies label each major clade with a distinct phenotype (Supplementary Table S3).

### TreeHarmonizer algorithm

Given a phylogenetic tree, TreeHarmonizer attempts to place variants on their appropriate position within the evolutionary context. Tree branches could be of the following classes: truncal (occurring jointly prior to clone differentiation in this context), internal (an informative variant for clone differentiation), or terminal (a privately obtained mutation for this specific clone). Variant placement is challenging because variants of different types may overlap (e.g., SNVs inside subclonal deletion) or be miscalled in a subset of sublines. TreeHarmonizer takes several input parameters. By default, it takes a phylogenetic tree; SNV, SV and CNA call sets; and an estimated variant call FN rate. For our data, the estimated FN rate is 15%, and the estimated FP rate is 0.001%, as determined by the phylogeny creation in the previous section. The SNVs call set are DeepVariant-called somatic SNVs per subline. The SV call set are Severus-called somatic SVs per subline. From the SV call set, TreeHarmonizer uses SVs labeled as deletions by the caller. The CNA call set is CNA calls from the tool, Wakhan. From the CNA call set, the TreeHarmonizer uses regions labeled as copy numbers 0 or 1. Variants of each type are assumed to be acquired independently.

For each distinct SNV, defined by chromosome, position, reference allele, and alternate allele, TreeHarmonizer begins by calculating its most recent common ancestor (MRCA) given the sublines in which it was called. Given the low a priori FP rate (0.001%) for our input, we assume that the existing variant set used to compute the MRCA is correct, and do not consider possible permutations of variant status for sublines. Future versions of TreeHarmonizer will allow for an FP rate parameter for better data generalizability. Given our estimated FN rate input parameter, and phylogenetic tree that is expected to consist of multiple major taxa, we define a clade-size based threshold. For clades of size 1 or 2, a variant must have the support of all sublines. For clades of size 3 and higher, the minimal subline support is the largest integer such that (*clade*_*size* − *support*)/*clade*_*size* > = *FN*, so that we approach as close as possible (but do not go below) the target FN rate. We allowed this to apply to clades of size 3 (even though this leads to an estimated FN rate of 33%), as the sublines within each of those clades had low branch support and high variability between the nearly-optimal phylogeny candidates.

Subline genomes may contain large deletions and structural variations that result in large losses across the genome. Such losses can lead to SNVs in those regions being deleted, which leads to an apparent contradiction with the phylogenetic tree structure under the infinite sites assumption. TreeHarmonizer models the loss of such variants, given deletion and information and assigns the “lost variant" genotype to the respective sublines. We refer to this procedure as regenotyping. In this dataset, we used SVs identified as deletions by Severus, and CNAs labeled as copy numbers 1 or 0 as regions of loss (since the majority of the chromosomes had 2 copies). Although different SV processes can lead to different loss types, all losses are treated in the same manner for regenotyping. For each SNV position, TreeHarmonizer creates a new candidate set of sublines consisting of the original calls, plus sublines where a loss may have deleted an SNV. We then evaluate the candidate set with several parameters. We consider whether the MRCA changes, and if the clade placement threshold is met with the new set of sublines. For this data, we allow for the regenotyping of all variants that pass their clade threshold for the new subline set, with two main following restrictions in order to be conservative. To account for the lack of phasing information in many spans of the genome, we employ a parsimony assumption – for an SNV position that is being regenotyped, if there exists an SNV called within the loss or deletion span being considered for the same subline, we assume that the loss occurred on the other haplotype, and we do not regenotype this SNV position. Furthermore, we do not allow for dramatic changes in variant placement (a shift from a clade low on the tree to one very high on the tree), here defined as a subline reinclusion rate of  > 100%, as those are significantly more unlikely. If the regenotyped candidate set is rejected, the original called set is used instead. Ultimately, for this dataset, we regenotyped using only Severus called deletions (SVs), as they are more focal and conservative than larger spanning CNAs. Among the placed SNVs, 1753 (0.39%) variants are regenotyped using detected deletion regions that would otherwise be considered erroneous and discarded. Full placement percentages are available in Supplementary Tables S4, S5.

A small proportion of SNVs and SVs remained unplaced after running TreeHarmonizer. A variant is considered unplaced if it did not pass the minimum threshold of sublines required by its clade size and FN rate formula. Just under 3.29% (*n *= 14,791) of SNVs remained unplaced. Around 24.24% (*n *= 3585) of unplaced SNVs fell just (up to 3 sublines) below the threshold limits for clonal placement, around 29.10% (*n *= 4304) of unplaced SNVs were called in 2 or 3 sublines across varying clades, potentially indicative of other unconsidered errors or artifacts in variant calling. SVs were placed onto the tree simply using the same clade placement thresholding formula as for SNVs. No regenotyping was performed on SVs. Since the number of unplaced SVs was relatively small compared to SNVs, we reviewed the unplaced variants and classified them into different categories. The vast majority of the 64 unplaced SVs were either in low-mappability regions characterized by increased mismatch rates (*n *= 21), clustered indels (*n *= 17) and secondary read alignments (*n *= 17) or mosaic repeat expansion (*n *= 6).

CNAs, as opposed to SNVs and SVs, were not placed, but rather TreeHarmonizer produces a percentage summary of amplifications and losses at each branch in the tree. As we are not currently associating all CNAs with specific SVs, the placement of CNAs onto the phylogeny is not as evolutionarily clear. Multiple independent events may impact the same span, and thus makes it difficult to determine unique, independent, CNA spans and define them as singular events. In order to produce this summary, we limited the definition of a gain or loss to a change from a baseline of CN = 2. Ranges of such gains and losses were extracted per subline from the Wakhan output. The current assignment method follows the assumption that - if a particular CNA span is represented by multiple sublines within a single subtree, that span has been amplified or lost earlier in time, and thus being subclonal or clonal in nature.

We begin by traversing the tree starting from the leaves, and find all such overlaps between multiple sublines in a subtree. More specifically, beginning at the leaves of the tree, we employ the following in a reverse level-order traversal — given a range *R*_1_ that corresponds to a set of sublines *S*_*r*1_, if there exists a *R*_2_ (with corresponding set *S*_*r*2_) such that *R*_1_ and *R*_2_ are super or subsets of one another, they are merged into a new range *O*_1,2_, along with their associated subline sets, *S*_1,2_ = *S*_*r*1_ ∪ *S*_*r*2_. Any ranges *R*_1_ and *R*_2_ which only partly overlap are split into three ranges: *R*_*rem*1_, *O*_1,2_, and *R*_*rem*2_. Range *O*_1,2_ is the overlap of *R*_1_ and *R*_2_, and is associated with each range’s corresponding subline sets. Ranges *R*_*rem*1_ and *R*_*rem*2_ are the remainder of each original range minus the overlap region. As a result, every internal (and truncal) branch now contain all overlapping ranges of their children, and exactly which sublines said overlaps are associated with.

Similarly to how we handle SNVs and SVs, we allow for a  ~ 15% false negative rate to model possibly missed CNA calls. Due to not requiring perfect clade completion in order to be considered valid, this may result in multiple “passing” candidate spans at the same subtree (with slightly different sets of sublines) that have partly or entirely overlapping ranges. Thus, per branch, any ranges *pR*_1_ and *pR*_2_ that overlap with each other are merged into a new range *O*_*pr*1,*pr*2_, along with their associated subline sets, *S*_*pr*1,*pr*2_ = *S*_*pr*1_ ∪ *S*_*pr*2_.

We now finalize the assignment of ranges per branch with a level-order traversal. As we traverse down the tree, as ranges are assigned, we exclude any span that has already been associated with a parent branch (as it is assumed to have been affected earlier). Ranges are treated as mutable objects — if a part of said range currently being considered was already assigned to a parent, the range is split, and the unassigned remainder is then assigned at the current branch.

TreeHarmonizer does not currently consider the potential overlaps with SVs after the simplification of CNAs into gains and losses. As it stands, the direct association of SVs with CNAs is still an open problem. We are currently working on developing a model that can handle such overlapping scenarios and hope to incorporate it into TreeHarmonizer in future studies. As such, the individual ranges described in the CNA summarization methodology are not currently user accessible.

We note that our approach to identify regions of loss from CNA and SV calls has limitations in the challenging scenario of overlapping gains and losses. We used Severus deletion calls and Wakhan CNA profiles to filter variants in the lost regions. For the scenario of overlapping gain and loss events that result in a diploid state for a genomic region, if such an event had discoverable breakpoints, then it would have been seen in the form of a deletion called by Severus and would have been considered in variant placement. If there are overlapping events with no detectable breakpoints, then it would be much more difficult to call, and we do not currently handle such events. For example, this may occur in regions with copy-neutral losses of heterozygosity if both loss and gain involve the whole chromosomes. However, we note that such overlapping gains/losses with no informative breakpoints are statistically less likely, compared to the typically observed events, and we expect that they should not substantially affect the resulting tree structure. Such complex gains/losses and rearrangements are difficult to detect for all existing methods that work with phylogenies using CNAs. We are currently working towards developing a model that can handle such overlapping CNA scenarios and hope to incorporate it into TreeHarmonizer in future studies.

### Assessing the effectiveness of TreeHarmonizer in determining the placement of variants impacted by filtering during tree construction

The partition function algorithm^26^ uses a tree sampling strategy to estimate the probability that, given a set of mutation calls, a mutation should be placed at the most recent common ancestor of a clade. To measure whether TreeHarmonizer effectively re-determines the placement of variants impacted by stringent filtering criteria during tree construction, we compute for those variants the respective partition function values (i) at their placement determined by ScisTree^31^, and (ii) at their placement by TreeHarmonizer, with the trees sample from the space defined by the original set of variant calls prior to filtering.

We focused on the set of variants whose placement differed following the application of TreeHarmonizer. By definition, if two variants are called in an identical set of sublines in the unfiltered data, their partition function values at a given placement in the tree will be the same if the values are estimated on the same set of sampled trees. Therefore, instead of computing partition function values per variant, we compute per group of variants that (i) were called in an identical set of sublines in the unfiltered data, (ii) shared the same placement by ScisTree, and (iii) shared the same altered placement by TreeHarmonizer. We computed the partition function values with 1000 sampled trees, 0.001 false positive rate and 0.15 false negative rate, and the results on the 27 groups are reported in (Supplementary Fig. S2). A one-tailed paired-sample *t* test was applied to the partition function values of the 27 groups.

### Mutational signature analysis along the tumor phylogeny

The cumulative placed SNV profiles of the 23 sublines were used as input samples to SigProfileAssignment (v0.1.9)^104^, a mutational signature fitting tool chosen for its superior overall performance and specifically for the number of mutations per sample at hand^105^. The 23 SNV profiles were translated into mutational spectra over 96 categories for joint refitting, and COSMICv3.4^36^ mm10 signatures were used as the reference catalog, as it has been shown that excluding sets of signatures from refitting often leads to poorer performance^105^.

SigProfilerAssignment outputted signature activity estimates per subline, as well as signature probability per individual mutation per subline. Each mutation placed at a particular branch was assigned the most probable signature given the aggregated signature probabilities for that mutation in sublines in the subtree below the branch. The activity of a mutational signature at a branch was then estimated by the proportion of mutations placed at the branch assigned to the signature. For concision of presentation, activities for signatures SBS7a, SBS7b, SBS7c, and SBS7d were aggregated and referred to as signature “SBS7”, and those for signatures SBS17a and SBS17b were aggregated and referred to as signature “SBS17”.

For each active signature, to assess whether signature activities have significant differences according to their evolutionary timing or between clades, each branch in the tumor phylogeny is labeled by (i) truncal, internal, terminal, and (ii) the color of the clade it is in (the trunk and two branches closest to the trunk have NA as clade assignment), and analysis of variance (ANOVA) with post-hoc Tukey HSD (Honestly Significant Difference) test was performed to see if there were significant differences between pairs along either independent variable. Since there is only 1 truncal branch, and the 3 branches closest to the root are only grouped together due to a lack of clade labels, numerically significant results (adjusted *p*-value  < .05) involving those as a group in the pair are not reported. In other words, only significant results between internal and terminal branches, or between pairs of clades, are reported (Supplementary Fig. S3).

We note that the current signature decomposition results point to some potential limitations of the current signature decomposition methods. For example, it is surprising to see the presence of SBS7 in the internal and terminal branches of the tree, as UV exposure is expected to be an early event in the evolution of this model. Because we ran SigProfilerAssignment on each subline independently and since the assignment of signatures is a statistical procedure, given that the algorithm detected the presence to SBS7 in each sample, it may overassign individual variants to this signature because of similarities in mutational profiles.

We also have considered two alternative approaches: (i) treating each branch as a separate sample by considering the mutations assigned only to that branch, and identify the signatures as well as signature exposure on that branch, or (ii) creating a pseudobulk of all the sublines for identifying signatures and estimate the exposure for each branch based on the signatures of the mutations assigned to that branch. We found the former tend to result in overfitting - evident by the notably higher number of mutational signatures noted as active - and the latter seems to sacrifice subclonal resolution. We chose to avoid these problems by taking the more balanced approach. While ours preserves major signals, it may sacrifice sensitivity to signature exposure shifts at smaller parts of the phylogeny, resulting in misassignment of common base changes. New methods are likely needed to properly capture the local dependencies of signature exposure in the tree, although it is out of the scope of the current work.

### Computing dN/dS values along the tumor phylogeny

Placed SNVs were annotated using Ensembl Variant Effect Predictor (v102)^106^. For any given branch, we computed the dN/dS value as the ratio of cumulative non-synonymous SNVs placed along the path from the trunk and including the current branch to that of cumulative synonymous SNVs, normalized by the ratio of synonymous sites to non-synonymous sites^24^. dN/dS values at internal branches were found to be significantly less than those at terminal branches via a one-tailed *t* test (Supplementary Fig. S4, *p* < *α* = . 05).

### Pathway enrichment analysis

Given a set of genes of interest - either that they are impacted by a certain type of variant at a particular time during the tumor evolution (e.g., placed at the trunk of the phylogeny) or in a particular clade - we performed Gene Ontology over-representation analysis to examine their potential functional impact^107,108^. Genes without a canonical name ("*Gm-*" and “*-Rik*" genes) were excluded from analysis. Using clusterProfiler (v3.21)^109^, we tested whether the candidate gene set significantly over-represents a Gene Ontology Biological Process gene set between sizes of 3 and 500. The resulting *p*-values were adjusted using the Benjamini-Hochberg procedure, and terms with *q*-value  < . 05 were determined to be statistically significant.

### Determining genes impacted by SVs

We used Padfoot (https://github.com/KolmogorovLab/Padfoot, commit ID:2d5237b) to annotate SV calls generated by Severus using the –specie mouse option and GRCm38.p4 GFF3 files with basic gene annotations. The *possible_fusion* and *exon_altering* events were manually validated using IGV and scRNA-seq alignments.

### Determining the set of cancer-relevant mouse genes-of-interest according to the COSMIC Gene Census

To obtain a set of mouse genes that likely have a functional impact in cancer, we selected those with one-to-one homology as genes curated in the COSMIC Cancer Gene Census (CGC, v101)^36^. Genes whose COSMIC homologs are uniquely labeled as ONC in CGC are labeled as ONC, and those whose COSMIC homologs are uniquely labeled as TSG are labeled as TSG. Those whose COSMIC homologs have no unique ONC/TSG labelings in CGC are labeled as ND (not designated). Among those without unique COSMIC ONC/TSG labelings, 4 genes (*Ezh2*: ONC^110^; *Rad21*: TSG^111^; *Tert*: ONC^112^; *Trp53*: TSG^113^) were given unique labels according to literature on melanoma or other cancer types. A gene is said to be impacted by CNA in a subline if it is entirely contained in a copy number gain or loss segment.

To determine if the gain or loss of a gene may be associated with its designation as ONC or TSG, we performed two *χ*^2^ tests on the set of COSMIC ONC/TSG genes impacted by CNA in any subline. One *χ*^2^ test evaluated enrichment of ONC that were gained in some sublines but not lost in any sublines. The other performed the corresponding test of TSG lost in some subline but not gained in any subline. The result was considered significant if *p* < *α* = 0. 05.

To see whether selection may be at play, we further examined the distribution of ONC/TSG around CNA boundaries. To that end, we specifically focused on COSMIC homolog pairs that are adjacent to each other along the genomic coordinate, where one is labeled ONC and the other TSG and have different absolute copy numbers in any subline, as they correspond to the nearest ONC/TSG COSMIC homologs upstream/downstream to a copy number change boundary. We counted in a total 14 instances where the ONC homolog is on the side of the boundary with a higher copy number state, and the TSG homolog is on the other. To assess the statistical significance of the observation, a null distribution was generated by counting instances of such observation in 5000 permutations of the ONC/TSG/ND labels of the COSMIC homologs. The *p*-value is the fraction of times the permutated labelings yield a greater or equal number of such observations than the actual labeling. We reject the null hypothesis if *p* < *α* = 0. 05.

### Timing analysis of chr4 copy-neutral LOH

The copy number calls from Wakhan suggest whole-chromosome copy-neutral LOH on chr4. Compared to other autosomes, there is a large proportion of clonal SNVs on chr4 that are homozygous, or in other words, have a median VAF of 1 across sublines evenly distributed across the chromosome (Supplementary Fig. S20). The number of such chr4 clonal SNVs provides evidence for the approximate timing of the underlying chromosomal events. Copy-neutral LOH is likely the result of one of the following two scenarios: (i) LOH followed by whole-chromosome duplication of the remaining haplotype, and (ii) mitotic nondisjunction followed by LOH in the trisomic cell; nevertheless, the scenarios are indistinguishable given current data because neither a subline with one nor a subline with three copies of chr4 was observed.

In the first scenario, we assume the copy-neutral LOH is a result of LOH followed by duplication (Supplementary Fig. S20). Without additional information, we cannot distinguish SNVs obtained before the LOH event and those obtained after LOH and before duplication, as in either case they would appear homozygous; that said, the number of observed homozygous SNVs upperbounds the number of SNVs obtained prior to LOH. Assuming the rate of SNV acquisition per genomic length is constant, the total number of SNVs acquired prior to whole-chromosome duplication is at most two times the number of observed homozygous SNVs. Accounting for the unobserved SNVs in the lost copy in the total number of clonal SNVs, we also add the number of observed homozygous SNVs to the total number of observed chr4 clonal SNVs. The fraction of SNVs acquired prior to whole-chromosome duplication out of total clonal SNVs gives an overestimation of the timing of the duplication, which turns out to be 0.53, or around halfway through the trunk of the tumor phylogeny.

In the second scenario, we assume the copy-neutral LOH is a result of LOH in the trisomic cell formed following mitotic nondisjunction (Supplementary Fig. S20). In this case, without additional information, we cannot resolve the timing of the LOH event from VAF distributions of observed clonal SNVs; however, the timing of mitotic nondijunction can be similarly estimated. The number of observed homozygous SNVs corresponds to the number of SNVs on the chr4 copy with Cdkn2a deletion in a diploid cell prior to mitotic nondisjunction. Again, assuming the rate of SNV acquisition per genomic length is constant, the total number of SNVs acquired prior to mitotic nondisjunction is roughly twice the number of observed homozygous SNVs. In the trisomic cell, the three copies of chr4 acquired SNVs independently. The number of observed number of chr4 clonal heterozygous SNVs, or in other words, those with median VAF  < 1, corresponds to that acquired after mitotic nondisjunction, normalized such that the amount of genomic content is equivalent to two copies of chr4. The fraction of SNVs acquired prior to mitotic nondisjunction out of total SNVs acquired gives an overestimation of the timing of mitotic nondisjunction, which is again, 0.53.

The above analyses lead to the conclusion that, assuming the rate of SNV acquisition per genomic length is constant, the tumor cell first acquired two of the chr4 copy carrying *Cdkn2a* deletion half way through the trunk of the tumor phylogeny, at the latest.

### Subline-specific differential methylation analysis

Given modified base calls, modified base counts for each sample are aggregated using modbam2bed^114^ (–aggregate –mod_base=5mC –cpg –mask). The number of methylated reads (N_*mod*_) and total read count (N_*mod*_ + N_*canon*_) at each CpG site with methylation calls for the 23 subline samples are compiled for smoothing using BSmooth^30^.

At a given branch in the phylogeny, the 23 sublines are bipartitioned into a case group, consisting of sublines in the subtree below the branch, and a control group, consisting of the others. With the case-control group assignment of sublines, we obtain a list of regions that are differentially methylated using BSmooth^30^. To reduce noise due to low coverage, only CpG sites covered by at least 3 reads in all sublines are considered. Signal-to-noise *t*-statistics were computed per CpG site using the smoothed methylation values, and a DMR is defined to be one that spans at least 10 consecutive CpG sites, with corrected *t*-statistics  < . 025 or  > . 975, and with absolute mean differences between groups at least . 2. As replicates are required for DMR testing using BSmooth, DMRs were computed at branches where the bipartition of the sublines they induce both have a size of at least 2.

To get insights into the potential functional significance of the subline-specific DMRs, we explored cancer-associated DMRs from the OncoDB database^54^. While the OncoDB differential methylation database does not include entries to human melanoma/skin cancer (SKCM), we compared the subclonal promoter/enhancer differential methylation identified in our data to those in human orthologs curated in OncoDB for 17 other cancer types (BLCA, BRCA, CESC, CHOL, COAD, ESCA, HNSC, KIRC, KIRP, LIHC, LUAD, LUSC, PAAD, PCPG, PRAD, THCA, and UCEC). Supplementary Figs. S22–S24 showing promoter hypo- or hypermethylation of mouse orthologs from the relevant OncoDB genes.

### Permutation test on the number of DMRs obtained

Given a case group of a certain size, we used a permutation test to assess whether significantly more DMRs were identified in the case group than in the control group. A null distribution was generated by 1000 (or, when the size of the case group is 2, all possible 253) random permutations of the case and control group labels over the sublines. We recorded the number of DMRs identified in each permutation using the permuted labels to implement the criteria described in the section above. The value of *p* is the fraction of permutations that yield a greater or equal number of DMRs than the actual case-control groups of interest. We reject the null hypothesis if *p* < *α* = 0. 05.

### Correlation between the number of DMRs and cumulative SNVs at a branch in the phylogeny

As the numbers of DMRs are not immediately comparable between tests involving case groups of different sizes, we focused on tests involving case groups of size 2 performed at branches parental to exactly 2 leaves, since there is a sufficient number (7) of such branches to assess correlation. The Pearson correlation was assessed between the cumulative numbers of SNVs from the trunk to the branch, and the number of DMRs detected between the 2 sublines below the branch and the rest of the 21 sublines. We reject the null hypothesis if *p* < *α* = 0. 05.

We note that we were unable to choose a larger DMR group size because there are fewer such groups in the phylogeny: for group sizes  > 2, there are at most 4 such nodes in the phylogeny (Supplementary Fig. S17), which is too small of a sample size to evaluate the existence of a linear correlation. While the number of observations for group size = 2 is still small, we were able to see a significant positive correlation between the number of SNVs and DMRs with a sample size of 7.

### Differential gene expression analysis of subline scRNA-seq data

scRNA-seq data was obtained as previously described^24^, and gene-level expected count values for the subline scRNA-seq data were obtained using RSEM^115^. As multiple cells were sequenced from each subline, the analysis treated them as biological replicates of the same subline. Genes with a total count less than 2^10^ were removed, and the decimal expected count values were rounded to the closest digit. DESeq2^116^ was used to assess differential gene expression, and the shrinkage estimator uses a normal prior (coef = 2). The log2 fold change values for the genes of interest were obtained. Note that not all genes of interest have their expression values available in the scRNA-seq data set, and hence, they are left out of the test for correlation.

### Discovering key regions where changes in methylation is monotonic along the branching order

To obtain candidate DMRs to test for monotonicity, we collected regions that exhibited differential methylation among the sublines within the red clade that potentially exhibit such a monotonic trend. As replicates are required for DMR testing with BSmooth, 4 DMR tests were performed with increasingly small subtrees as the case group (and the rest of the sublines in the red clade as the control group): first C11, C16, C8, C7, and C20; then C16, C8, C7, and C20; followed by C8, C7, and C20; and lastly C7 and C20. These groupings were chosen as they reflect cuts along the linear branching order in the red clade. The union of the 4 sets of DMRs were then taken, merging overlapping intervals. As this analysis is motivated by phenotypic observations, we focused our analysis on the regions overlapping (putative) promoters and enhancers of known genes, which we refer to as candidate DMRs. For each of the candidate DMRs, the Mann-Kendall test was performed on four orderings of the sublines – the order in which the sublines were placed (C13, C18, C15, C11, C16, C8, C7, C20), as well as the three variations that may be formed by swapping immediate siblings (C18 and C15, C7, and C20). Kendall’s *τ* value with the greatest absolute value was recorded. We set a minimum threshold of .7 for the absolute value of Kendall’s *τ*, as under assumptions it roughly corresponds to a Pearson correlation coefficient of .9^117^, which is commonly accepted to signify a strong magnitude of effect. Genes whose (putative) promoters or enhancers overlapped regions with an absolute value of Kendall’s *τ* over the threshold .7 were kept. How the statistical significance of the discovery was assessed is discussed in the following section.

### Permutation test on the number of genes associated with monotonic methylation changes along the branching order

We assessed the statistical significance of the number of genes we discover to be associated with monotonic methylation changes along the branching order in the red clade via a permutation test. Fixing the structure of the red subtree, we permuted the position of the 8 sublines in the structure. While there are 8! total permutations of 8 sublines, given the two sets of immediate siblings that are interchangeable in terms of order, and that there is symmetry in terms of the magnitude of correlation for the exact reverse of a particular order, for every permutation, there are 7 others that are equivalent for the purpose of our analysis. We obtain 1000 permutations from the 7! equivalence classes, sampling no more than one permutation from each equivalence class.

For each permuted positioning of 8 sublines in the structure of the red subtree, we obtained the number of genes whose (putative) promoters or enhancers overlapped regions that show monotonicity in terms of methylation following the given order using the algorithm described in the previous section. The *p*-value is the fraction of times the permuted orderings yield a greater or equal number of such genes than the actual ordering of the red clade suggested by Figure 2a. We reject the null hypothesis if *p* < *α* = 0. 05.

### Assessing sequencing statistics on the long-read, bWES, and scRNA-seq data of the 23 sublines

The bWES and scRNA-seq data (several cells per subline) for 23 sublines were obtained from prior studies^24,26^. Coverage data per technology was generated with samtools coverage^91^, and a weighed average was computed over the genome from per contig results. Read quality and count statistics such as N50, Q20(%), Q30(%) statistics were generated using seqkit stats^92^ and samtools view -c^91^. Uniquely mapped reads were generated using sambamba^118^ with different parameters per aligner formatting. For bWES data (aligned with bwa mem^119^), unaligned, secondary, and those reads with XA or SA tags and a MAPQ of 0 were removed. For long-read data (aligned with minimap2^94^), unaligned, secondary and MAPQ of 0 reads were removed. For scRNA-seq data (aligned with STAR^120^), a MAPQ of 255 defines uniquely mapped reads. For per-subline analyses of scRNA-seq data, individual sample bams were merged per subline that they correspond to. Statistics for per-subline scRNA-seq data were generated using the same procedures as per scRNA-seq sample.

### Calling somatic SNVs from bWES and Smart-Seq2 full-length scRNA-seq data on the 23 sublines

Small variants were called from bWES data per subline using DeepVariant(v1.6.1)^28^ with the default parameters provided for the WES model. Called variants were filtered down to SNVs and the target exome capture region. Somatic SNVs were determined by subtracting the long-read ONT splenocyte normal SNVs and the SNVs present in the FVB mouse line, the same methodology as for the long-read sequenced sublines. A bWES splenocyte sample was unavailable, and for equivalent somatic variant determination and comparison, the long-read ONT sample was used.

Small variants were called from scRNA-seq data per sample using DeepVariant(v1.5.0)^28^ with a custom RNA-seq model developed by the DeepVariant team^121^. It was necessary to use v1.5.0 due to DeepVariant transitioning to a different model architecture as of v1.6.0 than what the RNA-seq model was built on. Calling differences are not expected within the autosome between caller versions. Somatic SNVs were determined following the same methodology as for bWES. SNVs were filtered to bWES target regions and limited to those with a coverage of 8x or higher, and to those with a genotype quality of 18 and higher, as recommended per model benchmarking by the developers^121^.

### Calling copy number from bWES and Smart-Seq2 full-length scRNA-seq data on the 23 sublines

CNVkit^122^ was used to obtain the CNA calls from bWES data. CNVkit was run with external bWES data from a normal spleen sample^70^ as reference. The per-bin copy numbers were shown in Supplementary Fig. S21, and the per-segment copy number calls were used to determine the copy number status (i.e., loss, neutral, gain) of available genes in a subline.

Full-length scRNA-seq data were obtained using the Smart-seq2 protocol^123^ for several single cells per subline^24^, and inferCNV^124^ was used to obtain the CNA calls from the data. As Smart-seq2 data for normal melanocytes were not generated for the same study, inferCNV was run with external Smart-seq2 data from mature melanocytes^125^ as reference (cutoff = 1,sd_amplifier = 2,noise_logistic = TRUE; median filtering applied). The inferCNV i6 Hidden Markov Model was used to determine the copy number state, a posterior probability of .5 was used for filtering out likely normal observations, and a per-gene copy number status for each subline was derived.

### Benchmarking using pseudo-bulk simulations

We simulated pseudo-bulk tumor sequencing data by mixing together a subset of sublines, mimicking tumor samples with various degrees of heterogeneity. We created five pseudo-bulk samples, one that merges reads from all sublines into one sample, and four that merge reads from the sublines for each major color clade. For each pseudo-bulk sample, a corresponding set of variants placed by TreeHarmonizer was used as a benchmarking variant set. Variants that were unplaced by TreeHarmonizer were excluded from each call set, in order not to penalize for variants not determined to necessarily be false positives.

We evaluated precision and recall of several state-of-the-art variant callers: DeepSomatic v1.7.0^97^, ClairS v0.3.1^126^, DeepVariant v1.6.1^28^ and Clair3 v1.0.10^127^. Each caller was run with its default settings for ONT R10.4 chemistry. Somatic pseudobulk variants were determined via the same methodology as for per-subline SNV calls, see section above. ClairS showed a slight improvement when using our phasing information, haplotagged using Margin^95^ and Whatshap^96^ per pseudo-bulk sample (the same tools and parameters as per-subline SNV phasing) – rather than using its internal intermediate phasing step. Only this run is shown in Fig. 7b, c. As expected, as the number of clones in a pseudo-bulk dataset increases, the calling power substantially decreases, as seen by the reduced recall.

Overall, somewhat low recall rates on heterogeneous pseudo-bulk simulations suggest that there is still room to improve the variant calling methods for cancer data. This is not surprising, as specialized methods, such as DeepSomatic and ClairS, were released only recently, in parallel with our study. Public release of our dataset and variant calls will encourage further developments. We also note that while DeepSomatic performed better than DeepVariant on pseudo-bulk, we found that DeepVariant was a good fit for variant calling on individual sublines that represent a single clone due to low intrasubline heterogeneity.

### Validation of variant calls using orthogonal technologies and computational methods

Given the complex variational landscape of the B2905 evolution presented above, we sought to quantify the improvement in resolution enabled by long-read sequencing, as compared to the short-read bWES and scRNA-seq of B2905 sublines (Supplementary Note S1). Reassuringly, SNV calls produced from long reads were largely consistent with bWES calls inside the coding regions. As expected, long-read WGS methods covered a substantially higher portion of the genome and produced calls in the non-coding regions, not accessible to bWES and scRNA-seq.

Next, we validated large CNA calls and inerchromosomal fusion with spectral karyotyping (SKY) images available for a subsets of sublines. In most sublines, we observed approximately 90% concordance between long-read calls and SKY experiments, demonstrating high reliability of the long-read calls (Supplementary Note S2).

Finally, we compared DeepVariant, Severus, and Wakhan calls against the other state-of-the-art alternative long-read methods. Overall, we observed high concordance between alternative methods, suggesting that our variant call set used to trace evolutionary history is indeed highly reliable (Supplementary Note S3).

### Ethics statement

All the studies involving the use of animals and their tissues were performed in compliance with the guidelines of Association for Assessment and Accreditation of Laboratory Animal Care (AAALAC) and Animal Care and Use Committee (ACUC) of National Cancer Institute, NIH. NCI is accredited by the AAALAC and follows the Public Health Service Policy on the Care and Use of Laboratory Animals. All animals used in the cited study^20^ were cared for and used humanely according to the following policies: the US Public Health Service Policy on Humane Care and Use of Animals (2015); the Guide for the Care and Use of Laboratory Animals (2011); and the US Government Principles for Utilization and Care of Vertebrate Animals Used in Testing, Research, and Training (1985). No human sample was used in this study.

### Reporting summary

Further information on research design is available in the Nature Portfolio Reporting Summary linked to this article.

## Supplementary information

Supplementary Information

Reporting Summary

Transparent Peer Review file

## Data Availability

The original long-read sequencing data is available in the NCBI / NLM Sequence Read Archive (SRA) under BioProject PRJNA1307171: https://www.ncbi.nlm.nih.gov/bioproject/?term=PRJNA1307171. Other data is available under from SRA: bWES (PRJNA891351): https://www.ncbi.nlm.nih.gov/bioproject/?term=PRJNA891351; scRNA (PRJNA891349): https://www.ncbi.nlm.nih.gov/bioproject/?term=PRJNA891349; bWTS (PRJNA891350): https://www.ncbi.nlm.nih.gov/bioproject/?term=PRJNA891350. Source data for Figs. 2–5 and 7, variant calls, and other processed data are available in a Zenodo archive: 10.5281/zenodo.20072601^128^.

## Code availability

TreeHarmonizer is available at https://github.com/KolmogorovLab/TreeHarmonizer and alternatively at 10.5281/zenodo.20072601.

## References

[1] Yates, L. R. & Campbell, P. J. Evolution of the cancer genome. *Nat. Rev. Genet.* **13**, 795–806 (2012). 23044827 10.1038/nrg3317 PMC3666082

[2] Nowell, P. C. The clonal evolution of tumor cell populations. *Science* **194**, 23–28 (1976). 959840 10.1126/science.959840

[3] Stephens, P. J. et al. Massive genomic rearrangement acquired in a single catastrophic event during cancer development. *Cell* **144**, 27–40 (2011). 21215367 10.1016/j.cell.2010.11.055 PMC3065307

[4] Li, Y. et al. Patterns of somatic structural variation in human cancer genomes. *Nature* **578**, 112–121 (2020). 32025012 10.1038/s41586-019-1913-9 PMC7025897

[5] Wajed, S. A., Laird, P. W. & DeMeester, T. R. Dna methylation: an alternative pathway to cancer. *Ann. Surg.* **234**, 10–20 (2001). 11420478 10.1097/00000658-200107000-00003 PMC1421942

[6] Ciriello, G. et al. Cancer evolution: a multifaceted affair. *Cancer Discov.* **14**, 36–48 (2024). 38047596 10.1158/2159-8290.CD-23-0530 PMC10784746

[7] Black, J. R. & McGranahan, N. Genetic and non-genetic clonal diversity in cancer evolution. *Nat. Rev. Cancer* **21**, 379–392 (2021). 33727690 10.1038/s41568-021-00336-2

[8] Cortés-Ciriano, I., Gulhan, D. C., Lee, J. J.-K., Melloni, G. E. & Park, P. J. Computational analysis of cancer genome sequencing data. *Nat. Rev. Genet.* **23**, 298–314 (2022). 34880424 10.1038/s41576-021-00431-y

[9] Keskus, A. et al. Severus detects somatic structural variation and complex rearrangements in cancer genomes using long-read sequencing. *Nat. Biotechnol.* **44**, 247–257 (2026). 10.1038/s41587-025-02618-8 PMC12483193 40185952

[10] Zaccaria, S. & Raphael, B. J. Characterizing allele-and haplotype-specific copy numbers in single cells with chisel. *Nat. Biotechnol.* **39**, 207–214 (2021). 32879467 10.1038/s41587-020-0661-6 PMC9876616

[11] Olova, N. et al. Comparison of whole-genome bisulfite sequencing library preparation strategies identifies sources of biases affecting dna methylation data. *Genome Biol.* **19**, 1–19 (2018). 29544553 10.1186/s13059-018-1408-2 PMC5856372

[12] Li, Q. et al. Unraveling the hidden complexity of cancer through long-read sequencing. *Genome Res.* **35**, 599–620 (2025). 40113261 10.1101/gr.280041.124 PMC12047254

[13] Elrick, H. et al. Savana: reliable analysis of somatic structural variants and copy number aberrations using long-read sequencing.* Nat. Methods*** 22**, 1436–1446 (2025). 10.1038/s41592-025-02708-0 PMC12240814 40437218

[14] O’Neill, K. et al. Long-read sequencing of an advanced cancer cohort resolves rearrangements, unravels haplotypes, and reveals methylation landscapes.* Cell Genom.*** 4**, 10.1016/j.xgen.2024.100674 (2024). 10.1016/j.xgen.2024.100674 PMC11605692 39406235

[15] Evrony, G. D., Hinch, A. G. & Luo, C. Applications of single-cell dna sequencing. *Annu. Rev. Genom. Hum. Genet.* **22**, 171–197 (2021). 10.1146/annurev-genom-111320-090436 PMC8410678 33722077

[16] Williams, N. et al. Life histories of myeloproliferative neoplasms inferred from phylogenies. *Nature* **602**, 162–168 (2022). 35058638 10.1038/s41586-021-04312-6

[17] Mitchell, E. et al. Clonal dynamics of haematopoiesis across the human lifespan. *Nature* **606**, 343–350 (2022). 35650442 10.1038/s41586-022-04786-y PMC9177428

[18] Cipponi, A. et al. Mtor signaling orchestrates stress-induced mutagenesis, facilitating adaptive evolution in cancer. *Science* **368**, 1127–1131 (2020). 32499442 10.1126/science.aau8768

[19] Noonan, F. P. et al. Neonatal sunburn and melanoma in mice. *Nature* **413**, 271–272 (2001). 11565020 10.1038/35095108

[20] Gruen, C. et al. Melanoma clonal subline analysis uncovers heterogeneity-driven immunotherapy resistance mechanisms. Preprint at 10.1101/2023.04.03.535074 (2023).

[21] Pérez-Guijarro, E. et al. Multimodel preclinical platform predicts clinical response of melanoma to immunotherapy. *Nat. Med.* **26**, 781–791 (2020). 32284588 10.1038/s41591-020-0818-3 PMC8482620

[22] Akbani, R. et al. Genomic classification of cutaneous melanoma. *Cell* **161**, 1681–1696 (2015). 26091043 10.1016/j.cell.2015.05.044 PMC4580370

[23] Rajkumar, S. & Watson, I. R. Molecular characterisation of cutaneous melanoma: creating a framework for targeted and immune therapies. *Br. J. Cancer* **115**, 145–155 (2016). 27336610 10.1038/bjc.2016.195 PMC4947706

[24] Hirsch, M. et al. Stochastic modeling of single-cell gene expression adaptation reveals non-genomic contribution to evolution of tumor subclones.* Cell Syst.*** 16**, 101156 (2025). 10.1016/j.cels.2024.11.013 PMC11867576 39701099

[25] Mehrabadi, F. R. et al. Profiles of expressed mutations in single cells reveal subclonal expansion patterns and therapeutic impact of intratumor heterogeneity. Preprint at 10.1101/2021.03.26.437185 (2021).

[26] Mehrabadi, F. R. et al. A partition function algorithm to evaluate inferred subclonal structures in single-cell sequencing data. in *Research in Computational Molecular Biology*, 409–413 (Springer Nature Switzerland, Cham, 2025).

[27] Kolmogorov, M. et al. Scalable nanopore sequencing of human genomes provides a comprehensive view of haplotype-resolved variation and methylation. *Nat. Methods* **20**, 1483–1492 (2023). 37710018 10.1038/s41592-023-01993-x PMC11222905

[28] Poplin, R. et al. A universal snp and small-indel variant caller using deep neural networks. *Nat. Biotechnol.* **36**, 983–987 (2018). 30247488 10.1038/nbt.4235

[29] Ahmad, T. et al. Wakhan: reconstruction of chromosome-scale copy number profiles of tumor genomes with long-read sequencing. Preprint at 10.64898/2025.12.11.25342098 (2025).

[30] Hansen, K. D., Langmead, B. & Irizarry, R. A. Bsmooth: from whole genome bisulfite sequencing reads to differentially methylated regions. *Genome Biol.* **13**, R83 (2012). 23034175 10.1186/gb-2012-13-10-r83 PMC3491411

[31] Wu, Y. Accurate and efficient cell lineage tree inference from noisy single cell data: the maximum likelihood perfect phylogeny approach. *Bioinformatics* **36**, 742–750 (2019). 10.1093/bioinformatics/btz676 31504211

[32] Hoek, K. S. et al. In vivo switching of human melanoma cells between proliferative and invasive states. *Cancer Res.* **68**, 650–656 (2008). 18245463 10.1158/0008-5472.CAN-07-2491

[33] Day, C.-P. et al. "glowing head” mice: a genetic tool enabling reliable preclinical image-based evaluation of cancers in immunocompetent allografts. *PloS ONE* **9**, e109956 (2014). 25369133 10.1371/journal.pone.0109956 PMC4219677

[34] Rick, J. A., Brock, C. D., Lewanski, A. L., Golcher-Benavides, J. & Wagner, C. E. Reference genome choice and filtering thresholds jointly influence phylogenomic analyses. *Syst. Biol.* **73**, 76–101 (2023). 10.1093/sysbio/syad065 PMC13396950 37881861

[35] Goretsky, A. Kolmogorovlab/treeharmonizer: v1.0.1.* Zenodo*10.5281/zenodo.20072601 (2026).

[36] Sondka, Z. et al. Cosmic: a curated database of somatic variants and clinical data for cancer. *Nucleic Acids Res.* **52**, D1210–D1217 (2023). 10.1093/nar/gkad986 PMC10767972 38183204

[37] Kasar, S. et al. Whole-genome sequencing reveals activation-induced cytidine deaminase signatures during indolent chronic lymphocytic leukaemia evolution. *Nat. Commun.* **6**, 8866 (2015). 26638776 10.1038/ncomms9866 PMC4686820

[38] Nonaka, T. et al. Involvement of activation-induced cytidine deaminase in skin cancer development. * J. Clin. Investig.* **126**, 1367–1382 (2016). 26974156 10.1172/JCI81522 PMC4811119

[39] Halliwell, B. Cell culture, oxidative stress, and antioxidants: avoiding pitfalls. *Biomed. J.* **37**, 99–105 (2014). 24923566 10.4103/2319-4170.128725

[40] Mullard, A. The kras crowd targets its next cancer mutations. *Nat. Rev. Drug Discov.* **22**, 167–171 (2023). 36690752 10.1038/d41573-023-00015-x

[41] Johnson, D. B. et al. Comparative analysis of the gnaq, gna11, sf3b1, and eif1ax driver mutations in melanoma and across the cancer spectrum. *Pigment Cell Melanoma Res.* **29**, 470 (2016). 27089234 10.1111/pcmr.12482 PMC5678944

[42] Larue, L. & Beermann, F. Cutaneous melanoma in genetically modified animals. *Pigment Cell Res.* **20**, 485–497 (2007). 17935491 10.1111/j.1600-0749.2007.00411.x

[43] Damsky Jr, W. E. & Bosenberg, M. Mouse melanoma models and cell lines. *Pigment Cell Melanoma Res.* **23**, 853–859 (2010). 20973935 10.1111/j.1755-148X.2010.00777.x

[44] Yaman, B., Akalin, T. & Kandiloglu, G. Clinicopathological characteristics and mutation profiling in primary cutaneous melanoma. * Am. J. Dermatopathol.* **37**, 389–397 (2015). 25357015 10.1097/DAD.0000000000000241

[45] Shain, A. H. et al. The genetic evolution of melanoma from precursor lesions. *N. Engl. J. Med.* **373**, 1926–1936 (2015). 26559571 10.1056/NEJMoa1502583

[46] Ghaffar, A. et al. Variants of nav3, a neuronal morphogenesis protein, cause intellectual disability, developmental delay, and microcephaly. *Commun. Biol.* **7**, 831 (2024). 38977784 10.1038/s42003-024-06466-1 PMC11231287

[47] Bugaeva, O. et al. Tumour suppressor neuron navigator 3 and matrix metalloproteinase 14 are co-expressed in most melanomas but downregulated in thick tumours. *Acta Derm. Venereol.* **103**, adv00883 (2023). 36883877 10.2340/actadv.v103.298 PMC10010123

[48] Amicone, L. et al. Transgenic expression in the liver of truncated met blocks apoptosis and permits immortalization of hepatocytes.* EMBO J.*** 16**, 495–503 (1997). 10.1093/emboj/16.3.495 PMC1169653 9034332

[49] Levy, C., Khaled, M. & Fisher, D. E. Mitf: master regulator of melanocyte development and melanoma oncogene. *Trends Mol. Med.* **12**, 406–414 (2006). 16899407 10.1016/j.molmed.2006.07.008

[50] Garraway, L. A. et al. Integrative genomic analyses identify mitf as a lineage survival oncogene amplified in malignant melanoma. *Nature* **436**, 117–122 (2005). 16001072 10.1038/nature03664

[51] Kinnaman, M. D. et al. Subclonal somatic copy-number alterations emerge and dominate in recurrent osteosarcoma. *Cancer Res.* **83**, 3796–3812 (2023). 37812025 10.1158/0008-5472.CAN-23-0385 PMC10646480

[52] Pouryazdanparast, P. et al. Distinctive clinical and histologic features in cutaneous melanoma with copy number gains in 8q24. * Am. J. Surg. Pathol.* **36**, 253–264 (2012). 22020039 10.1097/PAS.0b013e31823425cc

[53] Fu, Y., Timp, W. & Sedlazeck, F. J. Computational analysis of dna methylation from long-read sequencing.* Nat. Rev. Genet.* **26**, 620–634 (2025). 10.1038/s41576-025-00822-5 PMC12882999 40155770

[54] Tang, G., Cho, M. & Wang, X. Oncodb: an interactive online database for analysis of gene expression and viral infection in cancer. *Nucleic Acids Res.* **50**, D1334–D1339 (2021). 10.1093/nar/gkab970 PMC8728272 34718715

[55] Martin, F. J. et al. Ensembl 2023. *Nucleic Acids Res.* **51**, D933–D941 (2022). 10.1093/nar/gkac958 PMC9825606 36318249

[56] Frankish, A. et al. Gencode reference annotation for the human and mouse genomes. *Nucleic Acids Res.* **47**, D766–D773 (2019). 30357393 10.1093/nar/gky955 PMC6323946

[57] Dreos, R., Ambrosini, G., Périer, R. C. & Bucher, P. The Eukaryotic Promoter Database: expansion of EPDnew and new promoter analysis tools. *Nucleic Acids Res.* **43**, D92–D96 (2014). 25378343 10.1093/nar/gku1111 PMC4383928

[58] Meylan, P., Dreos, R., Ambrosini, G., Groux, R. & Bucher, P. EPD in 2020: enhanced data visualization and extension to ncRNA promoters. *Nucleic Acids Res.* **48**, D65–D69 (2019). 10.1093/nar/gkz1014 PMC7145694 31680159

[59] ENCODE Project, C. An integrated encyclopedia of dna elements in the human genome. *Nature* **489**, 57–74 (2012). 22955616 10.1038/nature11247 PMC3439153

[60] Luo, Y. et al. New developments on the encyclopedia of dna elements (encode) data portal. *Nucleic Acids Res.* **48**, D882–D889 (2020). 31713622 10.1093/nar/gkz1062 PMC7061942

[61] Hitz, B. C. et al. The encode uniform analysis pipelines. Preprint at 10.1101/2023.04.04.535623 (2023).

[62] Abascal, F. et al. Expanded encyclopaedias of dna elements in the human and mouse genomes. *Nature* **583**, 699–710 (2020). 32728249 10.1038/s41586-020-2493-4 PMC7410828

[63] Casazza, A. et al. Sema3e–plexin d1 signaling drives human cancer cell invasiveness and metastatic spreading in mice. * J. Clin. Investig.* **120**, 2684–2698 (2010). 20664171 10.1172/JCI42118 PMC2912191

[64] Wen, J. et al. Genetic and transcriptional insights into immune checkpoint blockade response and survival: lessons from melanoma and beyond. *J. Transl. Med.* **23**, 467 (2025). 40269853 10.1186/s12967-025-06467-6 PMC12020166

[65] Perego, M. et al. A slow-cycling subpopulation of melanoma cells with highly invasive properties. *Oncogene* **37**, 302–312 (2018). 28925403 10.1038/onc.2017.341 PMC5799768

[66] Chen, E. et al. A novel hepatocellular carcinoma-specific mtorc1-related signature for anticipating prognosis and immunotherapy. *Aging (Albany NY)* **15**, 7933–7955 (2023). 37589508 10.18632/aging.204862 PMC10497017

[67] Roshan, M. K. et al. Role of akt and mtor signaling pathways in the induction of epithelial-mesenchymal transition (emt) process. *Biochimie* **165**, 229–234 (2019). 31401189 10.1016/j.biochi.2019.08.003

[68] Zeng, H. et al. Bi-allelic loss of cdkn2a initiates melanoma invasion via brn2 activation. *Cancer Cell* **34**, 56–68 (2018). 29990501 10.1016/j.ccell.2018.05.014 PMC6084788

[69] Udart, M., Utikal, J., Krähn, G. M. & Peter, R. U. Chromosome 7 aneusomy. a marker for metastatic melanoma?: expression of the epidermal growth factor receptor gene and chromosome 7 aneusomy in nevi, primary malignant melanomas and metastases. *Neoplasia* **3**, 245–254 (2001). 11494118 10.1038/sj.neo/7900156 PMC1505589

[70] Wolf, Y. et al. Uvb-induced tumor heterogeneity diminishes immune response in melanoma. *Cell* **179**, 219–235.e21 (2019). 31522890 10.1016/j.cell.2019.08.032 PMC6863386

[71] Corces, M. R. et al. The chromatin accessibility landscape of primary human cancers. *Science* **362**, eaav1898 (2018). 30361341 10.1126/science.aav1898 PMC6408149

[72] McGranahan, N. & Swanton, C. Clonal heterogeneity and tumor evolution: past, present, and the future. *Cell* **168**, 613–628 (2017). 28187284 10.1016/j.cell.2017.01.018

[73] McPherson, A. et al. Divergent modes of clonal spread and intraperitoneal mixing in high-grade serous ovarian cancer. *Nat. Genet.* **48**, 758–767 (2016). 27182968 10.1038/ng.3573

[74] Yates, L. R. et al. Subclonal diversification of primary breast cancer revealed by multiregion sequencing. *Nat. Med.* **21**, 751–759 (2015). 26099045 10.1038/nm.3886 PMC4500826

[75] Funnell, T. et al. Single-cell genomic variation induced by mutational processes in cancer. *Nature* **612**, 106–115 (2022). 36289342 10.1038/s41586-022-05249-0 PMC9712114

[76] Leppä, A.-M. et al. Single-cell multiomics analysis reveals dynamic clonal evolution and targetable phenotypes in acute myeloid leukemia with complex karyotype.* Nat. Genet.*** 56**, 2790–2803 (2024). 10.1038/s41588-024-01999-x PMC11631769 39587361

[77] Gaiti, F. et al. Epigenetic evolution and lineage histories of chronic lymphocytic leukaemia. *Nature* **569**, 576–580 (2019). 31092926 10.1038/s41586-019-1198-z PMC6533116

[78] Chaligne, R. et al. Epigenetic encoding, heritability and plasticity of glioma transcriptional cell states. *Nat. Genet.* **53**, 1469–1479 (2021). 34594037 10.1038/s41588-021-00927-7 PMC8675181

[79] Liu, Y. et al. Single-cell methylation sequencing data reveal succinct metastatic migration histories and tumor progression models. *Genome Res.* **33**, 1089–1100 (2023). 37316351 10.1101/gr.277608.122 PMC10538489

[80] Chen, M., Fu, R., Chen, Y., Li, L. & Wang, S.-W. High-resolution, noninvasive single-cell lineage tracing in mice and humans based on dna methylation epimutations. *Nat. Methods* **22**, 488–498 (2025). 39820752 10.1038/s41592-024-02567-1

[81] Hansen, K. D. et al. Increased methylation variation in epigenetic domains across cancer types. *Nat. Genet.* **43**, 768–775 (2011). 21706001 10.1038/ng.865 PMC3145050

[82] Johnson, K. C. et al. Single-cell multimodal glioma analyses identify epigenetic regulators of cellular plasticity and environmental stress response. *Nat. Genet.* **53**, 1456–1468 (2021). 34594038 10.1038/s41588-021-00926-8 PMC8570135

[83] Chen, F. et al. Global dna methylation differences involving germline structural variation impact gene expression in pediatric brain tumors. *Nat. Commun.* **16**, 4713 (2025). 40399292 10.1038/s41467-025-60110-y PMC12095544

[84] Zhao, S. G. et al. The dna methylation landscape of advanced prostate cancer. *Nat. Genet.* **52**, 778–789 (2020). 32661416 10.1038/s41588-020-0648-8 PMC7454228

[85] Rausch, T. et al. Long-read sequencing of diagnosis and post-therapy medulloblastoma reveals complex rearrangement patterns and epigenetic signatures. *Cell Genom.* **3**, 100281 (2023). 37082141 10.1016/j.xgen.2023.100281 PMC10112291

[86] Kim, J.-Y. et al. Prognostic value of structural variants in early breast cancer patients. *NPJ Breast Cancer* **10**, 64 (2024). 39068172 10.1038/s41523-024-00669-9 PMC11283467

[87] Filipski, K. et al. Dna methylation-based prediction of response to immune checkpoint inhibition in metastatic melanoma. *J. Immunother. Cancer* **9**, e002226 (2021). 34281986 10.1136/jitc-2020-002226 PMC8291310

[88] Jerez, P. A., Billingsley, K. J., Blauwendraat, C. et al. Processing frozen cells for population-scale oxford nanopore long-read dna sequencing sop. protocols.io. 10.17504/protocols.io.5jyl8pnk7g2w/v1 (2022).

[89] Oxford Nanopore Technologies. Guppy. https://nanoporetech.com/software/other/guppy/history?version=6-4-2 (2022).

[90] Oxford Nanopore Technologies. Dorado. https://github.com/nanoporetech/dorado (2023).

[91] Danecek, P. et al. Twelve years of samtools and bcftools.* GigaScience*** 10**, 10.1093/gigascience/giab008 (2021). 10.1093/gigascience/giab008 PMC7931819 33590861

[92] Shen, W., Sipos, B. & Zhao, L. Seqkit2: A swiss army knife for sequence and alignment processing.* iMeta*** 3**, 10.1002/imt2.191 (2024). 10.1002/imt2.191 PMC11183193 38898985

[93] Leger, A. & Leonardi, T. pycoqc, interactive quality control for oxford nanopore sequencing. *J. Open Source Softw.* **4**, 1236 (2019).

[94] Li, H. Minimap2: pairwise alignment for nucleotide sequences. *Bioinformatics* **34**, 3094–3100 (2018). 29750242 10.1093/bioinformatics/bty191 PMC6137996

[95] Shafin, K. et al. Haplotype-aware variant calling with pepper-margin-deepvariant enables high accuracy in nanopore long-reads. *Nat. Methods* **18**, 1322–1332 (2021). 34725481 10.1038/s41592-021-01299-w PMC8571015

[96] Martin, M. et al. Whatshap: fast and accurate read-based phasing. Preprint at 10.1101/085050 (2016).

[97] Park, J. et al. Deepsomatic: Accurate somatic small variant discovery for multiple sequencing technologies. *Nat. Biotechnol.*10.1038/s41587-025-02839-x (2025). 10.1038/s41587-025-02839-x 41102444

[98] Li, H. et al. A synthetic-diploid benchmark for accurate variant-calling evaluation. *Nat. Methods* **15**, 595–597 (2018). 30013044 10.1038/s41592-018-0054-7 PMC6341484

[99] López-Cortegano, E. et al. The rate and spectrum of new mutations in mice inferred by long-read sequencing. *Genome Res.* **35**, 43–54 (2025). 39622636 10.1101/gr.279982.124 PMC11789640

[100] Rhie, A. et al. The complete sequence of a human y chromosome. *Nature* **621**, 344–354 (2023). 37612512 10.1038/s41586-023-06457-y PMC10752217

[101] El-Kebir, M. SPhyR: tumor phylogeny estimation from single-cell sequencing data under loss and error. *Bioinformatics* **34**, i671–i679 (2018). 30423070 10.1093/bioinformatics/bty589 PMC6153375

[102] Ciccolella, S. et al. Inferring cancer progression from single-cell sequencing while allowing mutation losses. *Bioinformatics* **37**, 326–333 (2020). 10.1093/bioinformatics/btaa722 PMC8058767 32805010

[103] Sashittal, P., Zhang, H., Iacobuzio-Donahue, C. A. & Raphael, B. J. ConDoR: tumor phylogeny inference with a copy-number constrained mutation loss model.* Genome Biol.*** 24**, 272 (2023). 10.1186/s13059-023-03106-5 PMC10688497 38037115

[104] Díaz-Gay, M. et al. Assigning mutational signatures to individual samples and individual somatic mutations with sigprofilerassignment. *Bioinformatics* **39**, btad756 (2023). 38096571 10.1093/bioinformatics/btad756 PMC10746860

[105] Medo, M., Ng, C. K. Y. & Medová, M. A comprehensive comparison of tools for fitting mutational signatures. *Nat. Commun.* **15**, 9467 (2024). 39487150 10.1038/s41467-024-53711-6 PMC11530434

[106] McLaren, W. et al. The ensembl variant effect predictor. *Genome Biol.* **17**, 122 (2016). 27268795 10.1186/s13059-016-0974-4 PMC4893825

[107] Ashburner, M. et al. Gene ontology: tool for the unification of biology. *Nat. Genet.* **25**, 25–29 (2000). 10802651 10.1038/75556 PMC3037419

[108] Consortium, T. G. O. et al. The Gene Ontology knowledgebase in 2023. *Genetics* **224**, iyad031 (2023). 36866529 10.1093/genetics/iyad031 PMC10158837

[109] Yu, G., Wang, L.-G., Han, Y. & He, Q.-Y. clusterprofiler: an r package for comparing biological themes among gene clusters. *OMICS* **16**, 284–287 (2012). 22455463 10.1089/omi.2011.0118 PMC3339379

[110] Zingg, D. et al. The epigenetic modifier ezh2 controls melanoma growth and metastasis through silencing of distinct tumour suppressors. *Nat. Commun.* **6**, 6051 (2015). 25609585 10.1038/ncomms7051

[111] Hill, V. K., Kim, J.-S. & Waldman, T. Cohesin mutations in human cancer. *Biochim. et. Biophys. Acta Rev. Cancer* **1866**, 1–11 (2016). 10.1016/j.bbcan.2016.05.002 PMC4980180 27207471

[112] Guo, Y. et al. Tert promoter mutations and telomerase in melanoma. *J. Oncol.* **2022**, 6300329 (2022). 35903534 10.1155/2022/6300329 PMC9325578

[113] Soussi, T. The p53 tumor suppressor gene: from molecular biology to clinical investigation. *Ann. N. Y. Acad. Sci.* **910**, 121–139 (2000). 10911910 10.1111/j.1749-6632.2000.tb06705.x

[114] Ltd., O. N. T. modbam2bed.https://github.com/epi2me-labs/modbam2bed (2023).

[115] Li, B. & Dewey, C. N. Rsem: accurate transcript quantification from rna-seq data with or without a reference genome. *BMC Bioinform.* **12**, 323 (2011). 10.1186/1471-2105-12-323 PMC3163565 21816040

[116] Love, M. I., Huber, W. & Anders, S. Moderated estimation of fold change and dispersion for rna-seq data with deseq2. *Genome Biol.* **15**, 550 (2014). 25516281 10.1186/s13059-014-0550-8 PMC4302049

[117] Gilpin, A. R. Table for conversion of kendall’s tau to spearman’s rho within the context of measures of magnitude of effect for meta-analysis. *Educ. Psychol. Meas.* **53**, 87–92 (1993).

[118] Tarasov, A., Vilella, A. J., Cuppen, E., Nijman, I. J. & Prins, P. Sambamba: fast processing of ngs alignment formats. *Bioinformatics* **31**, 2032–2034 (2015). 25697820 10.1093/bioinformatics/btv098 PMC4765878

[119] Li, H. et al. Aligning sequence reads, clone sequences and assembly contigs with bwa-mem. Preprint at 10.48550/arXiv.1303.3997 (2013).

[120] Dobin, A. et al. Star: ultrafast universal rna-seq aligner. *Bioinformatics* **29**, 15–21 (2012). 23104886 10.1093/bioinformatics/bts635 PMC3530905

[121] Cook, D. E. et al. A deep-learning-based rna-seq germline variant caller.* Bioinform. Adv.*** 3**, 10.1093/bioadv/vbad062 (2023). 10.1093/bioadv/vbad062 PMC10320079 37416509

[122] Talevich, E., Shain, A. H., Botton, T. & Bastian, B. C. Cnvkit: Genome-wide copy number detection and visualization from targeted dna sequencing. *PLOS Comput. Biol.* **12**, 1–18 (2016). 10.1371/journal.pcbi.1004873 PMC4839673 27100738

[123] Picelli, S. et al. Full-length rna-seq from single cells using smart-seq2. *Nat. Protoc.* **9**, 171–181 (2014). 24385147 10.1038/nprot.2014.006

[124] Tickle, T., Tirosh, I., Georgescu, C., Brown, M. & Hass, B. Infercnv of the trinity ctat project.https://github.com/broadinstitute/inferCNV (2024).

[125] Infarinato, N. R. et al. Bmp signaling: at the gate between activated melanocyte stem cells and differentiation. *Genes Dev.* **34**, 1713–1734 (2020). 33184221 10.1101/gad.340281.120 PMC7706702

[126] Zheng, Z. et al. Clairs: a deep-learning method for long-read somatic small variant calling.* Nat. Methods*10.1038/s41592-026-03152-4 (2023). 10.1038/s41592-026-03152-4 PMC13441973 42387002

[127] Zheng, Z. et al. Symphonizing pileup and full-alignment for deep learning-based long-read variant calling. *Nat. Comput. Sci.* **2**, 797–803 (2022). 38177392 10.1038/s43588-022-00387-x

[128] Y., Liu. Long-read sequencing of single cell-derived melanoma subclones reveals divergent and parallel genomic and epigenomic evolutionary trajectories (1.0).* Zenodo*10.5281/zenodo.20072601 (2025). 10.1038/s41467-026-75172-9 PMC13493970 42463712

[129] NIAID Visual & Medical Arts. Lab mouse. NIAID NIH BIOART Source https://bioart.niaid.nih.gov/bioart/279 (2024).

