# Supplementary material for: Long-read sequencing of single cell-derived melanoma sublines reveals divergent and parallel genomic and epigenomic evolutionary trajectories
Source: Nat Commun. 2026 Jul 16;17:8740. doi: 10.1038/s41467-026-75172-9 (PMC13493970; doi:10.1038/s41467-026-75172-9)
Supplement: Supplementary file 1 [file 41467_2026_75172_MOESM1_ESM.pdf]

Supplemental Material

Long-read sequencing of single cell-derived melanoma sublines reveals divergent and parallel genomic and epigenomic evolutionary trajectories

Yuelin Liu*, Anton Goretsky*, Ayse Keskus, Salem Malikic, Tanveer Ahmad, E. Michael Gertz, Farid Rashidi Mehrabadi, Michael Kelly, Maria Hernandez, Charlie Seibert, Juan Manuel Caravaca, Kayla Kline, Yongmei Zhao, Ying Wu, Biraj Shrestha, Bao Tran, Arindam Ghosh, Xiwen Cui, Antonella Sassano, Laksh Malik, Breeana Baker, Cornelis Blauwendraat, Kimberley J. Billingsley, Eva Perez-Guijarro, Glenn Merlino, Erin K. Molloy, S. Cenk Sahinalp#, Chi-Ping Day#, Mikhail Kolmogorov#

List of Supplementary Tables

S1 **Long-read ONT sequencing statistics for the 23 sublines, spleen, and parental B2905 line.** Q20(%) (and Q30(%)) represents the percentage of bases that have a PHRED quality score of 20 (and 30) or over. Avg Qual and Med Qual are the mean and median PHRED quality scores for all passing reads, respectively. Means of %, average and median statistics are recalculated with total base count. Mean and median of N50 are calculated using the final N50 output per subline. 6

S2 **WES sequencing statistics for the 23 sublines, spleen, and parental B2905 line.** Q20(%) (and Q30(%)) represents the percentage of bases that have a PHRED quality score of 20 (and 30) or over. Avg Qual and Med Qual are the mean and median PHRED quality scores for all passing reads, respectively. Means of %, average and median statistics are recalculated with total base count. Mean and median of N50 are calculated using the final N50 output per subline. 6

S3 **Phenotypes of the 4 major clades.** The phenotype for each of the major clades was determined by features defined by the behavior or characteristics of (select) sublines from the clade previously characterized (Methods) [8]. 7

S4 **`TreeHarmonizer` SNV placement statistics relative to each subline.** Clonal, subclonal and private represent the percentages of placed SNVs that were called in the respective subline. Regenotyped SNVs are those who were assigned the "lost variant" genotype by `TreeHarmonizer`. Total placed and unplaced counts are respective to each subline. Placed and unplaced counts contain duplicates (representing the same variant) between sublines. 7

S5 **`TreeHarmonizer` SV placement statistics respective to each subline.** Clonal, subclonal and private represent the percentages of placed SVs that were called in the respective subline. Total placed and unplaced counts are respective to each subline. Placed and unplaced counts contain duplicates (representing the same variant) between sublines. . . 8

S6 **DMRs associated with the red clade that overlap putative promoter or putative proximal enhancers of known genes.** A region is said to be a putative promoter for a gene if it is within -350bp to +50bp of the gene's annotated transcriptional start site (Ensembl Release 102) [14, 6]. Of the 17 putative promoters, 9 are also annotated in the EPDnew (v.003) database of experimentally validated promoters [3, 16]. A region is said to be a putative proximal enhancer for a gene if is has enhancer-like signature [1] and is within 2Kbp of the annotated transcriptional start site [5, 13, 10] of the gene. Note that *Gata2* was an outlier in that its putative promoter was noted to be hypermethylated while its expression has a positive log2 fold change (Figure 5d.); however, this may be due to the hypomethylation of a putative promoter of *Gata2* alternative isoforms, which went undetected as its mean methylation difference is not large enough to meet our criteria (Methods). 9
S7 **Weighted mean ploidy per subline.** Mean of weighted ploidies is also weighted. Median is calculated over subline averages. 10
S8 Comparison of CNAs and fusions detected by SKY and Wakhan across samples. The table shows the original SKY calls before correction. Several recurrent translocations were systematically miscalled in SKY images because the corresponding chromosomes have very similar probe colors: chr8-chr9 in SKY corresponds to chr8-chr10 in Severus; chr5-chr8 in SKY corresponds to chr5-chr18 in Severus. In addition, chr5-chr18 may be systematically missed in SKY because of the probe colors similarity. (s) denotes subclonal event. 36
S9 **SV counts by phylogenetic tree placement category for Sniffles2 and Severus.** . . 39
S10 **Summary of SV failure modes and discrepancies between Sniffles2 and Severus across major SV categories.** 41
S11 **CNV concordance statistics between Wakhan and CNVkit.** Exact match percentage refers to the percentage of equivalent copy number calls between the two callers over all 50kb bins. A two-sided Pearson correlation test was used to evaluate concordance between caller outputs. Provided are the correlation coefficient (r) and corresponding p-values. Note that p-values are very low due to the very high amount of bins, and statistical significance is trivial to achieve in this experimental context. The analysis focuses on the relationship between the correlation coefficient and the exact match percentage. . . 42

List of Supplementary Figures

S1 **SNVs and SVs placed by `TreeHarmonizer`. a.** Cumulative number of placed SNVs and SVs per subline, colored by clade. Medians are marked by dashed lines. The correlation between cumulative number of SNVs and SVs was statistically insignificant (rs(21)=.239, p=.2713) **b.** The number of SNVs and the number of SVs placed at each branch of the phylogeny strongly correlate with each other. **c.** Variant allele frequency distributions of placed SNVs. **d.** Variant allele frequency distributions of placed SVs. 11
S2 **Comparison among long-read, bWES, and scRNA-seq data of the 23 sublines. a.** Per-subline autosomal genomic coverage (%), uniquely mapped reads (%), and count of unique somatic SNVs profiled (Methods). **b.** Counts of unique, non-private somatic SNVs called within bWES target regions across the 23 sublines for each technology. **c.** Count of `Severus`-profiled SVs whose breakpoints have sufficient read coverage (200x after aggregating all reads from all sublines/cells) in bWES and scRNA-seq data. **d.** Example of genomic coverage at a near-exonic breakpoint associated with a subclonal SV across the 3 technologies. **e.** Example of genomic coverage at an intergenic breakpoint associated with a subclonal SV across the 3 technologies. **f.** Agreement on gene copy-number status (neutral, gain, loss) called from data from the 3 technologies across all sublines. For any subline, only genes with copy number calls available in all 3 technologies are included. **g.** Improvement in terms of partition function values [15] among variants impacted by filtering during tree construction, following variant placement by `TreeHarmonizer`. 12
S3 **Mutational signature activities along the tumor phylogeny.** In each panel, a point represents a branch in the phylogeny. A branch can either be categorized into truncal, internal or terminal, or by the color of the clade it is in. ANOVA with post-hoc Tukey HSD (Honestly Significant Difference) Test was performed, and the adjusted p-values of statistically significant pairs are reported. 13

S4 **SNVs along the tumor phylogeny. a.** dN/dS value distributions among truncal, internal, and terminal branches. dN/dS values at internal branches are significantly lower than that at terminal branches by one-tailed *t*-test. **b.** Select enrichment of genes harboring non-synonymous clonal mutations among GO Biological Process term. **c.-f.** Select enrichment of genes uniquely harboring non-synonymous mutations in the green (c.), orange (d.), blue (e.), and red (f.) clade among GO Biological Process terms. 14
S5 **SNV density and SV events present in individual sublines in the green clade.** . 15
S6 **SNV density and SV events present in individual sublines in the orange clade.**. 16
S7 **SNV density and SV events present in individual sublines in the blue clade.** . 17
S8 **SNV density and SV events present in individual sublines in the red clade.** . . 18
S9 **SV-disrupted genes and gene fusion annotated by `Padfoot`. a.** A clonal deletion affects exon 4-9 of *Nav3*. Long-read genomic reads of an representative subline from each major clade are shown. **b.** A subclonal deletion in C3 and C14 in *Met* resulted in the loss of exon 4-11. Long-read genomic reads from sublines C3 and C14 are shown. **c.** A subclonal deletion in C3 and C14 resulted in the fusion of *Map4* and *Smarcc1*. Long-read genomic reads from sublines C3 and C14, as well as scRNA-seq reads from a C14 cell corroborating the fusion event are shown. 19
S10 **Mouse orthologs of COSMIC genes impacted by (1) non-synonymous SNV, (2) SV breakpoint, (3) CNA, or (4) (hypo/hyper-) DMRs in their (putative) enhancers or promoters in 23 sublines (1/7).** Genes with "NA" copy number in a subline span detected CNA boundaries and therefore cannot be assigned a unique copy number. 20
S11 **Mouse orthologs of COSMIC genes impacted by (1) non-synonymous SNV, (2) SV breakpoint, (3) CNA, or (4) (hypo/hyper-) DMRs in their (putative) enhancers or promoters in 23 sublines (2/7).** Genes with "NA" copy number in a subline span detected CNA boundaries and therefore cannot be assigned a unique copy number. 21
S12 **Mouse orthologs of COSMIC genes impacted by (1) non-synonymous SNV, (2) SV breakpoint, (3) CNA, or (4) (hypo/hyper-) DMRs in their (putative) enhancers or promoters in 23 sublines (3/7).** Genes with "NA" copy number in a subline span detected CNA boundaries and therefore cannot be assigned a unique copy number. 22
S13 **Mouse orthologs of COSMIC genes impacted by (1) non-synonymous SNV, (2) SV breakpoint, (3) CNA, or (4) (hypo/hyper-) DMRs in their (putative) enhancers or promoters in 23 sublines (4/7).** Genes with "NA" copy number in a subline span detected CNA boundaries and therefore cannot be assigned a unique copy number. 23
S14 **Mouse orthologs of COSMIC genes impacted by (1) non-synonymous SNV, (2) SV breakpoint, (3) CNA, or (4) (hypo/hyper-) DMRs in their (putative) enhancers or promoters in 23 sublines (5/7).** Genes with "NA" copy number in a subline span detected CNA boundaries and therefore cannot be assigned a unique copy number. 24
S15 **Mouse orthologs of COSMIC genes impacted by (1) non-synonymous SNV, (2) SV breakpoint, (3) CNA, or (4) (hypo/hyper-) DMRs in their (putative) enhancers or promoters in 23 sublines (6/7).** Genes with "NA" copy number in a subline span detected CNA boundaries and therefore cannot be assigned a unique copy number. 25
S16 **Mouse orthologs of COSMIC genes impacted by (1) non-synonymous SNV, (2) SV breakpoint, (3) CNA, or (4) (hypo/hyper-) DMRs in their (putative) enhancers or promoters in 23 sublines (7/7).** Genes with "NA" copy number in a subline span detected CNA boundaries and therefore cannot be assigned a unique copy number. 26

S17 **Permutation tests on the number of DMRs obtained for all subtree sizes of the phylogeny in Figure 2a.** In each panel, "# Case" indicates the number of leaves in the subtree below the branch at which the test was performed. See Figure 5b. for "# Case = 8". Note that due to symmetry, no additional permutation test is done for "# Case = 16" to account for the subtree consisting of the orange, blue, and red in Figure 2a., as the empirical null distribution is identical to that of "# Case = 7" and the number of DMRs obtained is identical to that of the subtree of size 7 colored in green in Figure 2a. 27
S18 **Mouse orthologs of genes whose promoters are documented to be impacted by hypo- or hypermethylation in at least one of 17 types of human cancer according to OncoDB[27] and were found to be similarly impacted by promoter or enhancer differential methylation along the subline phylogeny (1/3).** 28
S19 **Mouse orthologs of genes whose promoters are documented to be impacted by hypo- or hypermethylation in at least one of 17 types of human cancer according to OncoDB[27] and were found to be similarly impacted by promoter or enhancer differential methylation along the subline phylogeny (2/3).** 29
S20 **Mouse orthologs of genes whose promoters are documented to be impacted by hypo- or hypermethylation in at least one of 17 types of human cancer according to OncoDB[27] and were found to be similarly impacted by promoter or enhancer differential methylation along the subline phylogeny (3/3).** 30
S21 **Associating truncating *Dnah6* mutation with (haplotype-specific) methylation of *Dnah6* promoter in major clades.** One representative subline from each major clade is shown. Clonal truncating mutation *Dnah6* p.W1807* is marked in the left panel displaying unphased reads, and a heterozygous variant chr6: 73,220,494 [C>T] is marked on the right panel with reads phased according to the variant. In C11, the high VAF of *Dnah6* p.W1807*, together with the homozygosity of chr6: 73,220,494 [C>T], not only indicates LOH in C11, but also provides evidence that the two variants exist on the same haplotype. As the phased reads on the right panel are also colored by DNA methylation, we observe that methylation at *Dnah6* promoter is specific to the haplotype harboring chr6: 73,220,494 [C>T], and hence *Dnah6* p.W1807*. We observe haplotype-specific methylation of the *Dnah6* promoter in the green, orange, and blue clade, and hypermethylation in the red clade. Note that while here we observe a few reads from the wild-type copy of *Dnah6* in C11, we see a complete loss of the wild-type copy of *Dnah6* in all other sublines in the red clade, and along with that a complete hypermethylation of the *Dnah6* promoter. 31
S22 **Delay of growth initiation (DGI) of sublines in C57BL/6 and nude mice.** DGI is defined as the number of days between cell implantation in a mouse and the tumor started continuous growing without regression. **a.** One million cells of each subline were implanted into each of the five C57BL/6 mice (Day 1). The tumor size was measured twice a week. The mean DGI of the sublines in each clade, as well as the median across sublines means within a clade, was shown. No tumor was grown from any mice implanted with cells of C19, C6, or C13. Note that two mice implanted with C5 cells did not grow tumor, and they were not included in the calculation of mean DGI. These results suggested that, on average, sublines in blue clade induced the stronger host immunity than those in other clades. **b.** To investigate the association of the DGI with host immunity, the subline from each clade with the highest DGI following C57BL/6 implantation was tested in immunocompromised host. For each of C19 (green clade), C5 (orange clade), C6 (blue clade), and C13 (red clade), one million cells were implanted into each of the five immunocompromised nude mice (Day 1), and the DGI values measured from each mouse, as well as the median across mouse DGI values for a subline are shown. As expected, we observed the tumor grew in all mice with diminishing DGI as compared to the implantation in C57BL/6 mice. These results indicated that DGI is associated with subline response to host immunity. 32
S23 **SNV evidence for the timing clonal CNA events.** **a.** Median (across sublines) VAF distribution for clonal SNVs on chr4 and other autosomes. **b.** Genomic distributions of homozygous (median VAF = 1) and heterozygous (median VAF < 1) chr4 clonal SNVs. **c.** Two indistinguishable scenarios that may have resulted in the copy-neutral LOH. . . . 32

S24 **CNA profiles of 23 sublines obtained using long-read, bWES, and scRNA-seq data. a.** Long-read-derived CNA profiles using `Wakhan` [2] (Figure 2e.; Methods) **b.** CNA profiles derived by `CNVkit` [25] from bWES data [15](Methods). **c.** CNA profiles derived by `inferCNV` [28] from full-length scRNA-seq data [9] obtained using the Smart-seq2 protocol [18] (Methods). 33
S25 **Spectral Karyotyping (SKY) imaging data for available sublines, as well as for the parental line.** 37
S26 **Whole chromosome paint validation of chr8-chr10 translocation in C1 using two chromosome probes.** 38
S27 **UpSet plots showing SNV concordance between variant callers, stratified by clonality.** Clonal here is defined as the presence of a unique variant in all sublines, subclonal as presence in two or more but not all, and private as presence in one subline. . 40
S28 **The correlation between number of SNVs and Sniffles2 SVs calls placed at each branch of the phylogeny.** 40
S29 **CNA profiles of the 23 sublines using long-read data across CNA callers. a.** CNA integer calls as obtained by CNVkit using 50kb bins. **b.** CNA integer calls as obtained by Wakhan using 50kb bins. Telomere and centromere regions are not shown or colored. 43
S30 Extending SCARLET and ConDoR benchmarking to higher quantities of variants. Plotted are the observed points, and a projected dashed line based on the curve of best fit per sampling seed. A 95% confidence interval is also shown per seed. ScisTree + TreeHarmonizer represents the total runtime of the two tools together on this dataset as described in Methods. 44

List of Supplementary Notes

S1 Long-read whole-genome sequencing substantially improves resolution of genomic variants 34

S2 Validation of large long-read SV and CNA calls against spectral karyotyping (SKY) images 35

S3 Comparison of SNV, SV and CNA calls against orthogonal tools 39

S4 Evaluating alternative methods for joint SNV and CNA phylogeny reconstruction 44

Subline	# Reads	Total Length	Max Length	Mean Length	Avg Depth	N50	Q20(%)	Q30(%)	Avg Qual	Med Qual
C1	3,878,605	83,059,872,680	281,118	21,414.9	29.18	29,251	87.94	74.02	18.44	19.891
C3	3,847,028	86,209,871,989	966,796	22,409.5	31.18	32,971	80.13	64.80	17.95	20.000
C4	1,883,328	37,715,867,826	307,236	20,026.2	13.67	29,906	80.48	63.96	16.70	18.000
C5	3,628,129	75,421,594,079	821,988	20,788.0	27.22	34,327	80.43	65.20	18.43	20.000
C6	3,902,522	84,360,107,492	695,762	21,616.8	30.58	32,074	82.14	66.47	18.21	20.000
C7	4,822,610	79,929,281,043	854,291	16,573.9	28.73	30,743	80.94	64.79	18.16	19.000
C8	4,267,557	82,984,330,581	247,823	19,445.4	30.43	26,286	87.31	72.82	18.22	19.251
C9	9,876,652	61,339,127,538	110,146	6,210.5	23.19	11,383	81.41	65.28	16.14	17.000
C10	3,855,208	75,232,321,981	584,728	19,514.5	27.26	30,439	84.18	69.18	18.42	20.000
C11	3,749,668	75,507,014,613	215,132	20,137.0	28.50	27,720	87.15	73.12	18.15	19.535
C12	3,927,940	81,589,237,368	737,990	20,771.5	29.45	34,927	82.63	66.80	18.34	20.000
C13	10,839,502	75,051,614,400	107,524	6,923.9	28.18	11,230	82.12	65.86	16.35	17.000
C14	5,058,904	92,251,479,203	198,176	18,235.5	28.60	27,970	85.93	70.91	17.72	19.000
C15	3,522,049	80,912,615,895	291,845	22,973.2	29.30	35,038	81.63	66.08	17.46	19.000
C16	2,645,838	50,726,621,539	280,336	19,172.2	18.35	33,058	74.89	57.77	16.01	18.000
C17	3,054,146	29,144,016,270	116,810	9,542.4	10.93	17,309	84.13	68.60	17.04	18.000
C18	4,790,854	44,290,513,825	306,231	9,244.8	16.86	18,063	73.86	56.17	15.95	17.000
C19	3,623,933	78,417,582,081	466,186	21,638.8	28.49	34,885	86.75	73.28	18.55	20.000
C20	3,663,675	77,163,089,740	607,498	21,061.7	27.32	33,248	79.99	62.59	17.30	19.000
C21	2,575,410	53,705,600,160	372,912	20,853.2	19.36	35,252	83.86	68.11	18.04	20.000
C22	2,370,554	51,298,610,931	403,499	21,639.9	18.88	34,978	84.58	69.46	18.30	20.000
C23	3,262,926	75,206,706,356	394,666	23,048.9	27.21	34,899	85.16	70.51	17.86	20.000
C24	3,833,398	73,141,221,559	516,441	19,080.0	26.55	32,724	82.88	66.80	18.46	20.000
**Mean**	4,212,193	69,767,752,137	429,788	18,362.0	25.19	29,073	82.95	67.50	17.77	19.240
**Median**	3,833,398	75,421,594,079	372,912	20,137.0	27.29	32,074	82.63	66.80	18.04	19.535
Spleen Normal	13,291,753	73,498,824,861	143,742	5,529.7	27.32	9,975	82.80	68.58	16.51	17.000
Parental	3,720,422	85,913,740,338	926,409	23,092.5	31.05	35,411	83.92	68.75	18.53	20.000

Table S1: **Long-read ONT sequencing statistics for the 23 sublines, spleen, and parental B2905 line.** Q20(%) (and Q30(%)) represents the percentage of bases that have a PHRED quality score of 20 (and 30) or over. Avg Qual and Med Qual are the mean and median PHRED quality scores for all passing reads, respectively. Means of %, average and median statistics are recalculated with total base count. Mean and median of N50 are calculated using the final N50 output per subline.

Subline	# Reads	Total Length	Max Length	Mean Length	Avg Depth	Q20(%)	Q30(%)	AvgQual
C1	214,587,122	26,514,866,642	151	123.6	151.87	99.39	98.08	32.47
C2	177,351,022	21,885,891,023	151	123.4	127.55	99.36	98.01	32.30
C3	236,326,610	29,241,058,731	151	123.7	160.17	99.41	98.13	32.58
C4	175,342,036	21,589,058,024	151	123.1	129.77	99.38	98.05	32.42
C5	205,967,396	25,398,503,020	151	123.3	143.05	99.37	98.02	32.35
C6	214,023,384	26,646,667,373	151	124.5	143.36	99.40	98.11	32.50
C7	223,313,600	27,574,075,712	151	123.5	157.99	99.40	98.07	32.49
C8	208,777,688	25,667,257,080	151	122.9	149.20	99.38	98.08	32.39
C9	235,523,830	28,811,943,724	151	122.3	169.79	99.34	97.99	32.19
C10	216,816,122	26,730,779,028	151	123.3	149.54	99.40	98.14	32.53
C11	198,685,460	23,999,015,045	151	120.8	141.63	99.31	97.89	32.03
C12	190,688,314	23,302,161,699	151	122.2	133.15	99.33	97.97	32.14
C13	237,138,312	29,056,964,816	151	122.5	155.32	99.39	98.11	32.47
C14	172,757,040	21,201,029,234	151	122.7	125.52	99.36	98.05	32.30
C15	220,232,000	27,124,320,850	151	123.2	149.10	99.39	98.14	32.34
C16	185,659,520	22,843,064,280	151	123.0	131.73	99.35	98.04	32.15
C17	192,082,428	23,612,072,157	151	122.9	139.55	99.35	98.02	32.11
C18	217,033,816	26,777,924,287	151	123.4	153.69	99.38	98.12	32.29
C19	206,930,234	25,388,741,657	151	122.7	141.87	99.35	98.02	32.10
C20	203,123,840	25,125,725,020	151	123.7	148.59	99.39	98.12	32.32
C21	251,065,838	30,831,059,682	151	122.8	177.12	99.29	97.93	31.86
C22	194,541,570	24,051,444,930	151	123.6	139.71	99.39	98.14	32.38
C23	186,776,028	22,557,078,917	151	120.8	140.54	99.28	97.86	31.85
C24	182,117,204	22,497,739,309	151	123.5	132.99	99.34	98.02	32.14
**Mean**	206,119,184	25,351,185,093	151	123.0	145.53	99.36	98.05	32.28
**Median**	206,448,815	25,393,622,339	151	123.3	143.21	99.38	98.05	32.30

Table S2: **WES sequencing statistics for the 23 sublines, spleen, and parental B2905 line.** Q20(%) (and Q30(%)) represents the percentage of bases that have a PHRED quality score of 20 (and 30) or over. Avg Qual and Med Qual are the mean and median PHRED quality scores for all passing reads, respectively. Means of %, average and median statistics are recalculated with total base count. Mean and median of N50 are calculated using the final N50 output per subline.

Feature \ Clade	Green	Orange	Blue	Red
Growth rate in C57BL/6 mice	++	+	+/-	+++
Response to host immunity (Delay of growth initiation in C57BL/6)	High	Medium	Very high	Low
ICB response (anti-CTLA4 vs. IgG)	Medium	High	(growing too slow for study)	Low
Cell cycle gene expression	Medium	High	Medium	Low
Wnt signaling gene expression	Mixed	Canonical		Non-canonical
**Phenotype**	**Mixed, Less Sensitive to ICB**	**Proliferative, Sensitive to ICB**	**Immunogenic, Susceptible to Host Immunity**	**Invasive, ICB Resistant**

Table S3: **Phenotypes of the 4 major clades.** The phenotype for each of the major clades was determined by features defined by the behavior or characteristics of (select) sublines from the clade previously characterized (Methods) [8].

Subline	Clonal (%)	Subclonal (%)	Private (%)	Regenotyped	Total Placed	Total Unplaced
C1	50.92	6.91	42.17	3	66,172	6,090
C3	63.47	13.55	22.98	3	52,661	7,613
C4	57.02	7.89	35.09	8	53,905	5,894
C5	65.56	0.57	33.86	2	51,199	6,767
C6	76.19	7.59	16.22	0	44,258	6,665
C7	67.21	19.62	13.17	2	49,692	5,475
C8	64.48	14.16	21.36	57	51,922	4,742
C9	59.46	1.97	38.56	0	55,089	4,759
C10	65.43	3.69	30.88	1566	50,932	6,361
C11	59.27	5.37	35.36	2	57,140	4,939
C12	66.47	5.05	28.48	19	50,743	6,815
C13	62.08	2.52	35.41	11	51,401	4,088
C14	64.26	14.15	21.59	3	50,411	6,526
C15	65.06	22.53	12.41	4	50,482	6,949
C16	53.68	7.60	38.72	2	58,846	5,008
C17	53.49	1.15	45.37	7	55,490	3,771
C18	62.74	22.53	14.74	1	49,929	5,211
C19	51.04	1.06	47.90	50	57,948	6,723
C20	66.18	19.31	14.51	11	50,030	5,797
C21	69.88	6.90	23.22	1	47,280	6,764
C22	52.74	7.26	39.99	9	62,225	6,969
C23	59.29	1.31	39.40	0	56,589	6,572
C24	75.39	7.21	17.40	2	44,618	6,702
**Mean**	62.23	8.69	29.08	76	52,998	5,965
**Median**	63.47	7.21	30.88	3	51,401	6,361

Table S4: **`TreeHarmonizer` SNV placement statistics relative to each subline.** Clonal, subclonal and private represent the percentages of placed SNVs that were called in the respective subline. Regenotyped SNVs are those who were assigned the "lost variant" genotype by `TreeHarmonizer`. Total placed and unplaced counts are respective to each subline. Placed and unplaced counts contain duplicates (representing the same variant) between sublines.

**Subline**	**Clonal (%)**	**Subclonal (%)**	**Private (%)**	**Total Placed**	**Total Unplaced**
C1	77.69	3.85	18.46	130	33
C3	73.57	7.86	18.57	140	40
C4	82.40	4.00	13.60	125	29
C5	80.47	0.00	19.53	128	39
C6	67.55	1.32	31.13	151	41
C7	76.12	13.43	10.45	134	31
C8	61.08	9.58	29.34	167	32
C9	67.39	0.00	32.61	138	14
C10	66.88	1.95	31.17	154	40
C11	49.05	3.81	47.14	210	35
C12	54.01	1.60	44.39	187	44
C13	39.64	0.90	59.46	222	12
C14	69.50	7.80	22.70	141	35
C15	64.56	20.89	14.56	158	40
C16	49.75	6.90	43.35	203	37
C17	76.23	1.64	22.13	122	17
C18	65.75	23.29	10.96	146	16
C19	34.77	0.72	64.52	279	43
C20	64.15	11.32	24.53	159	43
C21	88.70	1.74	9.57	115	36
C22	69.13	3.36	27.52	149	36
C23	43.97	0.86	55.17	232	48
C24	75.57	1.53	22.9	131	33
**Mean**	65.13	5.58	29.29	161.78	33
**Median**	67.39	3.36	24.53	149	36

Table S5: `TreeHarmonizer` **SV placement statistics respective to each subline.** Clonal, subclonal and private represent the percentages of placed SVs that were called in the respective subline. Total placed and unplaced counts are respective to each subline. Placed and unplaced counts contain duplicates (representing the same variant) between sublines.

Chr	Start	End	Direction	Putative Promoter	EPDnew	Proximal Enhancer
chr2	156447300	156448010	hyper	*Gm14225*	-	*Epb41l1*, *Gm14225*
chr6	30736515	30739964	hyper	*Mest*	*Mest*	*Mest*, *Mir335*, *Gm27522*, *Gm27568*, *Gm44296*
chr6	43264995	43267174	hyper	*Arhgef5*, *Gm44731*	*Arhgef5*	*Arhgef5*, *Gm44731*
chr6	52203136	52204841	hyper	*Hoxa5*	-	*Hoxa5*, *Hoxa6*, *Hoxaas3*, *Hoxa3*, *Gm28308*
chr6	73220915	73223008	hyper	*Dnah6*, *Gm40377*	*Dnah6*	*Dnah6*, *Gm40377*
chr6	88188695	88190058	hyper	-	-	*Gm38708*
chr6	88193674	88194653	hyper	*Gata2*	-	*Gata2*
chr6	116628225	116629385	hyper	*Zfp422*	*Zfp422*	*Zfp422*, *Gm44154*
chr7	23166848	23167923	hyper	*Gm8728*	-	-
chr10	83140358	83141188	hyper	-	-	*Chst11*, *1700025N21Rik*
chr11	28582204	28583082	hyper	-	-	*Ccdc85a*
chr3	79285435	79286044	hypo	-	-	*Rapgef2*, *6430573P05Rik*
chr3	146220813	146221659	hypo	*Lpar3*	*Lpar3*	*Lpar3*
chr4	130171758	130174329	hypo	-	-	*Tinagl1*
chr4	155774166	155775696	hypo	*Vwa1*	*Vwa1*	*Vwa1*
chr5	14025106	14026982	hypo	*Sema3e*	*Sema3e*	*Sema3e*, *Gm43519*
chr6	14897121	14900597	hypo	-	-	*Foxp2*
chr6	117168413	117169671	hypo	*Cxcl12*	*Cxcl12*	*Cxcl12*
chr8	34524973	34526019	hypo	-	-	*Gm33968*
chr9	54300511	54301797	hypo	*Gldnos*	-	-
chr11	65160117	65161795	hypo	*1700086D15Rik*	-	*Arhgap44*, *1700086D15Rik*
chr11	96279456	96280673	hypo	-	-	*Hoxb8*
chr12	82586939	82588254	hypo	*Rgs6*	-	-
chr15	27467528	27468632	hypo	-	-	*Ank*
chr17	31299560	31300475	hypo	*Slc37a1*	-	-
chrX	52613765	52614700	hypo	*Gpc3*	*Gpc3*	*Gpc3*, *A630012P03Rik*

Table S6: **DMRs associated with the red clade that overlap putative promoter or putative proximal enhancers of known genes.** A region is said to be a putative promoter for a gene if it is within -350bp to +50bp of the gene's annotated transcriptional start site (Ensembl Release 102) [14, 6]. Of the 17 putative promoters, 9 are also annotated in the EPDnew (v.003) database of experimentally validated promoters [3, 16]. A region is said to be a putative proximal enhancer for a gene if is has enhancer-like signature [1] and is within 2Kbp of the annotated transcriptional start site [5, 13, 10] of the gene. Note that *Gata2* was an outlier in that its putative promoter was noted to be hypermethylated while its expression has a positive log2 fold change (Figure 5d.); however, this may be due to the hypomethylation of a putative promoter of *Gata2* alternative isoforms, which went undetected as its mean methylation difference is not large enough to meet our criteria (Methods).

Subline	Weighted Ploidy
C1	2.41
C3	2.37
C4	2.33
C5	2.21
C6	2.25
C7	2.17
C8	2.26
C9	2.29
C10	2.21
C11	2.19
C12	2.08
C13	2.28
C14	2.25
C15	2.38
C16	2.20
C17	2.21
C18	2.38
C19	1.95
C20	2.25
C21	2.24
C22	2.38
C23	2.24
C24	2.26
**Mean**	2.25
**Median**	2.25

Table S7: **Weighted mean ploidy per subline.** Mean of weighted ploidies is also weighted. Median is calculated over subline averages.

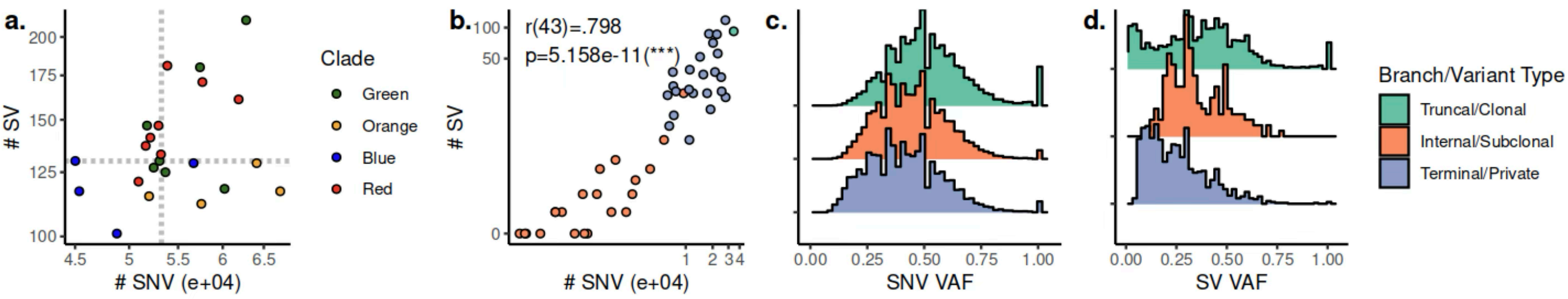

Figure S1: **SNVs and SVs placed by `TreeHarmonizer`. a.** Cumulative number of placed SNVs and SVs per subline, colored by clade. Medians are marked by dashed lines. The correlation between cumulative number of SNVs and SVs was statistically insignificant (rs(21)=.239, p=.2713) **b.** The number of SNVs and the number of SVs placed at each branch of the phylogeny strongly correlate with each other. **c.** Variant allele frequency distributions of placed SNVs. **d.** Variant allele frequency distributions of placed SVs.

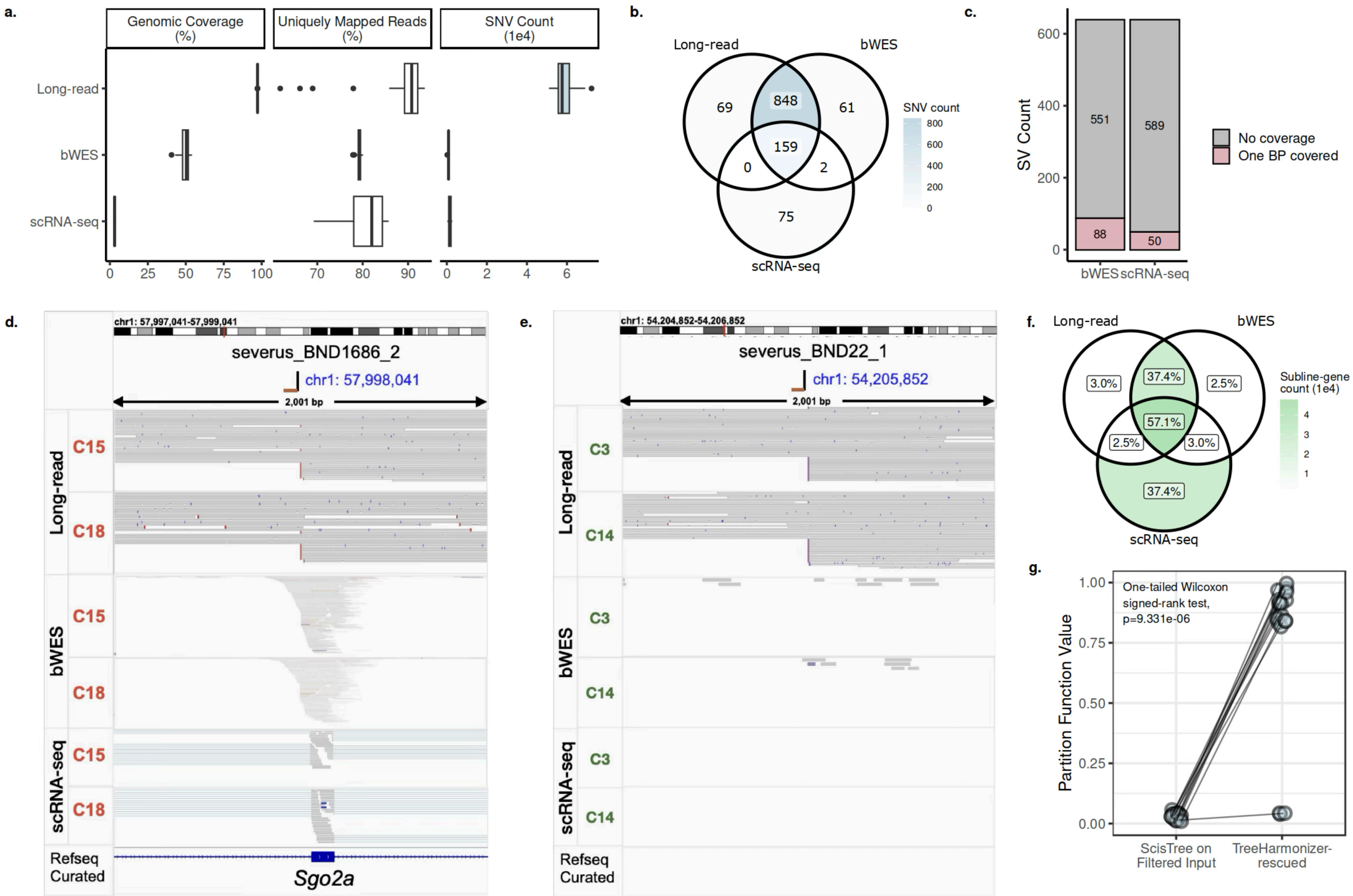

Figure S2: **Comparison among long-read, bWES, and scRNA-seq data of the 23 sublines.** **a.** Per-subline autosomal genomic coverage (%), uniquely mapped reads (%), and count of unique somatic SNVs profiled (Methods). **b.** Counts of unique, non-private somatic SNVs called within bWES target regions across the 23 sublines for each technology. **c.** Count of `Severus`-profiled SVs whose breakpoints have sufficient read coverage (200x after aggregating all reads from all sublines/cells) in bWES and scRNA-seq data. **d.** Example of genomic coverage at a near-exonic breakpoint associated with a subclonal SV across the 3 technologies. **e.** Example of genomic coverage at an intergenic breakpoint associated with a subclonal SV across the 3 technologies. **f.** Agreement on gene copy-number status (neutral, gain, loss) called from data from the 3 technologies across all sublines. For any subline, only genes with copy number calls available in all 3 technologies are included. **g.** Improvement in terms of partition function values [15] among variants impacted by filtering during tree construction, following variant placement by `TreeHarmonizer`.

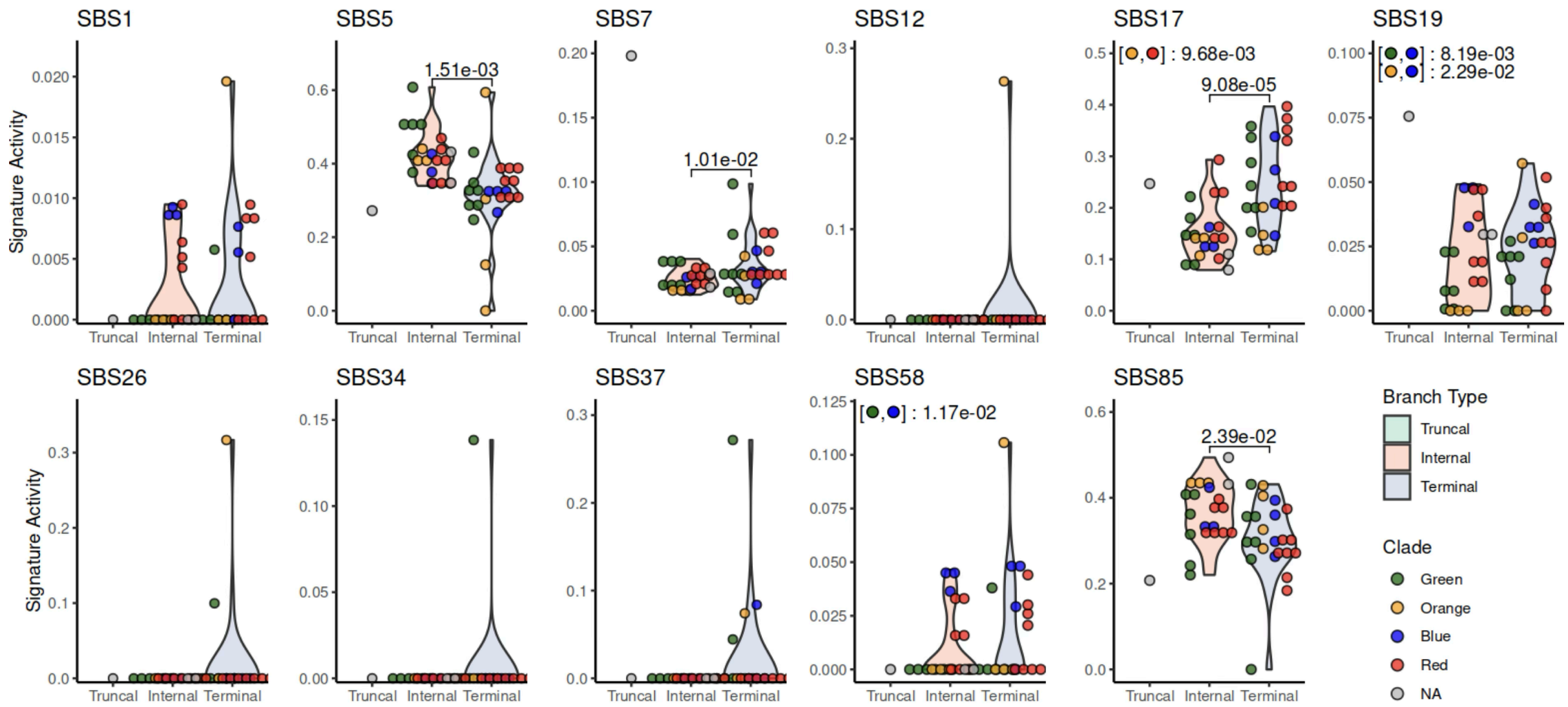

Figure S3: **Mutational signature activities along the tumor phylogeny.** In each panel, a point represents a branch in the phylogeny. A branch can either be categorized into truncal, internal or terminal, or by the color of the clade it is in. ANOVA with post-hoc Tukey HSD (Honestly Significant Difference) Test was performed, and the adjusted p-values of statistically significant pairs are reported.

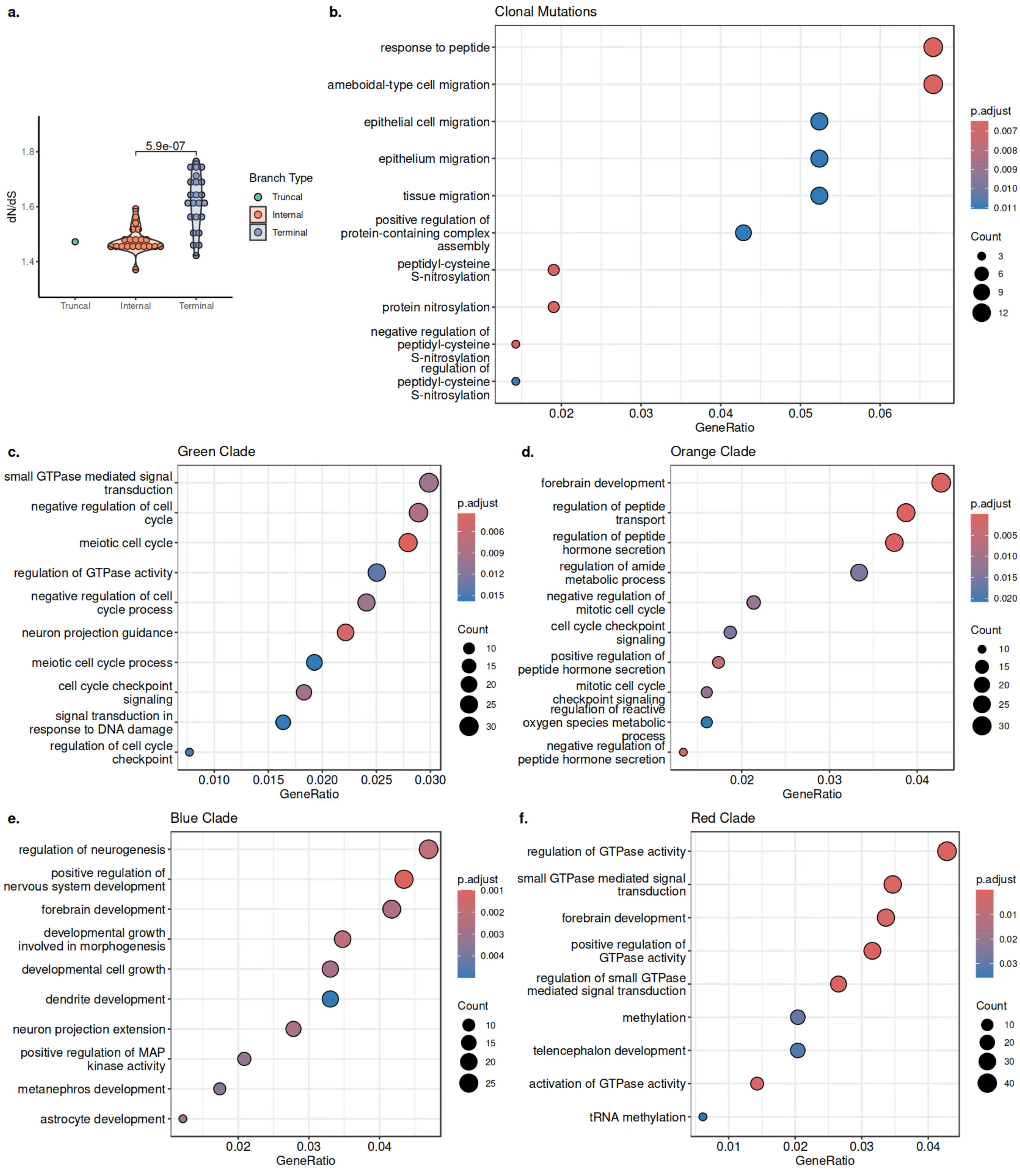

Figure S4: **SNVs along the tumor phylogeny. a.** dN/dS value distributions among truncal, internal, and terminal branches. dN/dS values at internal branches are significantly lower than that at terminal branches by one-tailed t-test. **b.** Select enrichment of genes harboring non-synonymous clonal mutations among GO Biological Process term. **c.-f.** Select enrichment of genes uniquely harboring non-synonymous mutations in the green (c.), orange (d.), blue (e.), and red (f.) clade among GO Biological Process terms.

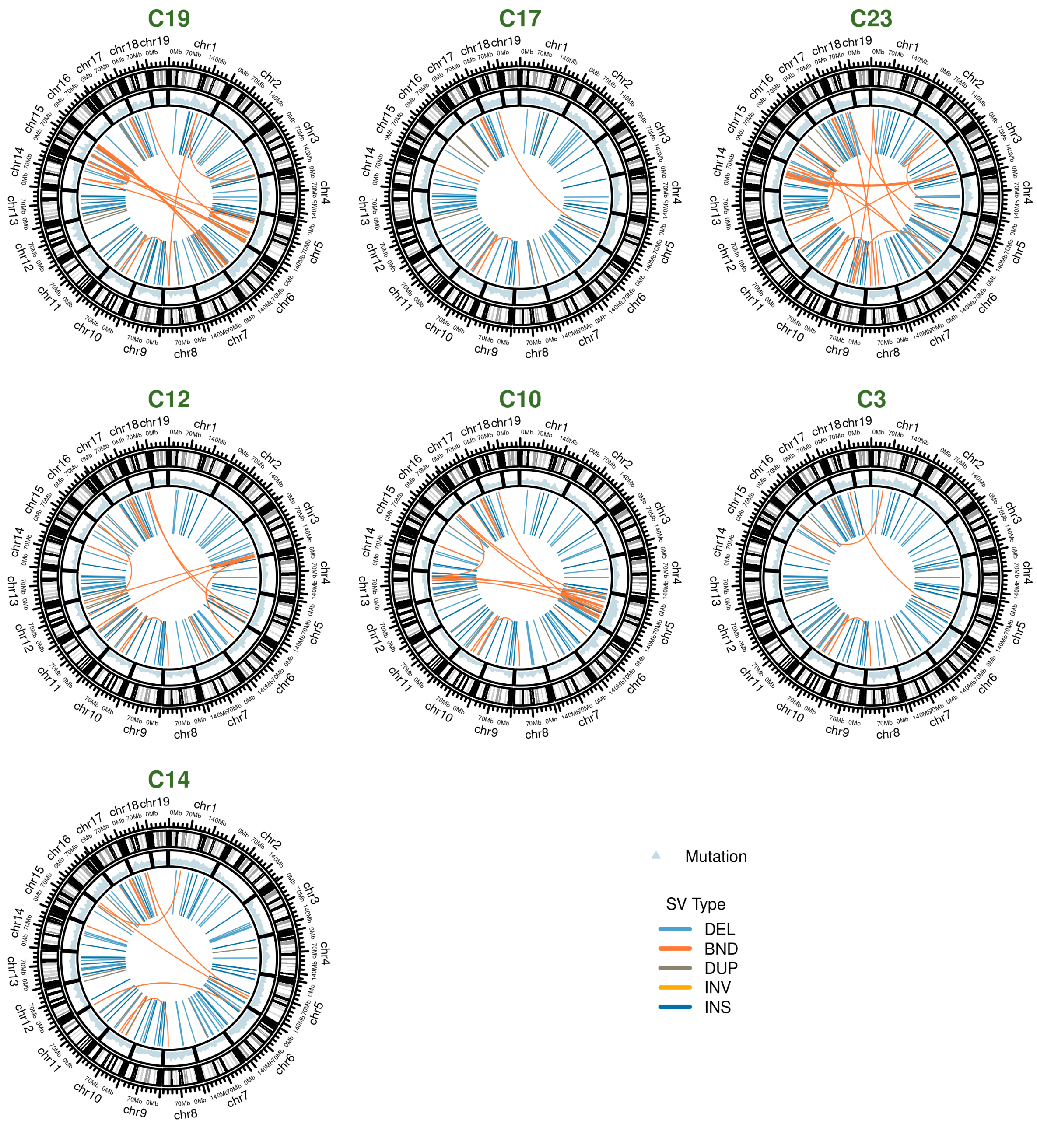

Figure S5: **SNV density and SV events present in individual sublines in the green clade.**

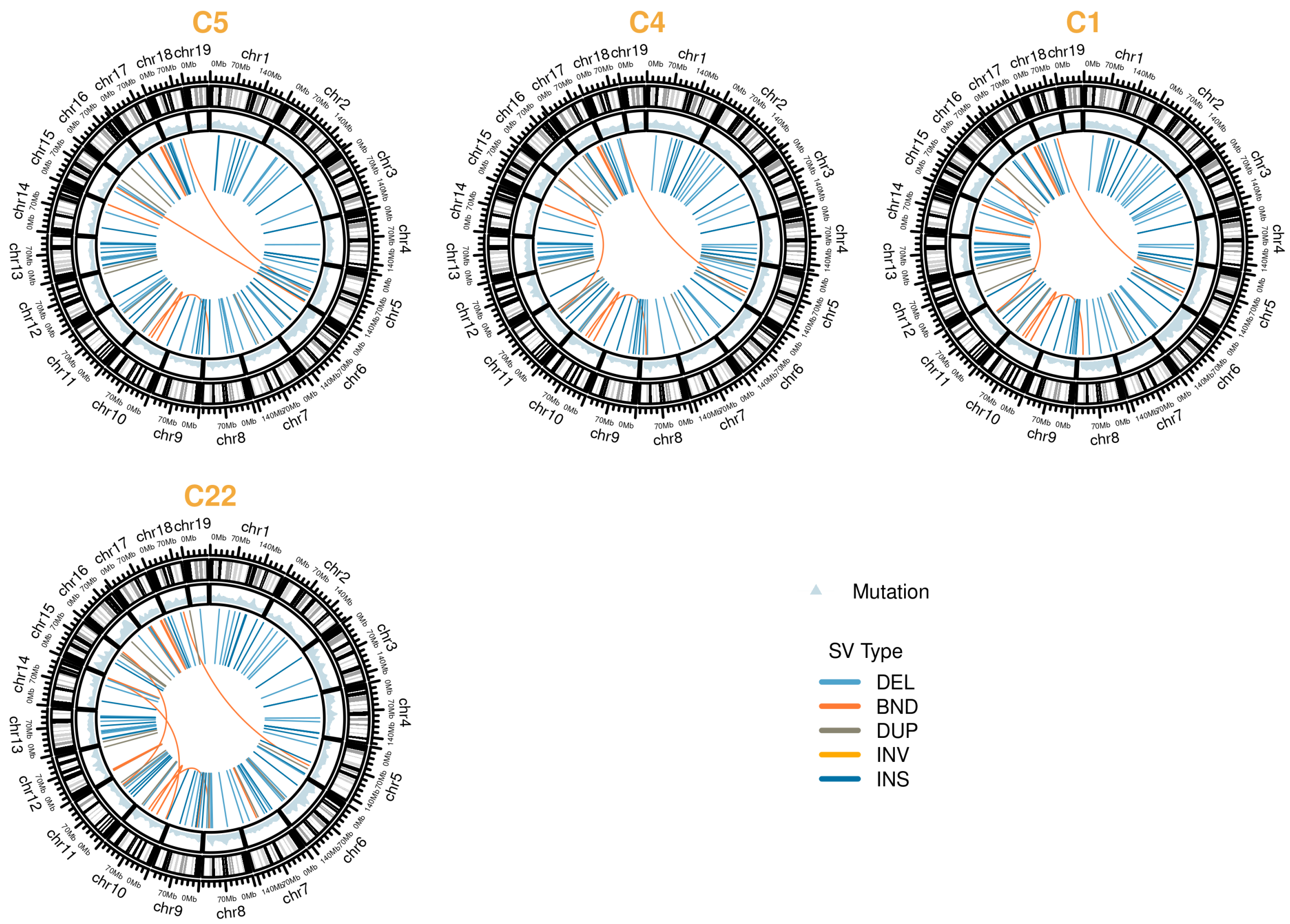

Figure S6: **SNV density and SV events present in individual sublines in the orange clade.**.

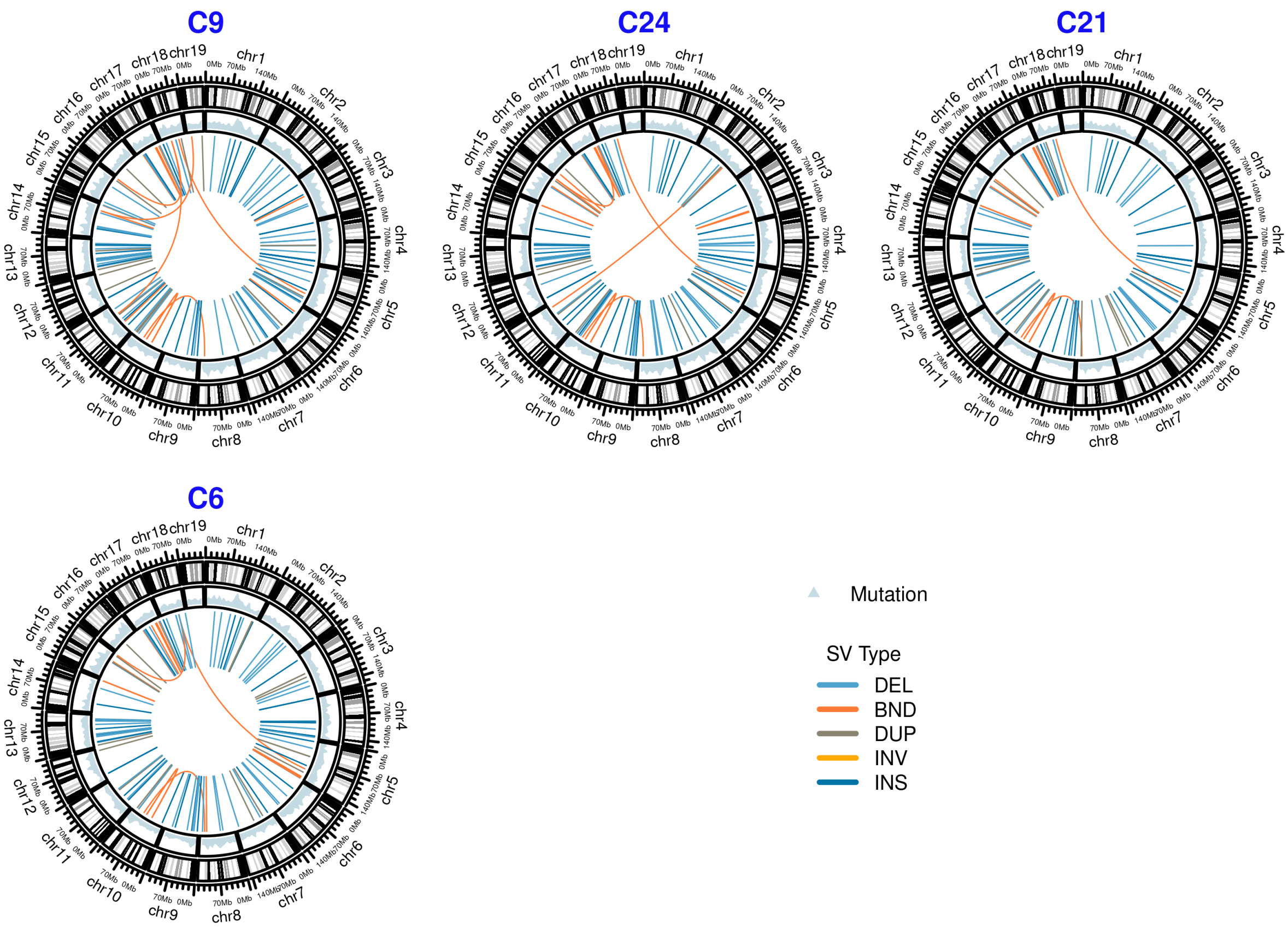

Figure S7: **SNV density and SV events present in individual sublines in the blue clade.**

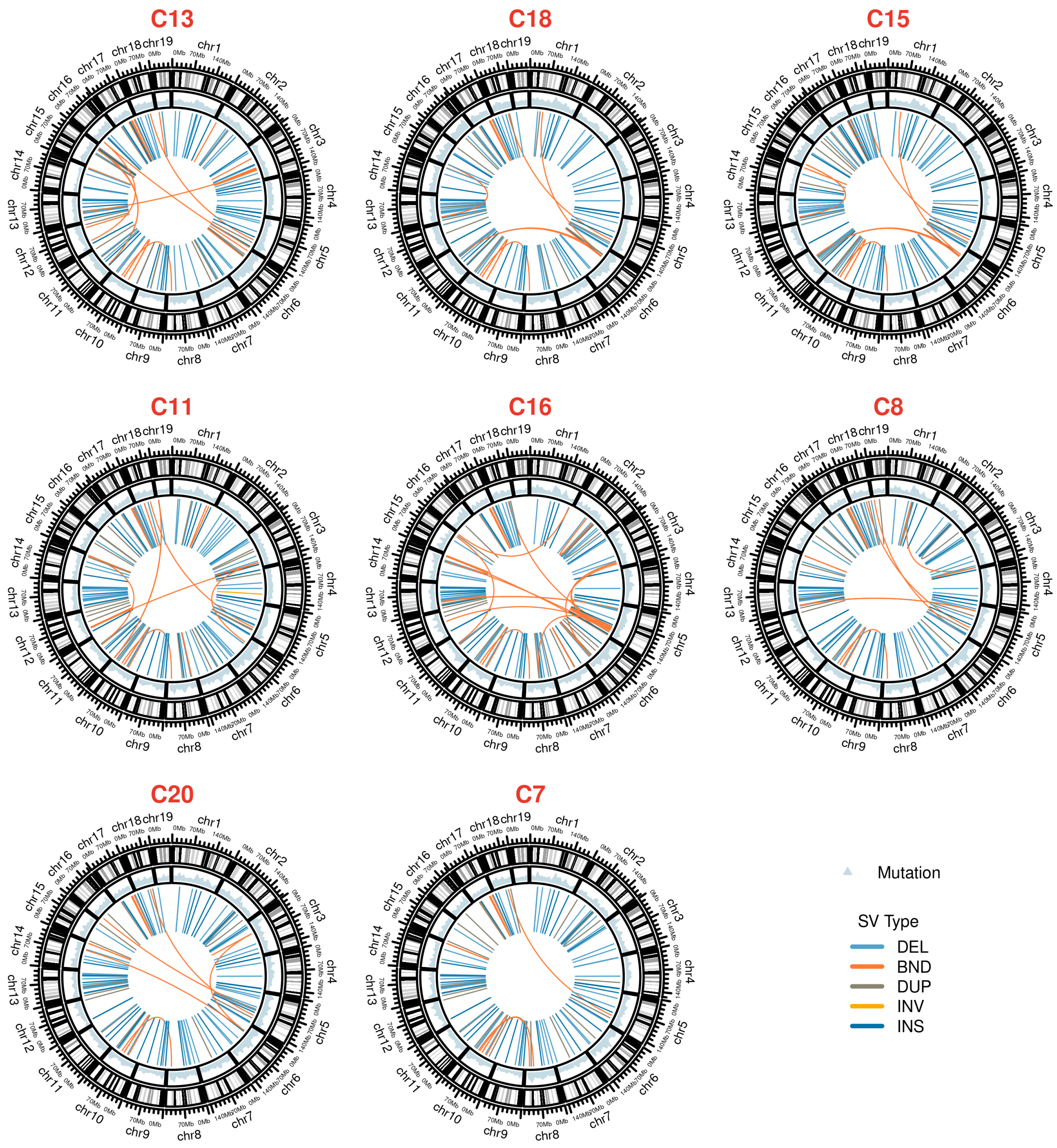

Figure S8: **SNV density and SV events present in individual sublines in the red clade.**

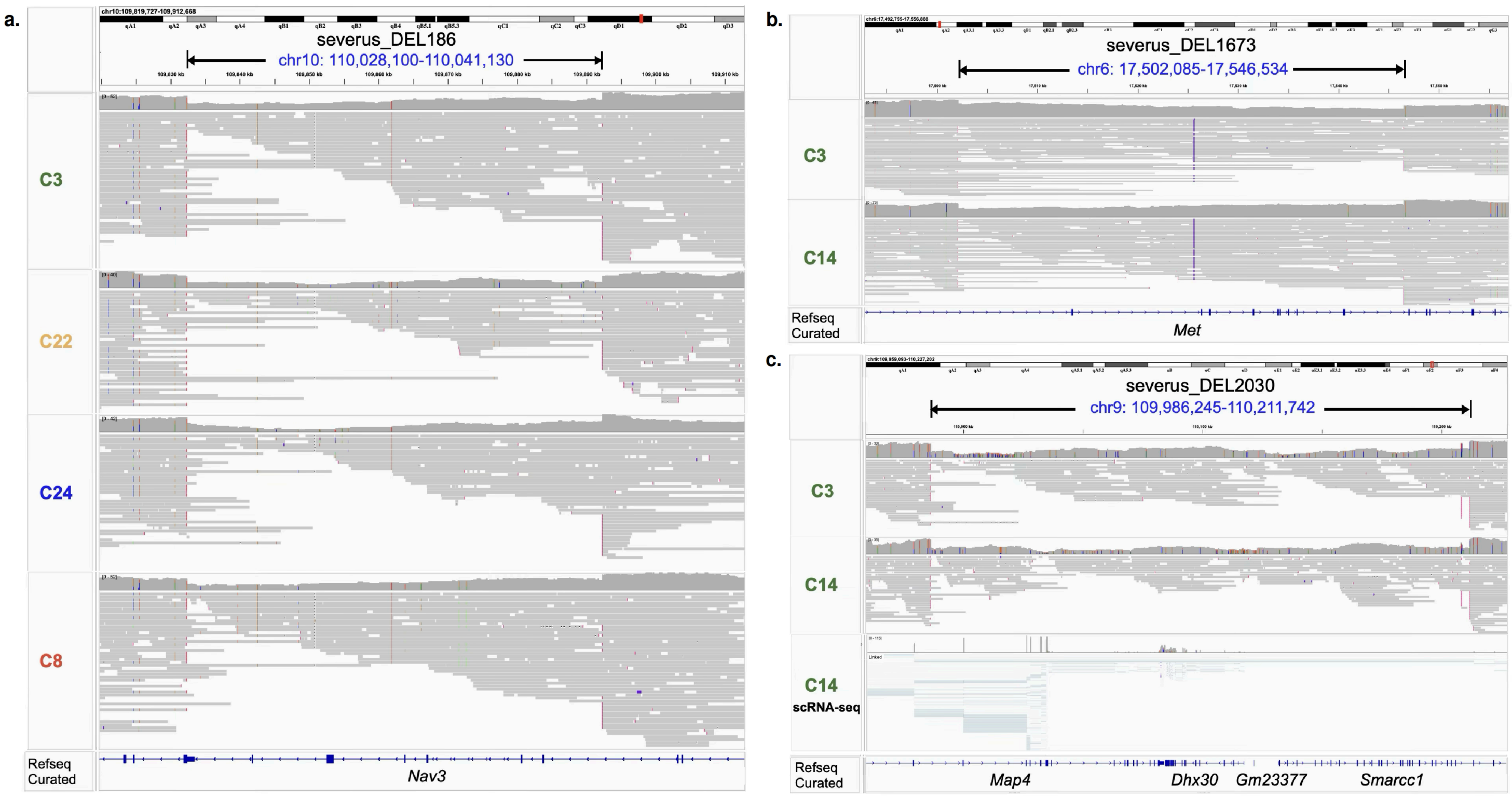

Figure S9: **SV-disrupted genes and gene fusion annotated by `Padfoot`. a.** A clonal deletion affects exon 4-9 of *Nav3*. Long-read genomic reads of an representative subline from each major clade are shown. **b.** A subclonal deletion in C3 and C14 in *Met* resulted in the loss of exon 4-11. Long-read genomic reads from sublines C3 and C14 are shown. **c.** A subclonal deletion in C3 and C14 resulted in the fusion of *Map4* and *Smarcc1*. Long-read genomic reads from sublines C3 and C14, as well as scRNA-seq reads from a C14 cell corroborating the fusion event are shown.

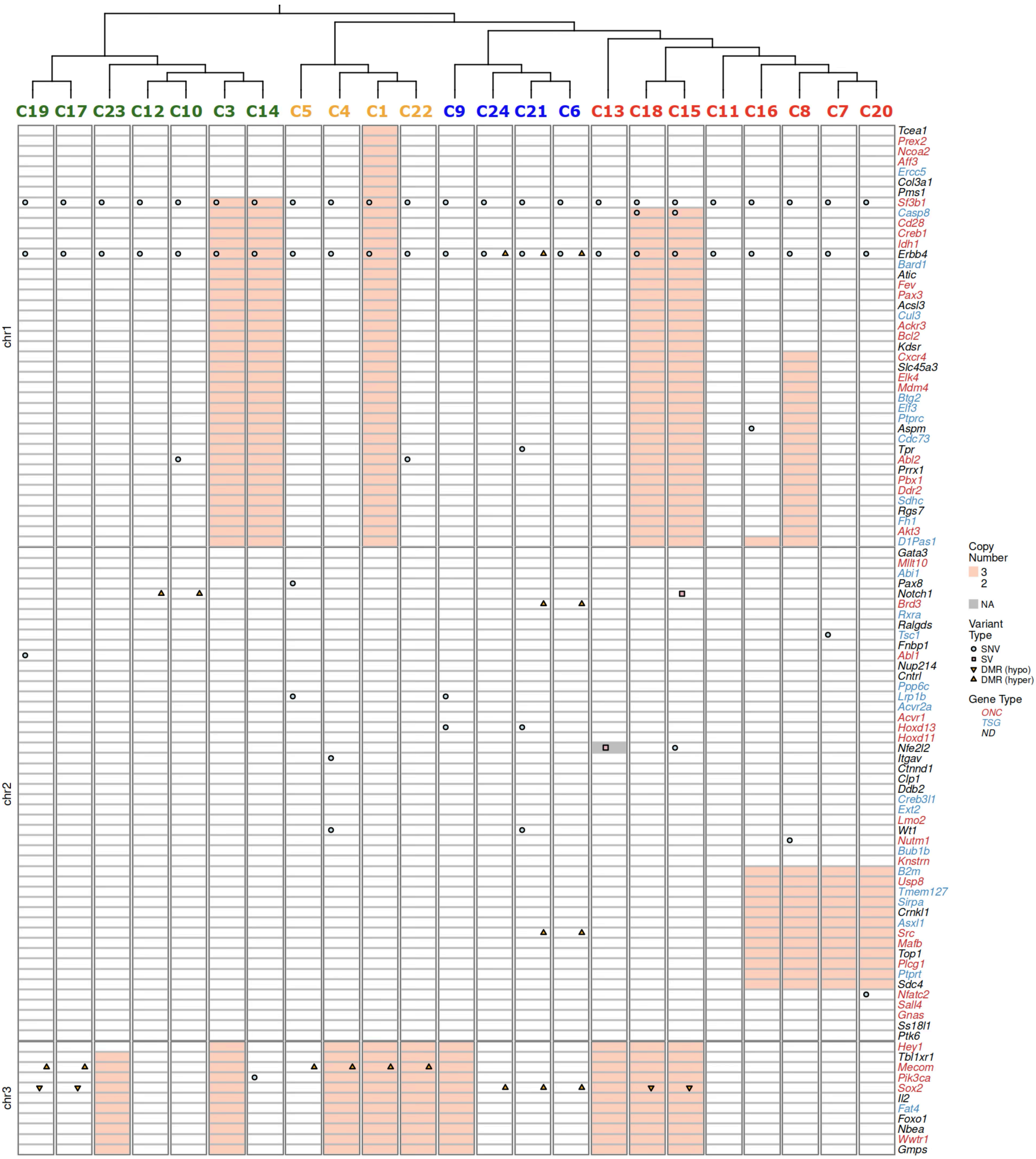

Figure S10: **Mouse orthologs of COSMIC genes impacted by (1) non-synonymous SNV, (2) SV breakpoint, (3) CNA, or (4) (hypo/hyper-) DMRs in their (putative) enhancers or promoters in 23 sublines (1/7).** Genes with "NA" copy number in a subline span detected CNA boundaries and therefore cannot be assigned a unique copy number.

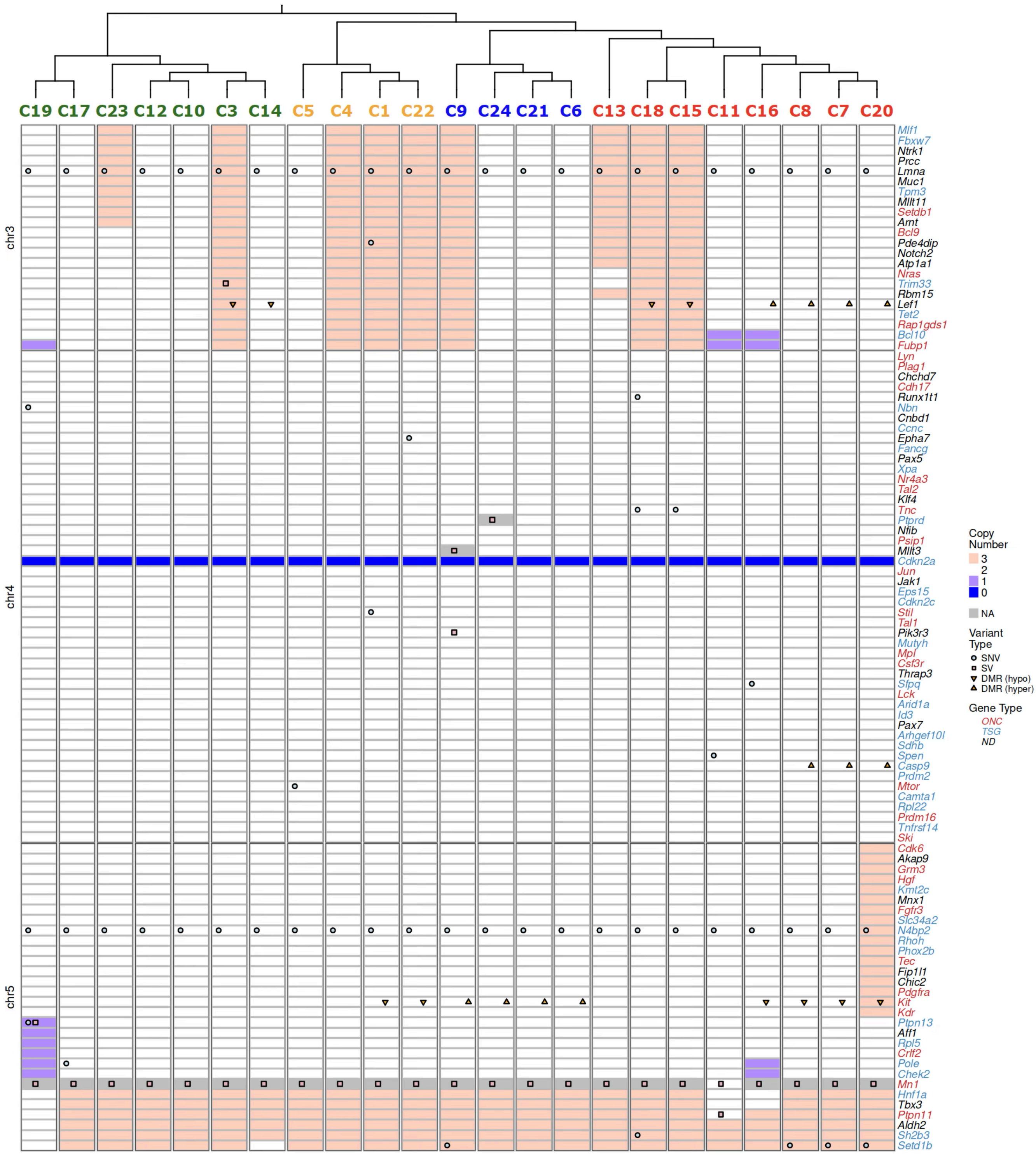

Figure S11: **Mouse orthologs of COSMIC genes impacted by (1) non-synonymous SNV, (2) SV breakpoint, (3) CNA, or (4) (hypo/hyper-) DMRs in their (putative) enhancers or promoters in 23 sublines (2/7).** Genes with "NA" copy number in a subline span detected CNA boundaries and therefore cannot be assigned a unique copy number.

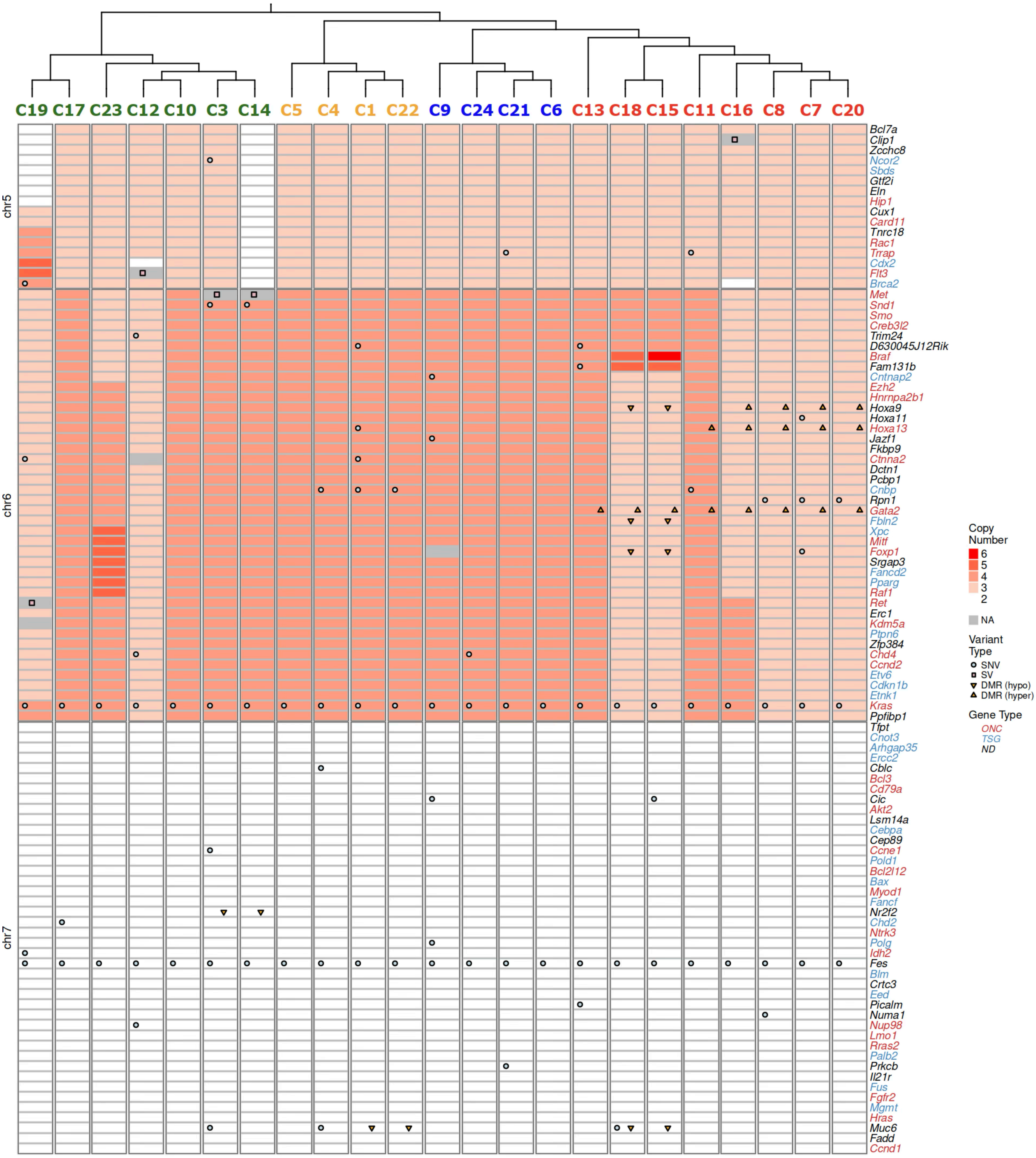

Figure S12: **Mouse orthologs of COSMIC genes impacted by (1) non-synonymous SNV, (2) SV breakpoint, (3) CNA, or (4) (hypo/hyper-) DMRs in their (putative) enhancers or promoters in 23 sublines (3/7).** Genes with "NA" copy number in a subline span detected CNA boundaries and therefore cannot be assigned a unique copy number.

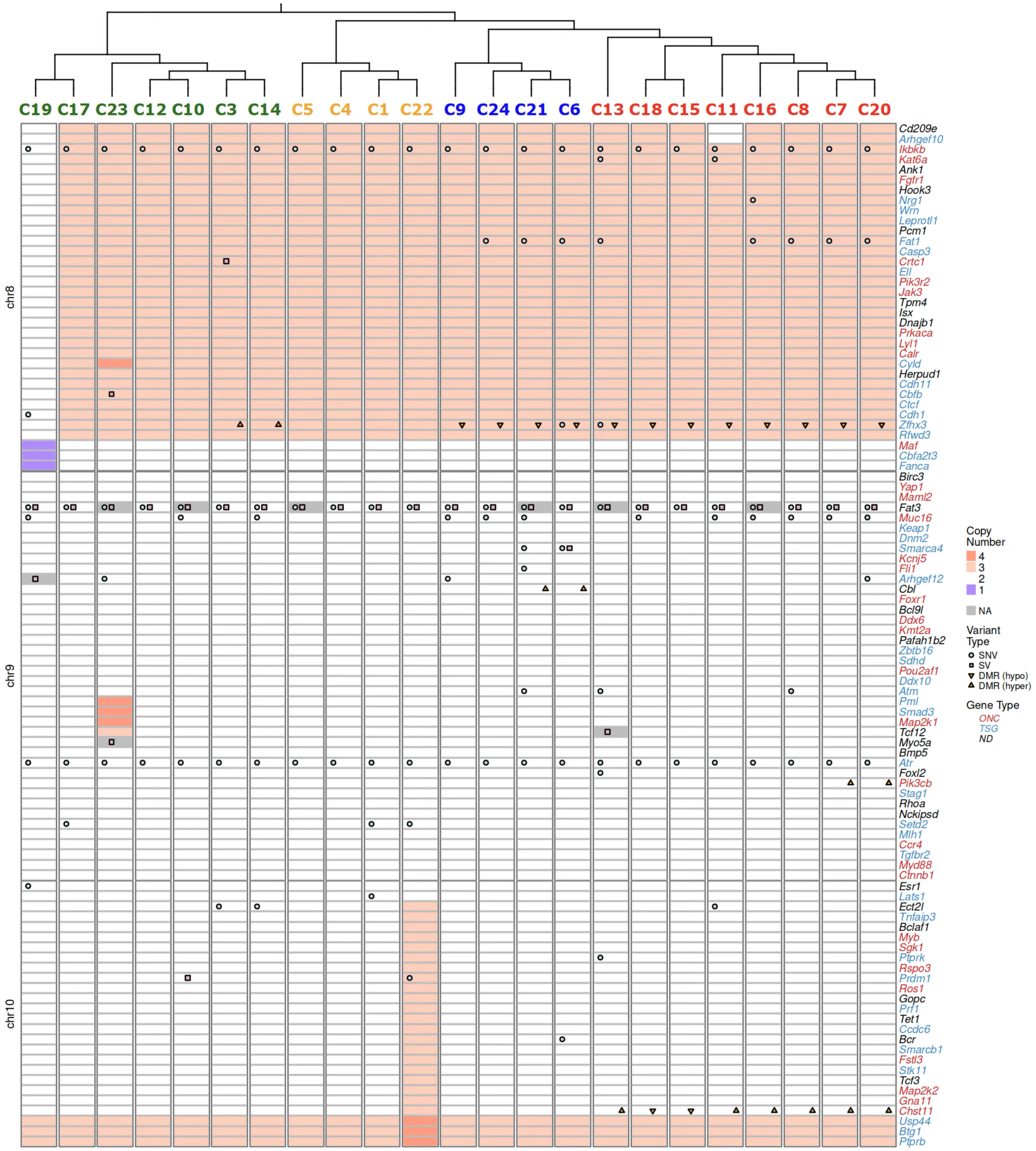

Figure S13: **Mouse orthologs of COSMIC genes impacted by (1) non-synonymous SNV, (2) SV breakpoint, (3) CNA, or (4) (hypo/hyper-) DMRs in their (putative) enhancers or promoters in 23 sublines (4/7).** Genes with "NA" copy number in a subline span detected CNA boundaries and therefore cannot be assigned a unique copy number.

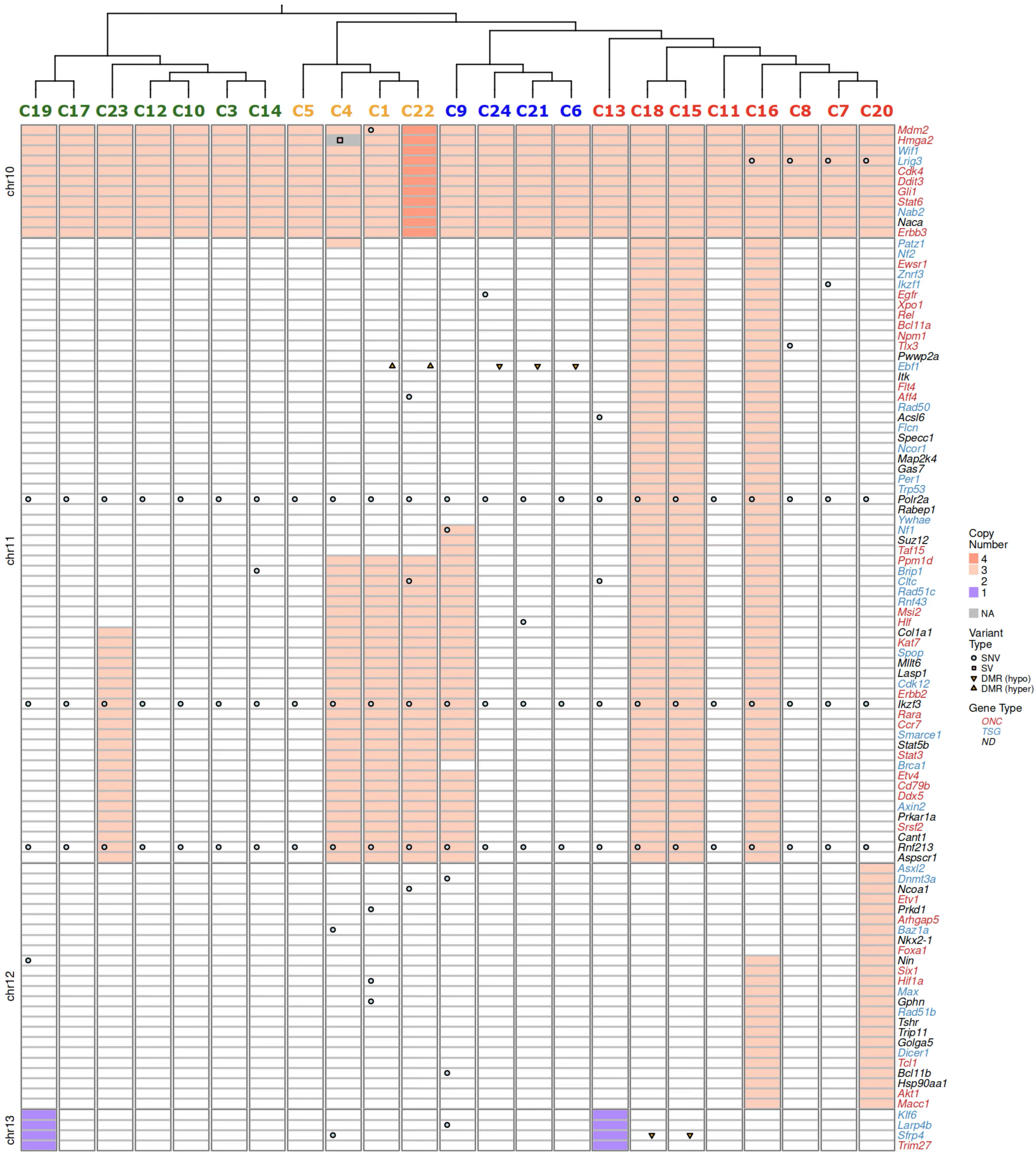

Figure S14: **Mouse orthologs of COSMIC genes impacted by (1) non-synonymous SNV, (2) SV breakpoint, (3) CNA, or (4) (hypo/hyper-) DMRs in their (putative) enhancers or promoters in 23 sublines (5/7).** Genes with "NA" copy number in a subline span detected CNA boundaries and therefore cannot be assigned a unique copy number.

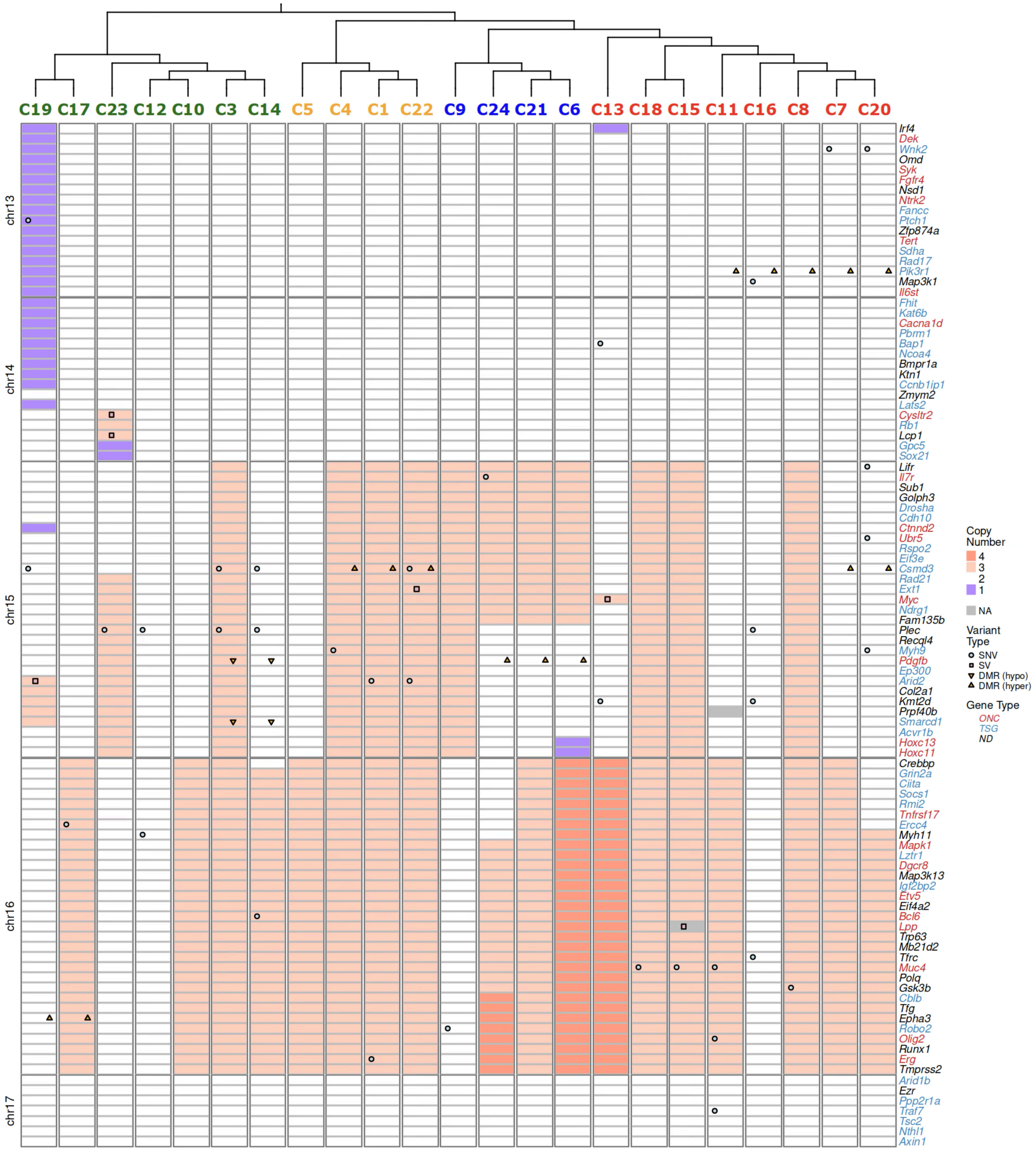

Figure S15: **Mouse orthologs of COSMIC genes impacted by (1) non-synonymous SNV, (2) SV breakpoint, (3) CNA, or (4) (hypo/hyper-) DMRs in their (putative) enhancers or promoters in 23 sublines (6/7).** Genes with "NA" copy number in a subline span detected CNA boundaries and therefore cannot be assigned a unique copy number.

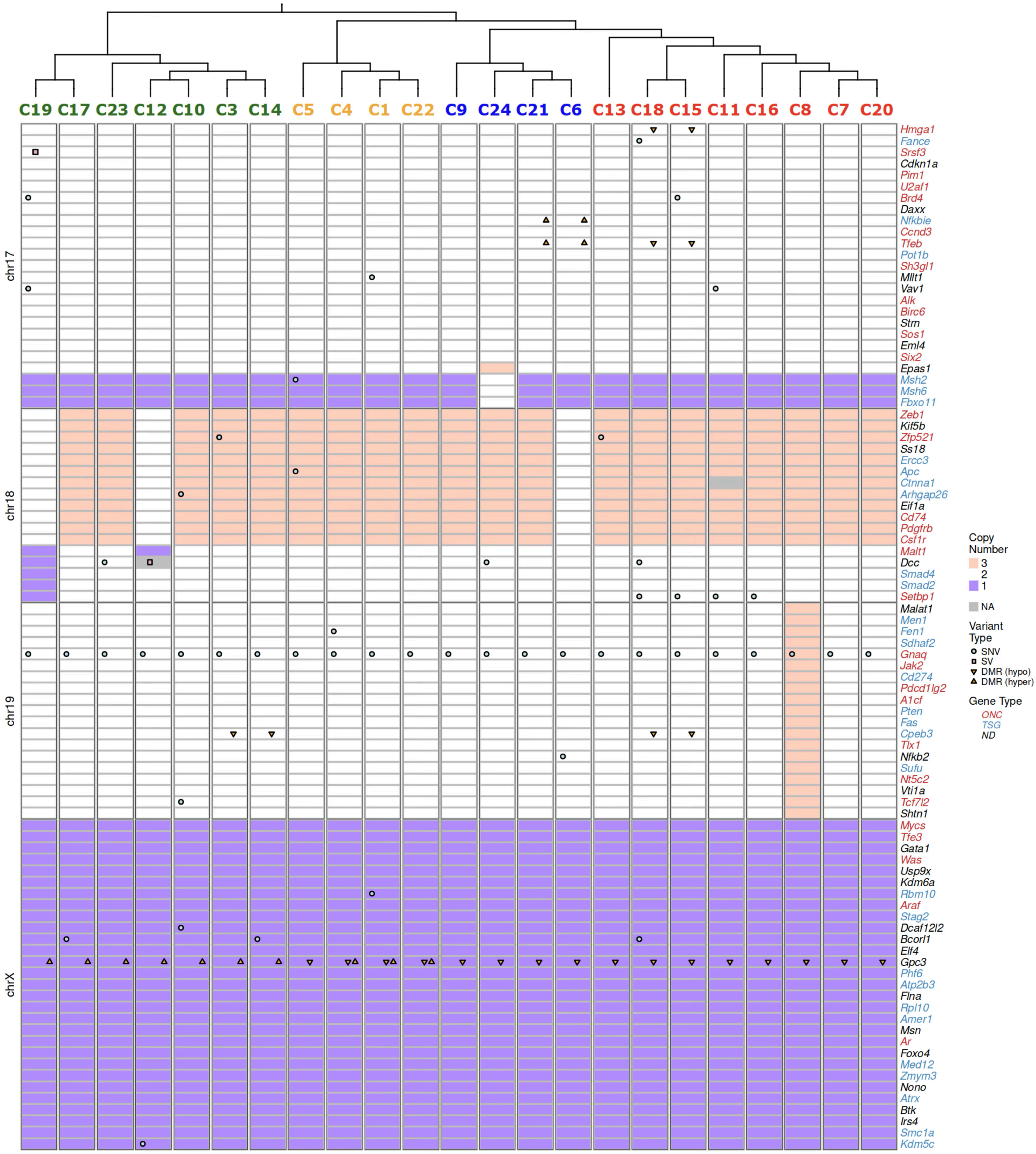

Figure S16: **Mouse orthologs of COSMIC genes impacted by (1) non-synonymous SNV, (2) SV breakpoint, (3) CNA, or (4) (hypo/hyper-) DMRs in their (putative) enhancers or promoters in 23 sublines (7/7).** Genes with "NA" copy number in a subline span detected CNA boundaries and therefore cannot be assigned a unique copy number.

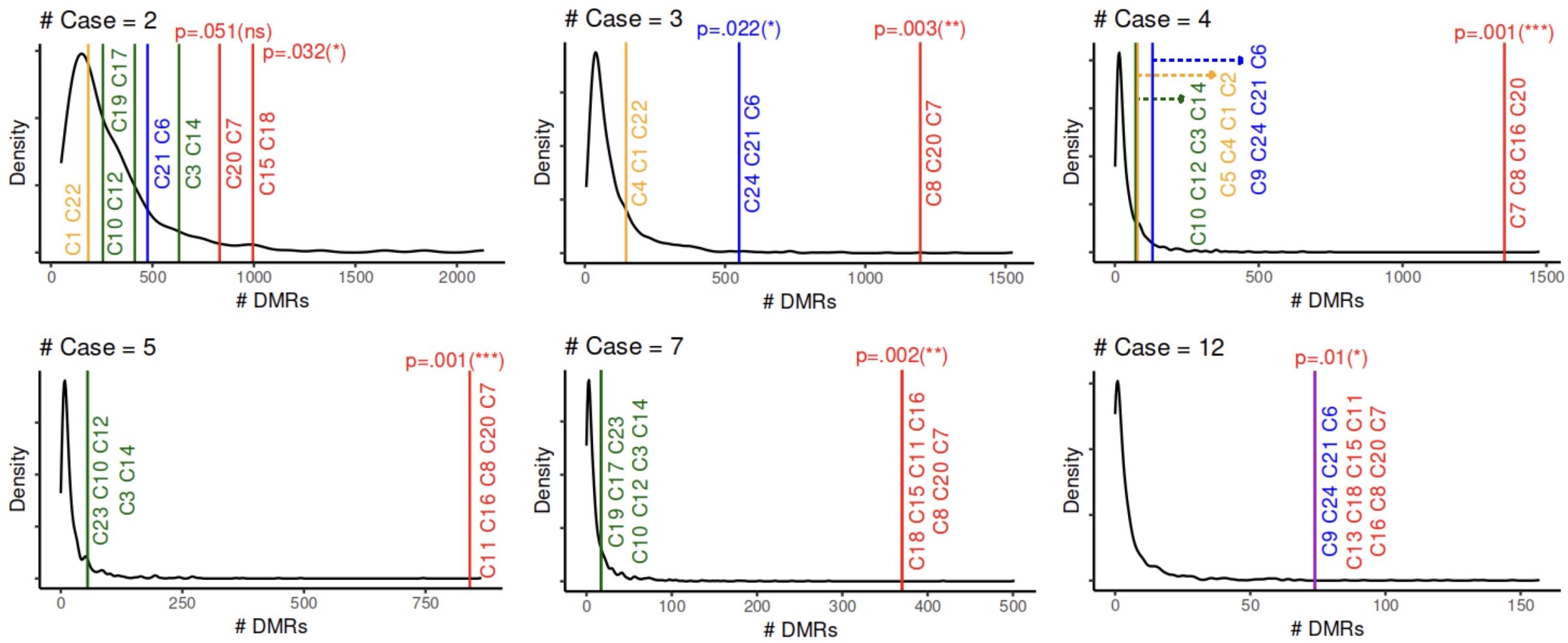

Figure S17: **Permutation tests on the number of DMRs obtained for all subtree sizes of the phylogeny in Figure 2a.** In each panel, "# Case" indicates the number of leaves in the subtree below the branch at which the test was performed. See Figure 5b. for "# Case = 8". Note that due to symmetry, no additional permutation test is done for "# Case = 16" to account for the subtree consisting of the orange, blue, and red in Figure 2a., as the empirical null distribution is identical to that of "# Case = 7" and the number of DMRs obtained is identical to that of the subtree of size 7 colored in green in Figure 2a.

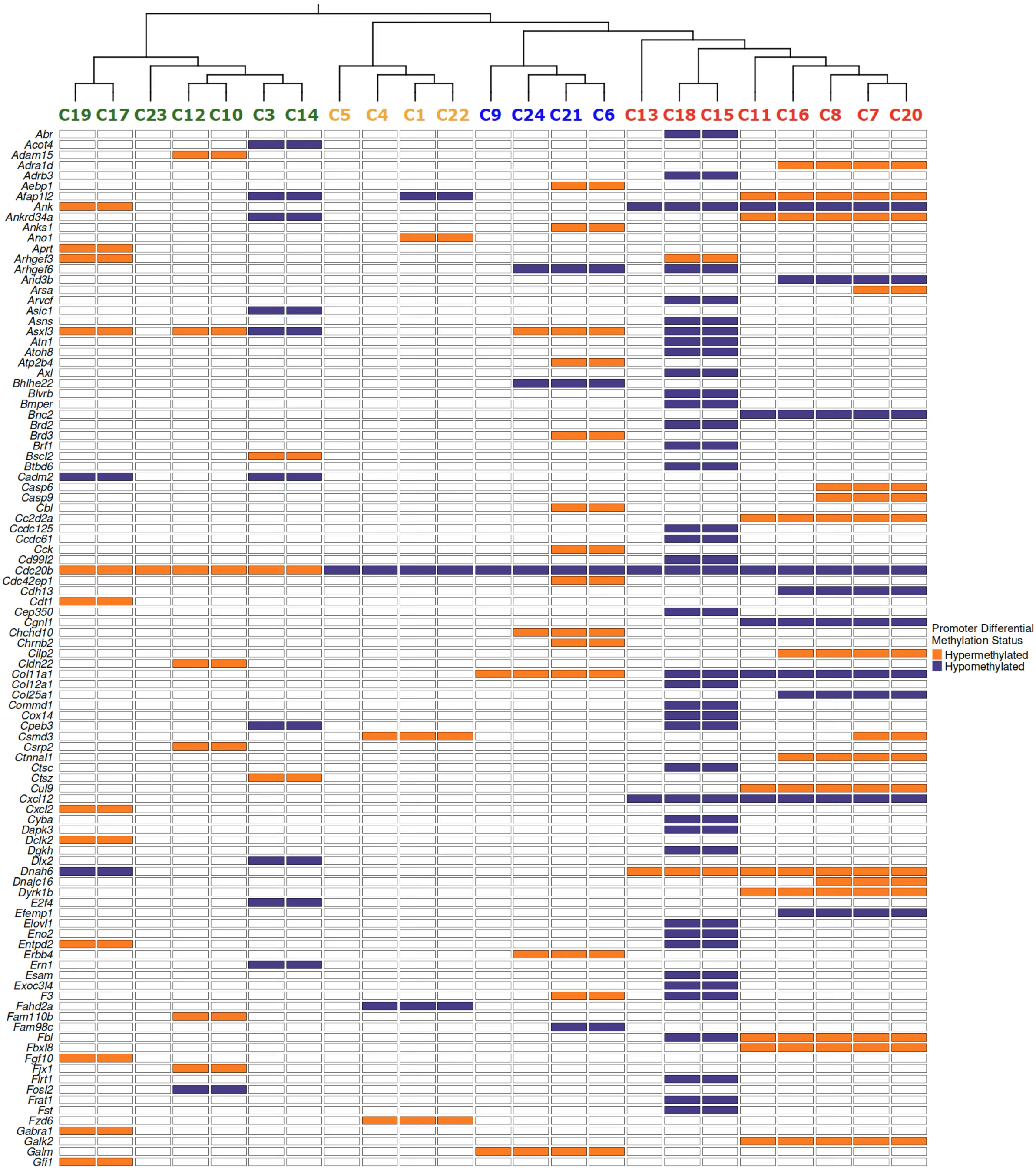

Figure S18: **Mouse orthologs of genes whose promoters are documented to be impacted by hypo- or hypermethylation in at least one of 17 types of human cancer according to OncoDB[27] and were found to be similarly impacted by promoter or enhancer differential methylation along the subline phylogeny (1/3).**

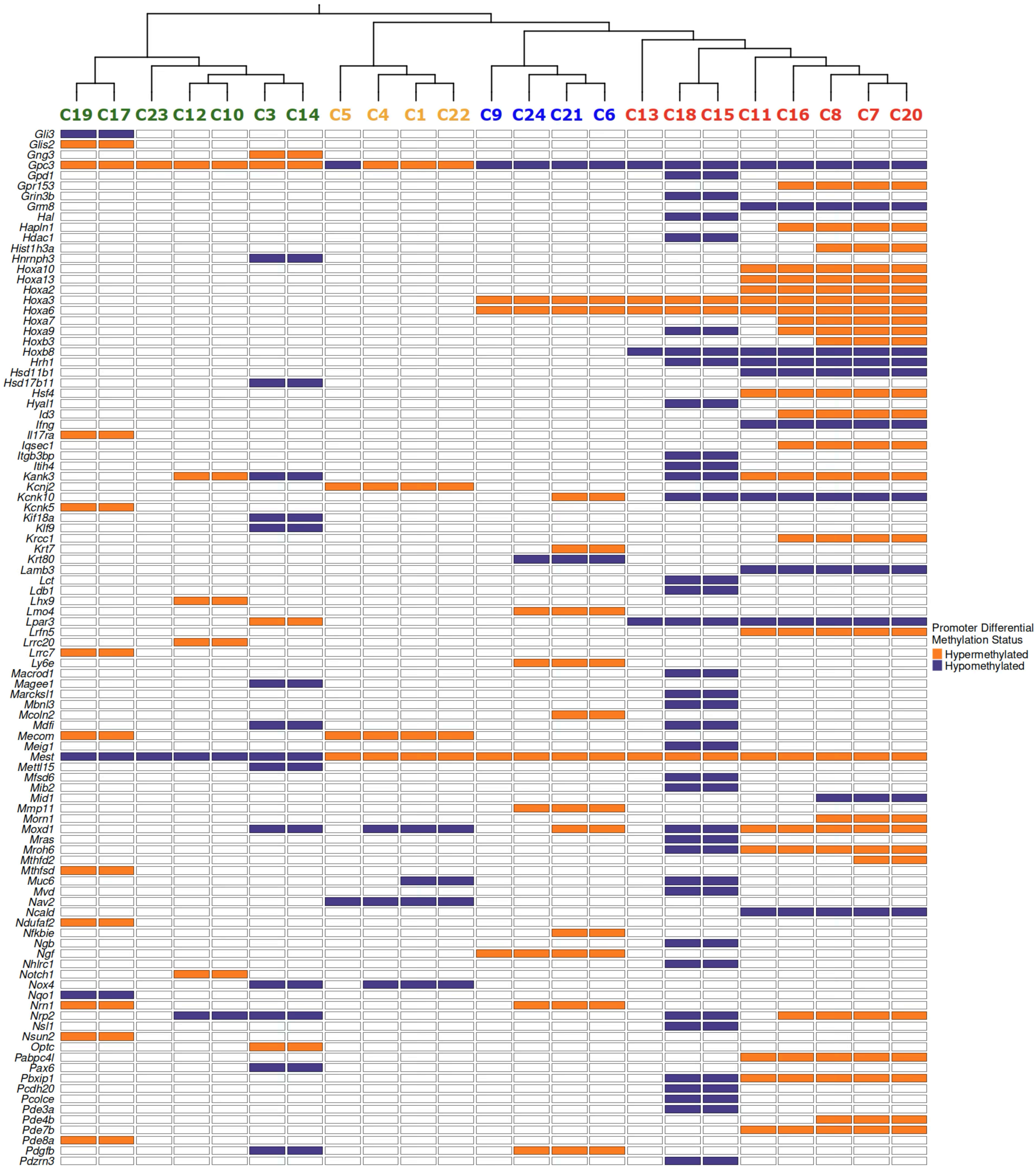

Figure S19: **Mouse orthologs of genes whose promoters are documented to be impacted by hypo- or hypermethylation in at least one of 17 types of human cancer according to OncoDB[27] and were found to be similarly impacted by promoter or enhancer differential methylation along the subline phylogeny (2/3).**

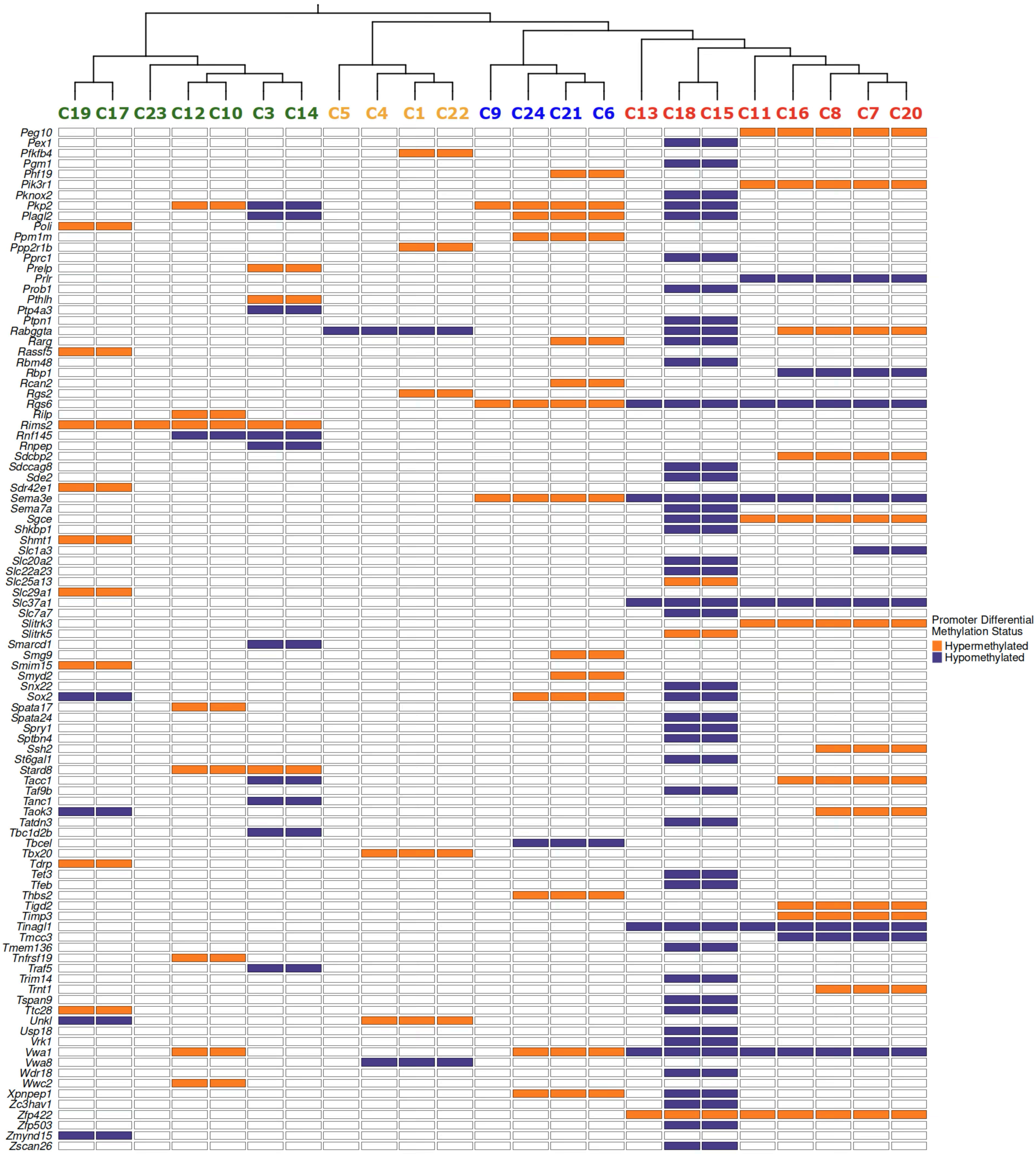

Figure S20: **Mouse orthologs of genes whose promoters are documented to be impacted by hypo- or hypermethylation in at least one of 17 types of human cancer according to OncoDB[27] and were found to be similarly impacted by promoter or enhancer differential methylation along the subline phylogeny (3/3).**

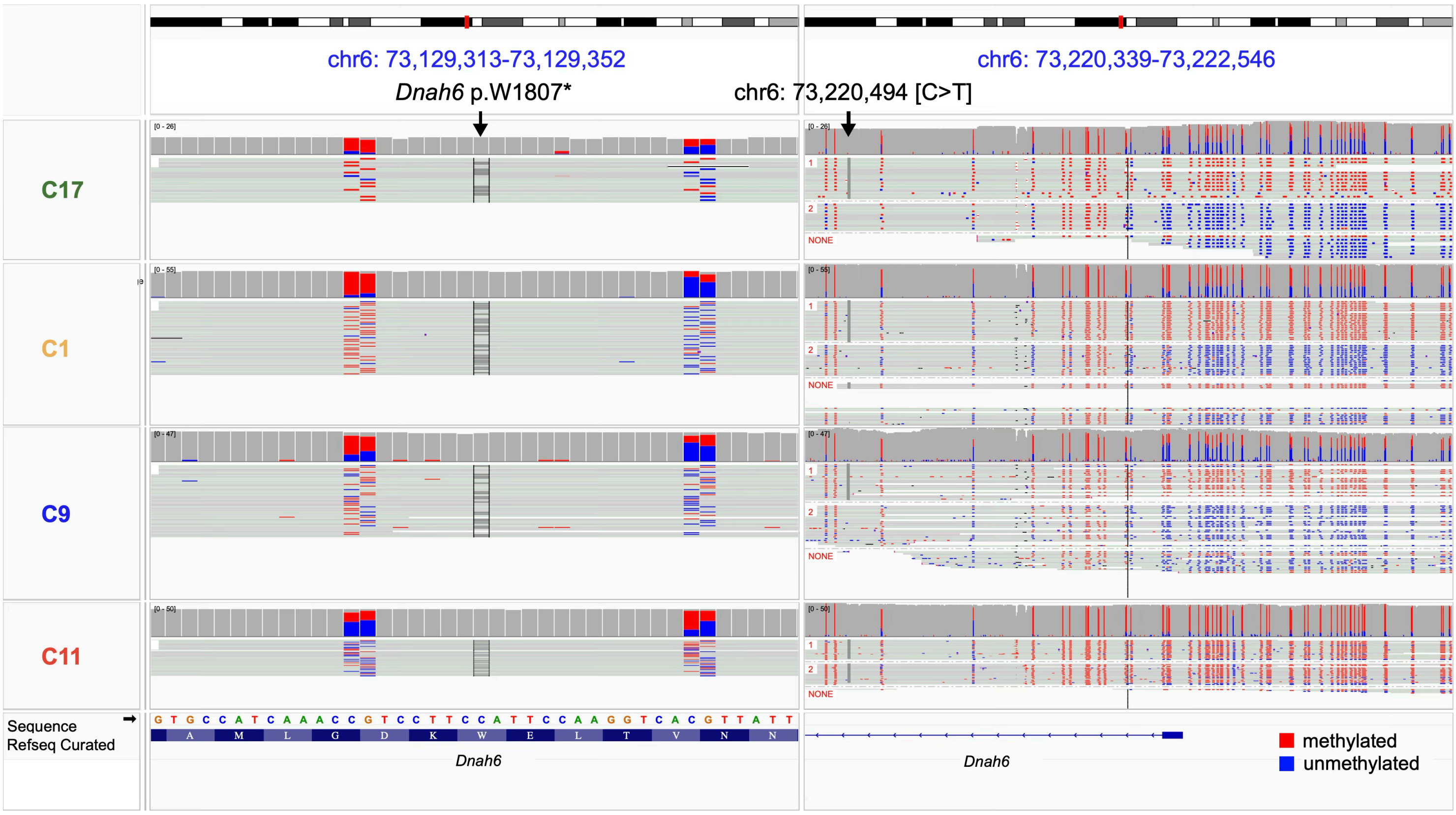

Figure S21: **Associating truncating *Dnah6* mutation with (haplotype-specific) methylation of *Dnah6* promoter in major clades.** One representative subline from each major clade is shown. Clonal truncating mutation *Dnah6* p.W1807* is marked in the left panel displaying unphased reads, and a heterozygous variant chr6: 73,220,494 [C>T] is marked on the right panel with reads phased according to the variant. In C11, the high VAF of *Dnah6* p.W1807*, together with the homozygosity of chr6: 73,220,494 [C>T], not only indicates LOH in C11, but also provides evidence that the two variants exist on the same haplotype. As the phased reads on the right panel are also colored by DNA methylation, we observe that methylation at *Dnah6* promoter is specific to the haplotype harboring chr6: 73,220,494 [C>T], and hence *Dnah6* p.W1807*. We observe haplotype-specific methylation of the *Dnah6* promoter in the green, orange, and blue clade, and hypermethylation in the red clade. Note that while here we observe a few reads from the wild-type copy of *Dnah6* in C11, we see a complete loss of the wild-type copy of *Dnah6* in all other sublines in the red clade, and along with that a complete hypermethylation of the *Dnah6* promoter.

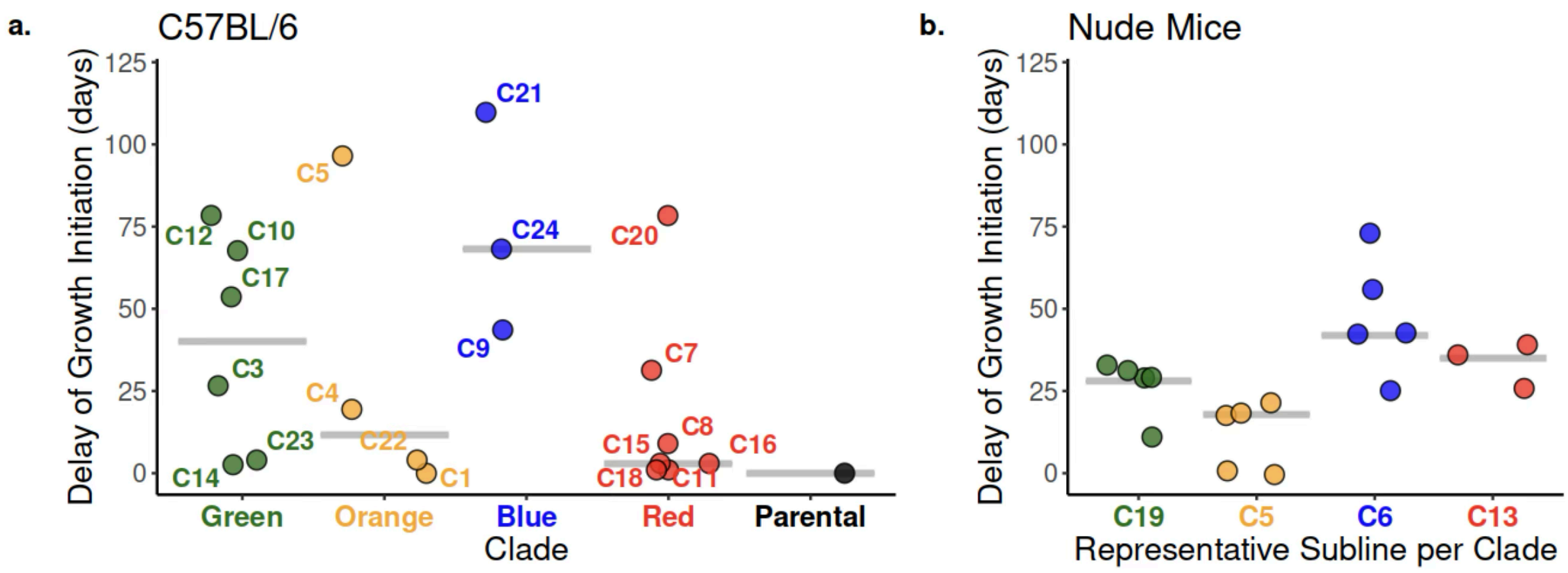

Figure S22: **Delay of growth initiation (DGI) of sublines in C57BL/6 and nude mice.** DGI is defined as the number of days between cell implantation in a mouse and the tumor started continuous growing without regression. **a.** One million cells of each subline were implanted into each of the five C57BL/6 mice (Day 1). The tumor size was measured twice a week. The mean DGI of the sublines in each clade, as well as the median across sublines means within a clade, was shown. No tumor was grown from any mice implanted with cells of C19, C6, or C13. Note that two mice implanted with C5 cells did not grow tumor, and they were not included in the calculation of mean DGI. These results suggested that, on average, sublines in blue clade induced the stronger host immunity than those in other clades. **b.** To investigate the association of the DGI with host immunity, the subline from each clade with the highest DGI following C57BL/6 implantation was tested in immunocompromised host. For each of C19 (green clade), C5 (orange clade), C6 (blue clade), and C13 (red clade), one million cells were implanted into each of the five immunocompromised nude mice (Day 1), and the DGI values measured from each mouse, as well as the median across mouse DGI values for a subline are shown. As expected, we observed the tumor grew in all mice with diminishing DGI as compared to the implantation in C57BL/6 mice. These results indicated that DGI is associated with subline response to host immunity.

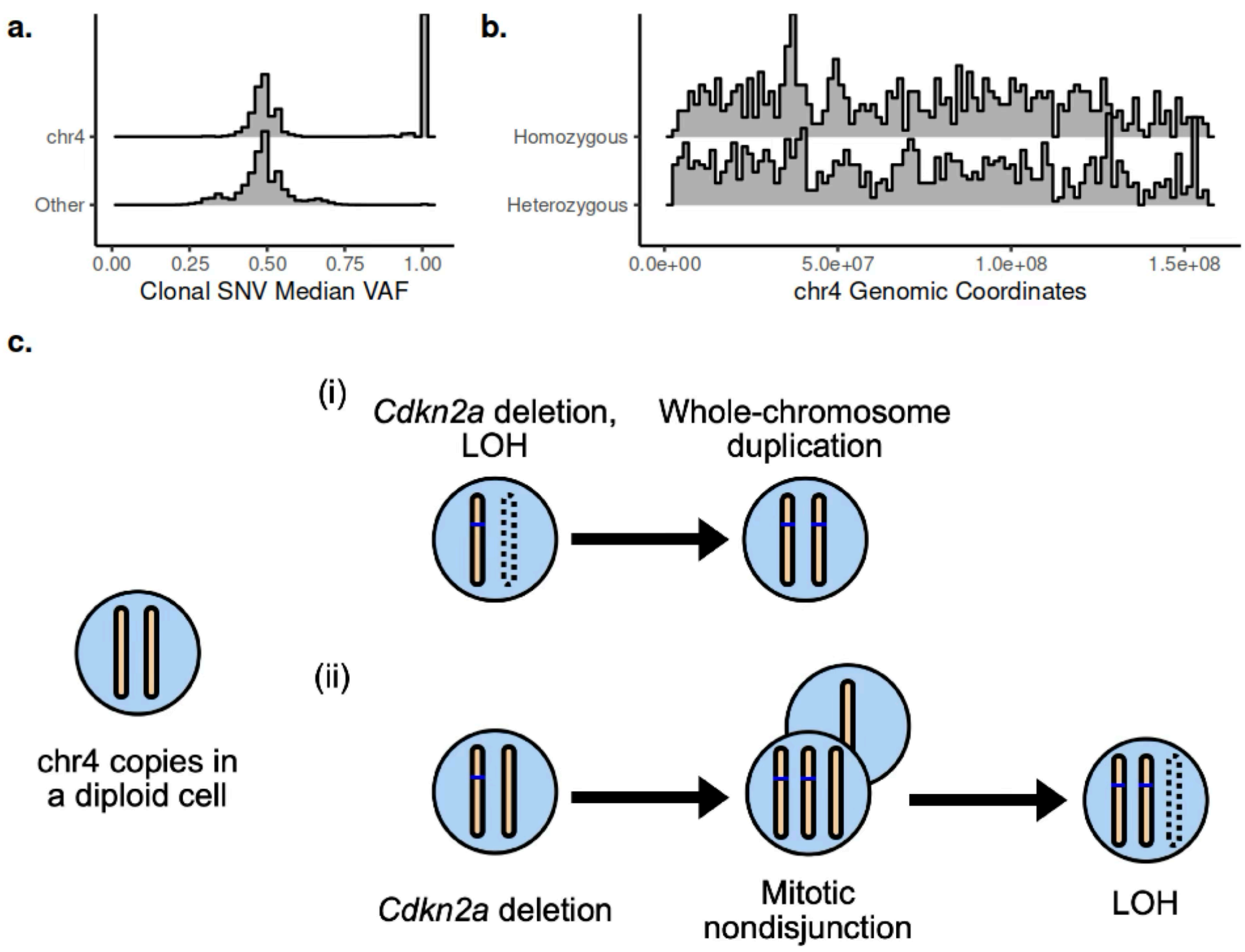

Figure S23: **SNV evidence for the timing clonal CNA events. a.** Median (across sublines) VAF distribution for clonal SNVs on chr4 and other autosomes. **b.** Genomic distributions of homozygous (median VAF = 1) and heterozygous (median VAF $<$ 1) chr4 clonal SNVs. **c.** Two indistinguishable scenarios that may have resulted in the copy-neutral LOH.

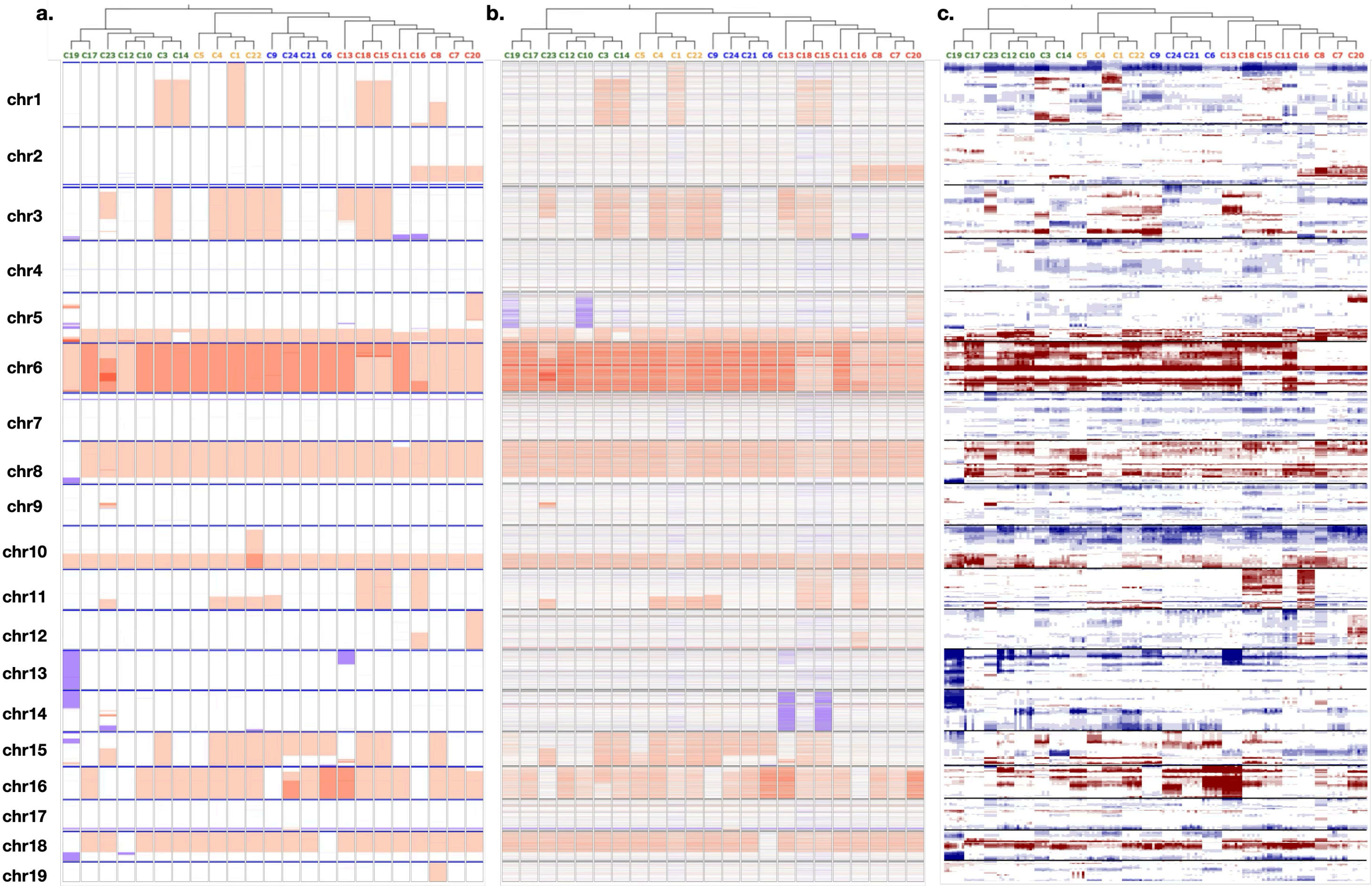

Figure S24: **CNA profiles of 23 sublines obtained using long-read, bWES, and scRNA-seq data. a.** Long-read-derived CNA profiles using `Wakhan` [2] (Figure 2e.; Methods) **b.** CNA profiles derived by `CNVkit` [25] from bWES data [15](Methods). **c.** CNA profiles derived by `inferCNV` [28] from full-length scRNA-seq data [9] obtained using the Smart-seq2 protocol [18] (Methods).

S1 Long-read whole-genome sequencing substantially improves resolution of genomic variants

Given the complex variational landscape of the B2905 evolution presented above, we next sought to quantify the improvement in resolution enabled by long-read sequencing, as compared to the short-read bWES and scRNA-seq of B2905 sublines [9, 15].

We compared different technologies in terms of quality and consistency of variant calls, as well as genomic coverage - which refers to the regions of the genome that are accessible to technology and covered by at least a single read (not to be confused with coverage depth, which refers to sequencing redundancy). For the SV discovery, we require the exact breakpoint coordinates to be known for an SV call to be made. Although approximate SV boundaries could be often discovered from sparse data, they can not fully resolve parallel events, which was critical in our study. We also note that other recent studies provided comprehensive evaluation of long-read sequencing for somatic SV discovery and illustrated their substantial improvements over short-read sequencing [11, 4].

Overall, long-read data had substantially better mappability and covered more variants (Figure S2a.), consistent with other benchmarks [12]. For our purpose of studying tumor evolution, the advantage is two-fold. First, long-read sequencing offers signals across the whole genome instead of focusing on exonic, expressed regions. This leads to (i) a substantially higher number of profiled somatic SNVs (Figure S2a.), (ii) identification of non-exonic SV breakpoints, and (iii) more contiguous estimation of copy number including non-exonic regions. Secondly, the reduction in mapping ambiguity avoids noisy signals due to mapping artifacts, amplifying real signals.

Using the available bWES and scRNA-seq data, we also validated the accuracies of the long-read profiled variants, quantifying the improvement in resolution when possible. Within bWES target regions, non-private somatic SNVs are $> 93\%$ consistent between long-read and bWES (Figure S2b.). Among those, $> 15\%$ are also corroborated by the scRNA-seq data . For each of the 639 `Severus`-profiled somatic SVs in long-read, we examined whether at least one of the breakends it is associated with has sufficient read coverage (200x after aggregating all reads from all sublines/cells) in bWES and scRNA-seq, as an overestimation of whether it can possibly be profiled in those technologies. Respectively, $> 86\%$ and $> 92\%$ of `Severus`-profiled somatic SVs have no breakends of sufficient coverage in bWES and scRNA-seq data (Figure S2c.), suggesting most SVs would have been missed in short-read data. We also profiled copy numbers from bWES and scRNA-seq data (Methods) to gauge the accuracy of long-read calls. Looking across all sublines, among genes with copy number estimates available across all 3 technologies, $> 94\%$ had consistent copy number status (neutral, gain, loss) between long-read and bWES (Figure S2f.). Among those, a majority also has its status corroborated in scRNA-seq calls, even though scRNA-seq calls suffer from noise and sparsity (Figure S2f., Supplementary Figure S24).

Importantly, as the detection of parallel CNA events relies on the association of contiguous copy number changes with exact SV breakpoints, using long-read whole-genome sequencing is in practice necessary for that purpose. For example, the 2 key subclonal parallel CNAs observed on chr1 (Figure 4) in our work, distinguished by their respective breakpoints, both have insufficient support in bWES and scRNA-seq data at the breakpoint loci, regardless of whether it is near-exonic (Figure S2d.) or intergenic (Figure S2e.).

In addition, we validated the `TreeHarmonizer` placement of SNVs using the orthogonal partition function approach [15] that estimates the likelihood of mutation placement (Methods). For the SNVs that were rescued by `TreeHarmonizer`, the partition function reported significantly higher scores (one-tailed paired-sample t-test $\mathrm{t}(26) = 11.99, p = 2.133e{-}12$), indicating accurate placement in the phylogeny (Figure S2g).

Aside from providing higher resolution SNV, SV, and CNA profiling, as well as being critical for identifying parallel CNAs, long-read sequencing additionally enables simultaneous profiling of methylation, which is impossible with short-read technologies without additional bisultifte-treated matched samples. Together, these demonstrate that in the specific context of studying tumor evolution, long-read whole-genome sequencing provides unrivaled advantages in revealing both genomic and epignomic insights compared to short-read alternatives.

S2 Validation of large long-read SV and CNA calls against spectral karyotyping (SKY) images

To validate SV and CNA calls produced in this study, we generated spectral karyotyping (SKY) images for 12 sublines and parental cell line (Figure S25). The SKY technique can detect large-scale karyotype changes, for example gain or loss of whole chromosomes. Further, translocations between different chromosomes could be detected as hybrid chromosomes with probes with different colors. It is however difficult to detect small SVs, or copy-neutral SVs with breakpoints inside the same chromosome. Thus, here we focused on validation of whole-chromosome CNAs reported by Wakhan and inter-chromosomal SVs inferred with long reads. For each subline, we manually overlapped CNA and SV calls with the annotated translocations in SKY images and the number of chromosome copies (Table S8). Overall, we observed a high agreement in both number of copies and inter-chromosomal fusions between the technologies.

In a limited number of cases, we also observed apparent systematic disagreements in certain fusion calls. A likely explanation for that is that some SKY fusions were miscalled because of the color similarity of the chromosome probes. For example, clonal translocation chr8-chr10 reported by Severus seem to correspond to chr8-chr9 call in some SKY images; however manual inspection of probes coloring support chr8-chr10 fusion instead (Figure S25). Similarly, chr5-chr8 translocation in some SKY images likely corresponds to chr5-chr18 in Severus. In addition, chr5-chr18 may be systematically missed in SKY because of the probe colors of two chromosomes were similar. Separately, we found that six long-read calls with evidence of subclonality were not supported by SKY.

To validate this hypothesis, we performed an additional SKY experiment with C1 subline with just chr8 and chr10 probes, and confirmed the chr8-chr10 translocation, which was initially miscalled as chr8-chr9 by the analysis software (Figure S26). In addition, in the case of C13, an initial chr8-chr9 call changed to chr8-chr10 call with the HiSKY software update (v8.2.2). This validates our reasoning that some translocations were systematically miscalled in SKY experiments.

With the miscalls noted above corrected, and subclonal long-read calls removed, most sublines showed approximately 90% agreement on average between SKY and long-read calls (Table S8). C19 and C22 sublines were outliers with surprisingly little consistency between the technologies. It is possible that these sublines were mislabelled, as the analysis of these samples with multiple technologies was performed by different groups over several years. Overall, this comparison confirms high quality of the large CNA and SV calls produced by the long-read methods in this study.

Culture Methods. Sublines were cultured for SKY analysis by NCI Molecular Cytogenetics Core Facility. The metaphases were arrested by incubation with Colcemid (15210-040, KaryoMax Colcemid Solution, Invitrogen, Carlsbad, Calif., USA) (10ug/ml) 3 hours prior to harvest. Sublines that can form appropriate metaphases were subjected to SKY analysis. Cells were collected and treated with hypotonic solution (KCL 0.075M,6858-04, Macron Chemical) for 15 minutes at 37°C and fixed with methanol: acetic acid 3:1. Slides were prepared and aged overnight for use in SKY analysis.

Spectral Karyotyping. The metaphases were hybridized with the 21-color mouse SKY paint kit (FPRPR0030, ASI) according to manufacturer's protocol. Hybridization was carried out in a humidity chamber at 37°C for 16 hours. The post-hybridization rapid wash procedure was used with 0.4XSSC at 72°C for 4 minutes. Detection was carried out following ASI manufacturer's protocol. Spectral images of the hybridized metaphases were acquired using Hyper Spectral Imaging system (Applied Spectral Imaging Inc., CA) mounted on top of an epi-fluorescence microscope (Imager Z2, Zeiss). Images were analyzed using HiSKY 7.2 acquisition software (Genasis, Applied Spectral Imaging Inc., CA). G-banding was simulated by electronic inversion of DAPI counterstaining. An average of 10-15 mitoses of comparable staining intensity and quality was examined per cell-line and were compared for chromosomal abnormality. The karyotype was determined by comparison to the standard ideogram of banding patterns for mouse chromosomes.

Sample	SKY chr. copies	Long-read chr. copies	SKY fusions	Long-read fusions
C1	chr1:3, chr3:3, chr6:5, chr8:3, chr15:3, chr16:3 chr18:3	chr1:3, chr3:3, chr6:4, chr8:3, chr15:3, chr16:3, chr18:3	chr8-chr9, chr5-chr18 chr15-chr11	chr5-chr18, chr8-chr10, chr11-chr15
C4	chr3:3, chr5:3, chr6:3, chr8:4, chr13:1, chr15:3, chr16:3, chr18:3	chr3:3, chr6:4, chr8:3, chr15:3, chr16:3, chr18:3	chr8-chr9, chr8-chr15, chr5-chr18, chr15-chr11	chr5-chr18, chr8-chr10, chr11-chr15
C5	chr5:3, chr6:4, chr14:1, chr16:4, chr18:3	chr6:4, chr8:3, chr16:3, chr18:3	chr5-chr8, chr8-chr9, chr5-chr18	chr5-chr15, chr5-chr18, chr8-chr10
C6	chr6:4, chr8:3, chr15:3, chr16:4	chr6:4, chr8:3, chr15:3, chr16:4	chr8-chr9, chr15-chr18, chr5-chr18	chr5-chr18, chr8-chr10, chr15-chr18
C8	chr6:3, chr8:3, chr18:4	chr6:3, chr8:3, chr18:3, chr15:3 (s), chr16:3 (s), chr19:3 (s)	chr8-chr9, chr1-chr3 (s),	chr5-chr18, chr8-chr10
C11	chr6:4, chr16:3, chr18:3	chr6:4, chr8:3 (s), chr16:3, chr18:3	chr8-chr9, chr5-chr18	chr3:chr12 (s), chr5-chr18, chr8-chr10
C12	chr6:4, chr8:3, chr16:3, chr18:1	chr6:3, chr8:3, chr15:3, chr16:3, chr18:3	chr5-chr18, chr8-chr9	chr5-chr18, chr8-chr10
C13	chr1:1, chr6:3, chr8:3, chr10:1, chr15:1, chr16:4, chr18:3	chr2:3, chr6:4, chr8:3, chr16:4, chr18:3	chr3-chr5, chr9-chr8, chr3-chr13, chr5-chr18	chr3-chr13, chr5-chr18, chr8-chr10
C14	chr1:3, chr5:3, chr6:4, chr8:3, chr16:3	chr1:3, chr6:4, chr8:3, chr16:3, chr18:3	chr1-chr15, chr5-chr16, chr5-chr8, chr8-chr9	chr1-chr15, chr5-chr16, chr5-chr18, chr8-chr10
C15	chr1:3, chr3:3, chr6:3, chr8:3, chr11:3, chr16:3, chr18:3	chr1:3, chr3:3, chr6:3, chr8:3, chr11:3, chr15:3, chr16:3, chr18:3	chr8-chr9, chr5-chr18, chr1-chr14	chr1-chr6, chr5-chr18, chr8-chr10
C19	chr1:3, chr3:1, chr5:3, chr6:3, chr8:4, chr16, chr18:1, chr19:1	chr6:3, chr13:1	chr1-chr7, chr5-chr19, chr5-chr14, chr5-chr8, chr8-chr9, chr11-chr15	chr5-chr15, chr5-chr18, chr8-chr10
C22	chr1:3, chr3:3, chr6:4, chr7:1, chr16:3, chr18:3	chr3:3, chr6:4, chr8:3, chr10:3, chr15:3, chr16:3, chr18:3	chr1-chr11, chr5-chr18 chr11-chr15	chr5-chr18, chr8-chr10, chr10-chr14, chr11-chr15

Table S8: Comparison of CNAs and fusions detected by SKY and Wakhan across samples. The table shows the original SKY calls before correction. Several recurrent translocations were systematically miscalled in SKY images because the corresponding chromosomes have very similar probe colors: chr8-chr9 in SKY corresponds to chr8-chr10 in Severus; chr5-chr8 in SKY corresponds to chr5-chr18 in Severus. In addition, chr5-chr18 may be systematically missed in SKY because of the probe colors similarity. (s) denotes subclonal event.

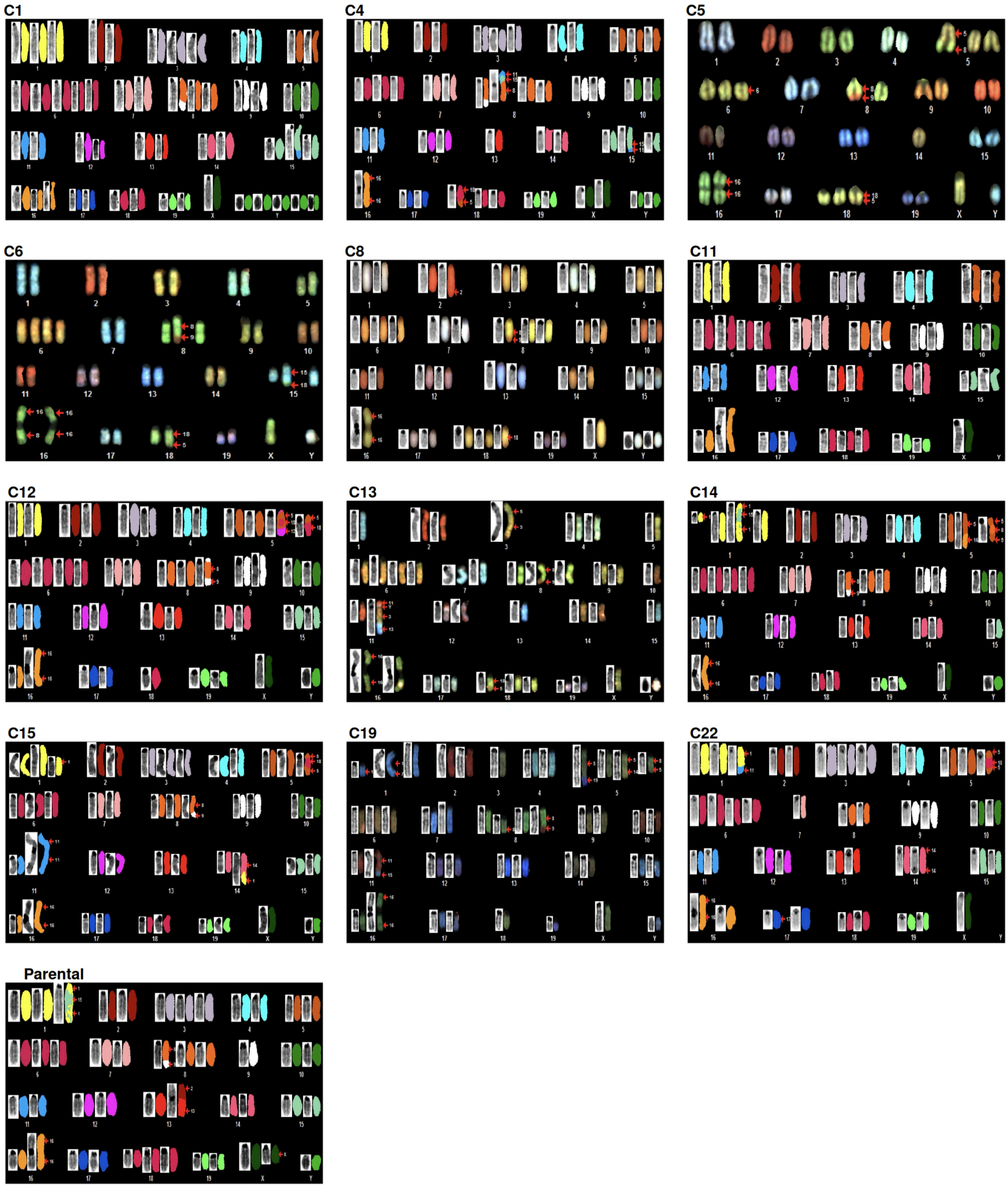

Figure S25: **Spectral Karyotyping (SKY) imaging data for available sublines, as well as for the parental line.**

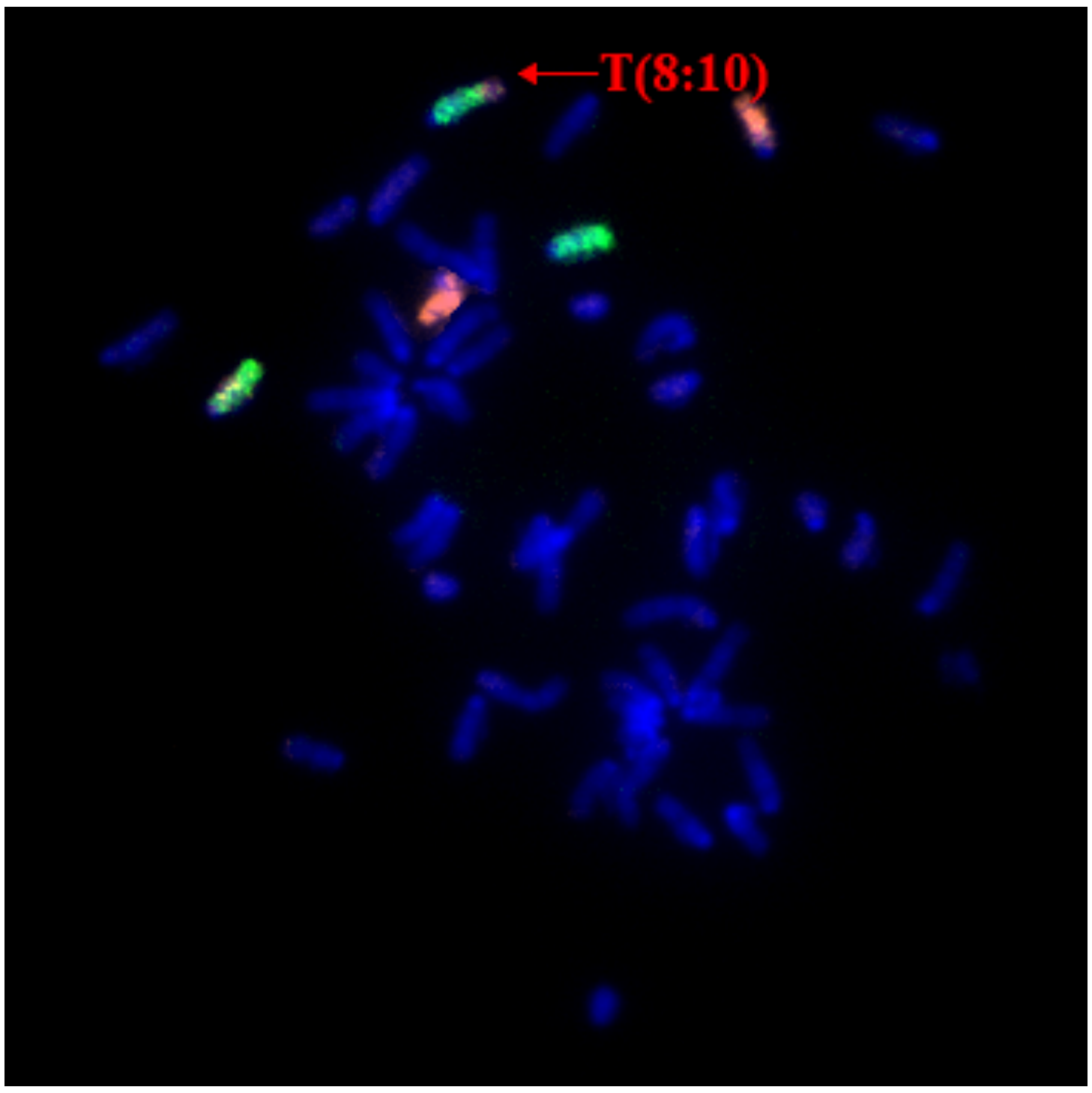

Figure S26: **Whole chromosome paint validation of chr8-chr10 translocation in C1 using two chromosome probes.**

S3 Comparison of SNV, SV and CNA calls against orthogonal tools

In order to validate the DeepVariant SNV calls, Severus SV calls and Wakhan CNA calls, we compared the variants to those called by orthogonal state of the art tools.

SNV callers comparison. While there exist many SNV calling methods, only several state of the art tools currently support long-read sequencing technologies. We thus compared the results of our selected tool, DeepVariant [19], to that of Clair3 [30], ClairS [31] and DeepSomatic [17]. DeepVariant and Clair3 are tools largely developed for detecting germline SNVs in a variety of datasets. Both have models and pipelines specifically developed for long-read ONT data for the R10.4 chemistry. DeepSomatic and ClairS are the somatic variant calling counterparts to the original germline calling tools. We ran each of these 4 tools with their default recommended parameters for ONT R10.4 data on each subline independently. We stratified all of the SNV calls into three groups, Clonal, Subclonal, and Private, by using a simplified definition – a variant is considered clonal if it is called in all the sublines, subclonal if called in 2 or more but not all, and private if it is called in only one subline. We then analyzed the intersections of unique variants between all of the callers, which were visualized using UpSet plots (Supplementary Figures S27a, S27b, S27c). For an unknown reason DeepSomatic was unable to generate calls for subline C16. It was thus excluded from this analysis across all callers.

Reassuringly, we found high concordance between DeepVariant, ClairS and DeepSomatic across clonal, subclonal and private variants in per-subline SNV calls. Clair3 stands out among the callers with a substantially lower number of clonal variants, and a substantially higher number of private variants. Both these differences indicate a likely higher false positive and false negative rate for Clair3 versus the other tools.

Given the high concordance between callers and a lack of a ground truth, we believe that in general, DeepVariant, DeepSomatic and ClairSomatic are reasonable for use in downstream analysis. DeepVariant was originally chosen for several reasons. Firstly, DeepSomatic was not available at the time this analysis began, and in its current state still suffers from various bugs that make it more difficult to use and validate. Clair3 was rejected due to its higher FP and FN rate for somatic mutations. ClairS was unpublished at the time the core analysis for this manuscript was performed.

SV callers comparison. Somatic SVs were identified using Severus and Sniffles2 [24], the only two long-read SV callers supporting multisample analysis. Sniffles2 (v2.7.1) was run in multisample mode for all sublines and the normal sample, using the --tandem-repeats option. Germline SVs specific to the FVB strain were filtered from the multisample vcf using a maximum distance threshold of 500 bp. Multisample VCFs produced by Severus and Sniffles2 were subsequently merged using minda [11] with default parameters. The resulting SV set was annotated with VNTR and collapsed duplication regions generated by Severus.

We placed the detected SVs from both tools on the tree using TreeHarmonizer. SVs detected by both tools (n = 489) were all consistent with the phylogenetic tree. In contrast, SVs unique to Sniffles2 (n = 1703) were often inconsistent with the tree structure, likely driven by mismapped reads in collapsed duplication regions (76%) (Supplementary Tables S9, S10). By comparison, most SVs uniquely detected by Severus were supported by the phylogenetic relationships.

Separately, we also verified that number of Sniffles2 SV calls placed in the different branches of the tree were correlated with the number of corresponding SNVs (Supplementary Figure S28).

This analysis suggests that (i) most informative subclonal and clonal SVs detected by Severus were also supported by Sniffles2 and (ii) Sniffles2 generated many likely false-positive calls. This is likely because Severus was specifically designed for cancer genomes. Therefore we used Severus calls for the evolutionary analysis.

SV Category	Sniffles2	Severus
Unplaced	1059	77
Private	795	876
Clonal	266	96
Subclonal	72	57

Table S9: **SV counts by phylogenetic tree placement category for Sniffles2 and Severus.**

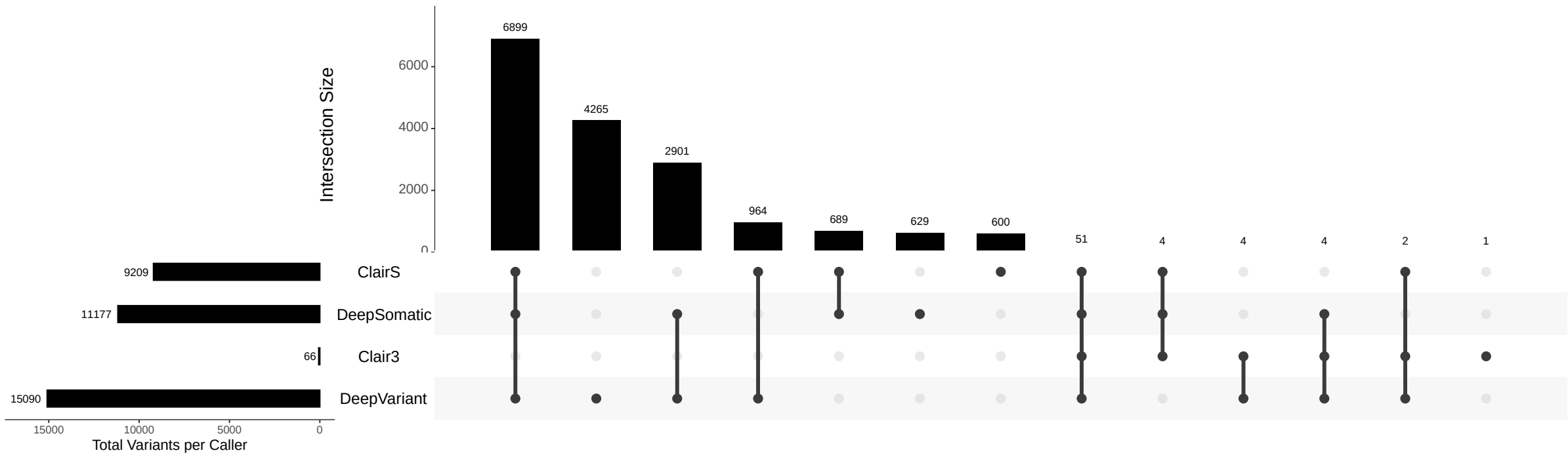

(a) **Clonal SNVs stratified by variant caller.**

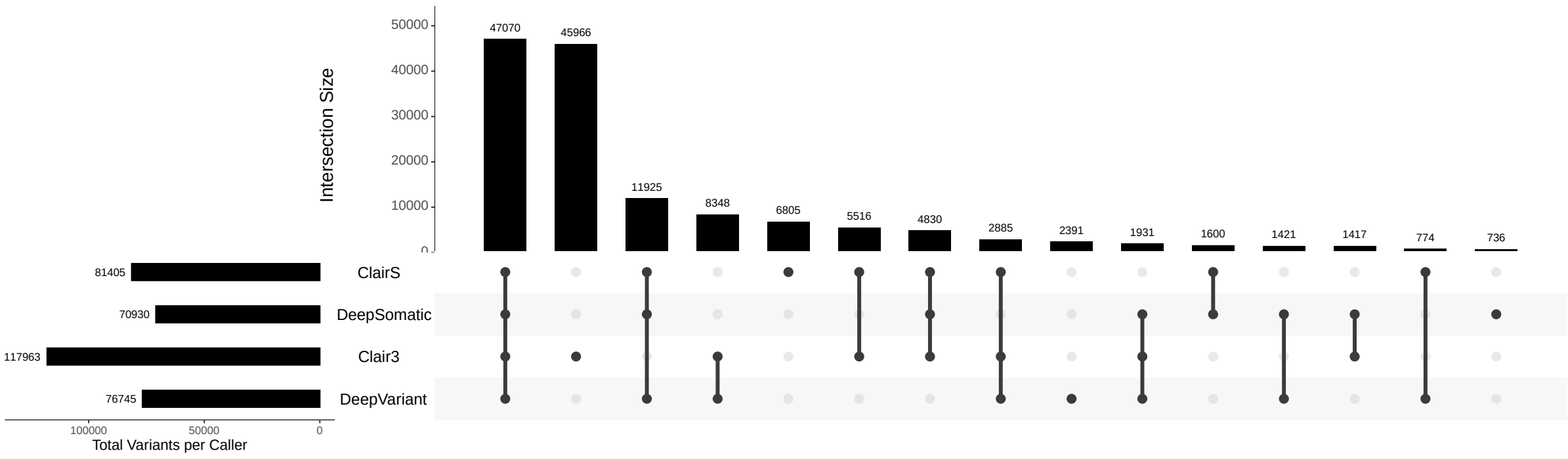

(b) **Subclonal SNVs stratified by variant caller.**

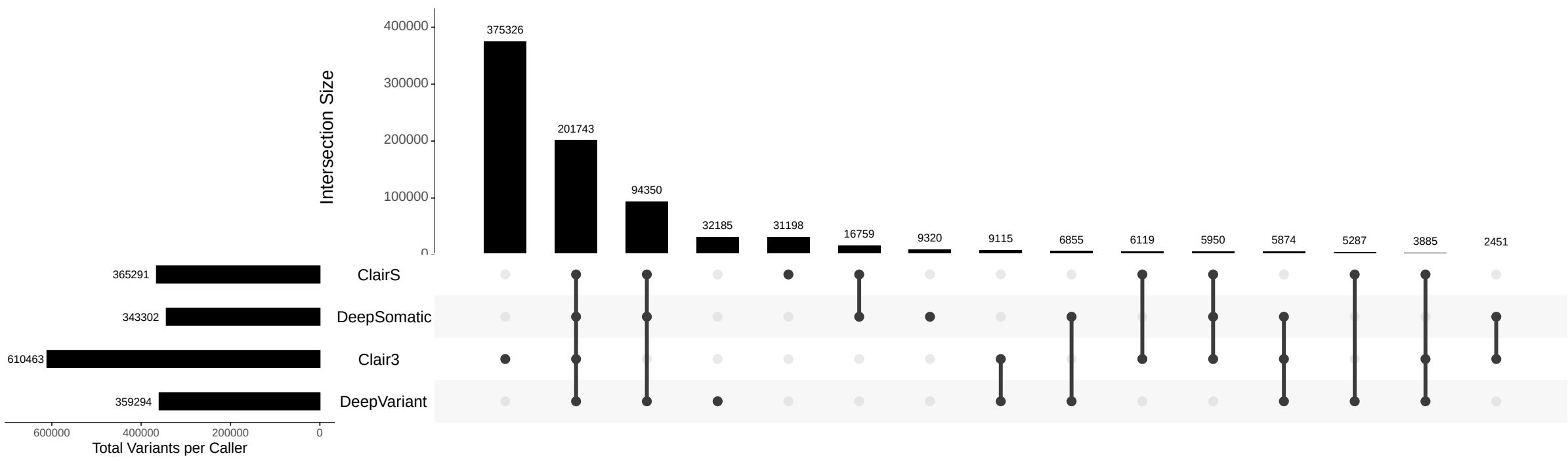

(c) **Private SNVs stratified by variant caller.**

Figure S27: **UpSet plots showing SNV concordance between variant callers, stratified by clonality.** Clonal here is defined as the presence of a unique variant in all sublines, subclonal as presence in two or more but not all, and private as presence in one subline.

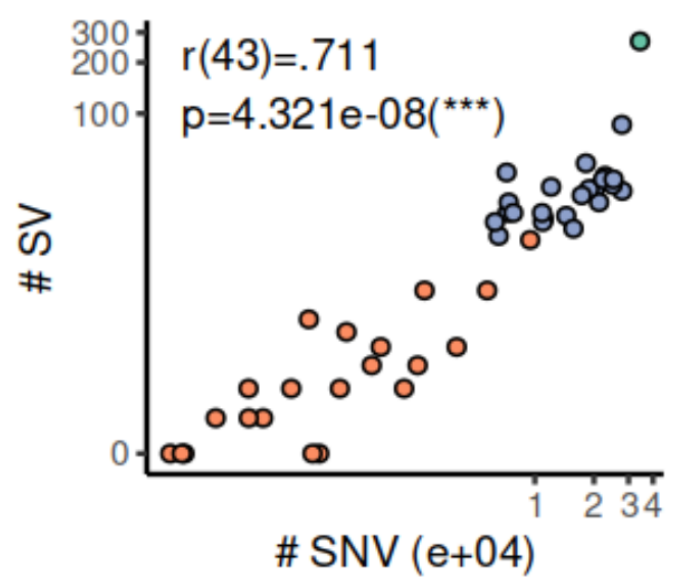

Figure S28: **The correlation between number of SNVs and Sniffles2 SVs calls placed at each branch of the phylogeny.**

SV Category	Sniffles2	Severus
Shared between both tools	489	489
Collapsed duplication	659	102
VNTR	634	35
Nearby SV	220	159
Failed SV in Severus	67	-
Unplaced / private	93	307
Clonal / subclonal	30	14

Table S10: **Summary of SV failure modes and discrepancies between Sniffles2 and Severus across major SV categories.**

CNA callers comparison. Finally, we compared the CNA calls of Wakhan against another popular caller, CNVkit [26]. Most other state of the art callers, such as PURPLE [20], were not suitable, as they rely on human genome annotations, which cannot be easily changed by the end-user. CNVkit was run with 50kb bin sizes to match the bins used in Wakhan by default. Both Wakhan and CNVkit produce integer copy number calls files with exact basepair start and end positions for that call. In order to facilitate a comparison between the two tool's integer calls, we divided each of the reported copy number spans into segments of 50kb each. (This step is necessary as having 50kb bin sizes for calling does not result in the calls being output in 50kb segments, rather it affects the span for which candidate copy number changes are considered). If a breakpoint fell between these 50kb segments, the copy number for the bin before and after was determined with a weighted average by the length of its span.

We find that the concordance between the integer copy number calls of Wakhan and CNVkit was very high, with a Pearson correlation coefficient of $r = 0.9530$, ($p = 1.0621e - 24944$) across all sublines (Table S11, Figure S29). We further measure the concordance between the callers as the percentage of bins with matching integer copy numbers across each subline. We find that across all sublines we have an exact match between 92.84% of all 50kb bins. The vast majority of sublines (n=17) have exact matching percentages of >= 95%. We note that two sublines, C1 and C18 stand out in having significantly lower exact matching percentages of 47.18% and 51.40% respectively, while still having very high correlations of $r = 0.9530$ ($p = 2.94943e-24946$) and $r = 0.8998$ ($p = 9.52699e-17322$), respectively. This discrepancy indicates that while the callers are indeed concordant with respect to the progression of copy number changes along the genome, there is a systemic miscalling between the callers of the exact copy number integer. Indeed upon further investigation that is exactly what we find, where 50% of the bins that were called as copy number 2 in Wakhan were called as copy number 1 in CNVkit. This suggests that much of the coverage across these sublines falls very near the border where the baseline CN1 coverage is determined. It is noted that the p-values are very low due to the high number of bins, and statistical significance is trivial to achieve in such a comparison where bins are genomically related.

Furthermore, several differences between the tools further explain lower concordance levels. Firstly, exact breakpoints around copy number changes will likely differ between callers. This is due to the fact that Wakhan is able to incorporate precise breakpoint information from SV callers such as Severus, whereas CNVkit does not. Furthermore, the standard segmentation method of CNVkit (cbs, circular binary segmentation) is not able to detect smaller, focal amplifications and losses. While a hidden markov model mode exists within CNVkit for such focal aberrations, it is currently labeled as experimental only, and thus was not benchmarked against.

Overall, the high concordance between Wakhan and CNVkit suggest that the copy number calls used in the evolutionary analysis are highly reliable.

**Subline**	**# Bins**	**Pearson's r**	**Pearson's p**	**Exact Match %**
C1	48080	0.9530	$2.94943e-24946$	47.18
C3	48080	0.9872	$2.39976e-38348$	99.39
C4	48080	0.9861	$1.50433e-37464$	99.31
C5	48075	0.9466	$1.07771e-23641$	96.70
C6	48067	0.9873	$1.78688e-38403$	99.26
C7	48068	0.8769	$3.14673e-15296$	95.38
C8	48080	0.9661	$9.65852e-28284$	98.75
C9	48077	0.9852	$7.05786e-36825$	99.23
C10	48075	0.9857	$4.69013e-37216$	99.38
C11	48077	0.8961	$1.34313e-16961$	93.31
C12	48080	0.8634	$6.04884e-14289$	96.61
C13	48071	0.9467	$9.49335e-23647$	95.45
C14	48079	0.9863	$9.69533e-37666$	99.39
C15	48068	0.8705	$5.47468e-14802$	91.89
C16	48075	0.8988	$9.0844e-17217$	95.44
C17	48072	0.9691	$7.49641e-29225$	98.29
C18	48080	0.8998	$9.52699e-17322$	51.40
C19	48070	0.8874	$3.12087e-16169$	94.64
C20	48070	0.9760	$6.09304e-31839$	99.31
C21	48079	0.9862	$3.38166e-37558$	99.38
C22	48077	0.9633	$7.12234e-27449$	97.25
C23	48080	0.8910	$5.73703e-16488$	91.91
C24	48080	0.9372	$4.2414e-21990$	96.58
Mean	48076	0.9411	$4.82258e-22647$	92.84
Median	48077	0.9530	$1.0621e-24944$	96.70

Table S11: **CNV concordance statistics between Wakhan and CNVkit.** Exact match percentage refers to the percentage of equivalent copy number calls between the two callers over all 50kb bins. A two-sided Pearson correlation test was used to evaluate concordance between caller outputs. Provided are the correlation coefficient (r) and corresponding p-values. Note that p-values are very low due to the very high amount of bins, and statistical significance is trivial to achieve in this experimental context. The analysis focuses on the relationship between the correlation coefficient and the exact match percentage.

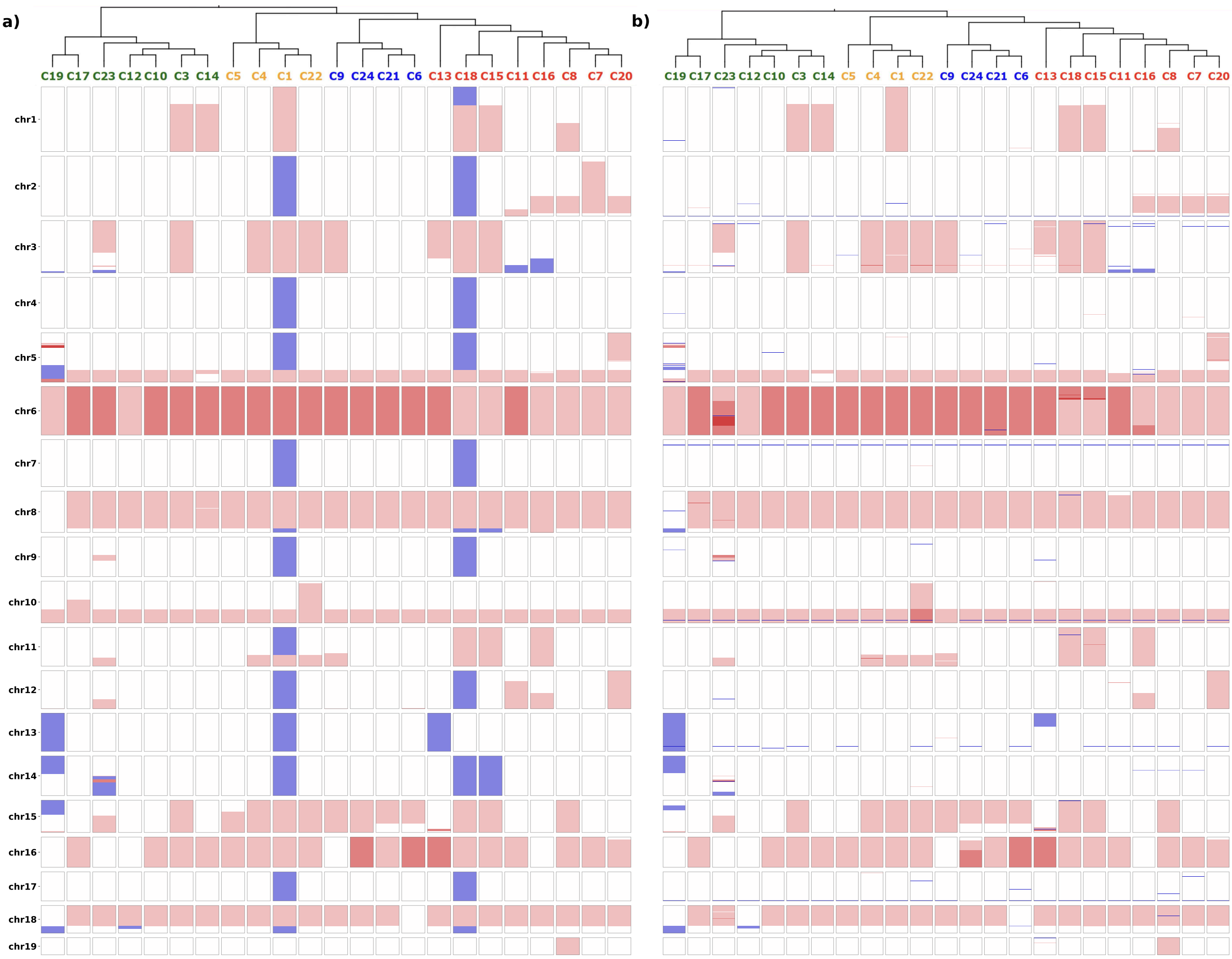

Figure S29: **CNA profiles of the 23 sublines using long-read data across CNA callers. a.** CNA integer calls as obtained by CNVkit using 50kb bins. **b.** CNA integer calls as obtained by Wakhan using 50kb bins. Telomere and centromere regions are not shown or colored.

S4 Evaluating alternative methods for joint SNV and CNA phylogeny reconstruction

There exist several methods that are conceptually similar to TreeHarmonizer in that they jointly consider SNVs, SVs and CNAs in phylogenetic placement. However, all of these methods have practical limitations that make it difficult to apply them to the analysis of single-cell derived subclones. Many methods, such as TUSV-ext [7], and Meltos [21], are designed for multi-regional bulk sequencing samples as input, and thus are solving a clonal deconvolution problem. In our experimental setup, our samples already represent distinct individual clones, which requires a different approach. Methods such as SCsnvcna [29] require a CNV tree inferred from an orthogonal biological dataset (for example, a separate set of cells) - which is also not applicable to our scenario since the number of subclones is relatively small, compared to a number of analyzed cells in a single-cell experiment.

There do exist tools that can, in principle, be applicable to our data. Two such tools are SCARLET [23] and ConDoR [22], which were designed for single-cell input. We examined the possibility of running these tools for our dataset. In our data, there were ∼450k unique SNVs among all sublines, ∼270k of which were phylogenetically informative. That set was then filtered down to ∼10.8k high quality SNVs, as described in Methods. In contrast, SCARLET and ConDoR were both originally benchmarked on much smaller sets of variants, up to 100 SNVs (with CNV clusters of size 3-5). Both tools were expressly tested and made for limited targeted sequencing methods, and thus would be limited to significantly smaller amounts of SNVs.

To test if alternative tools could be applied for our data, we extended the benchmarking that was done on SCARLET and ConDoR to increasing quantities of variants that were subsampled from across our 23 sublines (Supplementary Figures S30a S30b). We sampled SNVs in steps of 25, up to 300 SNVs for ConDoR, with three different random sampling seeds. We provided 4 CNV clusters to ConDoR using hierarchical clustering, given the knowledge that our dataset is broken down into 4 major color clades.

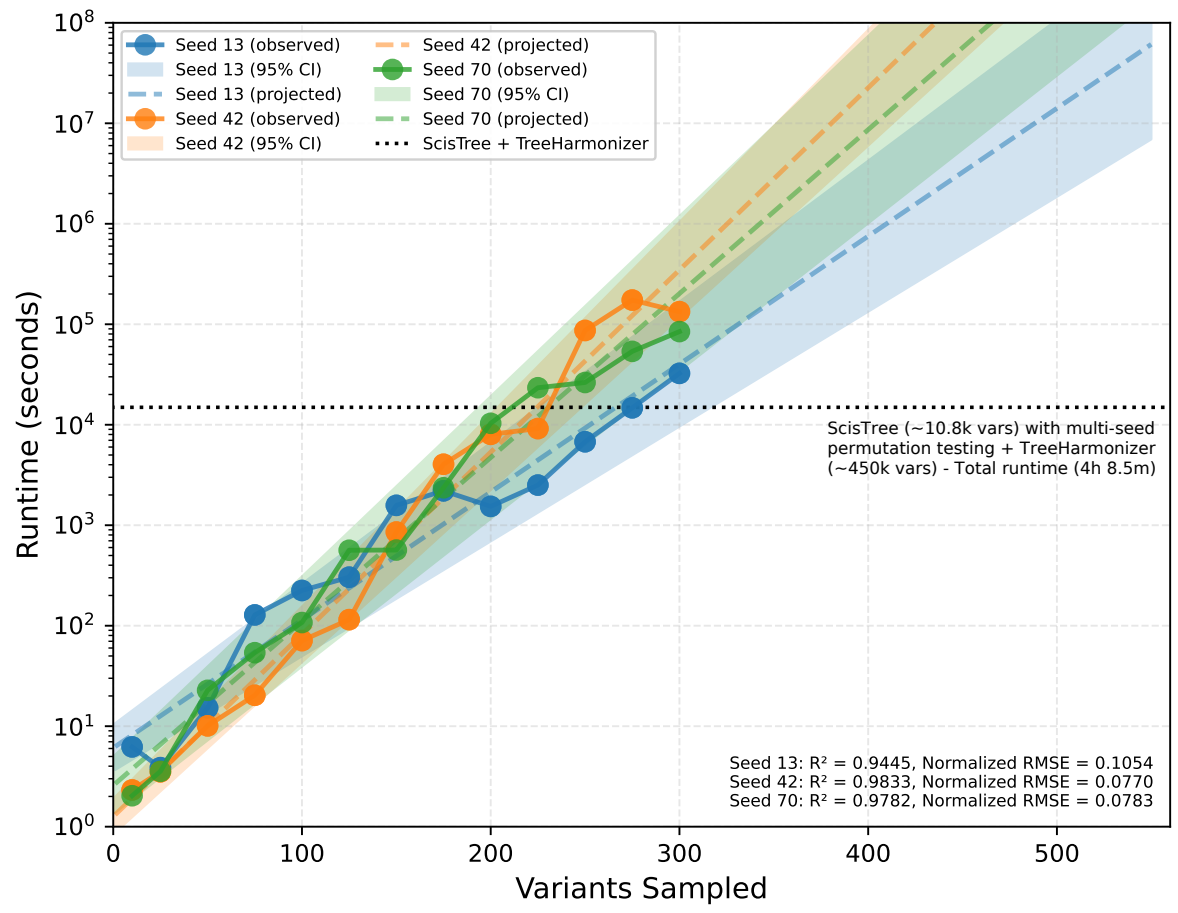

(a) **ConDoR benchmarking**

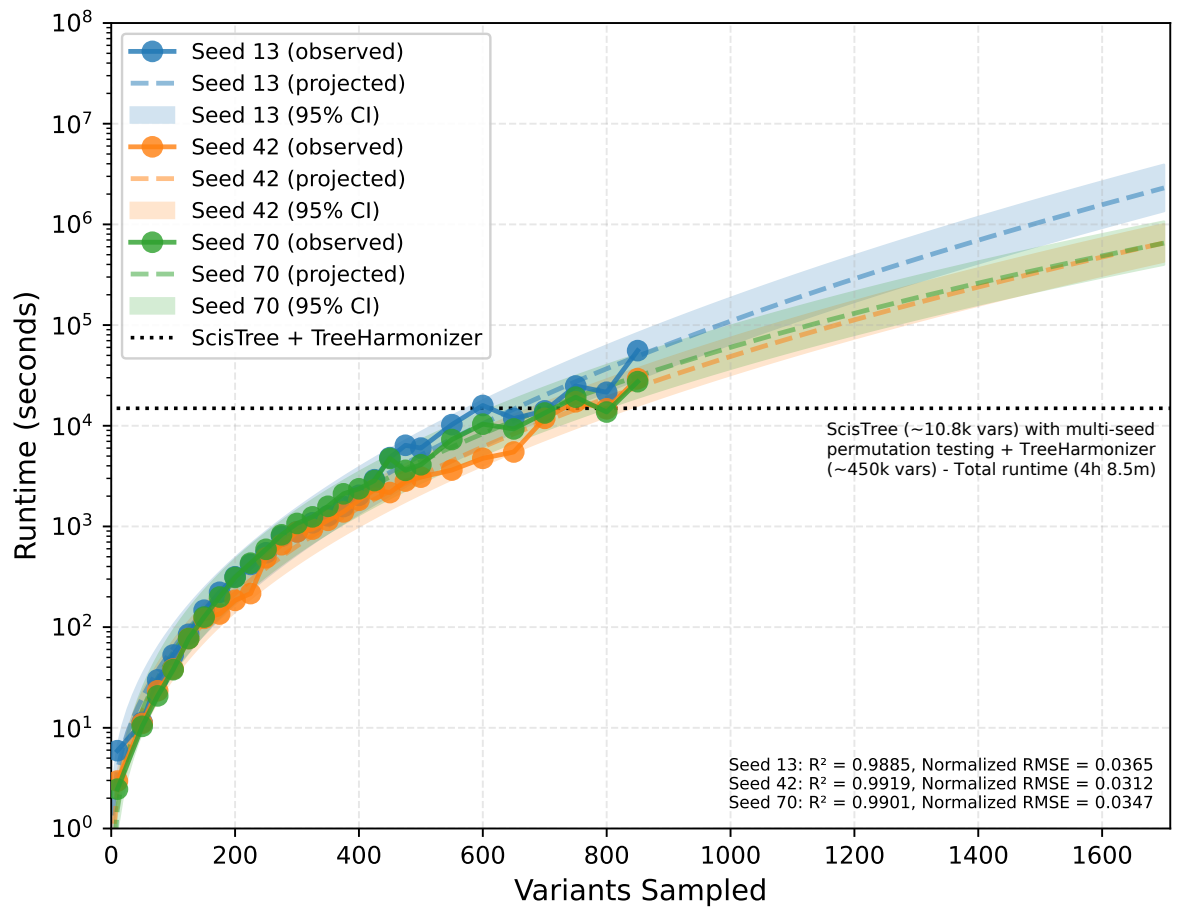

(b) **SCARLET benchmarking**

Figure S30: Extending SCARLET and ConDoR benchmarking to higher quantities of variants. Plotted are the observed points, and a projected dashed line based on the curve of best fit per sampling seed. A 95% confidence interval is also shown per seed. ScisTree + TreeHarmonizer represents the total runtime of the two tools together on this dataset as described in Methods.

The experiment was repeated 5 times on the same cluster with 100GB of RAM and 56 available CPUs. Runtimes were averaged over all repetitions. We found that ConDoR scales exponentially, with an observed runtime of around 27 hours for 2 out of 3 seeds at 300 SNVs. Based on the average fit line equation across all seeds,

$$t = 2.701768 \cdot e^{0.036212(v)}$$

where t represents the total runtime, and v represents the number of variants, even running on just 500 variants with $k = 1$ would take over 49,000 hours, rendering it functionally impossible to run our data with any reasonable sampling. Increasing the value of k (allowable losses under the k-dollo model) further increases the computation time.

SCARLET was run with sampled SNVs in steps of 25 up to 500 SNVs, and in steps of 50 until 850 SNVs. Due to the increased memory usage of SCARLET over ConDoR, it was run on a cluster with 256GB of RAM, with 56 available CPUs. Each subsample run was repeated 8 times per variant quantity. We find that SCARLET scales in a sub-exponential manner. The most appropriate fit for the observed data was the power law of the exponential, where we see a more polynomial-like trend for smaller quantities of variants that stretches out into an exponential at larger quantities of variants. Over all three seeds, the average fit curve equation is the following:

$$t = e^{(1.181858 \cdot v^{0.360375} - 1.696283)}$$

where t represents the runtime, and v represents the number of variants.

As such it is projected that running on just 2000 variants would take just under a month. This performance renders SCARLET also functionally impossible to run on our data with any reasonable sampling. Overall, this analysis illustrates that the alternative methods that jointly consider SNV and CNA for phylogeny reconstruction are currently limited to smaller sets of mutation and are not applicable for the analysis performed in this work.

From the conceptual point of view, in both SCARLET and ConDoR the tree inference is performed simultaneously with variant placement. TreeHarmonizer, in contrast, does not infer the tree; rather, the tree is already inferred using SNVs by a method of choice (in our case ScisTree). TreeHarmonizer assigns and corrects the single nucleotide mutational placements on the given tree using the FN rate of the most optimal tree, deletions found by an SV caller (in our case Severus), and copy number alteration calls on individual (single cell derived) sublines (by Wakhan) - which are partly based on the SV calls. In summary, SNV and SV calls are made first, independently, then CNA calls, then finally, TreeHarmonizer is run afterwards. TreeHarmonizer places SNVs, SVs, and CNAs independently. While SV and CNA calls are not intrinsically independent, assigning causality and dependence is a very difficult problem that no existing method accomplishes, and remains an open problem that we are working towards solving.

Supplementary References

[1] F. Abascal, R. Acosta, N. J. Addleman, J. Adrian, V. Afzal, R. Ai, B. Aken, J. A. Akiyama, O. A. Jammal, H. Amrhein, S. M. Anderson, et al. Expanded encyclopaedias of dna elements in the human and mouse genomes. *Nature*, 583(7818):699–710, 2020.

[2] T. Ahmad, A. G. Keskus, S. Aganezov, A. Goretsky, I. Rodriguez, B. Yoo, L. A. Lansdon, E. A. Repnikova, L. Zhang, Y. Liu, et al. Wakhan: reconstruction of chromosome-scale copy number profiles of tumor genomes with long-read sequencing. *medRxiv*, pages 2025–12, 2025.

[3] R. Dreos, G. Ambrosini, R. C. Périer, and P. Bucher. The Eukaryotic Promoter Database: expansion of EPDnew and new promoter analysis tools. *Nucleic Acids Research*, 43(D1):D92–D96, 11 2014.

[4] H. Elrick, C. M. Sauer, J. Espejo Valle-Inclan, K. Trevers, M. Tanguy, S. Zumalave, S. De Noon, F. Muyas, R. Cascão, A. Afonso, et al. Savana: reliable analysis of somatic structural variants and copy number aberrations using long-read sequencing. *Nature Methods*, pages 1–11, 2025.

[5] C. ENCODE Project. An integrated encyclopedia of dna elements in the human genome. *Nature*, 489(7414):57–74, Sep 2012.

[6] A. Frankish, M. Diekhans, A.-M. Ferreira, R. Johnson, I. Jungreis, J. Loveland, J. M. Mudge, C. Sisu, J. Wright, J. Armstrong, I. Barnes, A. Berry, A. Bignell, S. Carbonell Sala, J. Chrast, F. Cunningham, T. Di Domenico, S. Donaldson, I. T. Fiddes, C. García Girón, J. M. Gonzalez, T. Grego, M. Hardy, T. Hourlier, T. Hunt, O. G. Izuogu, J. Lagarde, F. J. Martin, L. Martínez, S. Mohanan, P. Muir, F. C. P. Navarro, A. Parker, B. Pei, F. Pozo, M. Ruffier, B. M. Schmitt, E. Stapleton, M.-M. Suner, I. Sycheva, B. Uszczynska-Ratajczak, J. Xu, A. Yates, D. Zerbino, Y. Zhang, B. Aken, J. S. Choudhary, M. Gerstein, R. Guigó, T. J. P. Hubbard, M. Kellis, B. Paten, A. Reymond, M. L. Tress, and P. Flicek. Gencode reference annotation for the human and mouse genomes. *Nucleic Acids Res*, 47(D1):D766–D773, Jan 2019.

[7] X. Fu, H. Lei, Y. Tao, and R. Schwartz. Reconstructing tumor clonal lineage trees incorporating single-nucleotide variants, copy number alterations and structural variations. *Bioinformatics*, 38(Supplement_1):i125–i133, 2022.

[8] C. Gruen, H. H. Yang, A. Sassano, E. Wu, V. Gopalan, K. L. Marie, A. Castro, F. R. Mehrabadi, C. H. Wu, I. Church, et al. Melanoma clonal subline analysis uncovers heterogeneity-driven immunotherapy resistance mechanisms. *bioRxiv*, 2023.04.03.535074, 2023.

[9] M. Hirsch, S. Pal, F. R. Mehrabadi, S. Malikic, C. Gruen, A. Sassano, E. Pérez-Guijarro, G. Merlino, S. C. Sahinalp, E. K. Molloy, et al. Stochastic modeling of single-cell gene expression adaptation reveals non-genomic contribution to evolution of tumor subclones. *Cell Systems*, 16(1), 2025.

[10] B. C. Hitz, L. Jin-Wook, O. Jolanki, M. S. Kagda, K. Graham, P. Sud, I. Gabdank, J. S. Strattan, C. A. Sloan, T. Dreszer, L. D. Rowe, N. R. Podduturi, V. S. Malladi, E. T. Chan, J. M. Davidson, M. Ho, S. Miyasato, M. Simison, F. Tanaka, Y. Luo, I. Whaling, E. L. Hong, B. T. Lee, R. Sandstrom, E. Rynes, J. Nelson, A. Nishida, A. Ingersoll, M. Buckley, M. Frerker, D. S. Kim, N. Boley, D. Trout, A. Dobin, S. Rahmanian, D. Wyman, G. Balderrama-Gutierrez, F. Reese, N. C. Durand, O. Dudchenko, D. Weisz, S. S. P. Rao, A. Blackburn, D. Gkountaroulis, M. Sadr, M. Olshansky, Y. Eliaz, D. Nguyen, I. Bochkov, M. S. Shamim, R. Mahajan, E. Aiden, T. Gingeras, S. Heath, M. Hirst, W. J. Kent, A. Kundaje, A. Mortazavi, B. Wold, and J. M. Cherry. The encode uniform analysis pipelines. *bioRxiv*, Apr 2023.

[11] A. Keskus, A. Bryant, T. Ahmad, B. Yoo, S. Aganezov, A. Goretsky, A. Donmez, L. A. Lansdon, I. Rodriguez, J. Park, Y. Liu, X. Cui, J. Gardner, B. McNulty, S. Sacco, J. Shetty, Y. Zhao, B. Tran, G. Narzisi, A. Helland, D. E. Cook, P.-C. Chang, A. Kolesnikov, A. Carroll, E. K. Molloy, I. Pushel, E. Guest, T. Pastinen, K. Shafin, K. H. Miga, S. Malikic, C.-P. Day, N. Robine, C. Sahinalp, M. Dean, M. S. Farooqi, B. Paten, and M. Kolmogorov. Severus: accurate detection and characterization of somatic structural variation in tumor genomes using long reads. *medRxiv*, Mar 2024.

[12] Q. Li, A. G. Keskus, J. Wagner, M. B. Izydorczyk, W. Timp, F. J. Sedlazeck, A. P. Klein, J. M. Zook, M. Kolmogorov, and M. C. Schatz. Unraveling the hidden complexity of cancer through long-read sequencing. *Genome research*, 35(4):599–620, 2025.

[13] Y. Luo, B. C. Hitz, I. Gabdank, J. A. Hilton, M. S. Kagda, B. Lam, Z. Myers, P. Sud, J. Jou, K. Lin, U. K. Baymuradov, K. Graham, C. Litton, S. R. Miyasato, J. S. Strattan, O. Jolanki, J.-W. Lee, F. Y. Tanaka, P. Adenekan, E. O'Neill, and J. M. Cherry. New developments on the encyclopedia of dna elements (encode) data portal. *Nucleic Acids Res*, 48(D1):D882–D889, Jan 2020.

[14] F. J. Martin, M. R. Amode, A. Aneja, O. Austine-Orimoloye, A. G. Azov, I. Barnes, A. Becker, R. Bennett, A. Berry, J. Bhai, S. K. Bhurji, A. Bignell, S. Boddu, P. R. Branco Lins, L. Brooks, S. B. Ramaraju, M. Charkhchi, A. Cockburn, L. Da Rin Fiorretto, C. Davidson, K. Dodiya, S. Donaldson, B. El Houdaigui, T. El Naboulsi, R. Fatima, C. G. Giron, T. Genez, G. S. Ghattaoraya, J. G. Martinez, C. Guijarro, M. Hardy, Z. Hollis, T. Hourlier, T. Hunt, M. Kay, V. Kaykala, T. Le, D. Lemos, D. Marques-Coelho, J. C. Marugán, G. A. Merino, L. P. Mirabueno, A. Mushtaq, S. N. Hossain, D. N. Ogeh, M. P. Sakthivel, A. Parker, M. Perry, I. Piližota, I. Prosovetskaia, J. G. Pérez-Silva, A. I. A. Salam, N. Saraiva-Agostinho, H. Schuilenburg, D. Sheppard, S. Sinha, B. Sipos, W. Stark, E. Steed, R. Sukumaran, D. Sumathipala, M.-M. Suner, L. Surapaneni, K. Sutinen, M. Szpak, F. F. Tricomi, D. Urbina-Gómez, A. Veidenberg, T. A. Walsh, B. Walts, E. Wass, N. Willhoft, J. Allen, J. Alvarez-Jarreta, M. Chakiachvili, B. Flint, S. Giorgetti, L. Haggerty, G. R. Ilsley, J. E. Loveland, B. Moore, J. M. Mudge, J. Tate, D. Thybert, S. J. Trevanion, A. Winterbottom, A. Frankish, S. E. Hunt, M. Ruffier, F. Cunningham, S. Dyer, R. D. Finn, K. L. Howe, P. W. Harrison, A. D. Yates, and P. Flicek. Ensembl 2023. *Nucleic Acids Research*, 51(D1):D933–D941, 11 2022.

[15] F. R. Mehrabadi, E. S. Azer, J. D. Bridgers, E. Pérez-Guijarro, K. L. Marie, H. H. Yang, C. Gruen, C. H. Wu, W. Robinson, H. Liu, C. Kizilkale, M. C. Kelly, C. Smith, S. Chin, J. Ebersole, S. Burkett, A. Buluc, M. P. Lee, E. K. Molloy, T. M. Przytycka, G. Merlino, C.-P. Day, S. Malikić, F. Ergun, and S. C. Sahinalp. A partition function algorithm to evaluate inferred subclonal structures in single-cell sequencing data. In S. Sankararaman, editor, *Research in Computational Molecular Biology*, pages 409–413, Cham, 2025. Springer Nature Switzerland.

[16] P. Meylan, R. Dreos, G. Ambrosini, R. Groux, and P. Bucher. EPD in 2020: enhanced data visualization and extension to ncRNA promoters. *Nucleic Acids Research*, 48(D1):D65–D69, 11 2019.

[17] J. Park, D. E. Cook, P.-C. Chang, A. Kolesnikov, L. Brambrink, J. C. Mier, J. Gardner, B. McNulty, S. Sacco, A. Keskus, et al. Deepsomatic: Accurate somatic small variant discovery for multiple sequencing technologies. *bioRxiv*, 2024.

[18] S. Picelli, O. R. Faridani, Å. K. Björklund, G. Winberg, S. Sagasser, and R. Sandberg. Full-length rna-seq from single cells using smart-seq2. *Nature Protocols*, 9(1):171–181, 2014.

[19] R. Poplin, P.-C. Chang, D. Alexander, S. Schwartz, T. Colthurst, A. Ku, D. Newburger, J. Dijamco, N. Nguyen, P. T. Afshar, S. S. Gross, L. Dorfman, C. Y. McLean, and M. A. DePristo. A universal snp and small-indel variant caller using deep neural networks. *Nature Biotechnology*, 36(10):983–987, Sept. 2018.

[20] P. Priestley, J. Baber, M. P. Lolkema, N. Steeghs, E. De Bruijn, C. Shale, K. Duyvesteyn, S. Haidari, A. van Hoeck, W. Onstenk, et al. Pan-cancer whole-genome analyses of metastatic solid tumours. *Nature*, 575(7781):210–216, 2019.

[21] C. Ricketts, D. Seidman, V. Popic, F. Hormozdiari, S. Batzoglou, and I. Hajirasouliha. Meltos: multi-sample tumor phylogeny reconstruction for structural variants. *Bioinformatics*, 36(4):1082–1090, 2020.

[22] P. Sashittal, H. Zhang, C. A. Iacobuzio-Donahue, and B. J. Raphael. ConDoR: tumor phylogeny inference with a copy-number constrained mutation loss model. *Genome Biology*, 24:272, 2023.

[23] G. Satas, S. Zaccaria, G. Mon, and B. J. Raphael. Scarlet: Single-cell tumor phylogeny inference with copy-number constrained mutation losses. *Cell Systems*, 10(4):323–332.e8, 2020.

[24] M. Smolka, L. F. Paulin, C. M. Grochowski, D. W. Horner, M. Mahmoud, S. Behera, E. Kalef-Ezra, M. Gandhi, K. Hong, D. Pehlivan, et al. Detection of mosaic and population-level structural variants with sniffles2. *Nature biotechnology*, 42(10):1571–1580, 2024.

[25] E. Talevich, A. H. Shain, T. Botton, and B. C. Bastian. Cnvkit: Genome-wide copy number detection and visualization from targeted dna sequencing. *PLOS Computational Biology*, 12(4):1–18, 04 2016.

[26] E. Talevich, A. H. Shain, T. Botton, and B. C. Bastian. Cnvkit: genome-wide copy number detection and visualization from targeted dna sequencing. *PLoS computational biology*, 12(4):e1004873, 2016.

[27] G. Tang, M. Cho, and X. Wang. Oncodb: an interactive online database for analysis of gene expression and viral infection in cancer. *Nucleic Acids Research*, 50(D1):D1334–D1339, 10 2021.

[28] T. Tickle, I. Tirosh, C. Georgescu, M. Brown, and B. Hass. infercnv of the trinity ctat project.

[29] L. Zhang, H. W. Bass, J. Irianto, and X. Mallory. Scsnvcna: Integrating snvs and cnas on a phylogenetic tree from single-cell dna sequencing data. *bioRxiv*, pages 2022–08, 2022.

[30] Z. Zheng, S. Li, J. Su, A. W.-S. Leung, T.-W. Lam, and R. Luo. Symphonizing pileup and full-alignment for deep learning-based long-read variant calling. *Nature Computational Science*, 2(12):797–803, Dec. 2022.

[31] Z. Zheng, J. Su, L. Chen, Y.-L. Lee, T.-W. Lam, and R. Luo. Clairs: a deep-learning method for long-read somatic small variant calling. Aug. 2023.
