# Supplementary material for: Long-read sequencing of single cell-derived melanoma sublines reveals divergent and parallel genomic and epigenomic evolutionary trajectories
Source: Nat Commun. 2026 Jul 16;17:8740. doi: 10.1038/s41467-026-75172-9 (PMC13493970; doi:10.1038/s41467-026-75172-9)
Supplement: Supplementary file 2 [file 41467_2026_75172_MOESM2_ESM.pdf]

nature portfolio

Corresponding author(s): Mikhail Kolmogorov

Last updated by author(s): 5/12/2026

Reporting Summary

Nature Portfolio wishes to improve the reproducibility of the work that we publish. This form provides structure for consistency and transparency in reporting. For further information on Nature Portfolio policies, see our Editorial Policies and the Editorial Policy Checklist.

Statistics

For all statistical analyses, confirm that the following items are present in the figure legend, table legend, main text, or Methods section.

n/a	Confirmed	
☐	☒	The exact sample size (*n*) for each experimental group/condition, given as a discrete number and unit of measurement
☒	☐	A statement on whether measurements were taken from distinct samples or whether the same sample was measured repeatedly
☐	☒	The statistical test(s) used AND whether they are one- or two-sided<br>*Only common tests should be described solely by name; describe more complex techniques in the Methods section.*
☒	☐	A description of all covariates tested
☐	☒	A description of any assumptions or corrections, such as tests of normality and adjustment for multiple comparisons
☐	☒	A full description of the statistical parameters including central tendency (e.g. means) or other basic estimates (e.g. regression coefficient) AND variation (e.g. standard deviation) or associated estimates of uncertainty (e.g. confidence intervals)
☐	☒	For null hypothesis testing, the test statistic (e.g. *F*, *t*, *r*) with confidence intervals, effect sizes, degrees of freedom and *P* value noted<br>*Give P values as exact values whenever suitable.*
☒	☐	For Bayesian analysis, information on the choice of priors and Markov chain Monte Carlo settings
☒	☐	For hierarchical and complex designs, identification of the appropriate level for tests and full reporting of outcomes
☐	☒	Estimates of effect sizes (e.g. Cohen's *d*, Pearson's *r*), indicating how they were calculated

Our web collection on statistics for biologists contains articles on many of the points above.

Software and code

Policy information about availability of computer code

Data collection	Nanopore MinKNOW software was used for read generation: basecalling was performed with guppy v6.4.2 for the 4khz samples and with dorado v0.3.4 for the 5khz samples. Sequencing quality was accessed using samtools v1.21, seqkit v2.7.0, and pycoQC v2.5.2.
Data analysis	Read alignment was performed with minimap2 2.30. Phasing and haplotagging were performed with margin v2.3.1 and Whatshap. v2.1 Variant calling was performed with DeepVariant v1.6.1 for subline long-read and bWES data, DeepVariant v1.5.0 for subline scRNA-seq data, and with Dipcall v0.3 for the FVB assembly. Structural variant calling for sublines was performed with Severus v1.3, and that for the FVB assembly with hapdiff commit e0abbb9, freely available at: https://github.com/KolmogorovLab/hapdiff. Copy number calling was performed with Wakhan v0.1.2, freely available at: https://github.com/KolmogorovLab/Wakhan, on long-read data, with CNVkit v0.9.12 for bWES data, and with inferCNV v1.19.1 on scRNA-seq data. Phylogeny reconstruction was performed with ScisTree v1.2.0.6, freely available at: https://github.com/yufengwudcs/ScisTree. The placement of variants onto the phylogeny and subsequent regenotyping was done by TreeHarmonizer commit 3df2891, freely available at: https://github.com/KolmogorovLab/TreeHarmonizer. Mutation signature refitting was performed with SigProfileAssignment v0.1.9. Variant effect was annotated with Ensembl Variant Effect Predictor v102. Pathway enrichment analysis was performed with clusterProfilerv3.21. Structural variant annotation was performed with Padfoot commit d5237b, freely available at: https://github.com/KolmogorovLab/Padfoot. Base modification calling was performed with modbam2bed v0.10.0. Differential methylation analysis was performed with the BSmooth method integrated within the bsseq v3.21 package. Gene-level expected count values were obtained for the subline scRNA-seq data using RSEM v1.3.2. Differential expression analysis was performed with DESeq2 v1.42.1. Uniquely mapped reads were quantified with sambamba v1.0.1. Results from the pseudobulk benchmarking experiments were obtained using DeepSomatic v1.7.0, ClairS v0.3.1, DeepVariant v1.6.1, and Clair3 v1.0.10.<br><br>Other software packages involved in data analysis and visualization include: R.utils v2.12.3, dplyr v1.1.4, tidyverse v2.0.0, tidytree v0.4.6, purrr v1.0.4, tibble v3.3.0, repr v1.1.7, readxl v1.4.3, Matrix.utils v 0.9.8, TxDb.Mmusculus.UCSC.mm10.knownGene v3.10.0, org.Mm.eg.db v3.18.0,

biomaRt v2.58.2, GenomicRanges v1.54.1, GenomicFeatures v1.54.4, plyranges v1.22.0, regioneRv1.34.0, reshape2 v1.4.4, data.table v1.14.10, pracma v2.4.4, glue v1.8.0, Seurat v5.3.0, ggplot2 v3.5.2, ggtree v3.10.1, ggtreeExtra v1.12.0, ggrepel v0.9.5, ggVennDiagram v1.5.4, ggsankey v0.0.99999, ggnewscale v0.5.1, ggimage v0.3.3, ggsci v3.0.3, ggridges v0.5.5, ggpubr v0.6.0, ggtext v0.1.2, ggplotify v0.1.2, ggfortify v0.4.17, circlize v0.4.16, EnrichedHeatmap v1.32.0, ComplexHeatmap v2.18.0, RColorBrewer v1.1-3, colorspace v2.1-0, cowplot v1.1.2, magick v2.8.5, Gviz v1.46.1, rtracklayer v1.62.0, scales v1.4.0, enrichplot v1.22.0, grid v4.3.2, karyoploteR v1.28.0, VariantAnnotation v1.48.1, geomtextpath v0.1.4,

For manuscripts utilizing custom algorithms or software that are central to the research but not yet described in published literature, software must be made available to editors and reviewers. We strongly encourage code deposition in a community repository (e.g. GitHub). See the Nature Portfolio guidelines for submitting code & software for further information.

Data

Policy information about availability of data

All manuscripts must include a data availability statement. This statement should provide the following information, where applicable:

- Accession codes, unique identifiers, or web links for publicly available datasets
- A description of any restrictions on data availability
- For clinical datasets or third party data, please ensure that the statement adheres to our policy

The unaligned long-read sequencing data (.bam files) have been deposited in the NCBI / NLM Sequence Read Archive (SRA) under BioProject accession number PRJNA1307171.
Variant calls and other processed data are available in a Zenodo archive: https://doi.org/10.5281/zenodo.16883901
Orthogonal data are also available under NCBI / NLM SRA:
bWES - BioProject Accession PRJNA891351 / NCBI GEO GSE215961
scRNA - BioProject Accession PRJNA891349 / NCBI GEO GSE215959
bWTS - BioProject Accession PRJNA891350 / NCBI GEO GSE215962

Research involving human participants, their data, or biological material

Policy information about studies with human participants or human data. See also policy information about sex, gender (identity/presentation), and sexual orientation and race, ethnicity and racism.

Reporting on sex and gender	Does not apply, as the study does not involve human data.
Reporting on race, ethnicity, or other socially relevant groupings	Does not apply, as the study does not involve human data.
Population characteristics	Does not apply, as the study does not involve human data.
Recruitment	Does not apply, as the study does not involve human data.
Ethics oversight	Does not apply, as the study does not involve human data.

Note that full information on the approval of the study protocol must also be provided in the manuscript.

Field-specific reporting

Please select the one below that is the best fit for your research. If you are not sure, read the appropriate sections before making your selection.

☒ Life sciences ☐ Behavioural & social sciences ☐ Ecological, evolutionary & environmental sciences

For a reference copy of the document with all sections, see nature.com/documents/nr-reporting-summary-flat.pdf

Life sciences study design

All studies must disclose on these points even when the disclosure is negative.

Sample size	This study have sequenced all available sublines that were generated from the B2905 in a previous study, so no sample size calculation was performed. For animal study, please see the section "Animal and other research organisms".
Data exclusions	No data exclusions were made
Replication	Not applicable, as this study developed a computational method rather than statistically measured a biological phenotype. The code to replicate the results is openly available.
Randomization	Not applicable - as there were not comparison groups in this study.
Blinding	No applicable - this study did not compare biological phenotypes.

Reporting for specific materials, systems and methods

We require information from authors about some types of materials, experimental systems and methods used in many studies. Here, indicate whether each material, system or method listed is relevant to your study. If you are not sure if a list item applies to your research, read the appropriate section before selecting a response.

Materials & experimental systems

n/a	Involved in the study
☒	☐ Antibodies
☐	☒ Eukaryotic cell lines
☒	☐ Palaeontology and archaeology
☐	☒ Animals and other organisms
☒	☐ Clinical data
☒	☐ Dual use research of concern
☒	☐ Plants

Methods

n/a	Involved in the study
☒	☐ ChIP-seq
☒	☐ Flow cytometry
☒	☐ MRI-based neuroimaging

Eukaryotic cell lines

Policy information about cell lines and Sex and Gender in Research

Cell line source(s)	Sublines were previously derived from the B2905 cell line, which was generated from a M4 mouse model (a UV-irradiated male C57BL/6 mouse) in a previous study (Perez-Guijarro, 2020)
Authentication	Authentication of all cell lines was performed by HGF transgene genotyping, whole exome sequencing, Spectral karyotyping (SKY), and long-read sequencing described in this study. Cell lines were confirmed as Mycoplasma negative using a MycoAlert Mycoplasma Detection Kit (Lonza, LT07-418)
Mycoplasma contamination	All the cell lines were confirmed to be Mycoplasma-negative using a MycoAlert Detection Kit.
Commonly misidentified lines (See ICLAC register)	None

Animals and other research organisms

Policy information about studies involving animals; ARRIVE guidelines recommended for reporting animal research, and Sex and Gender in Research

Laboratory animals	C57BL/6N mouse
Wild animals	Not applicable.
Reporting on sex	6-12 weeks-old female mice were used in the cited animal study.
Field-collected samples	The animal studies that generated data used in this study were reported by Gruen et al. [23] as following. 6-12 weeks-old female C57BL/6NCr mice were supplied by Charles River facility in NCI-Frederick, with no selection on the weight. They were housed in AAALAC-accredited cage as a group of five, with ad libitum food and water, and a 12-hour light cycle. All mouse experiments were performed in accordance with Animal Study Protocols approved by the ACUC, NCI, NIH. For testing the in vivo growth kinetics of each subline, 10^6 melanoma cells from each cell line were implanted subcutaneously (s.c.) into five mice and tumor growth was monitored until endpoint. Tumor size and body weight were measured 2 times per week and the endpoint was established as the occurrence of one of the following events: (1) tumor size reached 1000 mm3; (2) tumor became ulcerated;and/or (3) the mouse showed moribund status or sickness behavior.
Ethics oversight	All the studies involving the use of animals and their tissues were performed in compliance with the guidelines of Association for Assessment and Accreditation of Laboratory Animal Care (AAALAC) and Animal Care and Use Committee (ACUC) of National Cancer Institute, NIH. NCI is accredited by the AAALAC and follows the Public Health Service Policy on the Care and Use of Laboratory Animals. All animals used in the cited study \cite{gruen2023melanoma} were cared for and used humanely according to the following policies: the US Public Health Service Policy on Humane Care and Use of Animals (2015); the Guide for the Care and Use of Laboratory Animals (2011); and the US Government Principles for Utilization and Care of Vertebrate Animals Used in Testing, Research, and Training (1985). No human sample was used in this study.

Note that full information on the approval of the study protocol must also be provided in the manuscript.

Plants

Seed stocks	Not applicable - no experiments involving plants
Novel plant genotypes	Not applicable
Authentication	Not applicable
