# Supplementary material for: Long-read sequencing of single cell-derived melanoma sublines reveals divergent and parallel genomic and epigenomic evolutionary trajectories
Source: Nat Commun. 2026 Jul 16;17:8740. doi: 10.1038/s41467-026-75172-9 (PMC13493970; doi:10.1038/s41467-026-75172-9)
Supplement: Supplementary file 3 [file 41467_2026_75172_MOESM3_ESM.pdf]

nature portfolio

# Peer Review File

# Long-read sequencing of single cell-derived melanoma sublines reveals divergent and parallel genomic and epigenomic evolutionary trajectories

Corresponding Author: Dr Mikhail Kolmogorov

**This file contains all reviewer reports in order by version, followed by all author rebuttals in order by version.**

Version 0:

Reviewer comments:

Reviewer #1

(Remarks to the Author)
In this study titled "Long-read sequencing of single cell-derived melanoma subclones reveals divergent and parallel genomic and epigenomic evolutionary trajectories", Liu and Goretsky et al. make a fascinating use of long-read sequencing technology, recapitulating the mutational information obtained from traditional Illumina sequencing while also profiling of additional mutational processes, including large structural variants and epigenomic alterations.

The study profiles subclones of B2905 murine melanoma cell line. All three Mel114433, HCmel1274, and B2905 have been invaluable resource in melanoma studies. The high mutation burden and inherent heterogeneity of the B2905 (M4) line presents a good opportunity to perform single cell resolution profiling mutational processes. I commend the authors for developing TreeHarmonizer, which enables the placement of SVs, SNVs, and CNVs within a phylogenetic tree. I look forward to seeing this tool further developed into a standalone package. Overall, I view this manuscript primarily as a methods paper that presents a computational framework for mutational profiling using long-read sequencing data along with epigenome. That said, I recommend that the authors more clearly acknowledge the limitations of deriving conclusions from a murine disease model when considering applicability to human melanoma.

However, as with any platform, there are some shortcomings that should be considered. I hope my comments and recommendation with help improve the clarity and scientific rigor of the manuscript:

• The manuscript lacks the distinction between melanoma subtypes which can be problematic. By default, the term "melanoma" refers to cutaneous melanoma, given its predominance, and presenting these mutations without that clarification risks misleading readers about their relevance in the most common melanoma subtype. I kindly ask the authors to make this distinction.

• A careful examination of the manuscript and its results provide clear insights into the mutational processes of melanoma, and I commend the authors for this achievement. The methodology is rigorous and well-executed. However, the study is highly specific to this very specific murine cell line, so caution is warranted when extrapolating these findings to human patients. I suggest that the manuscript clarify this limitation more explicitly. The mutational profiles do not match to what is reported in human melanoma, more specifically cutaneous melanoma. I have made a sincere attempt to expand on this in some of the following specific comments.

• The author mentions "Myc homolog is commonly amplified in human melanoma". However, the MYC is a proto-oncogene, and this amplification is observed in advanced/metastatic melanoma per TCGA data. It is not a driver. Also, Myc homolog is very broad term. It's almost always c-MYC.

• The authors constantly make references to human melanoma. But the melanoma subtypes are very distinct. The driver mutations and the known tumorigenesis process differ significantly. They should clarify the specific melanoma model that they are trying to replicate using the presented model.

• It is similar for MITF as well which is a more nuanced "possible" oncogene.

• I have similar concerns with the statement: "Among the clonal SNVs, we observe hotspot coding mutations, such as KRAS (G12D) and GNAQ (Q209L) that are well-known cancer drivers, and variants in SF3B1 and TFAP2A, which are frequently mutated genes in melanoma [75, 45]."

First, the citation for KRAS refers to a news article ("The KRAS crowd targets its next cancer mutations"), which is not peer-reviewed. While KRAS is a well-established oncogene in several cancers, KRAS mutations are exceedingly rare in cutaneous melanoma and are not considered hallmark drivers of this disease.

Second, the cited article "Comparative analysis of the GNAQ, GNA11, SF3B1, and EIF1AX driver mutations in melanoma and across the cancer spectrum" clearly states that GNAQ/GNA11 mutations occur in only ~0.5–1% of non-uveal melanomas and are otherwise largely specific to uveal melanoma (~80%). Similarly, SF3B1 mutations are characteristic of uveal melanoma rather than cutaneous melanoma.

Finally, TFAP2A is primarily studied as a lineage-specific transcription factor regulating melanocyte and melanoma biology, but it is not widely recognized as a gene that is "frequently mutated" in melanoma.

• Padfoot (https://github.com/KolmogorovLab/Padfoot) is not accessible. The github does not contain this tool.

• Authors write "On average, this yielded 69.77Gbp of data per subline with a mean read N50 of 29.1Kbp and an average of 82.95% of base pairs with a quality score of 20+ (Supplementary Table S1)." This statement lacks prospective. It would help if they can clarify how this QC values compares to a traditional WES run.

• In Figure 2a, compared to what is reported in melanoma, the fraction of SBS7 is only appreciable in the trunk. This makes sense since this is the key site of the UV radiation induced mutational event. However, the proportion of SBS 7 is worryingly low. In contrast, some of the other signature SBS5 higher than what is expected. SBS5 is not attributable to a mutagen and more linked to aging. As a DNA repair error induced signature, it may be linked to high proliferation. Do the authors think this due to something like genetic drift? While the authors also tried to explain the SBS 85, it is not a common signature in melanoma. Do you think it is linked to the immunogenicity of this particular murine cell line. Given the specific purpose of this line, it may explain this signature. Additionally, given the sample, did the authors attempted to generate de novo signatures and then attempt to assign to the known signatures as well as identify possibly new signatures?

• The authors provide an analysis of structural variants (SVs) identified by long-read sequencing and attempt to assess whether these SVs could have been detected using bWES or scRNA-seq by evaluating coverage at breakends. While this approach may suggest the increased resolution afforded by long-read sequencing, the claim that >86–92% of SVs would have been missed in short-read data highly overstate the limitations of conventional technologies. Coverage does not equate to detectability. It also depends on mapping, algorithm sensitivity, read pairing etc. In real experiments, including in ones published for multiple prominent studies in the field have made SV calls with far lower coverage with high sensitivity and specificity. I am also confused with the use of scRNA-seq data for this. scRNA data inherently have sparse coverage. This use overstates detectability limitations because most SVs occur in non-expressed regions. While I do not question the clear advantage of long-read sequencing for detecting large SVs, the way the results are presented gives a somewhat exaggerated impression of the deficiencies of short-read methods.

Minor comment:
• "For concision of presentation, activities for signatures SBS7a, SBS7b, SBS7c, and SBS7c were aggregated" should probably be "SBS7a, SBS7b, SBS7c, and SBS7d".

(Remarks on code availability)

Reviewer #2

(Remarks to the Author)
In this manuscript, the authors investigate the genomic and DNA methylation heterogeneity among 23 subclones derived from a mouse melanoma cell line established in a recent study, using Oxford Nanopore long-read sequencing. They further develop a computational approach to classify different types of variants along the phylogenetic branches and identify subclonal or private alterations that may contribute to the aggressive phenotypes observed in certain sublines. Overall, the study is interesting and provides valuable sequencing data that may serve as a resource for the community. However, several points require clarification and additional discussion to improve the clarity and interpretability of the work.

1.TreeHarmonizer takes a pre-constructed phylogenetic tree as input. Could the authors clarify whether the resulting tree topology may differ from the input tree? If so, how should such topological discrepancies be interpreted? Additionally, how do the authors reconcile noticeable differences in phylogenetic structures reported across different studies using the same subclones (Hirsch et al, Gruen et al and this one)?

2.Have the authors considered scenarios in which mutations occurring in regions with copy-number gain (≥3 copies) in the parental line might be lost in sublines that subsequently lose extra copies (e.g., reverting to diploid)? Such changes could remove somatic mutations without being categorized as "deletions," potentially leading to loss of heterozygosity or variant absence in those sublines.

3.Based on the description, UV exposure occurred during tumor model generation but not during the establishment of the parental and sublines. Nevertheless, SBS7-type mutations were observed on internal and terminal branches, albeit at lower fractions. Could the authors elaborate on possible biological or technical explanations for this observation?

4.Could the authors comment on the impact of the relatively lower sequencing quality of ONT on SNV detection?

5.In Figure 2a, the numbers of different variant types (SNV, SV, CNA, and DMR) are shown as colored blocks. The order of these blocks, however, varies among the trunk, internal, and terminal branches. For consistency and easier visual comparison, the order of categories should remain uniform across panels.

6.In the Abstract, the authors state that they “introduce an approach for long-read sequencing of single-cell-derived subclones.” This statement is rather vague. Please revise.

7.Please indicate the estimated ploidy of each subline. Did any sublines exhibit evidence of whole-genome doubling?

8.The sequencing depth for each subline should be provided in a supplementary table, to enable assessment of variant detection sensitivity.

9.The order of references appears very random. Please ensure that references are cited sequentially according to their first appearance in the text.

(Remarks on code availability)
The code provided appears to be specifically for the analyses presented in this manuscript. While this is acceptable if the intent is to illustrate how the analyses were performed, in this case the software constitutes a key component and is intended for public dissemination. Therefore, it would be preferable for the authors to provide a cleaner, standalone version of the code that is broadly applicable and more accessible for other researchers to use.

Reviewer #3

(Remarks to the Author)

The manuscript proposes novel insights into tumor evolution using long-read sequencing of single cell-derived melanoma subclones. The authors developed a new computational framework to integrate genomic and epigenomic (SNVs, SVs and DMRs ) to reveal evolutionary trajectories. The manuscript is well-organized and the project is solid. However, here are some major concerns:
1. Nanopore long-read sequencing generally presents lower sequencing accuracy when compared with short-read technology. How did the authors specifically control the false discovery of SVs and SNVs?
2. In Table S1, can the authors add in a depth calculation (such as 10X or 50X)? ‘we removed SNVs with low coverage’: Dose it refer to the low-coverage SNVs per subclone or across all the 23-subclones? Is it possible that a variant (SNV, SV or DMR) is positive for one subclone but negative for the others, just caused by the uneven sequencing depth across the 23 samples? In Table S1, the lowest # Reads is 1.8M, while the highest is 10.8M. Will different depths influence the evolutionary trajectory results? Low depth for a certain loci or region will also result in false positives or false negatives? How did the author balance these?
3. Figure 2 & 3: Only chr1-chr19 were analyzed. Why were chrX and chrY excluded from the study?
4. Does the tool TreeHarmonizer treat all the SNVs and SVs independently, or will it consider the association of neighboring variants, such as Linkage Disequilibrium?
5. Does the algorithm consider the genomic location of the variants, such as repetitive regions (which are more challenging in variant detection)? Or their location at the exon, intron or intergenic regions?
6. “Indeed, TreeHarmonizer variant placement led to a significant increase (one-tailed paired-sample t-test $t(26) = 11.99$, $p = 2.133e-12$) in their partition function values, confirming accurate variant placement (Figure 7g.).” Does the data follow normal distribution so that the t-test can be applied?
7. There is a lack of systematic comparison of the tool TreeHarmonizer with the existing tools. Please provide more details on the algorithm novelty of the proposed tool, as well as performance comparison (accuracy, computational efficiency, as well as other evaluation metrics).
8. On the TreeHarmonizer GitHub README file, detailed installation pipeline is available. However, there is no instruction on how to run the tool. Especially in the ‘Quick Run’ section, no detailed instruction on how to run the demo data and how to understand the output file.
9. Besides mouse, is the tool and analysis pipeline generalizable to other species (such as human or rat)?
10. Have the key SNVs, SVs or DMRs detected from the long-read data been experimentally validated?
11. Please discuss and compare long-read WGS and scRNA-seq in revealing the heterogeneity within the sublines.
12. Has the study specifically considered the Haplotype phasing?

Minor:
1. Please provide more biological interpretation of the defined clonal, subclone and private variants.
2. Please well define placed variants vs unplaced variants.
3. Figure 3: Please well define different types of SVs (BND, DEL, DUP, INS and INV) in Fig 3b and 3c.

(Remarks on code availability)
On the TreeHarmonizer GitHub README file, detailed installation pipeline is available. However, there is no instruction on how to run the tool. Especially in the ‘Quick Run’ section, no detailed instruction on how to run the demo data and how to understand the output file.

Reviewer #4

(Remarks to the Author)

This manuscript presents a Nanopore dataset of single-cell-derived tumor sublines, and the pipeline of variant calling (including CNAs, SVs, and CNAs) and phylogeny reconstruction. Additionally, a new method TreeHarmonizer is developed to place all types of variants on a phylogenetic tree. Mutation signatures, relative evolution rates, and methylation states are analyzed along the phylogenetic tree. This dataset is a valuable resource to study evolution. However, I have the following concerns regarding the computational analysis.

Major comments.
1. Previous methods have been developed to reconstruct phylogeny using SNV, SV, and CNA (e.g., TUSV-ext reconstructs phylogenetic using all three modalities, SCARLET and Condor use SNVs and CNAs). Additionally, SCsnvcna places SVs on an existing phylogenetic tree with CNAs information; Meltos places SVs on a SNV-based phylogenetic tree and further refines the tree. Despite of these methods that can achieve similar results, the performance of TreeHarmonizer is not evaluated or benchmarked. TUSV-ext: https://academic.oup.com/bioinformatics/article/38/Supplement_1/i125/6617488 SCARLET: https://www.sciencedirect.com/science/article/pii/S2405471220301150 Condor: https://genomebiology.biomedcentral.com/articles/10.1186/s13059-023-03106-5 SCsnvcna: https://pmc.ncbi.nlm.nih.gov/articles/PMC10760445/ Meltos: https://academic.oup.com/bioinformatics/article/36/4/1082/5581399
2. I'm confused about the following details of TreeHarmonizer method.
(1). The order of placing CNAs, SVs, and SNVs matters, as the reasoning of SCARLET and Condor papers. What's the order of placing these variants in TreeHarmonizer?
(2). After simplifying the CNAs into gains and losses, does TreeHarmonizer consider the potential overlapping with SVs?
(3). The assignment of overlapping internals and merging of CNA placement is very confusing. What are the underlying assumptions/justifications underlying these options? Are the refinement of a set of overlapping intervals of CNAs viewed as independent CNA events?
3. The last section indicates the SNV, SV, and CNA calls across the 23 sublines are curated and can be used to benchmark other tools. However, these SNV, SV, and CNA calls are each generated from a single computational tool without validating using either orthogonal sequencing measurement or using the consensus across multiple computational methods.
4. In particular, the rate of SNV acquisition is compared to that of SV. This claim requires accurate placement of SVs along the tree. I wonder (1) if applying other SV callers, e.g., Sniffle2, whether the observation still holds, and (2) if focusing the total number of SNVs and SVs of each subline and ignoring the tree structure, whether the observation still holds.
5. Along the same line, variants inferred from Nanopore sequencing are compared against bWES and scRNA-seq, showing bWES and scRNA-seq miss many calls due to the limitation of sequencing regions. But if focusing on variants that span gene regions (including CNAs, SNVs, and SVs whose breakpoints locate within exons), they should be supported by bWES and potentially also scRNA-seq if involved genes have sufficient expression. I wonder whether this can be used to verify the reliability of Nanopore variant calling.
6. Complex SVs are identified by grouping breakend junctions into clusters by Severus. Each complex SV are considered to be one complex event happening at a single time point, hence only involve SVs that are labeled at a single tree branch, while mixing SVs from multiple branches violates the concepts of complex SVs. Does Severus consider the information of phylogenetic tree and SV labeling of tree branches? It's not clear how this approach avoids mixing SVs from multiple branches.
7. Fig 5a shows the number of differential methylation regions between two clades of a subtree and the other clades. However, two clades as a group likely doesn't have enough power. I suggest enlarging the number of clades in a group to verify the observation.
8. Example DMRs at certain branches are shown in Fig 5. But as there are many branches in the tree, it will be more general to show the number of cancer-associated DMRs (e.g. from OncoDB or other databases) at each branch, to evaluate whether the various aggressiveness of subclones can be generally attributed to the cancer-associated DMRs at each sublineage.
9. A trajectory is a linear sequence of element. So how is "evolutionary trajectory" defined from a phylogenetic tree? From any ordered tree traversal or by ranking some tree distances?

Minor comments:
- The meaning of "subclone" is used differently, sometimes referring to the 4 sub lineages, and sometimes referring to the 23 sublimes.
- Page 7 has unbalanced parenthesis in sentence "We identified 65 such clusters involving a total of 298 SVs: of which four were composed of clonal SVs 3e.)"
- Maybe I missed it, but Fig 5d doesn't seem to be referred to in the main text.
- It seems that Venn diagrams are more suitable for the information in Fig 7b, as used in Fig 7f.

(Remarks on code availability)
The installation and running of the SV and SNV notebooks are smooth. But the path to the CNA notebook doesn't match the example data directory. And I would appreciate more documentation on the output specification.

Version 1:

Reviewer comments:

Reviewer #1

(Remarks to the Author)
The authors have thoroughly revised the manuscript and have carefully integrated my comments while addressing my concerns. I appreciate that the authors have appropriately included the limitations of the study. My initial concern was the emphasis on certain mutations and structural variants observed in the mouse model that, while highly relevant and important drivers in the animal system, are extremely rare in the clinical presentation of human skin cancer. In addition, the genomic landscapes of melanoma subtypes are distinct and therefore should not be combined into a single group. The overlap in mutational and structural profiles required careful and appropriate consideration. I also acknowledge that Padfoot is now accessible. I appreciate both the major

and minor revisions, and I believe this manuscript will serve as a valuable resource for the cancer research community. Congratulations to the authors.

(Remarks on code availability)

Reviewer #2

(Remarks to the Author)
The authors have addressed my previous comments.

(Remarks on code availability)
The standalone software looks good. The installation worked and a README file is available.

Reviewer #3

(Remarks to the Author)
Thanks for the great revision work! The authors have addressed most of my concerns.

However, when I tried to test the tool TreeHarmonizer, I encountered some issues.

(Remarks on code availability)
[1]

$ git clone git@github.com:KolmogorovLab/TreeHarmonizer.git
Cloning into 'TreeHarmonizer'...
@@@@@@@@@@@@@@@@@@@@@@@@@@@@@@@@@@@@@@@@@@@@@@@@@@@@@@@@@@@
@ WARNING: REMOTE HOST IDENTIFICATION HAS CHANGED! @
@@@@@@@@@@@@@@@@@@@@@@@@@@@@@@@@@@@@@@@@@@@@@@@@@@@@@@@@@@@
IT IS POSSIBLE THAT SOMEONE IS DOING SOMETHING NASTY!
Someone could be eavesdropping on you right now (man-in-the-middle attack)!
It is also possible that a host key has just been changed.
The fingerprint for the RSA key sent by the remote host is
SHA256:uNiVztksCsDhcc0u9e8BujQXVUpKZIDTMczCvj3tD2s.
Please contact your system administrator.
Add correct host key in /ihome/sliu/shl96/.ssh/known_hosts to get rid of this message.
Offending RSA key in /ihome/sliu/shl96/.ssh/known_hosts:2
Host key for github.com has changed and you have requested strict checking.
Host key verification failed.
fatal: Could not read from remote repository.

Please make sure you have the correct access rights
and the repository exists.

[2]
Alternatively, I downloaded the zipped package from GitHub and unzipped it.
I installed the package via Conda, but I still need to manually install the three packages (pandas, ete3, and intervaltree). The README files sound like the 'Via Conda' is recommended, and the manual installation is an alternative. But actually we need to install both.

Reviewer #4

(Remarks to the Author)
The revised manuscript addressed most of my previous concerns. But I still recommend improving the clarity of the description of results and methods. Specifically,

1. Fig 2a is a complicated panel, and including the explanation of each part (rectangles, pie charts) in figure legend helps with the understanding of it. Additionally, I couldn't find which part corresponds to the dN/dS color bar.
2. I couldn't find summary statistics about number of placed and unplaced SNVs and CNAs, but only SVs. But they are important statistics to learn about performances and expectations of TreeHarmonizer.
3. From TreeHarmonizer description, it's not very clear what are the exact criteria for placing a variant or leaving it as unplaced. My guess is leaving a variant unplaced is determined by the clade size and FN formula, but it benefits from explicitly state the criteria for a variant being unplaced.
4. The explanation of "trajectory" helps, however, the language is not precise enough, and multiple descriptions are used in different places. E.g., "a path from phylogeny root to the clade leaves", "phylogenetic order". In fact, the first description is not precise as one path from root to leaves include a root node, multiple unmeasured intermediate nodes, and a single leaf node, which doesn't give the order of leaf nodes and makes the description inaccurate.

5. In TreeHarmonizer description, what's the difference between "a deletion set" and "a loss set" in the inputs?
6. TreeHarmonizer requires "an estimated variant call FP rate" as input, but is it used in later algorithm quantitatively? I only see the assumption "Given the low a priori FP rate (0.001%)", but what if it is not low enough?
7. Although the numbers of placed CNAs are shown in Fig 2a, the example CNAs in Fig 4 don't include the information about where they are placed. Given the complexity of interval overlapping among sublines, it's worth marking the overlapping intervals (O_{pr1, pr2}) and union of sublines (S_{pr1,pr2}) in the examples, as well as which branch they are placed in the tree.

(Remarks on code availability)
This version of code has a much better README.

Version 2:

Reviewer comments:

Reviewer #3

(Remarks to the Author)
I would greatly appreciate the authors' thorough revisions. The GitHub tool has been significantly improved, and I was able to successfully install the software via Conda and run the demo data. Overall, I recommend the manuscript for publication.

(Remarks on code availability)
The GitHub tool has been significantly improved, and I was able to successfully install the software via Conda and run the demo data.

Reviewer #4

(Remarks to the Author)
The revised manuscript addressed all of questions, and it reads clearly now!

(Remarks on code availability)
The github has good documentation and I don't have further comments.

**Open Access** This Peer Review File is licensed under a Creative Commons Attribution 4.0 International License, which permits use, sharing, adaptation, distribution and reproduction in any medium or format, as long as you give appropriate credit to the original author(s) and the source, provide a link to the Creative Commons license, and indicate if changes were made.
In cases where reviewers are anonymous, credit should be given to 'Anonymous Referee' and the source.
The images or other third party material in this Peer Review File are included in the article's Creative Commons license, unless indicated otherwise in a credit line to the material. If material is not included in the article's Creative Commons license and your intended use is not permitted by statutory regulation or exceeds the permitted use, you will need to obtain permission directly from the copyright holder.
To view a copy of this license, visit https://creativecommons.org/licenses/by/4.0/

## Reviewer #1

In this study titled "Long-read sequencing of single cell-derived melanoma subclones reveals divergent and parallel genomic and epigenomic evolutionary trajectories", Liu and Goretsky et al. make a fascinating use of long-read sequencing technology, recapitulating the mutational information obtained from traditional Illumina sequencing while also profiling of additional mutational processes, including large structural variants and epigenomic alterations.

The study profiles subclones of B2905 murine melanoma cell line. All three, Mel114433, HCmel1274, and B2905, have been invaluable resources in melanoma studies. The high mutation burden and inherent heterogeneity of the B2905 (M4) line presents a good opportunity to perform single cell resolution profiling mutational processes. I commend the authors for developing TreeHarmonizer, which enables the placement of SVs, SNVs, and CNVs within a phylogenetic tree. I look forward to seeing this tool further developed into a standalone package. Overall, I view this manuscript primarily as a methods paper that presents a computational framework for mutational profiling using long-read sequencing data along with epigenome. That said, I recommend that the authors more clearly acknowledge the limitations of deriving conclusions from a murine disease model when considering applicability to human melanoma.

However, as with any platform, there are some shortcomings that should be considered. I hope my comments and recommendation with help improve the clarity and scientific rigor of the manuscript:

We thank the reviewer for their supportive comments. We overall agree that since the current study analyzed only a single murine melanoma model, the results should be projected to human melanoma tumorigenesis with caution. We thus followed all the reviewer's suggestions and revised the text accordingly. Please find our detailed responses below.

In addition, for this revision we have substantially rewritten the TreeHarmonizer code and manual to make it applicable to various other cancer types and datasets. As a part of a separate study, we are applying TreeHarnomizer to single-cell derived cell lines of a human pancreatic cancer, as a part of an ongoing collaboration with the Genome in a Bottle group aiming to create a benchmark for cancer genomics (https://www.nist.gov/programs-projects/cancer-genome-bottle).

Major Comments

1. The manuscript lacks the distinction between melanoma subtypes which can be problematic. By default, the term "melanoma" refers to cutaneous melanoma, given its predominance, and presenting these mutations without that clarification risks misleading readers about their relevance in the most common melanoma subtype. I kindly ask the authors to make this distinction.

   We thank the reviewer for pointing out this important distinction of melanoma subtype. In this study, we employed a mouse model previously characterized as a UV-induced cutaneous melanoma (*Nature* (2001) 413:271–272, *Nature Med.* (2020) 26:781-791). As can be seen in our responses to the reviewer's Points 2, 4 and 6 below, we have now updated and clarified the model characterization/description through the text. Please also see our response to the reviewer's Point 4 for a related revision in the Introduction and Discussion sections.

2. A careful examination of the manuscript and its results provide clear insights into the mutational processes of melanoma, and I commend the authors for this achievement. The methodology is rigorous and well-executed. However, the study is highly specific to this very specific murine cell line, so caution is warranted when extrapolating these findings to human patients. I suggest that the manuscript clarify this limitation more explicitly. The mutational profiles do not match to what is reported in human melanoma, more specifically cutaneous melanoma. I have made a sincere attempt to expand on this in some of the following specific comments.

   We agree with the reviewer that the biological results in our work should be extrapolated with caution to human cutaneous melanoma because of (i) limitation of using a single model and (ii) relatively rare driver genes profile.

   In the M4 model, hotspot KRAS mutations and other putative drivers (such as GNAQ, SF3B1, etc.) spontaneously occurred during the melanomagenesis following UV irradiation. In the paper that introduced the M4 model (Nature Med. (2020) 26:781-791), the mutation profiles of the mouse melanomas of this model were compared to The Cancer Genome Atlas (TCGA) skin cutaneous melanoma (SKCM) dataset through hierarchical clustering, and accordingly characterized as RAS-mutant cutaneous melanomas. Note that genomic studies of human cutaneous melanoma commonly characterize those carrying NRAS, HRAS, and KRAS mutations as RAS-mutated melanomas (please see the table below and response to Point 4 and 6). Nevertheless, we agree that the KRAS mutations are observed infrequently in UV-induced human cutaneous melanoma, and acknowledge that this model represents a relatively small proportion of cutaneous melanoma patients (who are understudied), therefore the results provide more a conceptual understanding of the evolution of genetic and genomic alterations beyond specific mutations. We have thus revised our manuscript to address the limitation and scope of this study as requested by the reviewer; please also see our responses to the reviewer's Points 4 and 6 for additional details and how we revised our paper accordingly.

   The table below is from: "Molecular characterisation of cutaneous melanoma: creating a framework for targeted and immune therapies." (Br J Cancer. 2016 Jun 23;115(2):145–155). https://www.nature.com/articles/bjc2016195

**Table 1.** Implications for clinical management of melanoma patients modified from TCGA comprehensive analysis

| Genomic Subtypes | BRAF | RAS | NF1 | Triple wild-type |
|---|---|---|---|---|
| • MAPK pathway | • BRAF V600 (E/K/R), K601E | • (N/H/K) RAS G12, G13, Q61 | • NF1 LoF mut | • KIT COSMIC mut/amp, PDGFRa amp, KDR (VEGFR2) amp |
| | | | **(BRAF non-V600 mut) | ○ rare COSMIC GNA11 mut, GNAQ mut |
| #Cell cycle pathway | CDKN2A mut/del/hyper-meth (~60%) | CDKN2A mut/del/hyper-meth (~70 %) | CDKN2A mut/del/hyper-meth (~70%) | CDKN2A mut/del/hyper-meth (~40%) |
| | CCND1 amp (~3%) CDK4 amp (3%) / # (rare CDK4 COSMIC mutations) | CCND1 amp (8%) # CDK4 amp (7%) / # (rare CDK4 COSMIC mutations) | # CCDK4 amp (4%) | CCND1 amp (11%) # CDK4 amp (~15%) |
| | RB1 mut (2%) | RB1 mut (8%) | RB1 mut (11%) | |
| *DNA Damage Response & Cell death pathways | TP53 mut (~10%) Note: ~ 90% wild-type | TP53 mut (20%) | TP53 mut (~30%) | TP53 mut (7%) |
| | | | | *BCL2 upregulation |
| | *MDM2 amplification (1%) | *MDM2 amplification (3%) | *MDM2 amplification (4%) | *MDM2 amplification (~15%) |
| †PI3K/Akt pathway | †PTEN mut/del (17%) | †PTEN mut/del (8%) | †PTEN mut/del (7%) | †PTEN mut/del (7%) |
| | †AKT3 overexpression (<10%) | †AKT3 overexpression (~40%) | †AKT3 overexpression (~30%) | †AKT3 overexpression (~20%) |
| | †(rare AKT1/3 and PIK3CA COSMIC mut) | †(rare AKT1/3 and PIK3CA COSMIC mut) | | |
| ‡Epigenetics | ‡IDH1 mut (rare EZH2 COSMIC mut) | ‡IDH1 mut (rare EZH2 COSMIC mut) | ‡IDH1 mut (rare EZH2 COSMIC mut) | ‡IDH1 mut (rare EZH2 COSMIC mut) |
| | ‡ARID2 mut (~15%) | ‡ARID2 mut (~15%) | ‡ARID2 mut (~30%) | |
| Telomerase pathway | Promoter mut (~75%) | Promoter mut (~70%) | Promoter mut (~85%) | Promoter mut (<10%) |
| | | | | TERT amp (~15%) |
| Highlighted additional pathways | PD-L1 amp, MITF amp, PPP6C mut (7%) | PPP6C mut (~14%) | | |
| Beta-catenin | CTNNB1 T41, S45 mut (~0.5%) | CTNNB1 G34 mut (~0.5%) | CTNNB1 D32,G34 mut (~0.5%) | CTNNB1 T41, S45 mut (~1%) |
| ^High Immune infiltration (pathology) | ~30% | ~25% | ~25% | ~40% |
| UV signature | ~90% | ~95% | ~95% | ~30% |
| Mutational burden | 12 mut/mb | 17 mut/mb | 39 mut/mb | 9.2 mut/Mb |
| Class 1: Clinically Actionable | •MEK inhibitors (e.g. trametinib, cobimetinib) | | | • C-KIT inhibitors (e.g imatinib, dasatinib, nilotinib, sunitinib) |
| | •BRAF inhibitors (e.g. dabrafenib, vemurafenib) | | | ○ PKC inhibitors (AEB071) |
| | Immunotherapies [e.g. mAb against immune checkpoint proteins (ipilimumab, nivolumab, pembrolizumab), high dose bolus IL-2, Interferon-α2b] | | | |
| Class 2: Translationally Actionable | | | •MEK inhibitors (e.g. trametinib, cobimetinib) | |
| | •ERK inhibitors | | | |
| | *MDM2/p53 interaction inhibitors (e.g. AMG232) | | | *MDM2/p53 interaction inhibitors (e.g AMG232) |
| | #CDK inhibitors (e.g. palbociclib) | | | |
| | pan-RAF inhibitor ( e.g. LY3009120) | | | |
| | ‡IDH1 inhibitors (e.g. AG-120, IDH305) | | | |
| | ‡EZH2 inhibitors (e.g. GSK126, EPZ005678, EI1) | | | |
| | †PI3K/mTOR inhibitors (e.g. MLN1117, OSI-027) | | | |
| | †Akt inhibitor (e.g. GSK2141795) | | | |
| Class 3: Pre-clinical | (PPP6C) Aurora kinase inhibitors (e.g. MLN8237) | | | |
| | Mutant RAF dimer inhibitors (e.g BGB659) | | | |
| | ‡ARID2 Chromatin remodelers (synthetic lethality) | | | *(BCL2) BH3 mimemitcs |

Abbreviations: LoF = loss-of-function; GoF = gain-of-function; Mb = Megabase; mut = mutation; TCGA = The Cancer Genome Atlas. Canonical pathways and frequency of significant genetic alterations in *BRAF*, *RAS* (*N-*, *H-* and *K-*), *NF1* and *Triple wild-type* melanomas are given. LoF and GoF mutations are indicated in blue and red panels, respectively. Potential predictive biomarkers are represented with symbols (•, #, *, †, ‡, #). Class 1: clinically actionable alterations that impact point-of-care decisions; Class 2: translationally actionable alterations that are deemed likely to be clinically relevant but more evidence is required to support use in point-of-care decision making; and Class 3: alterations that have biological importance based on preclinical studies, but clinical relevance is unclear. Modified from latest cutaneous melanoma TCGA study.

3. The author mentions “Myc homolog is commonly amplified in human melanoma”. However, the MYC is a proto-oncogene, and this amplification is observed in advanced/metastatic melanoma per TCGA data. It is not a driver. Also, Myc homolog is very broad term. It’s almost always c-MYC.

   We thank the reviewer for the correction. We agree that “homolog” is a misleading term: the intention behind its use was to highlight homology between human and mouse genes, but it was confusing in the context of the Myc gene family. We have now changed the term “Myc homolog” to “Myc” (gene symbol of c-Myc).

   The role of the MYC amplification: in cutaneous melanoma the primary driver alterations are in BRAF, RAS, NF1, etc., and MYC activation most commonly acts as a cooperating event, typically via copy-number gain/overexpression rather than recurrent hotspot mutation *(Nature Commun. (2024). 15:2025)*. Accordingly, we have revised our manuscript and no longer use the term “driver” associated with Myc; rather, we now highlight the fact that Myc is typically amplified in advanced cancers. Please see our revisions below in red:

   *“For example, parallel amplification of Myc was recurrently present in a majority of sublines (Figure 4, Supplementary Figure S15); likewise, the Myc proto-oncogene is commonly amplified in human cutaneous melanoma [Am J Surg Pathol (2012) 36(2):253–264]. In C13, Myc is duplicated via a focal SV spanning merely 1.6Kbp, likely reflecting targeted selection.”.*

4. The authors constantly make references to human melanoma. But the melanoma subtypes are very distinct. The driver mutations and the known tumorigenesis process differ significantly. They should clarify the specific melanoma model that they are trying to replicate using the presented model.

We again thank the reviewer for bringing up this important issue. As described in our response to the reviewer's earlier Point 2, the mouse melanoma that generated the B2905 cell line has been previously characterized as RAS-mutant cutaneous melanoma (Nature Med. (2020) 26:781-791); this characterization includes NRAS-, KRAS- and HRAS-mutated melanoma (referred as mutant N/H/K RAS) according to several studies of cutaneous melanoma (Cell (2015) 161:1681-1696; *Br. J. Cancer* (2016) 115:145–155; *Eur. J. Surg. Oncol.* (2017) 43: 581-593) based on hierarchical clustering of exonic mutations. In addition, the B2905 cell line shares other features of RAS-mutated melanoma, including high mutation burden and UV signature levels (*Nature Med.* (2020) 26:781-791).

In response to the reviewer's comments, we revised some of the statements in the paper to clarify the categorization of the model we used in the study, its limitations and scope with respect to the melanoma evolution it can model. Please see our revisions below in red.

*Introduction, fifth paragraph:*
*"... each derived from a single cell of the B2905 cell line [84], which was generated from a UV-induced cutaneous melanoma in a Hgf-transgenic mouse [Nature (2001) 413:271–272]. It carries a hot-spot Kras mutation and therefore was characterized [Nature Med. (2020) 26:781-791] as a model of UV-associated RAS-mutated cutaneous melanoma [Cell (2015) 161: 1681-1696; Br. J. Cancer (2016) 115: 145–155]."*

*Introduction, sixth paragraph:*
*"... linking epigenetic changes with gradual phenotypic differentiation in this experimental system. These results provided several possible mechanisms to drive subclonal evolution in UV-induced cutaneous melanoma for further validation in human melanoma."*

*Discussion, last (fifth) paragraph:*
*"Overall, our study highlights the advantage of long-read sequencing in resolving the the complete variational landscape of tumors, potentially benefiting precision oncology studies, as for various cancers it has been shown that variants other than SNVs, including SVs and DMRs, are important biomarkers for prognosis or therapeutic response (NPJ Breast Cancer (2024), 10:64, J. Immunother. Cancer (2021), 9(7):e002226). Since these results were based on a specific mouse model of RAS-mutated cutaneous melanoma, carrying a driver mutation in Kras (G12D) and other putative drivers like Gnaq (Q209L) that occur infrequently in human counterparts, it requires caution to analyze the representativeness of the identified mechanisms. However, our analytical framework is model agnostic and can have broader applications, and the results have important biological implications. For example, our analytical framework, including the TreeHarmonizer tool, could be adopted to multi-site or longitudinal human tumor datasets. Tracing the variants at different genomic, epigenetic, and transcriptional levels and integration of these data can expand the scope of evolutionary modeling of cancer beyond the classic viewpoint focusing on coding regions and/or small variants in a snapshot manner, leading to testable hypotheses to gain mechanistic insights, which in turn may help develop therapeutic strategies. Finally, by publicly releasing all sequencing data and curated variant call sets, we provide a unique benchmarking resource to accelerate the development and validation of new cancer genomics tools."*

5. It is similar for MITF as well which is a more nuanced "possible" oncogene.

While we had originally followed the COSMIC Gene Census, which characterizes *MITF* as oncogene, we agree with the reviewer that *MITF*'s oncogenic function is conditional (*Trends Mol Med* (2006) 12: 406-414). *MITF* is widely regarded as a lineage-survival oncogene in melanoma via copy number gains and overexpression. *MITF* is amplified in ~10% of primary and ~15–20% of metastatic cutaneous cases, for which higher copy number is linked to worse survival. Forcing *MITF* expression cooperates with *BRAF*(V600E) to transform primary human melanocytes; conversely, reducing *MITF* activity compromises melanoma cell survival (*Nature* (2005) 436:117-22). We have thus revised the associated statement in page 9, line 7 accordingly. Please see our revisions below in red.

*"These CNAs affected key ONCs, such as Met, Braf, and clonally mutated Kras (Figure 4, Supplementary Figure S12). Other putative targets included Mitf, a lineage-survival oncogene [Trends Mol Med* (2006) 12: 406-414*] via copy number gains and overexpression in ~10% of primary and ~15–20% of metastatic cutaneous cases [Nature* (2005) 436:117-22*]. "*

6. I have similar concerns with the statement: "Among the clonal SNVs, we observe hotspot coding mutations, such as KRAS (G12D) and GNAQ (Q209L) that are well-known cancer drivers, and variants in SF3B1 and TFAP2A, which are frequently mutated genes in melanoma [75, 45]."

   (i) First, the citation for KRAS refers to a news article ("The KRAS crowd targets its next cancer mutations"), which is not peer-reviewed. While KRAS is a well-established oncogene in several cancers, KRAS mutations are exceedingly rare in cutaneous melanoma and are not considered hallmark drivers of this disease.

   (ii) Second, the cited article "Comparative analysis of the GNAQ, GNA11, SF3B1, and EIF1AX driver mutations in melanoma and across the cancer spectrum" clearly states that GNAQ/GNA11 mutations occur in only ~0.5–1% of non-uveal melanomas and are otherwise largely specific to uveal melanoma (~80%). Similarly, SF3B1 mutations are characteristic of uveal melanoma rather than cutaneous melanoma.

   (iii) Finally, TFAP2A is primarily studied as a lineage-specific transcription factor regulating melanocyte and melanoma biology, but it is not widely recognized as a gene that is "frequently mutated" in melanoma.

   We surveyed the TCGA cutaneous melanoma dataset and accordingly agree with the reviewer that:
   (1) KRAS(G12) and GNAQ(Q209L) mutations are infrequent in human cutaneous melanoma (1.5-5% in TCGA human cutaneous melanoma [cBioPortal]; > 1.9% each in Cell (2015) 161: 1681-1696).
   (2) Gnaq mutations, which were also observed in human cutaneous melanoma (*Nature* 545.7653 (2017): 175-180), may not be distinct/independent drivers since they co-occur with other mutations;
   (3) SF3B1 mutation in this model is not in the hotspot region (around R625), and TFAP2A has a low mutation rate in human cutaneous melanoma - therefore, both of these could be background mutations.

   Accordingly, we revised the statement in page 6, sixth paragraph. Please see our revisions below in red.

   *"Among the clonal SNVs, we observe spontaneous hotspot coding mutations, such as Kras (G12D) and Gnaq (Q209L). Both have been shown to drive melanomagenesis in mouse models [Pigment Cell Melanoma Res (2007) 20:485-497; Pigment Cell Melanoma Res (2010) 23:853–859], though they have been observed only in a relatively small subset of human cutaneous melanoma [Cell (2015) 161: 1681-1696; Am J Dermatopathol (2015) 37:389-397; N Engl J Med (2015) 373:1926-36]. Other variants were either not in hotspot regions or rarely mutated in human cutaneous melanoma."*

7. Padfoot (https://github.com/KolmogorovLab/Padfoot) is not accessible. The github does not contain this tool.

   We thank the reviewer for pointing this out, and apologize for the oversight. We realized that the repository was in private mode, and have now switched it to be publicly accessible.

8. Authors write "On average, this yielded 69.77Gbp of data per subline with a mean read N50 of 29.1Kbp and an average of 82.95% of base pairs with a quality score of 20+ (Supplementary Table S1)." This statement lacks prospective. It would help if they can clarify how this QC values compares to a traditional WES run.

   We thank the reviewer for pointing out this lack of clarity. We have added a new supplementary table S2 for WES statistics for comparison with long-read summary statistics table S1. We have accordingly revised the statement above, adding WES information. Please see our revisions below in red.

   *"On average, this yielded 69.77Gbp of data per subline with a mean read N50 of 29.1Kbp and an average of 82.95% of base pairs with a quality score of 20+ (Supplementary Table S1). For comparison, WES runs on the same sublines yields an average length of 123 bp per read, with a 99.36% of base pairs with a quality score of 20+ (Supplementary Table S2). We note that most of the ONT base errors are indels, and SNP calling quality is similar for ONT and Illumina* [*Nature Methods (2023) 20:1483–1492*]."

| Subline | # Reads | Total Length | Max Length | Mean Length | Avg Depth | Q20(%) | Q30(%) | AvgQual |
|---|---|---|---|---|---|---|---|---|
| C1 | 214,587,122 | 26,514,866,642 | 151 | 123.6 | 151.87 | 99.39 | 98.08 | 32.47 |
| C2 | 177,351,022 | 21,885,891,023 | 151 | 123.4 | 127.55 | 99.36 | 98.01 | 32.30 |
| C3 | 236,326,610 | 29,241,058,731 | 151 | 123.7 | 160.17 | 99.41 | 98.13 | 32.58 |
| C4 | 175,342,036 | 21,589,058,024 | 151 | 123.1 | 129.77 | 99.38 | 98.05 | 32.42 |
| C5 | 205,967,396 | 25,398,503,020 | 151 | 123.3 | 143.05 | 99.37 | 98.02 | 32.35 |
| C6 | 214,023,384 | 26,646,667,373 | 151 | 124.5 | 143.36 | 99.40 | 98.11 | 32.50 |
| C7 | 223,313,600 | 27,574,075,712 | 151 | 123.5 | 157.99 | 99.40 | 98.07 | 32.49 |
| C8 | 208,777,688 | 25,667,257,080 | 151 | 122.9 | 149.20 | 99.38 | 98.08 | 32.39 |
| C9 | 235,523,830 | 28,811,943,724 | 151 | 122.3 | 169.79 | 99.34 | 97.99 | 32.19 |
| C10 | 216,816,122 | 26,730,779,028 | 151 | 123.3 | 149.54 | 99.40 | 98.14 | 32.53 |
| C11 | 198,685,460 | 23,999,015,045 | 151 | 120.8 | 141.63 | 99.31 | 97.89 | 32.03 |
| C12 | 190,688,314 | 23,302,161,699 | 151 | 122.2 | 133.15 | 99.33 | 97.97 | 32.14 |
| C13 | 237,138,312 | 29,056,964,816 | 151 | 122.5 | 155.32 | 99.39 | 98.11 | 32.47 |
| C14 | 172,757,040 | 21,201,029,234 | 151 | 122.7 | 125.52 | 99.36 | 98.05 | 32.30 |
| C15 | 220,232,000 | 27,124,320,850 | 151 | 123.2 | 149.10 | 99.39 | 98.14 | 32.34 |
| C16 | 185,659,520 | 22,843,064,280 | 151 | 123.0 | 131.73 | 99.35 | 98.04 | 32.15 |
| C17 | 192,082,428 | 23,612,072,157 | 151 | 122.9 | 139.55 | 99.35 | 98.02 | 32.11 |
| C18 | 217,033,816 | 26,777,924,287 | 151 | 123.4 | 153.69 | 99.38 | 98.12 | 32.29 |
| C19 | 206,930,234 | 25,388,741,657 | 151 | 122.7 | 141.87 | 99.35 | 98.02 | 32.10 |
| C20 | 203,123,840 | 25,125,725,020 | 151 | 123.7 | 148.59 | 99.39 | 98.12 | 32.32 |
| C21 | 251,065,838 | 30,831,059,682 | 151 | 122.8 | 177.12 | 99.29 | 97.93 | 31.86 |
| C22 | 194,541,570 | 24,051,444,930 | 151 | 123.6 | 139.71 | 99.39 | 98.14 | 32.38 |
| C23 | 186,776,028 | 22,557,078,917 | 151 | 120.8 | 140.54 | 99.28 | 97.86 | 31.85 |
| C24 | 182,117,204 | 22,497,739,309 | 151 | 123.5 | 132.99 | 99.34 | 98.02 | 32.14 |
| **Mean** | 206,119,184 | 25,351,185,093 | 151 | 123.0 | 145.53 | 99.36 | 98.05 | 32.28 |
| **Median** | 206,448,815 | 25,393,622,339 | 151 | 123.3 | 143.21 | 99.38 | 98.05 | 32.30 |

Table S2: **WES sequencing statistics for the 23 sublines, spleen, and parental B2905 line.** Q20(%) (and Q30(%)) represents the percentage of bases that have a PHRED quality score of 20 (and 30) or over. Avg Qual and Med Qual are the mean and median PHRED quality scores for all passing reads, respectively. Means of %, average and median statistics are recalculated with total base count. Mean and median of N50 are calculated using the final N50 output per subline.

9. In Figure 2a, compared to what is reported in melanoma, the fraction of SBS7 is only appreciable in the trunk. This makes sense since this is the key site of the UV radiation induced mutational event. However, the proportion of SBS 7 is worryingly low. In contrast, some of the other signature SBS5 higher than what is expected. SBS5 is not attributable to a mutagen and more linked to aging. As a DNA repair error induced signature, it may be linked to high proliferation. Do the authors think this due to something like genetic drift?

   The reviewer is correct that the proportion of SBS7 mutations in the trunk is approximately 20%, which is perhaps lower than expected for clonal mutations in a cutaneous melanoma. Another relevant observation is that SBS5 has a similar proportion. Even though the exact tumor progression scenario is difficult to derive from a single model, a possible explanation is the following: while the cancer was initiated by UV damage, it continued accumulating mutations via other mutational processes (e.g. defective DNA repair), which was followed by a strong clonal sweep. If this is indeed the case, there should be a substantial time gap between the tumor initiation and the emergence of the most recent common ancestor (MRCA) of all clones - which is supported by the high number of mutations in the trunk, in comparison to the internal tree branches.

   We have now added this possible explanation to the section "Evolutionary dynamics of somatic SNVs and mutational processes". See your revision below in red:

   *"Interestingly SBS7 signature explained only ~20% of truncal mutations, which may suggest that another strong clonal sweep occurred after cancer imitation"*

   While the authors also tried to explain the SBS 85, it is not a common signature in melanoma. Do you think it is linked to the immunogenicity of this particular murine cell line. Given the specific purpose of this line, it may explain this signature.

   SBS85 signature is usually associated with Action-induced deaminase (AID), an enzyme that causes hypermutation of immunoglobulin genes in B cells. One explanation may indeed be that SBS85 could be a signature of an immune response. Studies have shown that UV can induce AID expression in a human melanoma cell line (J Clin Invest. (2016) 126:1367-1382), and AID expression was detected in human cutaneous melanoma (Experimental Dermatology (2014) 23, 345–368).

   Another possible explanation is that SBS85 signature reflected a mouse-specific mutational process with mutational signature similar to SBS85 in human tumors. Since the COSMIC signatures were derived from a large-scale human cancer sequencing dataset, they may not reflect all mutational processes in mice. We have revised the corresponding paragraph as follows in red:

   *"Some signatures were present throughout the phylogeny, such as clock-like signatures (SBS1 and SBS5), and SBS85 (attributed to activation-induced cytidine deaminase; AID) (Figure 2a., Supplementary Figure S8). While SBS85 has been typically described in the context of lymphoid cancers [Nucleic Acids Res. (2023), 52(D1):D1210–D1217] and is unusual for melanoma, it may reflect a signature of immune response. It has also been shown that inflammation-induced AID expression in non-lymphoid cells promotes skin cancer development independently of UV damage [Nature Commun. (2015), 6(1):8866]. Another possible explanation is that SBS85 signature reflected a mouse-specific mutational process with mutational signature similar to SBS85 in human tumors."*

   Additionally, given the sample, did the authors attempt to generate de novo signatures and then attempt to assign to the known signatures as well as identify possibly new signatures?

Although this is a good suggestion for future studies, unsupervised generation of new signatures would require sequencing of hundreds of samples to reach statistical significance *(Alexandrov et al., Nature (2020) 578 (7793), 94-101)*, and therefore is beyond the scope and capacity of the current study.

10. The authors provide an analysis of structural variants (SVs) identified by long-read sequencing and attempt to assess whether these SVs could have been detected using bWES or scRNA-seq by evaluating coverage at breakends. While this approach may suggest the increased resolution afforded by long-read sequencing, the claim that >86–92% of SVs would have been missed in short-read data highly overstate the limitations of conventional technologies. Coverage does not equate to detectability. It also depends on mapping, algorithm sensitivity, read pairing etc. In real experiments, including ones published for multiple prominent studies in the field have made SV calls with far lower coverage with high sensitivity and specificity. I am also confused with the use of scRNA-seq data for this. scRNA data inherently have sparse coverage. This use overstates detectability limitations because most SVs occur in non-expressed regions.

    It seems that our coverage-based comparison was indeed misleading due to several reasons. First, we used an overloaded term “coverage”. In this context, we referred to presence/absense of any read coverage of a particular region, rather than “depth of coverage”, e.g. how many reads cover a region. For “depth of coverage“ agree with the reviewer that coverage does not equal detectability for a wide range of somatic variation types. However, to detect an exact SV breakpoint or SNP, one needs to have at least a single read that spans the variant site. Although terminology may not be ideal here, we couldn't think of a better term, and “coverage” is widely used in this context, e.g. in samtools (https://www.htslib.org/doc/samtools-coverage.html).

    Further, exact meaning of CNA and SV “detection” is also important: large CNAs could be detected from a sporadic WES coverage, however, exact SV breakpoints coordinates will be unknown. Similarly, expressed gene fusions could be detected from scRNA-seq without knowing the exact DNA breakpoint coordinate in case it is inside an intron. In this work, we are specifically interested in detecting precise breakpoint coordinates to resolve parallel CNAs, thus having non-zero read coverage of each breakpoint is necessary.

    To clarify the confusing parts we have now added the following discussion to the benchmarking section (now supplementary note S1) in red:

    *“We compared different technologies in terms of quality  and consistency of variant calls, as well as genomic coverage - which refers to the regions of the genome that are accessible to technology and covered by at least a single read (not to be confused with coverage depth, which refers to sequencing redundancy). For the SV discovery, we require the exact breakpoint coordinates to be known for an SV call to be made. Although approximate SV boundaries could be often discovered from sparse data, they can not fully resolve parallel events, which was critical in our study. We also note that other recent studies provided comprehensive evaluation of long-read sequencing for somatic SV discovery [Nature Biotech. (2021) 39(2):207–214; Nature, (2020) 578(7793)].”*

    While I do not question the clear advantage of long-read sequencing for detecting large SVs, the way the results are presented gives a somewhat exaggerated impression of the deficiencies of short-read methods.

We agree that this analysis is not a comprehensive comparison of short- and long-read sequencing technologies, and the reviewer rightly points out that other factors, like mapping accuracy should be considered. To avoid overinterpretation of our analysis in the Results section "Long-read whole-genome sequencing substantially improves resolution of genomic variants", we have moved the text and Figure 7 to the supplementary materials (the text is now moved to Supplementary Section S1, and Figure 7 is now Supplementary Figure S23). We also note there is a growing number of other comprehensive comparisons of short- and long-read technologies for SV detection (Keskus et al., 2025, Elrick et al. 2025), and we now added references to those for a more comprehensive comparison between short- and long-read technologies.

Minor Comments

1. "For concision of presentation, activities for signatures SBS7a, SBS7b, SBS7c, and SBS7c were aggregated" should probably be "SBS7a, SBS7b, SBS7c, and SBS7d".

   Indeed! We thank the reviewer for catching the typo. The text has been revised.

## Reviewer #2

Remarks to the Author

In this manuscript, the authors investigate the genomic and DNA methylation heterogeneity among 23 subclones derived from a mouse melanoma cell line established in a recent study, using Oxford Nanopore long-read sequencing. They further develop a computational approach to classify different types of variants along the phylogenetic branches and identify subclonal or private alterations that may contribute to the aggressive phenotypes observed in certain sublines. Overall, the study is interesting and provides valuable sequencing data that may serve as a resource for the community. However, several points require clarification and additional discussion to improve the clarity and interpretability of the work.

We thank the reviewer for their supportive feedback. Please find our detailed responses below.

Major Comments

1. (i) TreeHarmonizer takes a pre-constructed phylogenetic tree as input. Could the authors clarify whether the resulting tree topology may differ from the input tree? If so, how should such topological discrepancies be interpreted?

   By design, TreeHarmonizer assumes that the input tree topology is optimal, and will not modify it. SNV, SV, CNA and DMR Variants are then placed onto branches of that topology. It is important to note that our workflow ensures that the input tree topology is accurate. In the initial construction of the tree, we remove SNVs that fall within regions that have called deletions or losses, ensuring that the infinite sites assumption is not violated. In addition, one is able to assess the correctness of the tree by observing the proportion of variants that are placed or unplaced at a particular subtree.

   We have clarified this within the results section in Page 5 Section 2; please see our revision below in red:

   *"TreeHarmonizer assumes the input tree is optimal and most correct. It does not directly evaluate the validity of the existing topology nor does it change it."*

(ii) Additionally, how do the authors reconcile noticeable differences in phylogenetic structures reported across different studies using the same subclones (Hirsch et al, Gruen et al and this one)?

The reviewer is correct that the trees are not identical, however they are very similar. A few differences in placement of individual sublines are explained below, and overall we believe that the long-read based tree is more accurate.

The Hirsch et al and Gruen et al trees were both constructed using short read whole exome sequencing data introduced in an earlier paper [bioRxiv 2021.03.26.437185], where subline 2 appears to harbor cells from subline 3, and subline 19 appears to harbor cells from subline 21, as evidenced by single-cell whole transcriptome sequencing analysis (Figure 4, panel c of that paper). Because subline 2 was contaminated at an earlier stage of sample preparation, it was not possible to purify it and thus it was excluded from our current study. Fortunately the contamination of subline 19 was limited in scale (as demonstrated through a partition function analysis in a recent study in Proceedings of RECOMB 2025, pp 409-413) and occurred during (short read whole exome) sequencing, thus we were able to use an uncontaminated version of subline 19 for further (long read whole genome) sequencing. The new phylogeny places subline 19 in the green clade rather than the blue clade (this is also supported by the above partition function analysis) where sublines 17 and 19 form a distinct subclade. The only other difference between the trees is in the relative placement of the blue and the orange clades. In our current study, the orange clade is closer to the root than the blue clade. The trees mentioned by the reviewer placed the blue clade closer to the root based on a single mutation observed in the short read whole exome data. Our current study uses long reads covering the whole genome: not only do they feature many more mutations, but also their alignments are more reliable. Thus the tree we present in the current study better reflects the evolutionary history of the tumor. The fact that there are only minor differences between the trees despite each being based on a distinct sequencing technology provides further evidence for the overall correctness of the tree topology presented in our study.

We have now added a following clarification in red to the Methods section:

*“We note that the final tree is highly consistent with the trees reconstructed from WES data in companion studies [bioRxiv, 2023.04.03.535074; Cell Systems, (2025) 16(1)], with a few minor differences. First, different placement of C19 is explained by contamination in samples used for WES. Second, relative placement of the blue clade and the orange clade (except C5) was different, likely because of insufficient mutational signal in WES data. Since many more mutations were called using ONT sequencing, the tree described here is likely to be more accurate, compared to the WES-based trees.”*

2. Have the authors considered scenarios in which mutations occurring in regions with copy-number gain (≥3 copies) in the parental line might be lost in sublines that subsequently lose extra copies (e.g., reverting to diploid)? Such changes could remove somatic mutations without being categorized as “deletions,” potentially leading to loss of heterozygosity or variant absence in those sublines.

We thank the reviewer for pointing out such complex scenarios. We note that our approach to identify regions of loss from CNA and SV calls has limitations in the challenging scenario of overlapping gains and losses. We used Severus deletion calls and Wakhan CNA profiles to filter variants in the lost regions. For the scenario of overlapping gain and loss events that result in a diploid state for a genomic region, if such an event had discoverable breakpoints, then it would have been seen in the form of a deletion called by Severus and would have been considered in variant placement. If there are overlapping events with no detectable breakpoints, then it would be much more difficult to call, and we do not currently handle such events. For example, this may occur in regions with copy neutral losses of heterozygosity if both loss and gain involve the whole chromosomes. However, we note that such overlapping gains/losses with no informative breakpoints are statistically less likely, compared to the typically observed events, and we expect that they should not substantially affect the resulting tree structure. Such complex gains/losses and rearrangements are difficult to detect for all existing methods that work with phylogenies using CNAs. We are currently working towards developing a model that can handle such overlapping CNA scenarios and hope to incorporate it into TreeHarmonizer in the future studies.

We have now incorporated the following discussion into the Methods:

*"We note that our approach to identify regions of loss from CNA and SV calls has limitations in the challenging scenario of overlapping gains and losses. We used Severus deletion calls and Wakhan CNA profiles to filter variants in the lost regions. For the scenario of overlapping gain and loss events that result*

*in a diploid state for a genomic region, if such an event had discoverable breakpoints, then it would have been seen in the form of a deletion called by Severus and would have been considered in variant placement. If there are overlapping events with no detectable breakpoints, then it would be much more difficult to call, and we do not currently handle such events. For example, this may occur in regions with copy neutral losses of heterozygosity if both loss and gain involve the whole chromosomes. However, we note that such overlapping gains/losses with no informative breakpoints are statistically less likely, compared to the typically observed events, and we expect that they should not substantially affect the resulting tree structure. Such complex gains/losses and rearrangements are difficult to detect for all existing methods that work with phylogenies using CNAs. We are currently working towards developing a model that can handle such overlapping CNA scenarios and hope to incorporate it into TreeHarmonizer in the future studies."*

3. Based on the description, UV exposure occurred during tumor model generation but not during the establishment of the parental and sublines. Nevertheless, SBS7-type mutations were observed on internal and terminal branches, albeit at lower fractions. Could the authors elaborate on possible biological or technical explanations for this observation?

   We thank the reviewer for this interesting question. Indeed, it is surprising to see the presence of SBS7 in the internal and terminal branches of the tree, as UV exposure is expected to be an early event in the evolution of this model. We think that this is explained by technical limitations of the current mutational signatures analysis software, rather than a biological phenomenon.

From a technical perspective, this may be due to artifacts from SigProfilerAssignment, the state-of-the-art tool we employed for mutational signature detection. Our study used SigProfilerAssignment to estimate mutational signature exposure for each subline sample, then recompute per-branch signature exposure using a majority-vote strategy. Thus, SigProfilerAssignment is blind to the tree structure. Since the assignment of signatures is a statistical procedure, given that the algorithm detected the presence to SBS7 in each sample, it may overassign individual variants to this signature because of similarities in mutational profiles.

Instead of this approach, we have considered two alternatives: (i) treating each branch as a separate sample by considering the mutations assigned only to that branch, and identify the signatures as well as signature exposure on that branch, or (ii) creating a pseudobulk of all the sublines for identifying signatures and estimate the exposure for each branch based on the signatures of the mutations assigned to that branch. After trying those alternative approaches, we found the former tend to result in overfitting - evident by the notably higher number of mutational signatures noted as active - and the latter seems to sacrifice subclonal resolution. In our manuscript, we avoid these problems by taking the more balanced approach. While our approach preserves major signals, it sacrifices sensitivity to signature exposure shifts at smaller parts of the phylogeny. As a result, the estimated exposures per branch may contain potential artifacts (i.e. SBS7) possibly due to misassignment of C>T mutations. New methods are likely needed to properly capture the local dependencies of signature exposure in the tree, although it is out of the scope of the current work.

We have added the following discussion into the Methods:

*"We note that the current signature decomposition results point to some potential limitation of the current signature decomposition methods. For example, it is surprising to see the presence of SBS7 in the internal and terminal branches of the tree, as UV exposure is expected to be an early event in the evolution of this model. Because we ran SigProfilerAssignment on each subline independently and since the assignment of signatures is a statistical procedure, given that the algorithm detected the presence to SBS7 in each sample, it may overassign individual variants to this signature because of similarities in mutational profiles. We also have considered two alternatives approaches: (i) treating each branch as a separate sample by considering the mutations assigned only to that branch, and identify the signatures as well as signature exposure on that branch, or (ii) creating a pseudobulk of all the sublines for identifying signatures and estimate the exposure for each branch based on the signatures of the mutations assigned to that branch. We found the former tend to result in overfitting - evident by the notably higher number of mutational signatures noted as active - and the latter seems to sacrifice subclonal resolution. We chose to avoid these problems by taking the more balanced approach. While ours preserves major signals, it may sacrifice sensitivity to signature exposure shifts at smaller parts of the phylogeny, resulting in misassignment of common base changes. New methods are likely needed to properly capture the local dependencies of signature exposure in the tree, although it is out of the scope of the current work."*

4. Could the authors comment on the impact of the relatively lower sequencing quality of ONT on SNV detection?

The reviewer is correct that earlier versions of ONT chemistry had high base-level error rates, however recent protocols have largely resolved this issue. Us and other groups have shown recently that ONT is now comparable with Illumina on germline variants (https://www.nature.com/articles/s41592-023-01993-x; see Figure 2). We came to a similar conclusion in a recent study that benchmarked somatic variant calling of various technologies (https://www.nature.com/articles/s41587-025-02839-x; Figure 3). We note that analysis of small indels with ONT remains challenging, and thus we focused on SNVs in this work. We have now added the discussion below to the manuscript - see our revision in red:

*"On average, this yielded 69.77Gbp of data per subline with a mean read N50 of 29.1Kbp and an average of 82.95% of base pairs with a quality score of 20+ (Supplementary Table S1). For comparison, WES runs on the same sublines yields an average length of 123 bp per read, with a 99.36% of base pairs with a quality score of 20+ (Supplementary Table S2). We note that most of the ONT base errors are indels, and SNP calling quality is similar for ONT and Illumina* [*Nature Methods (2023) 20:1483–1492*]."

5. In Figure 2a, the numbers of different variant types (SNV, SV, CNA, and DMR) are shown as colored blocks. The order of these blocks, however, varies among the trunk, internal, and terminal branches. For consistency and easier visual comparison, the order of categories should remain uniform across panels.

   We thank the reviewer for this suggestion. We have updated the node label placements in Figure 2a so that there is more visual consistency. Note that it was difficult to modify the label placements for internal nodes, since (i) there is less space due to node density, and (ii) they each have an additional DMR label. The current placements for internal node labels, in our opinion, provide the best trade-off between the clarity and completeness of the presented information. That said, to make the figure more visually intuitive, we (i) modified the leaf node labels so that the SV label is presented "before" the SNV label, as they are for the internal nodes, and (ii) we modified the root node label placements to be consistent with the leaf node label placements.

6. In the Abstract, the authors state that they "introduce an approach for long-read sequencing of single-cell-derived subclones." This statement is rather vague. Please revise.

   We agree with the reviewer that it is a vague statement. We modified the text as suggested; see our revision below in red:

   *"Here we introduce an approach to identify and analyze the genomic and epigenetic events in different stages of tumoral evolution using long-read sequencing of single-cell-derived subclones."*

7. Please indicate the estimated ploidy of each subline. Did any sublines exhibit evidence of whole-genome doubling?

   We have now provided a new supplementary table S6 with ploidy values for each subline; see below. The median ploidy was 2.25, and we did not observe any evidence for whole genome doubling in our data. In addition, in this revision we have also provided a comparison of large CNA calls against the spectral karyotyping (SKY) for approximately half of sublines, which showed ~90% agreement between the long-read calls and SKY data (see Reviewer 3, question 10). We did not observe whole-genome duplication in the SKY data either.

| Subline | Weighted Ploidy |
|---|---|
| C1 | 2.41 |
| C3 | 2.37 |
| C4 | 2.33 |
| C5 | 2.21 |
| C6 | 2.25 |
| C7 | 2.17 |
| C8 | 2.26 |
| C9 | 2.29 |
| C10 | 2.21 |
| C11 | 2.19 |
| C12 | 2.08 |
| C13 | 2.28 |
| C14 | 2.25 |
| C15 | 2.38 |
| C16 | 2.20 |
| C17 | 2.21 |
| C18 | 2.38 |
| C19 | 1.95 |
| C20 | 2.25 |
| C21 | 2.24 |
| C22 | 2.38 |
| C23 | 2.24 |
| C24 | 2.26 |
| **Mean** | 2.25 |
| **Median** | 2.25 |

8. The sequencing depth for each subline should be provided in a supplementary table, to enable assessment of variant detection sensitivity.

   We thank the reviewer for noticing this missing information. A column (Avg Depth) has been added to Supplementary Table S1 (and consequently to WES statistics in Supplementary Table S2) to enable this assessment.

9. The order of references appears very random. Please ensure that references are cited sequentially according to their first appearance in the text.

   We thank the reviewer for this comment. We have changed the order of references to by their order of appearance in text.

Remarks on code availability

1. The code provided appears to be specifically for the analyses presented in this manuscript. While this is acceptable if the intent is to illustrate how the analyses were performed, in this case the software constitutes a key component and is intended for public dissemination. Therefore, it would be preferable for the authors to provide a cleaner, standalone version of the code that is broadly applicable and more accessible for other researchers to use.

   We agree with the reviewer that the software constitutes a key component of this manuscript. We have updated the TreeHarmonizer repository (https://github.com/KolmogorovLab/TreeHarmonizer) and released the software as a standalone tool in release 1.0.0. We maintain the presence of paper-specific jupyter notebooks and their respective environment in a directory within the repository for ease of access and replication.

**Reviewer #3**

The manuscript proposes novel insights into tumor evolution using long-read sequencing of single cell-derived melanoma subclones. The authors developed a new computational framework to integrate genomic and epigenomic (SNVs, SVs and DMRs ) to reveal evolutionary trajectories. The manuscript is well-organized and the project is solid. However, here are some major concerns:

We thank the reviewer for their helpful insights, please see our detailed responses below.

Major Comments

1. Nanopore long-read sequencing generally presents lower sequencing accuracy when compared with short-read technology. How did the authors specifically control the false discovery of SVs and SNVs?

   The reviewer is correct that earlier versions of ONT chemistry had high base-level error rates, however recent protocols have largely resolved this issue. Us and other groups have shown recently that ONT is now comparable with Illumina on germline SNP calls (https://www.nature.com/articles/s41592-023-01993-x). We came to a similar conclusion in a recent study that benchmarked somatic SNP calling of various technologies (https://www.nature.com/articles/s41587-025-02839-x). We note that analysis of small indels with ONT remains challenging, and thus we focused on SNP/SNV calls in this work. For somatic SV discovery, us and others have demonstrated that long-read sequencing is superior to short-read technologies (https://www.nature.com/articles/s41587-025-02618-8, https://www.nature.com/articles/s41592-025-02708-0). We have now added the following changes to the manuscript:

   *Results:*
   *"On average, this yielded 69.77Gbp of data per subline with a mean read N50 of 29.1Kbp and an average of 82.95% of base pairs with a quality score of 20+ (Supplementary Table S1). For comparison, WES runs on the same sublines yields an average length of 123 bp per read, with a 99.36% of base pairs with a quality score of 20+ (Supplementary Table S2). We note that most of the ONT base errors are indels, and SNP calling quality is similar for ONT and Illumina* [*Nature Methods (2023) 20:1483–1492*]."

   *Supplementary Note S1:*
   *"We compared different technologies in terms of quality and consistency of variant calls, as well as genomic coverage - which refers to the regions of the genome that are accessible to technology and covered by at least a single read (not to be confused with coverage depth, which refers to sequencing redundancy). For the SV discovery, we require the exact breakpoint coordinates to be known for an SV call to be made. Although approximate SV boundaries could be often discovered from sparse data, they can not fully resolve parallel events, which was critical in our study. We also note that other recent studies provided comprehensive evaluation of long-read sequencing for somatic SV discovery and illustrated their substantial improvements over short-read sequencing [10, 4]."*

2. In Table S1, can the authors add in a depth calculation (such as 10X or 50X)? 'we removed SNVs with low coverage': Dose it refer to the low-coverage SNVs per subclone or across all the 23-subclones? Is it possible that a variant (SNV, SV or DMR) is positive for one subclone but negative for the others, just caused by the uneven sequencing depth across the 23 samples? In Table S1, the lowest # Reads is 1.8M, while the highest is 10.8M. Will different depths influence the evolutionary trajectory results? Low depth for a certain loci or region will also result in false positives or false negatives? How did the author balance these?

   We have now added the mean sequencing depth to the table S1: the median depth per subline was 25x (ranging 10x - 31x). Based on the benchmarking studies referenced in the question above, we have sufficient depth for high-quality SNV and SV calling within individual sublines. Low coverage regions may still arise, but typically due to mapping issues, e.g. inside very long repeats, such as segmental duplications or pericentromeric regions. Low coverage filtering is a simple way to remove variants in these uncertain regions. We have now added the following clarification to the methods section titled "Constructing tumor phylogeny using long read sequencing data from 23 sublines":

   *"Although long-read sequencing can map the vast majority of the genome (Supplementary Figure S23a), highly repetitive regions (such as centromeres or segmental duplication) remain challenging and may remain unmapped in multiple sublines. To avoid sites with consistently low coverage, as their mutation calls are unreliable across many sublines, we also filtered out sites with mean or median coverage across all sublines < 20."*

   Further, even if the overall genomic coverage is sufficient, because of its fluctuations some regions may randomly receive smaller coverage; and the reviewer is correct that such cases should be handled with caution. To account for that, Severus was run in multi-sample mode, where as low as a single read can be used to detect SV in a sample, if it has reliable support in other samples. We have added the following clarification to the methods section "Extending Severus for multi-sample calling":

   *"SVs were called across all sublines in a single run using the multi-sample mode of Severus [9], which eliminates the need for ad-hoc re-genotyping. Unphased BAM files from each subline and the normal sample were provided as input. Severus first produces the calls from a combined alignment of all input samples. Then, it genotypes the presence or absence of each SV in each sample individually. This allows us to discover variants supported by only a few reads, if a variant is confidently called in other samples."*

   Finally, TreeHarmonizer was specifically designed to place variants in the tree even if some variant calls might be missing in individual sublines. Methods section describes that TreeHarmonizer was run with up to 15% false-negative rate in SNV calls, and therefore a limited amount of calls missing due to low coverage should not substantially affect phylogeny construction.

3. Figure 2 & 3: Only chr1-chr19 were analyzed. Why were chrX and chrY excluded from the study?

   Our initial analysis showed very high rates of likely-erroneous calls from chrY. This is explained by two factors: first, chrY is very difficult to assemble because of large number of repeats (https://pmc.ncbi.nlm.nih.gov/articles/PMC10752217/), thus it is likely that the gcrm38 chrY have reduced assembly quality, compared to the rest of the assembly. Secondly, since the M4 mouse was a F1 cross between C57B6J female and FVB male, it inherited FVB chrY, which seems to be substantially distinct from the reference version (which is based on C57B6J strain). Because of the many observed mapping artifacts, we excluded chrY from the analysis. Since we excluded chrY, we decided to focus on autosomes and excluded chrX as well.

In response to the reviewer request, we have now added any of CNA, non-synonymous SNV, SV, and DMR at COSMIC orthologs on chrX in new Supplementary Figure S16. It illustrates that the evolutionary history of chrX is consistent with the rest of the chromosomes. Thus in this revision we preferred not to incorporate the analysis of chrX to the rest of the main figures, as it would require redoing most of the analysis and statistics through the paper.

We have added the following note to the Results section:

*"Our initial analysis showed very high rates of likely-erroneous calls from chrY; In humans chrY is very difficult to assemble due to high repeat complexity (https://pmc.ncbi.nlm.nih.gov/articles/PMC10752217/), and this may influence read mapping in mouse chrY. Secondly, since the M4 mouse was a F1 cross between C57B6J female and FVB male, it inherited FVB chrY, which seems to be substantially distinct from the grcm38 (which is based on C57B6J strain). Because of the many observed mapping artifacts, we excluded chrY from the analysis, and decided to focus on autosomes (excluding chrX as well). In a separate analysis of CNAs, SNVs, SVs, and DMRs on chrX, we verified that its evolutionary history is consistent with the rest of the chromosomes (Supplementary Figure S16)."*

4. Does the tool TreeHarmonizer treat all the SNVs and SVs independently, or will it consider the association of neighboring variants, such as Linkage Disequilibrium?

   TreeHarmonizer treats all SNVs and SVs independently. Considering linked mutations could be useful for analysis of germline polymorphisms, however here we assume that somatic mutations are independent in the sense that they are randomly and sequentially acquired. In principle one can use long-reads to phase somatic variants into haplotypes, however, there still do not exist tools to phase multiple somatic haplotypes in cancer, as all long-read phasing tools have been developed for diploid genomes.

   We have added a clarification to the "TreeHarmonizer algorithm" section:
   *"Variants of each type are assumed to be acquired independently"*

5. Does the algorithm consider the genomic location of the variants, such as repetitive regions (which are more challenging in variant detection)? Or their location at the exon, intron or intergenic regions?

   For the TreeHarmonizer variant placement algorithm, it currently does not explicitly distinguish the genomic context of the variants. In principle, this would have been useful for the analysis of certain variant types, for example repeat expansion or contractions - as they can have specific error and evolutionary patterns. Because of increased indel error rates in ONT data, here we are focusing on SNVs to build the tree and place variants. Prior studies have shown that ONT SNV calls remain reliable in most of the genome, thus we don't need to consider the context of SNVs for evolutionary inference.

   However, other parts of the workflow, such as variant callers, are considering genomic context to improve output accuracy. For example, Severus detects unreliable genomic regions based on coverage irregularities. Severus also directly takes in a VNTR annotation file to consider those regions. Likewise, DeepVariant is based on a ML model that can learn the relationship between genomic context (e.g. elevated coverage for repeats) and variant call quality.

   We have now added the following clarification into the "Constructing tumor phylogeny using long read sequencing data from 23 sublines" section:

*"We note that our filtering model does not explicitly consider genomic context (e.g. repetitive regions), however SNV and SV variant callers use genomic context for quality control."*

6. "Indeed, TreeHarmonizer variant placement led to a significant increase (one-tailed paired-sample t-test t(26) = 11.99, p = 2.133e−12) in their partition function values, confirming accurate variant placement (Figure 7g.)." Does the data follow normal distribution so that the t-test can be applied?

   We thank the reviewer for this comment. We have modified the statistics in the figure (now Supplementary Figure S23g.) to use the non-parametric Wilcoxon signed-rank test instead, and we have modified its mentioning in the main text:

   *"Indeed, TreeHarmonizer variant placement led to a significant increase (one-tailed Wilcoxon signed-rank test p = 9.331e − 06) in their partition function values, confirming accurate variant placement (Supplementary Figure S23g.)"*

7. There is a lack of systematic comparison of the tool TreeHarmonizer with the existing tools. Please provide more details on the algorithm novelty of the proposed tool, as well as performance comparison (accuracy, computational efficiency, as well as other evaluation metrics).

   The novelty of TreeHarmonizer is a combination of (i) joint consideration of SNVs and CNAs and (ii) scalability for WGS input. CNA analysis is typically not utilized by the scalable tumor phylogeny reconstruction methods (such as ScisTree that is incorporated in our workflow). The reviewer is correct that there are several existing methods that are conceptually similar to TreeHarnomizer, as they jointly consider SNVs, SVs and CNAs phylogenetic placement. However, all these methods have practical limitations that make it difficult to apply them to the analysis of single-cell derived subclones. Overall, these alternative methods fall into two categories: (i) those designed for a different type of input (e.g. multi-sample bulk data) and thus are not applicable here or (ii) those designed for much smaller inputs, e.g. exome or targeted sequencing, and thus do not scale for whole-genome analysis.

   In contrast, SCARLET and ConDoR were designed for single-cell input, and in principle are applicable to our data since each sample is a single-cell derived subclone. In this revision we examined the possibility of running SCARLET and ConDoR for our dataset. The analysis shows that both tools do not scale to the number of variants (tens of thousands) we have in our dataset - as shown in the plots below (which depicts the runtime of each tool with respect to the number of variants subsampled). In contrast, the runtime of ScisTree on the ~10.8k filtered variants with multiple seed permutation testing takes only 4 hours. That is followed by TreeHarmonizer on the entire dataset of ~450k variants, which adds only an additional 8.5 minutes.

   The new analysis is described in the new supplementary section S4 and supplementary figure shown below. Also see our response to a relevant Question 1 from the Reviewer 4.

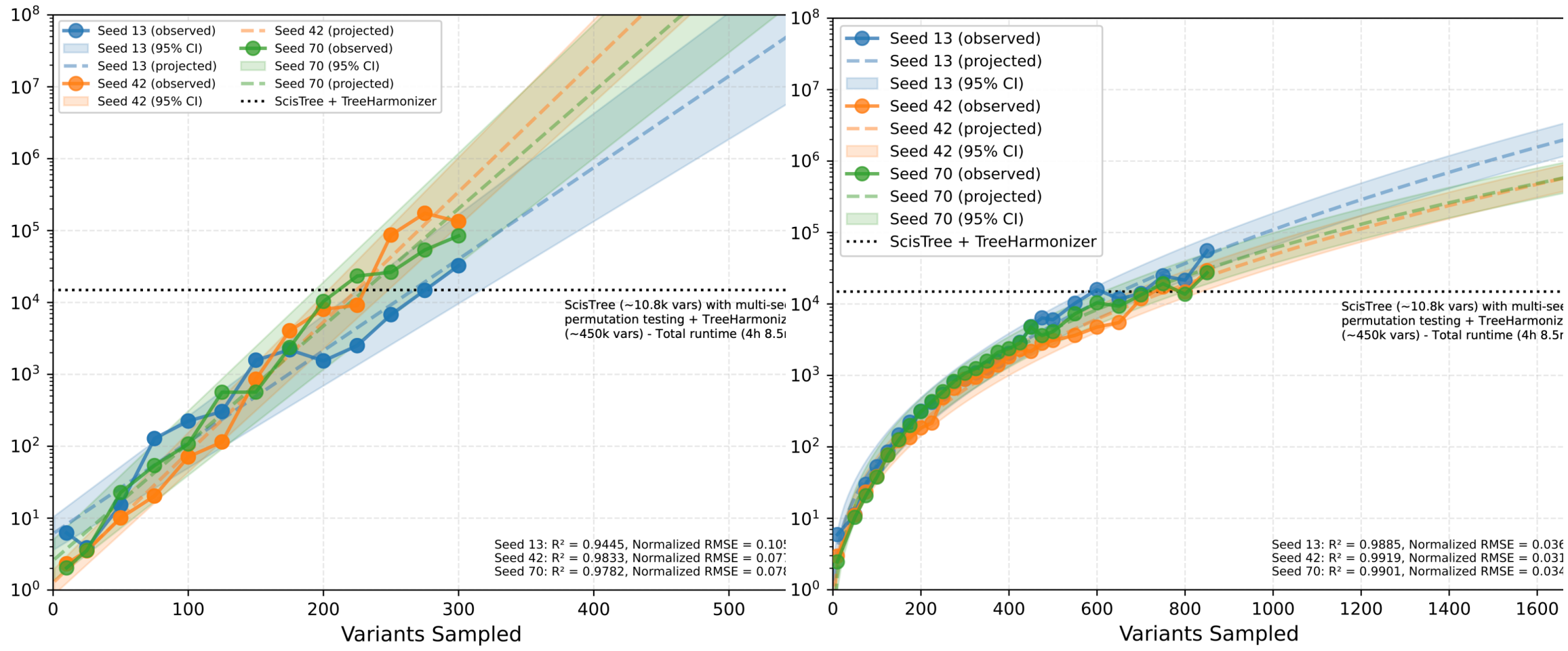


a) ConDoR Benchmarking b) SCARLET Benchmarking

In terms of evaluation, we note that since we do not have the correct placements of the variants in the tumor phylogeny, we can not directly evaluate TreeHarmonizer's variant placement accuracy. Since the alternative tools can not handle our dataset, we can not compare TreeHarmonizer's results with these tools either. However we have evaluated the placements of 27 variants by TreeHarmonizer, which differed from their original placements by ScisTree (the tool used to infer the tumor phylogeny) as given below (also in Figure S23 panel g in the Supplementary materials).

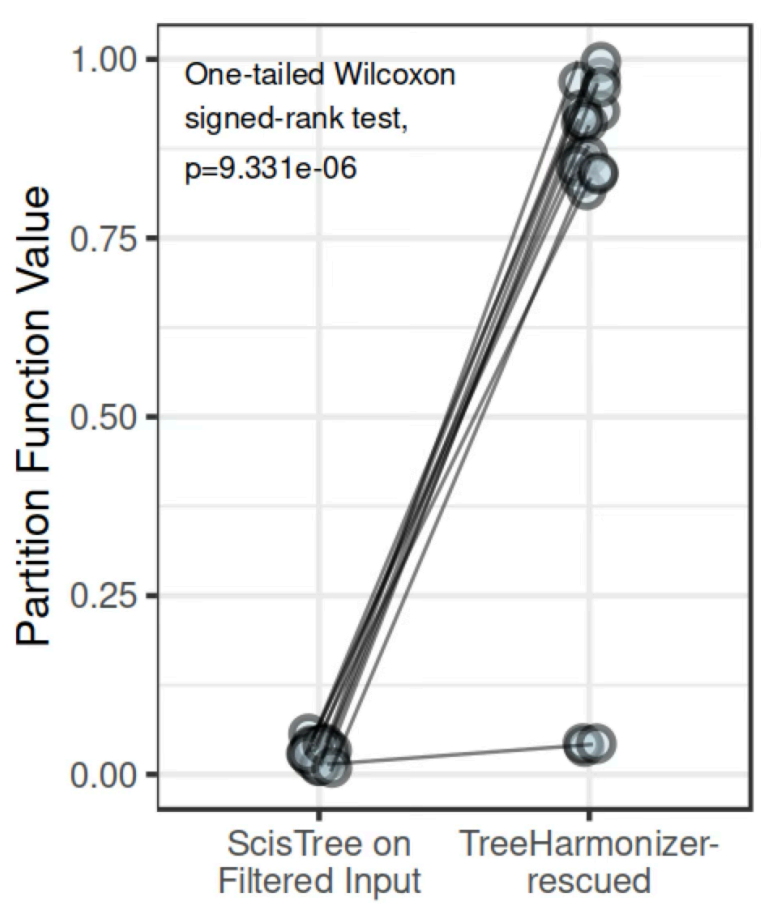


For this evaluation we used the partition function values, a recently introduced concept for evaluating the node placement of a variant in a given tumor phylogeny. The partition function is defined as the likelihood of the variant to be present in all descendant cells of the said node, across all possible scenarios for the tumor's evolution [Proceedings of RECOMB (2025) pp 409-413; Proceedings of WABI (2025) 5:1-18]. As can be seen in the figure, the TreeHarmonizer improves the partition function values of all 27 variants whose original (ScisTree) placements were altered by TreeHarmonizer. The partition function values of the original placements of these variants were all close to 0, and after TreeHarmonizer modification, 23 out of 27 variants' partition function values improved to nearly 1.

8. On the TreeHarmonizer GitHub README file, detailed installation pipeline is available. However, there is no instruction on how to run the tool. Especially in the 'Quick Run' section, no detailed instruction on how to run the demo data and how to understand the output file.

   We have now substantially improved the TreeHarnomizer code repository and manual to make sure it is easily applicable to other similar datasets. It is released as a fully standalone tool as of version 1.0.0. We also made sure that all relevant tools (including padfoot) developed by our group are now publicly available.

9. Besides mouse, is the tool and analysis pipeline generalizable to other species (such as human or rat)?

TreeHarmonizer and all variant calling methods in our workflow are general enough and should work with any organism. The key consideration would be the availability of high-quality reference genome. Another important factor is heterozygosity rate - mouse models are inbred, while wild animal models and humans have heterozygous germline variants that could be phased (see our response to the question 12 below for more detail). We are currently collaborating with the Genome in a Bottle consortium to apply TreeHarmonizer to multiple related pancreatic cancer cell lines to study the tumor clones (https://www.nist.gov/programs-projects/cancer-genome-bottle).

In response we added a clarification to the Discussion:

*"Tracing the variants at different genomic, epigenetic, and transcriptional levels and integration of these data can expand the scope of evolutionary modeling of cancer beyond the classic viewpoint focusing on coding regions and/or small variants in a snapshot manner, leading to testable hypotheses to gain mechanistic insights, which in turn may help develop therapeutic strategies. Likewise, the TreeHarmonizer method, as well as phylogenetic and variant calling methods described here are not limited to mouse genome analysis, and could be applied to multi-site or longitudinal human tumor datasets."*

10. Have the key SNVs, SVs or DMRs detected from the long-read data been experimentally validated?

(This response closely follows our response to a relevant Question 3 from the Reviewer 4)
In the revised manuscript, we have now incorporated several new validation and comparison experiments for all variant types, and highlighted the relevant existing comparisons. We now provide several lines of evidence of high quality of variant calls, primarily based on (i) orthogonal support by other experimental technologies, (ii) orthogonal support by alternative tools and (iii) support by multiple sublines and consistency with evolutionary history. We describe them as the following:

**For small variant calls**, we compared the SNVs in coding regions called using long-reads, short-read WES and scRNA, and showed the high consistency rates (94% and 67% for WES and scRNA, respectively). This is discussed in Supplementary Figure S23 and Supplementary section S1 (which was moved from the main text in response to another reviewer's comment). In addition, we have now compared the SNVs called by DeepVariant to several other state of the art variant callers (DeepSomatic, Clair3 and ClairS) that support long-read technologies and the ONT R10.4 chemistry. We find that there is high concordance between all four callers on subclonal (variants called in 2 or more but not all sublines) and private variants, and high concordance between all callers except for Clair3 in clonal calls. This is discussed further in a new Supplementary Section S3 and shown in accompanying Supplementary Figures S27a, b and c.

**For SV and CNA calls**, in this revision we have added a comparison of chromosome-scale rearrangements against the spectral karyotyping (SKY) images, available for 12 sublines (new Supplementary Figure S25). The results are summarized in a new supplementary section S2, illustrating ~90% consistency between large CNAs, interchromosomal SVs and SKY images.

In addition, we **compared our SV calls against the calls generated by Sniffles2** (the only other long-read SV caller that supports multi-sample mode, however not designed for cancer). We placed the detected SVs from both tools on the tree using TreeHarmonizer. SVs detected by both tools (n = 489) were all consistent with the phylogenetic tree. In contrast, SVs unique to Sniffles2 (n = 1703) were often inconsistent with the tree structure, likely driven by mismapped reads in collapsed duplication

regions (76%) (new supplementary Tables S9, S10). In comparison, most SVs uniquely detected by Severus were supported by the phylogenetic relationships. This analysis suggests that (i) most informative subclonal and clonal SVs detected by Severus were also supported by Sniffles2 and (ii) Sniffles2 generated many likely false-positive calls. This analysis is described further in a new Supplementary Section S3.

**We further compared the CNA calls against another state of the art caller, CNVkit**. The concordance between Wakhan and CNVkit was very high (median Pearson's correlation coefficient between Wakhan and CNVkit profiles = 0.9530, $p<1e-300$). Exact breakpoints around copy number changes may differ between callers, however. This is due to the fact that Wakhan is able to incorporate precise breakpoint information from SV callers such as Severus, whereas CNVkit does not. We compared the integer copy number calls between the two callers within equal size bins of 50kb, finding a median exact match of 96.70% of bins across all sublines. Furthermore, the standard segmentation method of CNVkit (cbs, circular binary segmentation) is not able to detect smaller, focal amplifications and losses. Other callers, such as PURPLE, rely heavily on the human genome, biases which cannot be changed by the end-user. This analysis is described further in a new Supplementary Section S3.

**For DMR calls,** it is difficult to validate the change in methylation directly, as it would require a costly experiment to generate bisulfide sequencing for each subline. Instead, we looked into the consistency between predicted DMRs and change in gene expression measured by scRNA-seq. Consistent with the general understanding, we found the mean methylation differences between case and control groups at promoter/enhancer regions are negatively correlated with the measured expression differences of the corresponding genes (see Figure 5d). This provides orthogonal validation that the DMRs at functionally interpretable regions have led to expected downstream changes in gene expression.

Overall, this analysis above suggests that SNV, SV, CNA and DMR calls are highly accurate and could be used to trace cancer evolution or designing new benchmarking studies. All comparisons are now described in detail in the new supplementary section S3.

11. Please discuss and compare long-read WGS and scRNA-seq in revealing the heterogeneity within the sublines.

    By experimental design, our sublines were derived from single-cell clones and thus we do not expect to see a lot of heterogeneity within sublines. This can be confirmed by analyzing the variant frequency distribution (Supplementary Figure 2). It shows VAF normally distributed around the peak of ~0.5, with the exception of private SVs that may suggest acquisition of new private SVs, however we did not observe major signatures of subclonality in our CNA analysis. Although we expect to adapt TreeHarmonizer to multiple bulk tumor samples with clonal heterogeneity, our current data does not permit the analysis of heterogeneity within sublines.

    We now added the following statement to the Discussion:
    *"We note that analysis of multi-site bulk samples, each consisting of multiple cancer clones, would require a different approach to resolve clonal deconvolution and heterogeneity"*

12. Has the study specifically considered the Haplotype phasing?

Although we initially tried the haplotype phasing of the germline variants, our mouse model is highly inbred and has very low heterogeneity. Therefore we did not attempt to separate germline haplotypes, unlike the recent human genomics studies. We note that another possibility is to phase somatic variants into contiguous haplotypes, however there are currently no existing tools that can perform that. Our group is now working on a method to address this challenge, and we hope to incorporate it in evolutionary analysis in the future.

We now added the following clarification to the “Data preparation and somatic SNV calling” section:
*We also note that although variant calling tools used in this study support germline haplotype phasing to improve variant calling quality, our mouse strain is highly inbred and thus germline variant phasing was not possible.*

Minor Comments

1. Please provide more biological interpretation of the defined clonal, subclone and private variants.

   To understand the biological function, we focused on the analysis of non-synonymous SNVs (NS-SNVs) and gene deletion. The clonal variants were putatively acquired in tumor-initiating events. Indeed, we found that they include hotspot driving mutations (Kras(G12D), Gnaq(Q209L)) (Figure 2), Cdkn2a deletion, and gain in chromosome 6 (containing proto-oncogenes Met, Braf, Mitf, Ret, Kras, etc.) (Figure S10), which can trigger the transformation of melanocytes.

   The subclonal variants are defined as those shared by two or more sublines in a clade. Their number in each node of the tree is much smaller than the number of private variants in individual sublines (Fig. 2a in the manuscript; please also see the figure below). When considering only NS-SNVs, the number of mutated genes was often too few to generate significant results in enrichment analysis. Also, the number of deleted genes is also very small either at subclonal or private levels. Therefore, we performed pathway enrichment analysis on the NS-SNV-carrying genes of each clade, which included both subclonal and private variants within the clade.

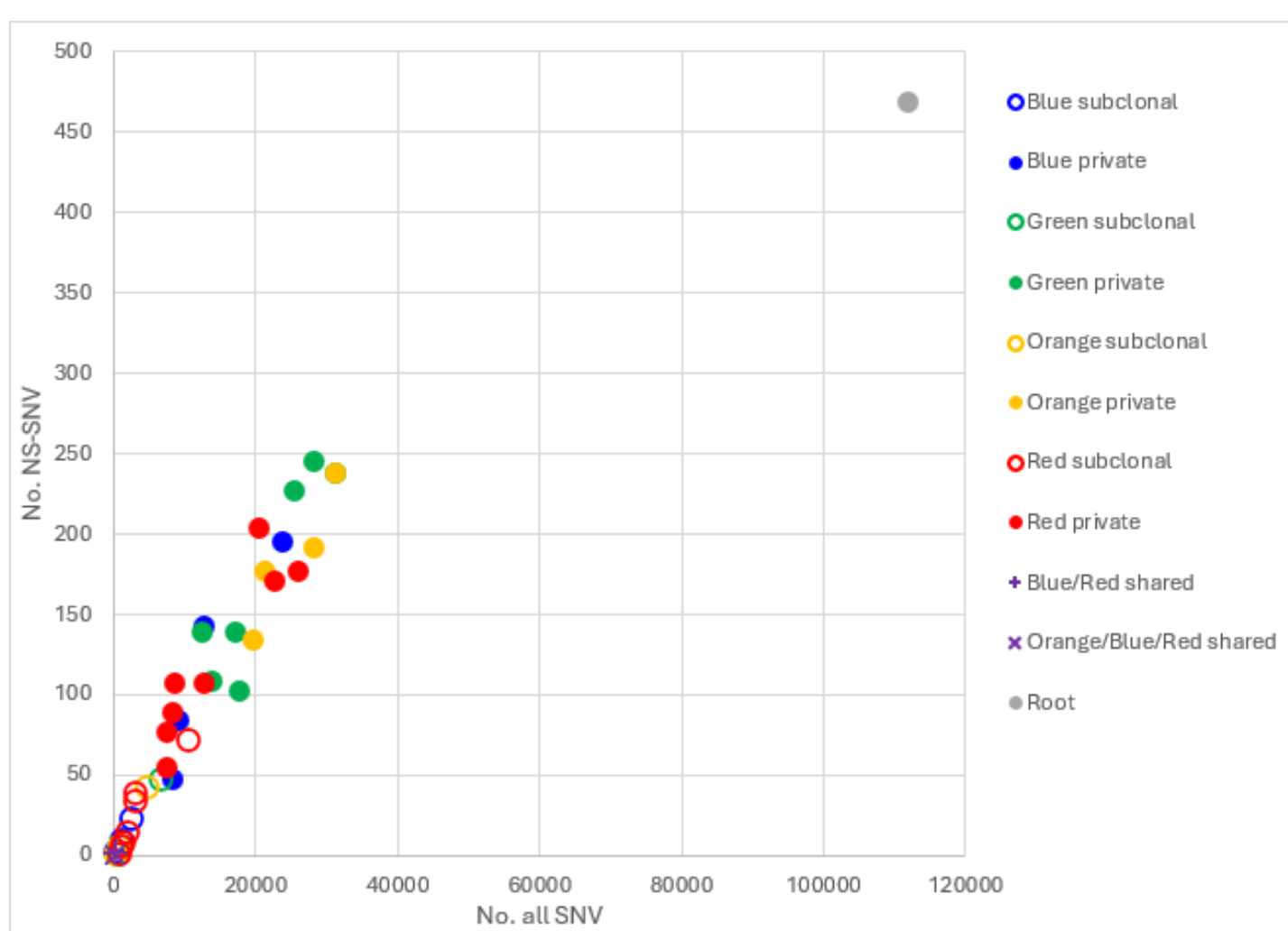


Number distribution of NS-SNVs vs. all SNVs across the root (clonal, gray circle), branch nodes (subclonal, open circles), and leaves (private, filled circles) in the tree of Fig. 2 in the manuscript.

The subclonal and private variants in individual clades (Green, Orange, Blue, and Red) did not include hotspot mutations and deletion of tumor suppressor genes. Instead, they occurred in the genes enriched in pathways related to the phenotypes (invasive, proliferative, and hybrid) of the clades. (Figure S7). These enrichment patterns implied that mutations may happen in the actively expressed genes. We have revised the statement and highlighted these observations in the last two paragraphs in page 11 as the following; our revisions are in red:

*"Overall, our long-read approach offers us a unique lens to observe the complete spectrum of genomic and epigenomic events at the subclonal level and provided insights into the evolution of the B2905 melanoma (Figure 6). We detected clonal mutations of all types that affected genes known to contribute to melanoma initiation, including hotspot SNVs in Kras and Gnaq, SNVs in frequently mutated sites of Sf3b1 and Tfap2a, homozygous deletion of Cdkn2a, and amplifications of Met, Braf, Mitf, Kras, Cdk4, and Erbb3. Importantly, CDKN2A deletion [67] (Cancer cell (2018), 34:56–68), amplification of human chromosome 7 (containing MET and BRAF) [68] (Neoplasia (2001), 3:245–254), and copy number gain of MITF (Nature (2005), 436:117–122) frequently happened during human melanoma progression. We observed a substantial proportion of UV damage signature on the clonal level, but not on subclonal levels; confirming UV damage as a key initiation event [69], but unlikely the driver of lineage differentiation.*

*On the subclonal level, SNVs occurred in genes enriched in pathways associated with the typical phenotypes of the clades. For example, cell proliferation-related pathways in Green and Orange clades, more neural development-related pathways in blue clade, and more cell migration-related pathways in red clade (Figure S7). These enrichment patterns implied that mutations may happen in the actively expressed genes [70] (Science (2018), 362:1898)."*

2. Please well define placed variants vs unplaced variants.

   We have added the following definition:
   "We thus developed a tool called TreeHarmonizer that (i) places variants on the branches of a phylogenetic tree and (ii) identifies variants in disagreement with the tree (we will refer to the corresponding variants as placed and unplaced, respectively). "

3. Figure 3: Please well define different types of SVs (BND, DEL, DUP, INS and INV) in Fig 3b and 3c.

   We thank the reviewer for this comment - we have added definitions for the different types of SVs in the captions of Figure 3.

Remarks on code availability

1. On the TreeHarmonizer GitHub README file, detailed installation pipeline is available. However, there is no instruction on how to run the tool. Especially in the 'Quick Run' section, no detailed instruction on how to run the demo data and how to understand the output file.

   We have now substantially improved the TreeHarnomizer code repository and manual to make sure it is easily applicable to other similar datasets. We also made sure that all relevant tools (including padfoot) developed by our group are now publicly available.

## Reviewer #4

This manuscript presents a Nanopore dataset of single-cell-derived tumor sublines, and the pipeline of variant calling (including CNAs, SVs, and CNAs) and phylogeny reconstruction. Additionally, a new method TreeHarmonizer is developed to place all types of variants on a phylogenetic tree. Mutation signatures, relative evolution rates, and methylation states are analyzed along the phylogenetic tree. This dataset is a valuable resource to study evolution. However, I have the following concerns regarding the computational analysis.

Major Comments

1. Previous methods have been developed to reconstruct phylogeny using SNV, SV, and CNA (e.g., TUSV-ext reconstructs phylogenetic using all three modalities, SCARLET and Condor use SNVs and CNAs). Additionally, SCsnvcna places SVs on an existing phylogenetic tree with CNAs information; Meltos places SVs on a SNV-based phylogenetic tree and further refines the tree. Despite of these methods that can achieve similar results, the performance of TreeHarmonizer is not evaluated or benchmarked. References: TUSV-ext, SCARLET, Condor, SCsnvcna, Meltos

   The reviewer is correct that there are several existing methods that are conceptually similar to TreeHarmonizer, as they jointly consider SNVs, SVs and CNAs phylogenetic placement. However, all these methods seem to have practical limitations that make it difficult to apply them to the analysis of single-cell derived subclones. Overall, these alternative methods fall into two categories: (i) those designed for a different type of input (e.g. multi-sample bulk data) and thus are not applicable here or (ii) those designed for much smaller inputs, e.g. exome or targeted sequencing, and thus do not scale for whole-genome analysis.

   Specifically for the methods mentioned by the reviewer: TUSV-ext and Meltos were designed for multi-regional bulk sequencing samples as input, and thus are solving a clonal deconvolution problem. In our experimental setup, our samples already represent distinct individual clones, which requires a different approach. SCsnvcna requires a CNV tree inferred from an orthogonal biological dataset (for example, a separate set of cells) - which is also not applicable to our scenario since the number of subclones is relatively small, compared to a number of analyzed cells in a single-cell experiment.

   In contrast, SCARLET and ConDoR were designed for single-cell input, and in principle are applicable to our data since each sample is a single-cell derived subclone. In this revision we examined the possibility of running SCARLET and ConDoR for our dataset. In our dataset, there were ~450k unique SNVs among all sublines, ~270k of which are phylogenetically informative. These were filtered down to ~10.8k high quality SNVs (as described in Methods). In contrast, SCARET and ConDoR were originally benchmarked on much smaller sets of variants, e.g. up to 100 SNVs. Therefore, in the updated manuscript we have profiled the scaling of these tools on increasing quantities of variants for all 23 sublines. We show that ConDoR scales exponentially, where even running on 500 variants and k=1 (acceptable losses under the k-dollo model) would take approximately 49,000 hours, thereby rendering it functionally impossible to run on our data with any reasonable sampling. Running on any higher value of k further increases the computation time. SCARLET also scales badly, specifically subexponentially, where running on only 2000 variants would take almost a month of computation time. The complete runtime analysis and discussion has been added to a new Supplementary Section S4 and Supplementary Figure S29.

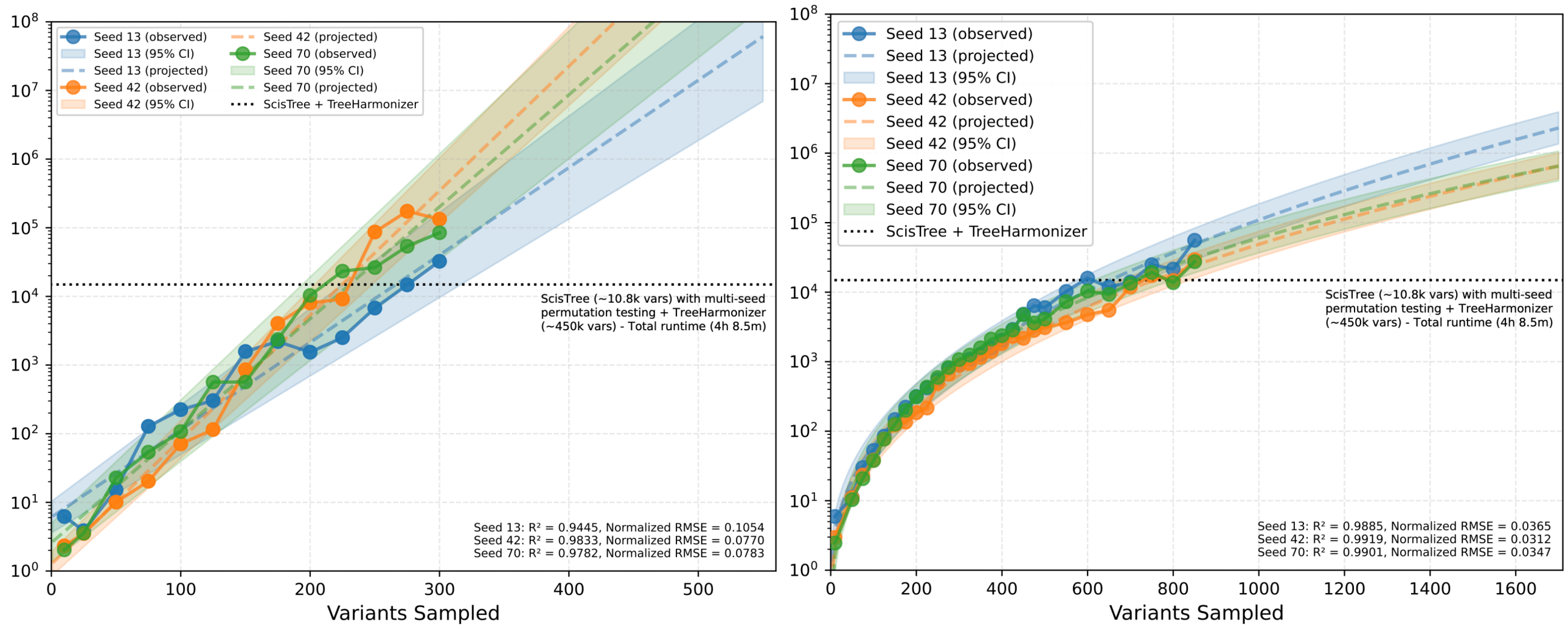


a) ConDoR Benchmarking

b) SCARLET benchmarking

2. I'm confused about the following details of TreeHarmonizer method.
   (i) The order of placing CNAs, SVs, and SNVs matters, as the reasoning of SCARLET and Condor papers. What's the order of placing these variants in TreeHarmonizer?

   In both SCARLET and ConDoR the tree inference is performed simultaneously with variant placement. TreeHarmonizer, in contrast, does not infer the tree; rather, the tree is already inferred using SNVs by a method of choice (in our case ScisTree). TreeHarmonizer assigns / corrects the single nucleotide mutational placements on the given tree using the FN rate of the most optimal tree, deletions found by an SV caller (in our case Severus), and copy number alteration calls on individual (single cell derived) sublines (by Wakhan) - which are partly based on the SV calls. In summary, SNV and SV calls are made first, independently, then CNA calls, then finally, TreeHarmonizer is run afterwards. TreeHarmonizer places SNVs, SVs, and CNAs independently. While SV and CNA calls are not intrinsically independent, assigning causality and dependence is a very difficult problem that no existing method accomplishes, and remains an open problem that we are working towards solving.

   We have now added the discussion above to the new supplementary section S4.

   (ii) After simplifying the CNAs into gains and losses, does TreeHarmonizer consider the potential overlapping with SVs?

   TreeHarmonizer does not currently consider the potential association/overlapping with SVs after the simplification of CNAs into gains and losses. As it stands, direct association of SVs with CNAs is still an open problem. We are currently working on developing a model that can handle such overlapping scenarios and hope to incorporate it into TreeHarmonizer in future studies.

   We have added the clarification above to the Methods section "TreeHarmonizer algorithm".

   (iii) The assignment of overlapping internals and merging of CNA placement is very confusing. What are the underlying assumptions/justifications underlying these options? Are the refinement of a set of overlapping intervals of CNAs viewed as independent CNA events?

The current form of assignment of overlapping intervals and merges is largely a consequence of the fact that defining a CNA span as a single "event" (caused by an SV) is a very difficult open problem.

The current assignment method follows the assumption that if a particular CNA span is shared by multiple sublines within a single subtree, that span has been amplified or lost earlier in time, and thus being subclonal or clonal in nature. We avoid referring to these spans as "events" as that would be inaccurate to do so without assigning causality to known SVs. To do such placements, we traverse the tree starting from the leaves, and find all such overlaps between multiple sublines in a subtree. Similarly to how we handle SNVs and SVs, we allow for a ~15% false negative rate to model possibly missed CNA calls. This may result in multiple "passing" candidate spans at the same subtree (with slightly different sets of sublines) that have partly or entirely overlapping ranges. As a result, any such overlapping spans that are currently treated separately get union-merged into one large span, with a union of all of the sublines that correspond to it. It is also why, once all possible ranges and overlaps have been determined, the final placement onto the tree is done beginning with the root - where as we go down the tree, if a particular range has already been assigned earlier (higher), it is removed from the current span to be placed, as it is assumed that it happened earlier. Whatever part of the span remains is placed at this current position.

Although this model allows us to place possibly overlapping CNA spans in the tree, we acknowledge that this is still limited as it does not explicitly separate overlapping gains and losses into independent events (defined by SVs). We are working on improving TreeHarmonizer and creating future tools and algorithms that can indeed correspond CNAs with SVs, which will result in more accurate placements.

We have revised the TreeHarmonizer algorithm methods section and highlighted our changes below in red:

*"CNAs were also placed onto the tree using TreeHarmonizer. As we are not currently associating all CNAs with specific SVs, the placement of CNAs onto the phylogeny is not as evolutionarily clear. Multiple independent events may impact the same span, and thus makes it difficult to determine unique, independent, CNA spans, and define them as singular events. In order to simplify the placement problem, we limited the definition of a gain or loss to a change from a baseline of CN=2. Ranges of such gains and losses were extracted per subline from the Wakhan output. The current assignment method follows the assumption that - if a particular CNA span is represented by multiple sublines within a single subtree, that span has been amplified or lost earlier in time, and thus being subclonal or clonal in nature.*

*To do such placements, we traverse the tree starting from the leaves, and find all such overlaps between multiple sublines in a subtree. More specifically, beginning at the leaves of the tree, we consider the following cases in a reverse level-order traversal. Given a range $R_1$ that corresponds to a set of sublines $S_{r1}$, if there exists a $R_2$ (with corresponding set $S_{r2}$) such that $R_1$ and $R_2$ are super or subsets of one another, they are merged into a new range $O_{1,2}$, along with their associated subline sets, $S_{1,2} = S_{R1} \cup S_{R2}$. Any ranges $R_1$ and $R_2$ which only partly overlap are split into three ranges: $R_{rem1}$, $O_{1,2}$ and $R_{rem2}$. Range $O_{1,2}$ is the overlap of $R_1$ and $R_2$, and is associated with each range's corresponding subline sets. Ranges $R_{rem1}$ and $R_{rem2}$ are the remainder of each original range minus the overlap region. As a result, every internal (and truncal) branch now contains all overlapping ranges of their children, and exactly which sublines said overlaps are associated with.*

*Similarly to how we handle SNVs and SVs, we allow for a ~15% false negative rate to model possibly missed CNA calls. Due to not requiring perfect clade completion in order to be considered valid, this may result in multiple "passing" candidate spans at the same subtree (with slightly different sets of sublines) that have partly or entirely overlapping ranges. Thus, per branch, any passing ranges $pR_1$ and $pR_2$ that overlap with each other in any way are merged into a new range $O_{pr1,pr2}$, along with their associated subline sets, $S_{pr1,pr2} = S_{pr1} \cup S_{pr2}$.*

*We now finalize the assignment of ranges per branch with a level-order traversal. As we traverse down the tree, as ranges are assigned we exclude any span that has already been associated with a parent branch (as it is assumed to have been affected earlier). Ranges are treated as mutable objects --- if a part of said range currently being considered was already assigned to a parent, the range is split, and the unassigned remainder is placed at the current branch."*

3. The last section indicates the SNV, SV, and CNA calls across the 23 sublines are curated and can be used to benchmark other tools. However, these SNV, SV, and CNA calls are each generated from a single computational tool without validating using either orthogonal sequencing measurement or using the consensus across multiple computational methods.

   (This response closely follows our response to a relevant Question 10 from the Reviewer 3)

   In the revised manuscript, we have now incorporated several new validation and comparison experiments for all variant types, and highlighted the relevant existing comparisons. We now provide several lines of evidence of high quality of variant calls, primarily based on (i) orthogonal support by other experimental technologies, (ii) orthogonal support by alternative tools and (iii) support by multiple sublines and consistency with evolutionary history. We describe them as the following:

   **For small variant calls**, we compared the SNVs in coding regions called using long-reads, short-read WES and scRNA, and showed the high consistency rates (94% and 67% for WES and scRNA, respectively). This is discussed in Supplementary Figure S23 and Supplementary section S1 (which was moved from the main text in response to another reviewer's comment). In addition, we have now compared the SNVs called by DeepVariant to several other state of the art variant callers (DeepSomatic, Clair3 and ClairS) that support long-read technologies and the ONT R10.4 chemistry. We find that there is high concordance between all four callers on subclonal (variants called in 2 or more but not all sublines) and private variants, and high concordance between all callers except for Clair3 in clonal calls. This is discussed further in a new Supplementary Section S3 and shown in accompanying Supplementary Figures S27a, b and c.

   **For SV and CNA calls**, in this revision we have added a comparison of chromosome-scale rearrangements against the spectral karyotyping (SKY) images, available for 12 sublines (new Supplementary Figure S25). The results are summarized in a new supplementary section S2, illustrating ~90% consistency between large CNAs, interchromosomal SVs and SKY images.

   In addition, we **compared our SV calls against the calls generated by Sniffles2** (the only other long-read SV caller that supports multi-sample mode, however not designed for cancer). We placed the detected SVs from both tools on the tree using TreeHarmonizer. SVs detected by both tools (n = 489) were all consistent with the phylogenetic tree. In contrast, SVs unique to Sniffles2 (n = 1703) were often inconsistent with the tree structure, likely driven by mismapped reads in collapsed duplication

regions (76%) (new supplementary Tables S9, S10). In comparison, most SVs uniquely detected by Severus were supported by the phylogenetic relationships. This analysis suggests that (i) most informative subclonal and clonal SVs detected by Severus were also supported by Sniffles2 and (ii) Sniffles2 generated many likely false-positive calls. This analysis is described further in a new Supplementary Section S3.

**We further compared the CNA calls against another state of the art caller, CNVkit**. The concordance between Wakhan and CNVkit was very high (median Pearson's correlation coefficient between Wakhan and CNVkit profiles = 0.9530, p<1e-300). Exact breakpoints around copy number changes may differ between callers, however. This is due to the fact that Wakhan is able to incorporate precise breakpoint information from SV callers such as Severus, whereas CNVkit does not. We compared the integer copy number calls between the two callers within equal size bins of 50kb, finding a median exact match of 96.70% of bins across all sublines. Furthermore, the standard segmentation method of CNVkit (cbs, circular binary segmentation) is not able to detect smaller, focal amplifications and losses. Other callers, such as PURPLE, rely heavily on the human genome, biases which cannot be changed by the end-user. This analysis is described further in a new Supplementary Section S3.

**For DMR calls,** it is difficult to validate the change in methylation directly, as it would require a costly experiment to generate bisulfide sequencing for each subline. Instead, we looked into the consistency between predicted DMRs and change in gene expression measured by scRNA-seq. Consistent with the general understanding, we found the mean methylation differences between case and control groups at promoter/enhancer regions are negatively correlated with the measured expression differences of the corresponding genes (see Figure 5d). This provides orthogonal validation that the DMRs at functionally interpretable regions have led to expected downstream changes in gene expression.

Overall, this analysis above suggests that SNV, SV, CNA and DMR calls are highly accurate and could be used to trace cancer evolution or designing new benchmarking studies. All comparisons are now described in detail in the new supplementary section S3.

4. In particular, the rate of SNV acquisition is compared to that of SV. This claim requires accurate placement of SVs along the tree. I wonder:

    a. if applying other SV callers, e.g., Sniffle2, whether the observation still holds

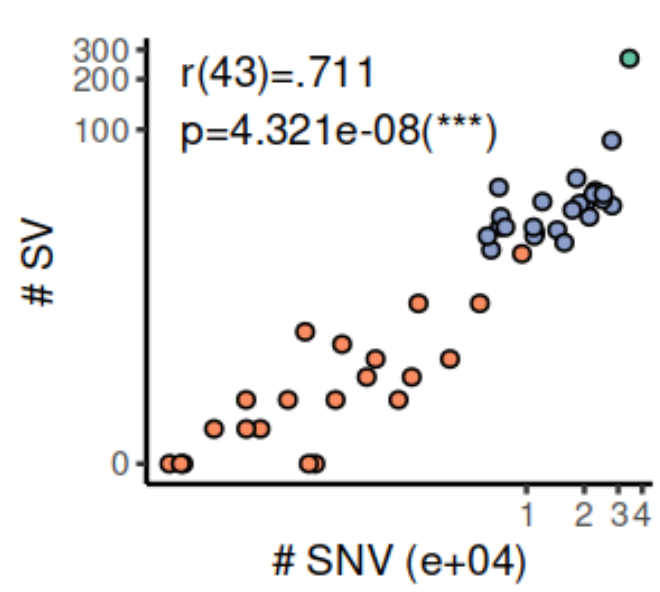


We thank the reviewer for this comment. We have placed SVs reported by Sniffles2, and the observation still holds (r(43)=.711, p=4.321e-08, see figure to the left). We have added this panel to the new supplementary figure S27 new supplementary note S3.

    b. if focusing the total number of SNVs and SVs of each subline and ignoring the tree structure, whether the observation still holds.

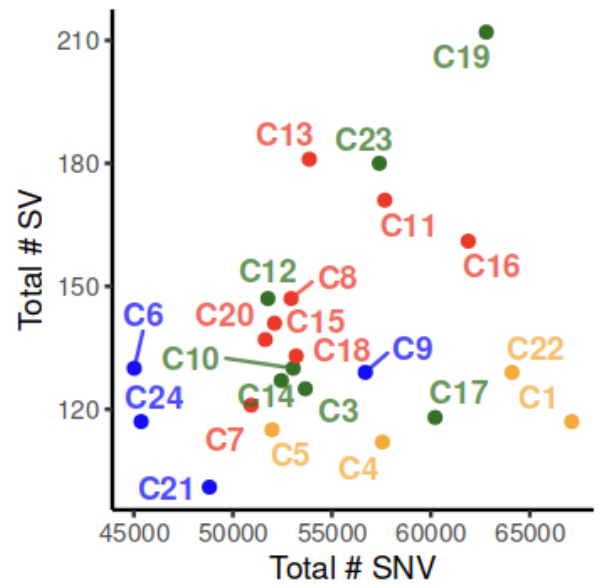


We thank the reviewer for the comment. If focusing on the total number of SNVs and SVs of each subline, while visually there is a weak positive correlation, the observation is not statistically significant (rs(21)=.239, p=.2713), possibly due to the smaller sample size or differences in subclone-specific rates. We have provided a visualization of the correlation on the left, which presents the same information as in Figure S2, but has each subline labelled. We have added a note to the Figure S2 caption: "The correlation between cumulative number of SNVs and SVs was statistically insignificant (rs(21)=.239, p=.2713)".

5. Along the same line, variants inferred from Nanopore sequencing are compared against bWES and scRNA-seq, showing bWES and scRNA-seq miss many calls due to the limitation of sequencing regions. But if focusing on variants that span gene regions (including CNAs, SNVs, and SVs whose breakpoints locate within exons), they should be supported by bWES and potentially also scRNA-seq if involved genes have sufficient expression. I wonder whether this can be used to verify the reliability of Nanopore variant calling.

   In our benchmarks, WES and scRNA-seq were useful to evaluate SNVs in coding regions and large-scale CNAs, but not SVs for the following reasons. First, Figure S23c (previously Figure 7c) shows that bWES and scRNA only covers a small proportion of SV breakpoints, inferred from long-read data. While in principle we could evaluate the overlapping regions, there are additional complications. For WES, it is produced through targeted amplification of exonic regions. Thus, to fully capture an SVs, two SV breakpoints would each need to fall into two different exons (assuming SVs with size larger than 1 exon length) - which would be a very rare event. scRNA can in principle capture breakpoints inside introns if SV results in an in-frame fusion, but it will not capture exact SV coordinates.

   Because of these, we opted not to perform extensive evaluation of WES and scRNA for SV calling. Instead, in the revised paper we now evaluate SV calls by comparing with orthogonal methods (Sniffles2) and SKY chromosome images (as described in our responses to Questions 1 and 3 from this reviewer).

6. Complex SVs are identified by grouping breakend junctions into clusters by Severus. Each complex SV are considered to be one complex event happening at a single time point, hence only involve SVs that are labeled at a single tree branch, while mixing SVs from multiple branches violates the concepts of complex SVs. Does Severus consider the information of phylogenetic tree and SV labeling of tree branches? It's not clear how this approach avoids mixing SVs from multiple branches.

   That's a great question. Currently Severus does not explicitly use phylogenetic tree information when constructing complex SV clusters. Instead, in multisample mode, Severus relies on a consistency constraint: if a complex SV is shared across multiple samples, then *all* junctions within that complex event must be present in exactly the same set of samples. This requirement prevents mixing of junctions that originate from different evolutionary branches. In our dataset, most complex SVs were private. For the smaller set of subclonal complex SVs, we verified that they were in agreement with the structure of the phylogenetic tree.

   We have now added the following clarification to the Methods section "Extending Severus for multi-sample calling":
   *"Severus then groups SVs into clusters to detect complex SVs. If a complex SV is shared across multiple samples, then all junctions within that complex event must be present in exactly the same set of samples".*

7. Fig 5a shows the number of differential methylation regions between two clades of a subtree and the other clades. However, two clades as a group likely doesn't have enough power. I suggest enlarging the number of clades in a group to verify the observation.

   We thank the reviewer for the comment. We'd like to clarify that Figure 5a aims to show the significant positive correlation between the number of SNVs and the number of DMRs accumulated up to a node. We were unable to choose a larger DMR group size because there are fewer such groups in the phylogeny: for group sizes > 2, there are at most 4 such nodes in the phylogeny (see Supplementary Figure S18), which is too small of a sample size to evaluate the existence of a linear correlation. While the number of observations for group size = 2 is still small, we were able to see a significant positive correlation between the number of SNVs and DMRs with a sample size of 7. We hope that, in the future, with a larger data set, we will be able to construct phylogenies with more leaves, and potentially have more internal nodes with the same group size to evaluate such correlations with.

   We added the following clarification to the Methods section "Correlation between the number of DMRs and cumulative SNVs at a branch in the phylogeny":
   *"We note that we were unable to choose a larger DMR group size because there are fewer such groups in the phylogeny: for group sizes > 2, there are at most 4 such nodes in the phylogeny (Supplementary Figure S18), which is too small of a sample size to evaluate the existence of a linear correlation. While the number of observations for group size = 2 is still small, we were able to see a significant positive correlation between the number of SNVs and DMRs with a sample size of 7."*

8. Example DMRs at certain branches are shown in Fig 5. But as there are many branches in the tree, it will be more general to show the number of cancer-associated DMRs (e.g. from OncoDB or other databases) at each branch, to evaluate whether the various aggressiveness of subclones can be generally attributed to the cancer-associated DMRs at each sublineage.

   We thank the reviewer for the valuable suggestion. While OncoDB differential methylation database does not include entries to human melanoma/skin cancer (SKCM), we compared the subclonal promoter/enhancer differential methylation identified in our data to those in human orthologs curated in OncoDB for 17 other cancer types (BLCA, BRCA, CESC, CHOL, COAD, ESCA, HNSC, KIRC, KIRP, LIHC, LUAD, LUSC, PAAD, PCPG, PRAD, THCA, and UCEC).

   We have added new Supplementary Figures S19-S21 showing promoter hypo- or hypermethylation of mouse orthologs from the relevant OncoDB genes. It now shows that many such genes have clade specific methylation. Functional analysis of these methylation changes will be an interesting topic for a follow-up study.

9. A trajectory is a linear sequence of element. So how is "evolutionary trajectory" defined from a phylogenetic tree? From any ordered tree traversal or by ranking some tree distances?

   Indeed our study uses the term *evolutionary trajectory* as the path (i.e. the linear sequence of nodes) on the tumor phylogeny that starts at the root and proceeds sequentially to reach a specific node (typically a leaf). Thus an evolutionary trajectory corresponds to a linear sequence of mutational acquisition events that a particular lineage of tumor cells go through.

   We now added a definition as follows:

*“To evaluate if methylation was driving gradual change in phenotype, we analyzed DMRs within the red clade along the trajectory (a path from phylogeny root to the clade leaves) and focused on (putative) promoter or enhancers of known genes.“*

Minor comments:

1. The meaning of “subclone” is used differently, sometimes referring to the 4 sub lineages, and sometimes referring to the 23 sublines.

   This was indeed confusing, and we would like to follow the suggestions of this comment: using “subclone” to refer to sublineage, and “sublines” to refer to the actual cell lines derived from the parental line (B2905). We now replaced “subclones” with “sublines” when referring to the actual cell lines through the text, including the manuscript title.

2. Page 7 has unbalanced parenthesis in sentence “We identified 65 such clusters involving a total of 298 SVs: of which four were composed of clonal SVs 3e.)”

   We have fixed this typo in the main text.

3. Maybe I missed it, but Fig 5d doesn’t seem to be referred to in the main text.

   We have added a reference to Figure 5d. in the main text.

4. It seems that Venn diagrams are more suitable for the information in Fig 7b, as used in Fig 7f.

   We have changed the visualization to use a Venn diagram like in panel f of what is now Supplementary Figure S23.

Remarks on code availability

The installation and running of the SV and SNV notebooks are smooth. But the path to the CNA notebook doesn't match the example data directory. And I would appreciate more documentation on the output specification.

We thank the reviewer for pointing out that issue, it has been corrected in the notebook. We have updated the TreeHarmonizer repository (https://github.com/KolmogorovLab/TreeHarmonizer) and released the software as a standalone tool in release 1.0.0. Output documentation has been added to the readme. We maintain the presence of paper-specific jupyter notebooks and their respective environment in a directory within the repository for ease of access and replication.

**REVIEWER COMMENTS**

**Reviewer #1 (Remarks to the Author):**

The authors have thoroughly revised the manuscript and have carefully integrated my comments while addressing my concerns. I appreciate that the authors have appropriately included the limitations of the study. My initial concern was the emphasis on certain mutations and structural variants observed in the mouse model that, while highly relevant and important drivers in the animal system, are extremely rare in the clinical presentation of human skin cancer. In addition, the genomic landscapes of melanoma subtypes are distinct and therefore should not be combined into a single group. The overlap in mutational and structural profiles required careful and appropriate consideration. I also acknowledge that Padfoot is now accessible. I appreciate both the major and minor revisions, and I believe this manuscript will serve as a valuable resource for the cancer research community. Congratulations to the authors.

We thank the reviewer for their helpful feedback that helped to improve the manuscript.

**Reviewer #2 (Remarks to the Author):**

The authors have addressed my previous comments.

**Reviewer #2 (Remarks on code availability):**

The standalone software looks good. The installation worked and a README file is available.

We thank the reviewer for their helpful feedback that helped to improve the manuscript.

**Reviewer #3 (Remarks to the Author):**

Thanks for the great revision work! The authors have addressed most of my concerns. However, when I tried to test the tool TreeHarmonizer, I encountered some issues.

We thank the reviewer for their helpful feedback that helped to improve the manuscript. Please see our response on the code issues below.

**Reviewer #3 (Remarks on code availability):**

[1]

```
$ git clone git@github.com:KolmogorovLab/TreeHarmonizer.git
Cloning into 'TreeHarmonizer'...
@@@@@@@@@@@@@@@@@@@@@@@@@@@@@@@@@@@@@@@@@
@@@@@@@@@@@@@@@@@@
```

@ WARNING: REMOTE HOST IDENTIFICATION HAS CHANGED! @
@@@@@@@@@@@@@@@@@@@@@@@@@@@@@@@@@@@@@@@
@@@@@@@@@@@@@@@@@@@
IT IS POSSIBLE THAT SOMEONE IS DOING SOMETHING NASTY!
Someone could be eavesdropping on you right now (man-in-the-middle attack)!
It is also possible that a host key has just been changed.
The fingerprint for the RSA key sent by the remote host is
SHA256:uNiVztksCsDhcc0u9e8BujQXVUpKZIDTMczCvj3tD2s.
Please contact your system administrator.
Add correct host key in /ihome/sliu/shl96/.ssh/known_hosts to get rid of this message.
Offending RSA key in /ihome/sliu/shl96/.ssh/known_hosts:2
Host key for github.com has changed and you have requested strict checking.
Host key verification failed.
fatal: Could not read from remote repository.

Please make sure you have the correct access rights
and the repository exists.

We thank the reviewer for sending us the error messages encountered and apologize for any difficulties in testing.

According to the error message, it seems that the error was caused because of an expired RSA certificate that is stored at the local machine. One way to fix this is to clone via the HTTPS rather than SSH option that Github provides. We have modified the manual to reflect this:

```
git clone https://github.com/KolmogorovLab/TreeHarmonizer.git
cd TreeHarmonizer
conda env create -f th_environment.yml -n tree_harmonizer
conda activate tree_harmonizer
```

Otherwise, the error is likely to do with the known_hosts file configuration on the machine which was used to clone the repository. Github provides the following instructions how to fix this: https://github.blog/news-insights/company-news/we-updated-our-rsa-ssh-host-key/.

[2]
Alternatively, I downloaded the zipped package from GitHub and unzipped it.
I installed the package via Conda, but I still need to manually install the three packages (pandas, ete3, and intervaltree). The README files sound like the 'Via Conda' is recommended, and the manual installation is an alternative. But actually we need to install both.

That is indeed a very unexpected circumstance. The `th_environment.yml` includes all the mentioned packages: pandas, ete3, and interval tree, and tells conda to install those packages. The "Manual installation" is indeed an alternative approach, and the conda installation is preferred. We do recommend copying and pasting directly from the install instructions provided

into the terminal, as that should clone the repository, change the current directory, create the environment, and then activate it in order for it to be used. We have tested the conda install procedure on several machines which have no prior installation history of these conda package or running this tool.

We ask the reviewer to please copy-paste the commands below, one at a time, the following procedure being amended with HTTPS cloning, and the th_environment.yml file updated in the repository with more specific package versions, just in case.

```
git clone https://github.com/KolmogorovLab/TreeHarmonizer.git
cd TreeHarmonizer
conda env create -f th_environment.yml -n tree_harmonizer
conda activate tree_harmonizer
```

If any of these commands return an error or TreeHarnomizer fails to run, then it is possible that the machine has an inconsistent conda configuration. If this is the case, we suggest a fresh installation of conda (we recommend miniforge3: https://conda-forge.org/download/), followed by bioconda (https://bioconda.github.io/#with-conda).

In the case the advice above does not fix the error, we would appreciate it if the reviewer could provide us with what error occurs when attempting to run the code after following the new "via conda" install commands, and we would be happy to help diagnose this issue further.

**Reviewer #4 (Remarks to the Author):**

The revised manuscript addressed most of my previous concerns. But I still recommend improving the clarity of the description of results and methods. Specifically,

We thank the reviewer for their helpful feedback and consideration. Please find our detailed responses below.

1. Fig 2a is a complicated panel, and including the explanation of each part (rectangles, pie charts) in figure legend helps with the understanding of it. Additionally, I couldn't find which part corresponds to the dN/dS color bar.

   Indeed, while we have included descriptions of rectangles (corresponding to variants) and pie charts (corresponding to mutational signature exposures) in the caption, we did not describe where dN/dS values were indicated in the legend. We have now added "Each branch in the phylogeny is colored by the corresponding dN/dS value" to the figure caption.

2. I couldn't find summary statistics about the number of placed and unplaced SNVs and CNAs, but only SVs. But they are important statistics to learn about performances and expectations of TreeHarmonizer.

We thank the reviewer for pointing out the lack of clarity regarding mutational "placement". Our responses for distinct types of mutations are below.

**SVs and SNVs**: Summary statistics for placed SNVs and SVs are currently provided within the section "*Phylogeny-guided harmonization with TreeHarmonizer provides evolutionary timing of genomic variants"* and Supplementary Tables S4 and S5. We have realized that Supplementary Tables S4 and S5 did not in fact include statistics on the unplaced variants, and in the revised version we added a "total unplaced" column. We have also revised the sentence providing these summary statistics for clarity:

*On our mouse melanoma dataset, `TreeHarmonizer` placed high proportions of all SNVs and SVs (96.71% and 95.49%, respectively). Of placed SNVs, 7.89%, 8.38% and 83.73% were clonal, subclonal and private, respectively. Within individual sublines, on average 62.23%, 8.69% and 29.08% of SNVs were clonal, subclonal and private, respectively (Supplementary Table S4). Proportionally, SVs were distributed similarly to SNVs, where 7.60%, 4.94% and 87.46% were clonal, subclonal, and private, respectively. Within individual sublines, on average 65.13%, 5.58%, and 29.29% were clonal, subclonal and private, respectively. The variant allele frequency (VAF) of placed clonal and subclonal variants was distributed around 50%, as expected for heterozygous somatic variants fixed in a given lineage (Supplementary Figure S2c.,d.).*

We have further revised the methods section, *TreeHarmonizer Algorithm*, to provide several numerical specifics for unplaced SNVs. As there are hundreds of thousands of SNVs in our dataset, and just over 14,000 unplaced SNVs, we could not analyze unplaced SNVs individually as done for SVs, but note errors are likely due to artifacts in variant calling, or other unconsidered errors.

*A small proportion of SNVs and SVs remained unplaced after running `TreeHarmonizer`. Just under 3.29% (n = 14,791) of SNVs remained unplaced. Around 24.24% (n = 3,585) of unplaced SNVs fell up to 3 sublines below the threshold limits for clonal placement, and around 29.10% (n = 4,304) of unplaced SNVs were called in 2 or 3 sublines across varying clades, potentially indicative of other unconsidered errors or artifacts in variant calling.*

**CNAs:** We realized that our CNA analysis terminology might have been confusing. We would like to clarify that CNAs are not "placed" as SVs and SNVs are. Rather, specifically for CNAs, TreeHarmonizer produces summary statistics reflecting the percentage of the genome amplified and lost with respect to each branch in the tree. As we currently do not define any CNA spans as individual events nor associate them directly with specific SVs, we changed our terminology in this revision, and now refer to this process as "summarizing" as opposed to "placement". We thus don't have "unplaced" CNAs in this analysis. Although deconvolution of CNA profiles into individual

events would have been ideal, this turned out to be a very difficult computational problem, and we are planning to address it in follow-up work.

We have now amended the use of “placement” of CNAs, and only use the term “summarizing” in the paper **-** specifically in the sections, *Recurrent CNAs in independent lineages suggest parallel adaptation process***,**

*Next, we analyzed the patterns of subclonal and private CNAs. While `TreeHarmonizer` does not directly place CNAs, we noted through profiling the copy number changes with analyses such as in Figure 2.e, and through `TreeHarmonizer` placement of SVs, that similar regions of the genome were amplified by different SV breakpoints in independent parts of the tree (Methods).*

and *TreeHarmonizer algorithm***:**

*CNAs, as opposed to SNVs and SVs, were not placed, but rather TreeHarmonizer produces a percentage summary of amplifications and losses at each branch in the tree. ~~were also placed onto the tree using `TreeHarmonizer`.~~ As we are not currently associating all CNAs with specific SVs, the placement of CNAs onto the phylogeny is not as evolutionarily clear. Multiple independent events may impact the same span, and thus makes it difficult to determine unique, independent, CNA spans and define them as singular events.*

*In order to produce this summary, ~~simplify the placement problem,~~ we limited the definition of a gain or loss to a change from a baseline of CN=2.*
*Ranges of such gains and losses were extracted per subline from the `Wakhan` output. The current assignment method follows the assumption that - if a particular CNA span is represented by multiple sublines within a single subtree, that span has been amplified or lost earlier in time, and thus being subclonal or clonal in nature…*

*…T~~o do such placements, w~~We begin by traversing the tree starting from the leaves, and find all such overlaps between multiple sublines in a subtree. More specifically, beginning at the leaves of the tree, we employ the following in a reverse level-order traversal…*

*…We now finalize the assignment of ranges per branch with a level-order traversal. As we traverse down the tree, as ranges are assigned we exclude any span that has already been associated with a parent branch (as it is assumed to have been affected earlier). Ranges are treated as mutable objects --- if a part of said range currently being considered was already assigned to a parent, the range is split, and the unassigned remainder is ~~placed~~ then assigned at the current branch…*

We also add the following clarification to the *TreeHarmonizer algorithm* section regarding the ranges:

*TreeHarmonizer does not currently consider the potential overlaps with SVs after the simplification of CNAs into gains and losses. As it stands, direct association of SVs with CNAs is still an open problem. We are currently working on developing a model that can handle such overlapping scenarios and hope to incorporate it into TreeHarmonizer in future studies. As such, the individual ranges described in the CNA summarization methodology are not currently user accessible.*

Furthermore, we have amended the caption to Figure 2.a to clarify that CNA numbers refer to amplification and loss percentages:

*a.} Phylogenetic tree with each node labeled with (i) counts of SNV, SV, CNA (impacted base pairs as percentage of the genome)...*

3. From TreeHarmonizer description, it's not very clear what are the exact criteria for placing a variant or leaving it as unplaced. My guess is leaving a variant unplaced is determined by the clade size and FN formula, but it benefits from explicitly state the criteria for a variant being unplaced.

   We thank the reviewer for pointing out this lack of clarity. Indeed, placing a variant just means that it has passed the placement criteria, as determined by the most recent common ancestor, clade size, and the FN formula. Unplaced variants are all those which did not meet the passing criteria. We have amended the results section to clarify this with the following:

   *We thus developed a tool called `TreeHarmonizer` that (i) places variants on the branches of a phylogenetic tree and (ii) identifies variants in disagreement with the tree ~~(we will refer to the corresponding variants as placed and unplaced, respectively)~~. `TreeHarmonizer` assumes the input tree is optimal and does not directly evaluate the validity of the existing topology or change it.*
   *Briefly, `TreeHarmonizer` takes as input (i) a phylogenetic tree and (ii) individual SNV, SV, and CNA calls for every subline. For each unique variant, it evaluates whether the variant is consistent with the input phylogeny based on (i) the most-recent common ancestor (MRCA) of all sublines carrying the variant, (ii) the variant status in all sublines, and (iii) the presence of nearby deletions ~~(Methods)~~. A variant is denoted as "placed" if it passes all of `TreeHarmonizer's` criteria for consistency with the tree and is assigned a branch that it is placed at. All variants that do not pass the criteria at any stage are denoted as "unplaced" (Methods). We denote a variant as clonal, subclonal, or private, if they were placed at the trunk, internal branch, or terminal branch of the tree, respectively (Figure 2.a.).*

   We further add a clarification to the Methods section upon the first use of the term "unplaced":

*A small proportion of SNVs and SVs remained unplaced after running* `TreeHarmonizer`. *A variant is considered unplaced if it did not pass the minimum threshold of sublines required by its clade size and FN rate formula.*

4. The explanation of “trajectory” helps, however, the language is not precise enough, and multiple descriptions are used in different places. E.g., “a path from phylogeny root to the clade leaves”, “phylogenetic order”. In fact, the first description is not precise as one path from root to leaves include a root node, multiple unmeasured intermediate nodes, and a single leaf node, which doesn’t give the order of leaf nodes and makes the description inaccurate.

We thank the reviewer for bringing our attention to this confusing definition. Indeed we have found multiple instances of similar language that refer to slightly different concepts. In the instance for when we refer to the order of the leaf nodes, - for example in DMRs analysis within the red clade - we now refer to this as **branching order**. A leaf’s branching order is determined by the number of segments traversed from the root of the tree to this node. The branching order within the red clade, we find, also directly agrees with each of the subline’s survival time, as shown in Figure 2.b.. In those groups of sublines where there is no inherent order, as they are the same distance from the starting node, we order those based on the survival time, namely the pairs C18 and C15, and C7 and C20. Thus, we have a resulting ordering of sublines as they differentiate, here being C13, C18, C15, C11, C16, C8, C7 and C20.

We have now revised this section as follows:

“*While the red clade was distinct from the rest of the phylogeny in terms of survival, it also showed the gradual changes of genotype and phenotype within the clade. Specifically we considered the ordering of the 8 sublines in the red clade with respect to their distances to the root, referred to as branching order (Figure 2.a.). These sublines followed a linear differentiation pattern, instead of a more balanced branching pattern in other clades. Further, survival monotonically improved along the path from the root of the phylogeny towards leaves C7/C20, $\tau(5)=1$, $p<.001$ (Methods, Figure 2.a.). To evaluate if methylation was driving gradual change in phenotype, we analyzed DMRs within the red clade with respect to the branching order (the relative ordering of subline pairs C7 and C20, as well as the pair C15 and C18 along with leaf C11 which are equidistant to the root, was determined based on survival time) and focused on (putative) promoter or enhancers of known genes.*”

*We found 146 genes whose (putative) promoter or enhancer overlap with regions displaying monotonic change in methylation rate along this resulting subline ordering, ~~evolutionary trajectory~~ which is found to be significantly more than what is expected by chance via a permutation test on the order of the sublines within the red clade ($p=.021$, Methods).*

In addition, we found several **other instances of the term trajectory** in the Discussion section, *Systematic analysis of multiple variation types provides insights into B2905 lineages differentiation*, which were unnecessarily confusing, and were removed.

- *Furthermore, our phylogenetic approach enabled the comparisons among different ~~evolutionary trajectories of~~ major clades that resulted in observed phenotypic heterogeneity….*
- *The red clade ~~trajectory~~ -- showing faster growth in immunocompetent hosts and higher ICB resistance -- stood out in…*
- *…expression changes. This suggests that DMRs may drive a gradual phenotypic ~~trajectory~~ change…*

Finally, we have amended references to the term evolutionary trajectory within Methods, to reference the established phylogenetic term, **branching order**:

- *Discovering key regions where changes in methylation is monotonic along the ~~evolutionary trajectory~~ branching order.*
- *…lastly C7 and C20. These groupings were chosen as they reflect cuts along the linear ~~evolutionary trajectory~~ branching order in the red clade. The union of the 4 sets of DMRs were then taken, merging overlapping intervals….*
- *Permutation test on the number of genes associated with monotonic methylation changes along the ~~evolutionary trajectory~~ branching order*
- *We assessed the statistical significance of the number of genes we discover to be associated with monotonic methylation changes along the ~~evolutionary trajectory~~ branching order in the red clade…*

5. In TreeHarmonizer description, what's the difference between "a deletion set" and "a loss set" in the inputs?

This was indeed confusing. The "a deletion set" terminology refers to ranges explicitly called as "deletions" by an SV caller, such as Severus. The "a loss set" terminology refers to those spans which are considered losses via copy number calling, with a caller such as Wakhan.

We have amended the text to no longer use said terms, and instead simply describe which information is used from the SV and CNA call sets. The amended text below also reflects our response to reviewer question 6.

*"By default, it takes a phylogenetic tree; SNV, SV and CNA call sets; and an estimated variant call FN rate; ~~an estimated variant call FP rate; a deletion set; and a loss set.~~ For our data, the estimated FN rate is 15%, and estimated FP rate is 0.001%, as determined by the phylogeny creation in the previous section. The SNV call set are DeepVariant-called somatic SNVs per subline. The SV call set are Severus-called somatic SVs per*

*subline. From the SV call set, TreeHarmonizer uses SVs labeled as deletions by the caller. The CNA call set is CNA calls from the tool, Wakhan. From the CNA call set, the TreeHarmonizer uses regions labeled as copy numbers 0 or 1."*

6. TreeHarmonizer requires "an estimated variant call FP rate" as input, but is it used in later algorithm quantitatively? I only see the assumption "Given the low a priori FP rate (0.001%)", but what if it is not low enough?

   We thank the reviewer for pointing out this issue. As of right now, TreeHarmonizer does not require or take an input FP rate. We have amended the description to reflect this.

   *"By default, it takes a phylogenetic tree; SNV, SV and CNA call sets; and an estimated variant call FN rate; ~~an estimated variant call FP rate; a deletion set; and a loss set.~~*

   As the a priori FP rate for this data and phylogenetic tree was so low, it was not necessary to implement its usage for this work. However, such a parameter is being added to TreeHarmonizer in the future to allow for better generalizability to other datasets. How such a parameter would work is not yet finalized, but the tool would likely look to the reads for more criteria and whether to accept a variant as a true positive or not.

   We have added the following to the text to reflect this future development:

   "*Given the low a priori FP rate (0.001%) for our input, we assume that the existing variant set used to compute the MRCA is correct, and do not consider possible permutations of variant status for sublines. Future versions of TreeHarmonizer will allow for an FP rate parameter for better data generalizability.*"

7. Although the numbers of placed CNAs are shown in Fig 2a, the example CNAs in Fig 4 don't include the information about where they are placed. Given the complexity of interval overlapping among sublines, it's worth marking the overlapping intervals (O_{pr1, pr2}) and union of sublines (S_{pr1,pr2}) in the examples, as well as which branch they are placed in the tree.

   We realized that the figure labeling might have been confusing. We'd like to clarify that the CNA numbers shown in Fig2a reflect percentages of the genome, rather than counts (like those for SNVs, SVs, or DMRs). They merely show the percentage of the genome impacted by amplification/loss among the sublines below the branch. Therefore, there is no direct correspondence between the numbers in Fig2a. and the example CNAs in Fig 4. We have clarified that the CNA numbers displayed in Fig2a. correspond to "(impacted base pairs as percentage of the genome)" in the figure caption.

   With respect to the interval labeling request, it is difficult to implement for two reasons. First, as we described above, TreeHarmonizer currently does not partition CNA profiles

into individual events. Even though the examples from Figure 4 could be labelled manually, another challenge is that the figure in fact highlights CNAs that are **either** in agreement, or in disagreement with the tree (representing examples of parallel CNAs). We feel that packing this information into a figure might make it very complex to interpret. Thus, we prefer to label the state of individual leaves, and keep the detailed description in the corresponding results section.

**Reviewer #4 (Remarks on code availability):**

This version of code has a much better README.

**REVIEWERS' COMMENTS**

Reviewer #3 (Remarks to the Author):

I would greatly appreciate the authors' thorough revisions. The GitHub tool has been significantly improved, and I was able to successfully install the software via Conda and run the demo data. Overall, I recommend the manuscript for publication.

Reviewer #3 (Remarks on code availability):

The GitHub tool has been significantly improved, and I was able to successfully install the software via Conda and run the demo data.

Reviewer #4 (Remarks to the Author):

The revised manuscript addressed all of questions, and it reads clearly now!

Reviewer #4 (Remarks on code availability):

The github has good documentation and I don't have further comments.

We thank both reviewers for their supportive and helpful feedback.
